# Development of a Neurotensin-Derived ^68^Ga-Labeled PET Ligand with High In Vivo Stability for Imaging of NTS_1_ Receptor-Expressing Tumors

**DOI:** 10.3390/cancers14194922

**Published:** 2022-10-08

**Authors:** Lisa Schindler, Jutta Moosbauer, Daniel Schmidt, Thilo Spruss, Lukas Grätz, Steffen Lüdeke, Frank Hofheinz, Sebastian Meister, Bernd Echtenacher, Günther Bernhardt, Jens Pietzsch, Dirk Hellwig, Max Keller

**Affiliations:** 1 Institute of Pharmacy, Faculty of Chemistry and Pharmacy, University of Regensburg, Universitätsstraße 31, D-93053 Regensburg, Germany; 2 Department of Nuclear Medicine, University Hospital Regensburg, Franz-Josef-Strauß-Allee 11, D-93053 Regensburg, Germany; 3 Section for Central Animal Laboratories, University of Regensburg, Franz-Josef-Strauß-Allee 11, D-93053 Regensburg, Germany; 4 Institute of Pharmaceutical and Biomedical Sciences, Johannes Gutenberg-University Mainz, Staudinger Weg 5, D-55128 Mainz, Germany; 5 Institute of Pharmaceutical Sciences, Albert-Ludwigs-University of Freiburg, D-79104 Freiburg, Germany; 6 Department of Positron Emission Tomography, Institute of Radiopharmaceutical Cancer Research, Helmholtz-Zentrum Dresden-Rossendorf, Bautzner Landstraße 400, D-01328 Dresden, Germany; 7 Department of Radiopharmaceutical and Chemical Biology, Institute of Radiopharmaceutical Cancer Research, Helmholtz-Zentrum Dresden-Rossendorf, Bautzner Landstraße 400, D-01328 Dresden, Germany; 8 Division of Immunology, LIT—Leibniz Institute for Immunotherapy, University Hospital Regensburg, Franz-Josef-Strauß-Allee 11, D-93053 Regensburg, Germany; 9 Faculty of Chemistry and Food Chemistry, School of Science, Technische Universität Dresden, 01069 Dresden, Germany

**Keywords:** positron emission tomography, tumor imaging, PET ligand, neurotensin NTS_1_ receptor, NT(8-13)

## Abstract

**Simple Summary:**

Cancer diagnostics based on molecular imaging techniques such as positron emission tomography (PET) requires radiolabeled tracers, which are taken up by tumors. As the neurotensin receptor type 1 (NTS_1_R) is present in certain malignant tumors, radiolabeled NTS_1_R ligands can serve as molecular tools for tumor imaging. A straightforward approach for developing NTS_1_R PET ligands would be the preparation of fluorine-18 or gallium-68 labeled analogs of the peptide neurotensin. However, as neurotensin derivatives are prone to enzymatic cleavage, structural modifications are needed to prevent peptide degradation while retaining NTS_1_R affinity. Applying a new strategy for peptide stabilization, it is possible to develop a peptidic gallium-68 labeled NTS_1_R PET ligand with high in vivo stability and high NTS_1_R affinity. Investigations of the PET ligand in mice with subcutaneous NTS_1_R-positive tumors revealed the NTS_1_R-mediated visualization of the tumor. Future developments, such as NTS_1_R PET ligands with improved biodistribution, will benefit from these results.

**Abstract:**

Overexpression of the neurotensin receptor type 1 (NTS_1_R), a peptide receptor located at the plasma membrane, has been reported for a variety of malignant tumors. Thus, targeting the NTS_1_R with ^18^F- or ^68^Ga-labeled ligands is considered a straightforward approach towards in vivo imaging of NTS_1_R-expressing tumors via positron emission tomography (PET). The development of suitable peptidic NTS_1_R PET ligands derived from neurotensin is challenging due to proteolytic degradation. In this study, we prepared a series of NTS_1_R PET ligands based on the C-terminal fragment of neurotensin (NT(8–13), Arg^8^-Arg^9^-Pro^10^-Tyr^11^-Ile^12^-Leu^13^) by attachment of the chelator 1,4,7,10-tetraazacyclododecane-1,4,7,10-tetraacetic acid (DOTA) via an *N*^ω^-carbamoylated arginine side chain. Insertion of Ga^3+^ in the DOTA chelator gave potential PET ligands that were evaluated concerning NTS_1_R affinity (range of *K*_i_ values: 1.2–21 nM) and plasma stability. Four candidates were labeled with ^68^Ga^3+^ and used for biodistribution studies in HT-29 tumor-bearing mice. [^68^Ga]UR-LS130 ([^68^Ga]**56**), containing an N-terminal methyl group and a *β*,*β*-dimethylated tyrosine instead of Tyr^11^, showed the highest in vivo stability and afforded a tumor-to-muscle ratio of 16 at 45 min p.i. Likewise, dynamic PET scans enabled a clear tumor visualization. The accumulation of [^68^Ga]**56** in the tumor was NTS_1_R-mediated, as proven by blocking studies.

## 1. Introduction

**Figure 1:**
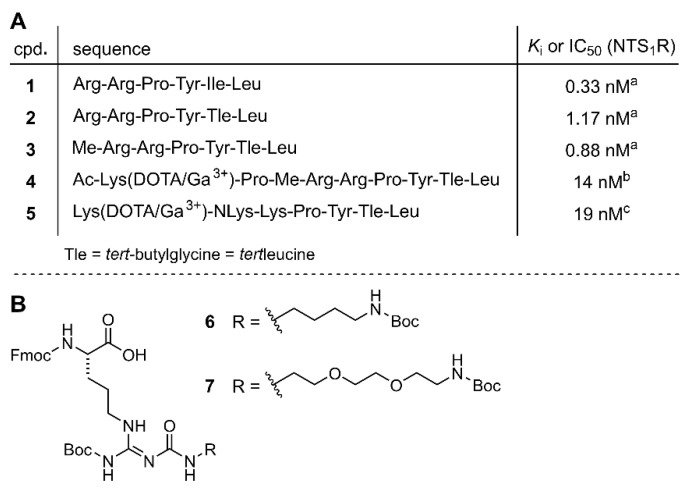
(**A**) Amino acid sequences and NTS_1_R affinities of NT(8-13) (**1**), NT(8-13) derivatives **2** and **3**, and NT(8-13)-derived potential PET ligands **4** and **5**. (**B**) Structures of the reported arginine building blocks **6** and **7** (Keller et al. [39]) which were applied in SPPS for the preparation of amino-functionalized precursor peptides. ^a^ *K*_i_ value; Schindler et al. [40], ^b^ IC_50_ value; Alshoukr et al. [41], ^c^ *K*_i_ value; Maschauer et al. [42].

Neurotensin (NT), a linear 13 amino acid peptide, acts as a hormone in the gastrointestinal tract, regulating, inter alia, motility and mucosal regeneration [1], and as a neurotransmitter and neuromodulator in the central nervous system, where it is involved, inter alia, in the regulation of body temperature, food intake, blood pressure, nociception, memory, and hormone secretion [2,3,4,5,6,7]. The effects of neurotensin are mainly mediated by the neurotensin receptors 1 and 2 (NTS_1_R, NTS_2_R), members of the family of G-protein coupled receptors. The NTS_1_R has emerged as an interesting target for tumor visualization and therapy due to its overexpression in a variety of tumors such as breast cancer, colorectal carcinoma, and (the prognostically poor) pancreatic adenocarcinoma [8,9,10]. The carboxyterminal hexapeptide of NT (NT(8-13), **1**, Figure 1A) was identified as the biologically active fragment, exhibiting the potency of full-length NT [11,12,13]. Therefore, peptide **1** has previously served as a lead structure for the development of imaging agents addressing the NTS_1_R [14,15,16,17,18,19,20].

^68^Ga- and ^18^F-labeled ligands of cell-surface receptors that are (over-)expressed in malignant tumors are considered useful tools for in vivo cancer imaging by positron emission tomography (PET) [21,22,23,24,25,26,27,28]. ^18^F-labeled PET tracers are advantageous with respect to half-life (about 110 min) and achievable resolution, but require a cyclotron for radionuclide synthesis and usually two or more radiosynthetic reaction steps [29,30]. In contrast, the advantage of ^68^Ga-labeled PET tracers lies in their fast one-step radiosynthesis (incorporation of ^68^Ga in a chelator moiety) and convenient radionuclide accessibility (^68^Ge/^68^Ga-generator); however, these tracers result in lower resolution images and the short half-life (68 min) does not allow a transfer between clinics. The development of PET ligands with favorable properties (e.g., high receptor affinity, high in vivo stability and appropriate pharmacokinetics) is challenging. The development of peptidic PET tracers, often acting as receptor agonists, can be convenient with respect to high target affinity and attachment of the label [31,32,33], but high proteolytic stability in vivo might not be easily achieved [33,34]. With respect to NTS_1_R PET ligands, two main strategies have been pursued [25], i.e., investigations of peptidic agonists and of non-peptidic antagonists [35,36,37]. To date, reported ^18^F- and ^68^Ga-labeled NTS_1_R antagonists exhibit higher receptor affinities and higher in vivo stabilities compared to peptidic NTS_1_R PET ligands explored with regard to in vivo tumor imaging. However, the pharmacokinetic profile of the antagonists is not well-suited for PET imaging based on short-lived radionuclides [38]. Unlike antagonists, agonist binding induces receptor internalization; thus, peptidic PET ligands potentially allow for a higher tracer uptake in the tumor.

Peptide **1** exhibits a plasma half-life of only a few minutes [40,43]; thus, NT(8-13) analogs require stabilizing structural modifications when intended to be used as tracers for NTS_1_R-targeted tumor imaging. A previously reported approach based on the replacement of amide bonds in the core structure of **1** by triazoles revealed that high affinity of the respective ^177^Lu-labeled analogs could not be combined with high in vitro serum stability [44]. The recent exploration of the introduction of trimethylsilylalanine instead of Ile^12^ or Leu^13^ for the preparation of ^68^Ga-labeled derivatives of **1** resulted only in moderate in vitro plasma stabilities as well [38]. The replacement of Ile^12^ in analogs derived from **1** by Tle^12^ (*α*-*tert*-butyl-Gly) represents one of the most frequently applied modifications to prevent C-terminal degradation [14,15,16,17,18,19,41,42,45,46,47,48,49,50,51,52,53,54], but is insufficient to prevent proteolytic degradation when applied, e.g., to **1** as the only structural alteration (**2**, Figure 1A) due to persisting N-terminal degradation [40]. However, additional N-methylation of either Arg^8^ or Arg^9^ in **1** resulted in excellent in vitro plasma stabilities (e.g., compound **3**, Figure 1A) [40]. 

Among reported ^68^Ga-labeled neurotensin-derived NTS_1_R PET ligands, peptides [^68^Ga]**4** and [^68^Ga]**5** (Figure 1A), and the 1,4,7-triazacyclononane-1,4,7-triacetic acid (NOTA)-conjugated analog of **5** represent the most promising candidates in terms of NTS_1_R affinity and achieved tumor-to-muscle activity ratios. Both peptides contain Tle in position 12, but differ with respect to the modification of the N-terminal segment. Whereas **4** is N^α^-methylated at Arg^8^ and represents an octapeptide, the hexapeptide **5** harbors a peptoid-like moiety at the N-terminus (NLys^8^). For both peptides in vivo stability data were not reported. However, for the ^111^In-labeled analog of **4**, 22% remaining intact tracer in blood plasma 15 min p.i. in mice has been reported [41], and for [^68^Ga]**5** a high in vitro stability in human serum (93% remaining intact tracer after 1 h) has been described [42]. Notably, in **4** and **5**, the 1,4,7,10-tetraazacyclododecane-1,4,7,10-tetraacetic acid (DOTA) chelator is attached to the *α*- or *ε*-amino group of Lys, which represents a common strategy for the conjugation of NT(8-13) analogs with chelating agents [16,19,52,54,55,56,57]. A recently introduced alternative strategy is the labeling of peptides via the side chain of Arg, based on amino-functionalized *N*^ω^-carbamoylated arginines derived from building blocks **6** and **7** (Figure 1B) [39,58,59]. Lately, the incorporation of **6** in the stabilized NT(8-13) core structure (**3**) and the subsequent attachment of a fluoroglycosyl moiety to the carbamoylated arginine side chain afforded an ^18^F-labeled NTS_1_R PET ligand showing high receptor affinity (*K*_i_ of the “cold” ligand = 4.3 nM) and high tumor uptake in vivo [60].

In the present study, we aimed at the development of a peptidic NTS_1_R PET ligand matching up with reported receptor antagonists in terms of NTS_1_R affinity and in vivo stability. For this purpose, *N*^ω^-carbamoylated arginines derived from **6** or **7** were incorporated into **3** or slightly modified analogs of **3**, optimized with respect to plasma stability, followed by the attachment of a DOTA chelator to the modified arginine side chain and insertion of stable (“cold”) Ga^3+^ or radioactive ^68^Ga^3+^. The potential NTS_1_R PET ligands (“cold” compounds) were characterized with respect to NTS_1_R and NTS_2_R affinity, and plasma stability. For selected peptides, the ^68^Ga-labeled analogs were prepared and studied in vivo in tumor-bearing mice. 

## 2. Materials and Methods

### 2.1. General Experimental Conditions

Solvents and buffer components, all purchased from commercial suppliers, were of analytical grade. Gradient grade MeOH for HPLC was obtained from Merck Chemicals (Darmstadt, Germany) and gradient grade MeCN for HPLC was from Sigma-Aldrich (Taufkirchen, Germany) or Merck. *N*,*N*-Diisopropylethylamine (DIPEA, 99%) and (*R*)-2-(Boc-amino)-3-(4’-fluoro-[1,1’-biphenyl]-4-yl)propanoic acid (**24**) were obtained from ABCR (Karlsruhe, Germany). HCOOH and K_2_CO_3_ were from Roth (Karlsruhe, Germany) and 1 M HCl was from VWR Chemicals (Fontenay-sous-Bois, France). Anhydrous *N*,*N*-Dimethylformamide (DMF) (99.8%), 1,1,1,3,3,3-hexafluoro-2-propanol (HFIP), Ga(NO_3_)_3_ hydrate, 7-methyl-1,5,7-triazabicyclo [4.4.0]dec-5-ene (MTBD), methyl-4-nitrobenzenesulfonate, 2-mercaptoethanol, n-octanol and 1-methyl-d-Trp were purchased from Sigma-Aldrich. DMF (for peptide synthesis, packed under nitrogen, code D/3848/PB17), 1-methylpyrrolidin-2-one (NMP) (for peptide synthesis, nitrogen flushed), anhydrous NMP (99.5%), CH_2_Cl_2_ and 1-hydroxy-1*H*-benzotriazole (HOBt) hydrate were obtained from Acros Organics/Fisher Scientific (Nidderau, Germany). When used for the coupling of non-standard Fmoc-amino acids (SPPS), HOBt hydrate, containing up to 3% water, was dried using a lyophilizer. 4-[(Boc-amino)methyl]-3-fluoro-benzoic acid (>95%) (**27**) was purchased from Activate Scientific (Prien am Chiemsee, Germany) and Boc-*ε*-aminocaproic acid succinimidyl ester (**30**) was purchased from Bachem (Bubendorf, Switzerland). DOTA-tris(*t*Bu)ester succinimidyl ester (**13**) was from CheMatech (Dijon, France). Trifluoroacetic acid (TFA) and absolute EtOH were obtained from Honeywell (Seelze, Germany). Collidine, 2-nitrobenzenesulfonylchloride and 1,8-diazabicyclo [5.4.0] undec-7-ene (DBU) were from Alfa Aesar/ThermoFisher (Heysham, UK). Piperidine and *N*,*N*,*N*′,*N*′-tetramethyl-*O*-(1*H*-benzotriazole-1-yl)-uronium hexafluorophosphate (HBTU) were purchased from Iris Biotech (Marktredwitz, Germany). Deuterated solvents were obtained from Deutero (Kastellaun, Germany). Bovine serum albumin (BSA) was purchased from Serva (Heidelberg, Germany). Oxyma pure, *N,N’*-diisopropylcarbodiimide (DIC), H-Leu-2-ClTrt resin (loading: 0.79 mmol/g), Fmoc-N-Me-Arg(Pbf)-OH, Fmoc-Pro-OH, Fmoc-Ile-OH and Fmoc-Tle-OH (Fmoc-l-*α*-*tert*-butylglycine) were from Merck Biosciences (Schwalbach am Taunus, Germany). Cl-2-ClTrt resin (loading: 1.6 mmol/g), Fmoc-l-allo-Ile-OH, Fmoc-Deg-OH, Fmoc-l-cPrGly-OH and Fmoc-*β*,*β*-diMe-Tyr(*t*Bu)-OH (rac) were obtained from Iris Biotech. Fmoc-Arg(Pbf)-OH and Fmoc-Tyr(*t*Bu)-OH were from Iris Biotech or Carbolution (St. Ingbert, Germany). Fmoc-*β*-cyclopropyl-l-Ala-OH, Fmoc-*β*-cyclopentyl-l-Gly-OH, Fmoc-*α*-methyl-l-Leu-OH and (*S*)-Fmoc-*α*-ethyl-Ala-OH were from ABCR, and Fmoc-(*S*)-2-amino-2-cyclobutylacetic acid was from Merck Chemicals. Ultrapure 4-(2-hydroxyethyl)-1-piperazineethanesulfonic acid (HEPES) was from Gerbu (Heidelberg, Germany). Peptide **1** (tris(hydrotrifluoroacetate)) was purchased from SynPeptide (Shanghai, China). The syntheses of reference peptides **2** [40] and **3** [40], arginine building blocks **6** [39] and **7** [39], NT(8-13) derivative **10** [60] and radioligand [^3^H]UR-MK300 [39] have been described elsewhere. Millipore water was used throughout for the preparation of buffers, stock solutions and HPLC eluents. 1.5- and 2-mL polypropylene reaction vessels with screw cap (in the following referred to as “reaction vessel with screw cap”) from Süd-Laborbedarf (Gauting, Germany) were used for the preparation and storage of stock solutions, and for small-scale reactions. 1.5- or 2-mL polypropylene reaction vessels (in the following referred to as “reaction vessel”) from Sarstedt (Nümbrecht, Germany) were used for the preparation of serial dilutions, for the synthesis, determination of the distribution coefficient and biodistribution measurements of ^68^Ga-labeled PET tracers and for the determination of stabilities in plasma. For the evaporation of solvents in 1.5- or 2-mL reaction vessels, a Savant Speed-Vac Plus SC110A vacuum concentrator (Thermo Fisher Scientific, Waltham, MA) was used. NMR spectra were recorded on a Bruker Avance 600 instrument (^1^H: 600 MHz, ^13^C: 151 MHz) (Bruker, Karlsruhe, Germany) at 300 K. The spectra were calibrated based on the solvent residual peaks (^1^H-NMR: DMSO-*d*_6_: δ = 2.50 ppm; ^13^C-NMR: DMSO-*d*_6_: δ = 39.52 ppm). ^1^H-NMR data are reported as follows: chemical shift δ in ppm (multiplicity (s = singlet, d = doublet, m = multiplet, br s = broad singlet), integral, coupling constant *J* in Hz). High resolution mass spectra (HRMS) were acquired with an Agilent 6540 UHD Accurate-Mass Q-TOF LC/MS system coupled to an Agilent 1290 HPLC system (Agilent Technologies, Santa Clara, CA), using an ESI source. Analyses were performed using the following LC method: column: Luna Omega C18, 1.6 μm, 50 × 2.1 mm (Phenomenex, Aschaffenburg, Germany), column temperature: 40 °C, flow: 0.6 mL/min, solvent/linear gradient: 0–4 min: 0.1% aqueous HCOOH/0.1% HCOOH in MeCN 95:5–2:98, 4–5 min: 2:98. Preparative HPLC was performed with a system from Knauer (Berlin, Germany) consisting of two K-1800 pumps and a K-2001 detector (compounds **8**, **9**, **11**, **12**, **14–21**, **23**, **25**, **26**, **28**, **29**, **31–57**), or a Prep 150 LC System from Waters (Eschborn, Germany) consisting of a 2545 binary gradient module, a 2489 UV/visible detector, and a Waters Fraction Collector III (compound **22**). A Kinetex-XB C18, 5 μm, 250 mm × 21 mm (Phenomenex) or a Gemini-NX C18, 5 μm, 250 mm × 21 mm (Phenomenex) served as RP-columns at a flow rate of 20 mL/min. Mixtures of 0.2% aq TFA (A1) and acetonitrile (B1), or 0.1% aq TFA (A2) and B1 were used as mobile phase. A detection wavelength of 220 nm was used throughout. Collected fractions were lyophilized using an Alpha 2–4 LD apparatus (Martin Christ, Osterode am Harz, Germany) or a Scanvac CoolSafe 100-9 freeze-dryer (Labogene, Allerød, Denmark) both equipped with a Vacuubrand RZ 6 rotary vane vacuum pump. Analytical HPLC analysis of compounds **8**, **9**, **11**, **12**, **14–23**, **25**, **26**, **28**, **29** and **31–57** was performed with a system from Agilent Technologies consisting of a 1290 Infinity binary pump equipped with a degasser, a 1290 Infinity Autosampler, a 1290 Infinity Thermostated Column Compartment, a 1260 Infinity Diode Array Detector and a 1260 Infinity Fluorescence Detector. A Kinetex-XB C18, 2.6 μm, 100 × 3 mm (Phenomenex) served as stationary phase at a flow rate of 0.5 mL/min or 0.6 mL/min. The oven temperature was set to 25 °C. UV detection was performed at 220 nm and fluorescence detection at 275/305 nm. The injection volume was 20 μL. Mixtures of 0.04% aq TFA (A3), 0.05% aq HCOOH (A4) or 0.1% aq HCOOH (A5) and B1 or MeOH (B2) were used as mobile phase. The following linear gradients were applied for purity controls: compounds **8**, **9**, **11**, **12**, **14–18**, **25**, **26**, **28**, **29**, **31–35** and **38–57** (flow rate 0.6 mL/min): 0–12 min: A3/B1 90:10–70:30, 12–16 min: 70:30–5:95, 16–20 min: 5:95; compounds 19–23, 36, 37 (flow rate 0.5 mL/min): 0–12 min: A4/B2 95:5–70:30, 12–16 min: 70:30–5:95, 16–20 min: 5:95. The following linear gradient was used for the analysis of plasma stability samples: 0–12 min: A3/B1 90:10–73:27, 12–16 min: 73:27–5:95, 16–20 min: 5:95. Retention (capacity) factors *k* were calculated from the retention times *t*_R_ according to *k* = (*t*_R_ − *t*_0_)/*t*_0_ (*t*_0_ = dead time). Peptides were characterized by ^1^H- and ^1^H-COSY NMR spectroscopy, HRMS, and RP-HPLC analysis. Additionally, ^13^C-NMR spectra were acquired of **50** and **51**. 

Annotation concerning the ^1^H-NMR spectra (solvent: DMSO-*d*_6_): in order to allow an integration of the signals interfering with the broad water signal at ca. 3.5 ppm, spectra were additionally recorded in DMSO-*d*_6_/D_2_O (4:1 *v*/*v* (**8**, **9**, **11**, **14–17**, **19–22**) or 5:1 *v*/*v* (**12**, **18**, **23**, **25**, **26**, **28**, **29**, **32–57**)).

### 2.2. Cell Culture and Preparation of HEK293T Cells Stably Expressing the Human NTS_2_R

All cells were cultured in 75 or 175 cm^2^ flasks (Sarstedt, Nuümbrecht, Germany) in a humidified atmosphere (95% air, 5% CO_2_) at 37 °C. HT-29 colon carcinoma cells (DSMZ-no. ACC 299) were maintained in antibiotic-free RPMI medium (Sigma-Aldrich) supplemented with 7.5% fetal bovine serum (FBS) (Sigma-Aldrich). HEK293T cells stably expressing the human NTS_2_R (HEK293T-hNTS_2_R cells) were essentially generated following a previously described procedure [61]. In brief, HEK293T cells (kind gift from Prof. Dr. Wulf Schneider, Institute for Medical Microbiology and Hygiene, University of Regensburg, Germany) were seeded on a 6-well plate (Sarstedt, Nümbrecht, Germany) in Dulbecco’s modified Eagle’s medium (Sigma-Aldrich) supplemented with 10% FBS, l-glutamine (2 mM) (Sigma-Aldrich) and Penicillin-Streptomycin (100 IU/mL and 0.1 mg/mL, respectively) (Sigma-Aldrich) at a density of 6 × 10^5^ cells/well. On the next day, cells were transfected with 2 µg of cDNA encoding the hNTS_2_R (cDNA Resource Center, Rolla, MO, USA, catalog no. NTSR200000) using X-tremeGENE^TM^ HP (Roche Diagnostics, Mannheim, Germany) as transfection reagent according to the manufacturer’s protocol. After two days of transfection, cells were detached with trypsin-ethylenediamine-tetraacetic acid (EDTA, Biochrom, Berlin, Germany) and transferred to a 15-cm dish (Sarstedt, Nümbrecht, Germany). After the cells had attached to the dish, G418 (Biochrom, Berlin, Germany) was added at a final concentration of 1 mg/mL. Selection was achieved by exchanging the medium every two to three days for two weeks. Subsequently, a clone with high NTS_2_R-expression, which was assessed radiochemically after addition of 10 nM of [^3^H]UR-MK300, was isolated. Cultivation was then continued with a reduced G418 concentration in the culture medium of 600 µg/mL. 

### 2.3. Radiochemical Binding Assays

#### 2.3.1. NTS_1_R Binding

Radioligand competition binding experiments with [^3^H]UR-MK300 (specific activity: 47.0 Ci/mmol [39] or 65.0 Ci/mmol; for structure see Figure S1, Supplementary Materials) at hNTS_1_R-expressing intact human HT-29 colon carcinoma cells were performed at 23 ± 1 °C as described previously [39]. Two different batches of the radioligand [^3^H]UR-MK300 were used. The *K*_d_ values of [^3^H]UR-MK300 amounted to 0.55 nM (mean value from two independent saturation binding experiments, each performed in triplicate) [40] and 0.41 ± 0.12 nM (mean value ± SD from two independent saturation binding experiments, each performed in triplicate). Specific binding data (obtained by subtracting unspecific binding from total binding) were normalized (100% = specifically bound radioligand in the absence of competitor) and plotted over log(concentration of competitor) followed by a four-parameter logistic fit (SigmaPlot 12.5, Systat Software, San José, CA, USA) (note: in the case of **40**, the lower curve plateau of the sigmoidal fit was constrained to >0). Resulting pIC_50_ values were converted to IC_50_ values and *K*_i_ values were calculated from the IC_50_ values according to the Cheng-Prusoff equation [62] using a *K*_d_ value of 0.55 nM (**8**, **9**, **11**, **14–17** and **19–22**) or 0.41 nM (**12**, **18**, **23**, **25**, **26**, **32–50**, **52** and **54–57**). The *K*_i_ values from individual experiments were transformed to p*K*_i_ values, followed by the calculation of mean p*K*_i_ values ± SD. 

#### 2.3.2. NTS_2_R Binding

NTS_2_R saturation and competition binding experiments were performed at intact HEK293T-hNTS_2_R cells at 23 ± 1 °C using [^3^H]UR-MK300 [39] as radioligand (two different batches were used; specific activities: 47.0 Ci/mmol [39] and 65.0 Ci/mmol). Two days prior to the experiment, white 96-well plates with clear bottoms (Costar, catalog no. 3610) were treated with poly-d-lysine hydrobromide (Sigma-Aldrich) for 10 min. The wells were washed with H_2_O and the plates were dried on air at rt overnight. Alternatively, plates were treated with a sterile solution of 5% (*w*/*v*) gelatin (Sigma-Aldrich) in H_2_O (50 µL) at rt for 1.5–2 h. The gelatin solution was removed, followed by the addition of a solution of 2.5% (*v*/*v*) of glutaraldehyde (Sigma-Aldrich) in H_2_O (50 µL) at rt for 10 min. After removal of the glutaraldehyde solution, the wells were washed twelve times with H_2_O and two times with culture medium (150–300 µL). One day before the experiment, cells were seeded in the treated plates at a density of 9 × 10^4^ cells/well. On the day of the experiment, the culture medium was carefully removed using a multi-channel pipette (Transferpette S-12, Brand, Wertheim, Germany) and the cells were washed once with Dulbecco’s phosphate-buffered saline (D-PBS) containing Ca^2+^ and Mg^2+^ (1.8 mM CaCl_2_, 2.68 mM KCl, 1.47 mM KH_2_PO_4_, 3.98 mM MgSO_4_, 136.9 mM NaCl and 8.06 mM Na_2_HPO_4_) (200 μL, rt) followed by the careful pre-filling of the wells with 180 µL (total binding) or 160 µL (unspecific and competition binding) of D-PBS, supplemented with 1% BSA and 100 μg/mL bacitracin (Serva, Heidelberg, Germany) (in the following referred to as binding buffer). To determine total binding, 20 µL of a solution of the radioligand in binding buffer (10-fold concentrated compared to the final concentration) were added. For the determination of unspecific binding, 20 µL of a solution of **1** in binding buffer (10-fold concentrated, used in 500-fold excess compared to the radioligand) and 20 µL of a 10-fold concentrated solution of the radioligand in binding buffer were added. To determine the displacing effect of a compound of interest, 20 µL of a solution of the respective compound in binding buffer (10-fold concentrated) and 20 µL of a 10-fold concentrated solution of the radioligand in binding buffer were added. During the incubation period of 2 h at 23 °C, the plates were gently shaken. After incubation, the liquid was carefully removed using a multi-channel pipette and the cells were carefully washed twice with ice-cold D-PBS (200 μL). 25 μL of lysis solution (8 M urea, 3 M acetic acid, and 1% Triton-X-100 in H_2_O) were added to each well and the plates were shaken at rt for 25 min, followed by the addition of liquid scintillator (Ultima Gold, PerkinElmer, Waltham, MA, USA) (200 μL). The plates were sealed with a transparent sealing tape (permanent seal for microplates, PerkinElmer, product no. 1450–461) and turned upside down several times to achieve complete mixing. Prior to the measurement of the radioactivity with a MicroBeta2 plate counter (PerkinElmer), the plates were kept in the dark for at least 1 h. All experiments were performed in triplicate. The *K*_d_ values of [^3^H]UR-MK300, determined for the different batches of radioligand by saturation binding experiments, amounted to 6.9 ± 1.8 nM (mean value ± SD from six independent determinations, each performed in triplicate) and 4.0 ± 1.5 nM (mean value ± SD from three independent determinations, each performed in triplicate) (for representative saturation binding curves see Figure S2, Supplementary Materials). Data from competition binding experiments were analyzed as described for NTS_1_R binding using a *K*_d_ value of 6.9 nM (**1**, **8**, **9**, **14**, **15**, and **19**) or 4.0 nM (**3**, **9–11**, **15–17**, **19–22**, **48–53**, and **56**). Note: in the cases of **49–53** and **56**, the lower curve plateau of the sigmoidal fit was constrained to >0. 

### 2.4. Fura-2 Ca^2+^-Assay

The fura-2 calcium assay on intact hNTS_1_R-expressing HT-29 cells was performed as previously described for human erythroleukemia cells [63] using a Perkin-Elmer LS50 B spectrofluorimeter (PerkinElmer, Rodgau, Germany). At a confluency of 80–95%, cells were trypsinized, detached from the culture flask and the assay was performed as described in the protocol. Net Ca^2+^ responses (basal cytosolic Ca^2+^ concentration subtracted from the measured Ca^2+^ concentration), induced by **1**, **21,** and **56**, were normalized (100% = effect elicited by 300 nM NT(8-13)) and plotted over log(concentration of agonist) followed by a four-parameter logistic fit (SigmaPlot 12.5, Systat Software). 

### 2.5. Investigation of the Stability of **8**, **9**, **11**, **12**, **14–23**, **38–49** and **54–57** in Human Plasma

The proteolytic stabilities of **8**, **9**, **11**, **12**, **14–23**, **38–49,** and **54–57** were investigated in human blood plasma/PBS (136.9 mM NaCl, 2.68 mM KCl, 5.62 mM Na_2_HPO_4_, 1.09 mM NaH_2_PO_4_ and 1.47 mM KH_2_PO_4_) pH 7.4 (1:2, *v*/*v*) according to a described procedure [40] with the following modifications: 5 mM stock solutions in MeCN/0.04% aq TFA (30:70 *v*/*v*) were used throughout for the addition of the peptides to plasma/PBS (1:2 *v*/*v*). As the RP-HPLC purity of 1-methyl-d-Trp, used as internal standard (IS) was <95%, the compound was purified by preparative HPLC to give a purity of >99%. The concentration of the peptides in plasma/PBS (1:2 *v*/*v*) was 80 and 4 µM (recovery determination) or 100 µM (stability tests). Data analysis was based on UV detection at 220 nm (**8**, **9**, **11**, **12**, **14–18**, **21–23**, **38–49**, and **54–57**) or fluorescence detection at 275/305 nm (**19** and **20**). Reference samples, representing 100% recovery, were prepared in duplicate (**8**, **9**, **11**, **14–16**, and **19–21**) or quadruplicate (**12**, **17**, **18**, **22**, **23**, **38–49**, and **54–57**). Recovery ratios were obtained by dividing the recovery of the peptide by the recovery of IS for each individual sample (n = 3–5). The obtained recoveries and the recovery ratios are summarized in Table S2, Supplementary Materials. Note: in the case of compounds **38–49**, which were prepared for testing the effects of various unnatural amino acids on peptide stability, no recovery ratios were determined. Instead, the recovery ratios determined for the previously reported, structurally closely related peptide Me-Arg-Arg-Pro-Tyr-Ile-Leu [40] were used for calculating the amount of remaining intact peptide in plasma.

### 2.6. Circular Dichroism (CD) Analysis

CD spectra of 100 µM aqueous solutions of **48** and **49** and a reference compound with the amino acid sequence Me-Arg-Arg-Pro-Tyr-Ile-Leu [40] were recorded in a 1 cm path length cuvette at 20 °C with a Jasco J-810 spectropolarimeter (Jasco, Tokyo, Japan) equipped with a PTC-423S Peltier temperature controller (Jasco). Instrumental parameters: spectral range, 180−300 nm; bandwidth, 1 nm; scanning speed, 500 nm/min. Each spectrum represents the average of three spectra, each recorded with 20 accumulations, after solvent subtraction. An “economy-size” singular-value decomposition (SVD) on a set of nine spectra (matrix A consisting of three spectra for each of **48**, **49** and the reference compound) was calculated in MATLAB (MathWorks, Natick, MA) as A = U·S·V^T^. Here, U is a matrix whose columns contain the linearly independent spectral components, S is a diagonal matrix containing the singular values, and V^T^ is the transpose of matrix V, which contains the linear coefficients associated with the spectral components in U.

### 2.7. Synthesis, In Vitro and In Vivo Characterization of PET Tracers [^68^Ga]21, [^68^Ga]33, [^68^Ga]37 and [^68^Ga]56

#### 2.7.1. PET Tracer Synthesis

The preparation of the ^68^Ga-labeled PET ligands [^68^Ga]**21**, [^68^Ga]**33**, [^68^Ga]**37** and [^68^Ga]**56** was performed on a Scintomics GRP^®^ synthesizer module (Scintomics GmbH, Fürstenfeldbruck, Germany) with the Scintomics Control Center software, the Reagent and Hardware Kit SC-01 and SC-01-H (ABX, Radeberg, Germany) and a Isomed 2010 activimeter (MED Nuklear-Medizintechnik, Dresden, Germany) for activity measurements. [^68^Ga]GaCl_3_ was eluted from a ^68^Ge/^68^Ga-generator GalliaPharm (Eckert&Ziegler, Berlin, Germany) with 0.1 M HCl (Eckert&Ziegler) (approximately 9 mL). A 0.12–0.15 mM solution of the precursor compound (**16**, **32**, **35** or **54**) in ultrapure H_2_O (Merck) (100 µL) was added to a HEPES buffer (ABX Kit; 1.5 M, pH 5.5) (3 mL), combined with the gallium eluate and the mixture was incubated for 6 min ([^68^Ga]**21**, [^68^Ga]**33**, [^68^Ga]**56**) or 16 min ([^68^Ga]**37**) at 125 °C, cooled down to approximately 120 °C and loaded on a C18 cartridge (Sep-Pak C18 Plus Short Cartridge, 55–105 µm, Waters, Milford, MA, USA). After a washing step with H_2_O (ca. 8 mL), the product was eluted from the cartridge with EtOH (effective volume ca. 1 mL) and the eluate was transferred into a 2-mL reaction vessel. The solvent was evaporated in a Savant Speed-Vac SVC100H vacuum concentrator (Savant Instruments, Farmingdale, NY, USA) equipped with pre-heated (100 °C) rotor inserts (aluminum blocks with bores for 1.5- and 2-mL reaction vessels) for approximately 50 min (note: a complete evaporation to dryness was avoided; the residual volume was approximately 10–30 µL), followed by uptake in 0.1% aq HCOOH (80–100 µL). The solution was subjected to preparative work-up using an HPLC system composed of a P4000 pump (Thermo Separation Products), a Degassex DG-4400 degasser (Phenomenex), a 2487 UV/visible detector (Waters) and a Rheodyne manual injector equipped with a 200 µL loop (note: the pump was directly controlled via the front panel and the UV/Vis-detectorwas remote-controlled using Waters Millennium Software). For the detection of γ-radiation, a B20/G-10 RADEye (Thermo Scientific, Erlangen, Germany) was placed close to the outlet tubing of the UV/Vis-detector (note: the vessel used for waste collection was shielded from the RADEye by 2 cm of lead). The stationary phase, a Luna C18(2), 3 μm, 150 × 4.6 mm (Phenomenex), was placed in a box of lead (wall thickness: 2 cm). The flow rate was 0.5 mL/min. Mixtures of A5 and B2 were used as mobile phase. The following linear gradients were applied: [^68^Ga]**21**: 0–16 min: A5/B2 85:15–65:35, 16–17 min: 65:35–5:95, 17–22 min: 5:95; [^68^Ga]**33** and [^68^Ga]**37**: 0–16 min: A5/B2 65:35–45:55, 16–17 min: 45:55–5:95, 17–22 min: 5:95; [^68^Ga]**56**: 0–16 min: A5/B2 80:20–60:40, 16–17 min: 60:40–5:95, 17–22 min: 5:95. UV detection was performed at 220 nm and 275 nm (note: the chosen conditions enabled a separation of excessive labeling precursor from the Ga^3+^-labeled species. For an exemplary chromatogram of a co-injection of **32** and **33** (50 µM each, injection volume 75 µL), and a chromatogram of the separation of [^68^Ga]**33** from remaining **32** after radiosynthesis see Figure S7, Supplementary Materials). The eluate, containing the PET ligand, was collected in a 2-mL reaction vessel immediately followed by removal of the solvent in a vacuum concentrator equipped with pre-heated (100 °C) aluminum blocks (35–40 min). However, a complete evaporation of the solvent was avoided. The aqueous residue (20–40 µL) was taken up in PBS (ABX Kit) (80–500 µL) yielding a solution referred to as “tracer stock” in the following (final activity 0.157–1.69 GBq/mL). Quality controls of the PET ligands were performed by HPLC analysis using a system from Agilent Technologies (Waldbronn, Germany) consisting of a 1100 Series quaternary pump equipped with a 1260 Infinity degasser, a 1100 Series Autosampler, a 1100 Series Thermostated Column Compartment, a 1100 Series Diode Array Detector, and a GABI Star radiometric detector (Raytest Isotopenmessgeräte GmbH, Straubenhardt, Germany). A Luna C18(2), 3 μm, 100 × 4.6 mm (Phenomenex) served as stationary phase. The flow rate was 0.95 mL/min and the temperature of the Column Compartment was set to 25 °C. The following linear gradients were applied: [^68^Ga]**21**: 0–9 min: A3/B1 95:5–72:28, 9–12 min: 72:28–5:95, 12–16 min: 5:95; [^68^Ga]**33** and [^68^Ga]**37**: 0–9 min: A3/B1 95:5–55:45, 9–12 min: 55:45–5:95, 12–16 min: 5:95; [^68^Ga]**56**: 0–9 min: A3/B1 95:5–65:35, 9–12 min: 65:35–5:95, 12–16 min: 5:95. UV detection was performed at 220 nm. Dilutions of the tracer stocks (5–25 µL, 0.17–1.39 MBq) were injected into the HPLC system. PET tracer-specific details about the syntheses including radiochemical yields and purities are provided in Table 1.

#### 2.7.2. Determination of the Distribution Coefficient logD_7.4_ of PET Ligands [^68^Ga]21, [^68^Ga]33, [^68^Ga]37 and [^68^Ga]56

The distribution coefficients logD_7.4_ of the radiotracers [^68^Ga]**21**, [^68^Ga]**33**, [^68^Ga]**37** and [^68^Ga]**56** were determined by adding a solution of the tracer in PBS (100 μL, ca. 0.20–0.34 MBq) to a mixture of n-octanol (500 μL) and PBS (pH 7.4) (400 μL) in a 2-mL HPLC vial (Agilent, article number 5182-0714) with a screw cap equipped with a septum (Agilent, article number 5182-0717). After vortexing the mixture for 2 min, two 100 μL aliquots of the upper phase (n-octanol) were taken. To obtain a sample of the lower phase (PBS), the HPLC vial was held upside down and approximately 100–150 µL of the aqueous phase were removed using a syringe equipped with a canula and collected in a reaction vessel. Two 10 µL aliquots of the aqueous phase were subjected to measurement. The activity of the aliquots was measured with a Canberra Genie 2000 system (Canberra, Rüsselsheim, Germany) using the Gamma Acquisition & Analysis Software Genie 2000 3.4.1. Experiments were performed in triplicate. The decay-corrected counts per minute (cpm) values were averaged (n = 3) and transformed to a distribution coefficient logD_7.4_ according to logD_7.4_ = log(A_octanol_/A_aqueous_), where A_octanol_ is the mean of the decay-corrected cpm values obtained for samples of the n-octanol phase, and A_aqueous_ is the mean of the decay-corrected cpm values obtained for samples of the aqueous phase.

#### 2.7.3. Mouse Xenograft Model

8–12 weeks old female NMRI nude (nu/nu) mice (body weight 22–32 g) (Charles River, Sulzfeld, Germany) were kept under specified pathogen free (SPF) conditions at 23 °C, 55% relative humidity and a 12 h light/dark cycle in the central animal facility of the University of Regensburg using type III cages from Tecniplast (Hohenpeißenberg, Germany). The animals took food (Ssniff, Soest, Germay) and autoclaved tap water ad libitum. For tumor cell implantation, the culture medium of HT-29 cells was removed, the cells were detached from the culture flask by incubation for 2 min in trypsin-EDTA and the suspension was centrifuged (5 min, rt, 164 g). The supernatant was removed, and the cell pellet was washed twice with sterile PBS or serum-free medium (5–6 mL). The cells were resuspended in sterile PBS to a final density of 1 × 10^7^ cells/mL. Mice were injected subcutaneously into the right flank with the HT-29 cell suspension (100 µL). After 2–4 weeks, when the tumors had reached a size of 50–500 mm^3^, animals were used for biodistribution and PET/computed tomography (CT) imaging studies (note: for PET/CT studies, the tumor size was at least 200 mm^3^). 

#### 2.7.4. Animal Anesthetization

Mice were anesthetized by i.p. injection (100 µL per 10 g body weight) using a mixture that was prepared by addition of ketamin (Medistar Arzneimittelvertrieb, Ascheberg, Germany, 10 wt%, 800 µL) and xylazine (Serumwerk, Bernburg (Saale), Germany, 2 wt%, 200 µL) to PBS (9 mL). 

#### 2.7.5. Biodistribution Studies

Aliquots of the tracer stock were diluted in PBS to give a volume of 200 µL. 80–100 µL (0.9–5.9 MBq) of this solution were injected into anesthetized HT-29 tumor-bearing nude mice via the tail vein. Animals were kept on a heating plate (set to 40 °C) or a pre-heated (ca. 45 °C) gel cushion and were killed by cardiac puncture 10 min, 25 min or 45 min p.i. immediately followed by taking blood, urine and tissue (i.e., tumor, kidney, liver, gall bladder, spleen, small intestine, heart, lung, brain, pancreas, femur and muscle) samples. Radioactivity measurement of the samples was performed with the Canberra Genie 2000 system described for the determination of the distribution coefficient logD_7.4_. Decay-corrected measured activities (cpm) were converted into activities (MBq) on the basis of an activity measurement with a ^68^Ge-calibration standard source (Eckert&Ziegler, Berlin, Germany). Sample activities were converted to percentage of injected dose per gram tissue (%ID/g). Blocking experiments were performed by co-injection of the tracer with NTS_1_R ligand **48** (ca. 700 nmol per mouse). Mice were killed 45 min p.i. and analyzed as described above. The animals used for the 45-min biodistribution experiments were the same as used for the PET/CT imaging studies.

#### 2.7.6. HPLC Analysis of Urine from Mice Injected with [^68^Ga]**21**, [^68^Ga]**33**, [^68^Ga]**37** or [^68^Ga]**56**

The analysis was performed with the urine obtained from biodistribution and PET imaging studies, respectively (t = 10 or 45 min). The urine was diluted with H_2_O (1:2–1:100 depending on the activity in the urine) and 10–80 µL of this solution were subjected to analysis by analytical HPLC using the same HPLC system and conditions as described for the quality controls of the PET ligands. To confirm the identity of [^68^Ga]**56** in the 45-min urine samples, an additional analysis was performed, using the aforementioned injection solution spiked with 100 µM **56**. Representative chromatograms of the HPLC analysis of the urine samples are shown in Figures S9–S11, Supplementary Materials.

#### 2.7.7. HPLC Analysis of Blood Plasma from Mice Injected with [^68^Ga]**56**

This analysis was performed with the blood obtained from biodistribution studies with [^68^Ga]**56** (t = 10 min). Blood (ca. 200 µL) was taken from the heart using a syringe that was rinsed with sodium heparin (25000 I.E., Ratiopharm, Ulm, Germany). The heparinized blood was transferred into a 1.5-mL reaction vessel immediately followed by centrifugation (5 min, 4 °C, 1200 g) using a Biofuge fresco centrifuge (Heraeus, Hanau, Germany). The supernatant (ca. 100 µL) was treated with the same volume of 10% aq TFA (precipitation of proteins) and the mixture was centrifuged (10 min, 4 °C, 16,100 *g*). The supernatant (70–100 µL) was subjected to analysis by analytical HPLC using the same HPLC system and conditions as described for the quality control of the PET ligand. To confirm the identity of [^68^Ga]**56** in the plasma samples, an additional analysis was performed, using the aforementioned injection solution spiked with 100 µM **56**. Representative chromatograms of the HPLC analysis of the plasma samples are shown in Figure S11C–E, Supplementary Materials.

#### 2.7.8. Determination of the Internalization of [^68^Ga]**56** in HT-29 Tumor Cells

HT-29 cells were seeded in 24-well TC plates (Sarstedt, catalog no. 83.3922) one day prior to the experiment at a density of 4.5 × 10^5^ cells/well. Shortly before the experiment, the culture medium was removed and the cells were washed once with PBS (500 μL, rt) followed by pre-filling of the wells with 250 µL of binding buffer (see procedure for NTS_2_R binding studies). For the determination of unspecific binding, the binding buffer was supplemented with **1** (2 µM). 50 µL aliquots of the [^68^Ga]**56** stock in PBS (1.5–2.8 MBq) were added to the wells, and the cells were incubated at 37 °C for 5, 10, 15, 25, 35, 55, or 75 min under gentle shaking. After completed incubation, the plates were immediately placed on ice, the liquid was removed by suction and the cells were washed twice with ice-cold PBS (500 μL). The cells were washed twice with ice-cold acid strip buffer (50 mM glycine and 125 mM NaCl in H_2_O, pH 3.0) (300 µL) for 5 min each, and the washings were combined in 5-mL polypropylene tubes. The cells were then lysed by the addition of lysis solution (see procedure for NTS_2_R binding studies) (250 µL) and shaken at 37 °C for 15 min. The lysates were transferred into 5-mL polypropylene tubes, the wells were washed with lysis solution (250 µL) and the washings were combined with the lysates. The activities were measured with the Canberra Genie 2000 system as described for the determination of the distribution coefficient logD_7.4_. The decay-corrected cpm values of unspecific binding were subtracted from the decay-corrected cpm values of total binding to obtain specific binding data for both surface-bound [^68^Ga]**56** (acid strip) and internalized [^68^Ga]**56** (cell lysate). The amount of internalized specific binding was normalized based on total specific binding, and the mean values from two independent experiments (each performed in triplicate) were plotted against the incubation time.

#### 2.7.9. PET/CT Imaging with [^68^Ga]**56**

PET/computed tomography (CT) imaging was performed using an EARL-certified clinical PET/CT scanner (Biograph mCT-S(40) with TrueV and Flow-4R technology, Siemens Healthcare, Erlangen, Germany) exhibiting, at 1 cm off-center, a spatial resolution for ^18^F of 4.1 mm in transversal and 4.7 mm in axial direction according to the NEMA NU2-2007 standard [64]. This PET/CT scanner is capable of obtaining PET images in small animal experiments with reported recovery coefficients from NEMA NU 4 phantom measurements of 0.21, 0.59, and 1.16 in rods with a diameter of 2, 3, and 4 mm, respectively [65]. After positioning the animal, topograms were acquired (70 kVp, 60 mA, slice 0.6 mm, manually terminated craniocaudal movement) with tube position at bottom and lateral for anteroposterior and lateral view, respectively. A CT scan (70 kVp, 140 mA) was performed without dose reduction (CARE Dose 4D and CARE kV off) with the minimal slice scanning thickness of 0.6 mm (40 × 0.6 mm) with a pitch of 0.35 and a rotation time of 0.5 s resulting in a typical acquisition time of about 27 s. For attenuation correction of the PET data acquisition, axial CT images were reconstructed with the full field of view (FoV) of 780 mm by the FAST reconstruction algorithm using the “B30f medium smooth” kernel with an increment of 0.6 mm, typically resulting in 369 images. For visual analysis, axial CT images were reconstructed with a reduced FoV of 100 mm by the SAFIRE reconstruction algorithm (strength = 3) by the “I49f medium” kernel with an increment of 0.3 mm, typically resulting in 737 images. For PET data acquisition, the animal bed was positioned in the center of the PET detector. Simultaneously with the injection of the tracer, a dynamic PET scan (list mode for 45 min in a single bed position) was started.

#### 2.7.10. Tracer Administration

Aliquots of the tracer stock of [^68^Ga]**56** were diluted in PBS to give a volume of 200 µL. 80–100 µL of this solution (3.2–5.9 MBq per mouse) were injected into the tail vein of anesthetized HT-29 tumor-bearing nude mice placed in the scanner. During the PET scan, mice were kept on a pre-warmed (approximately 45 °C) gel pad. Immediately after the PET scan, mice were killed followed by taking blood, urine, tumor tissue and organ tissues. Blocking experiments were carried out by co-injection of the tracer with **48** (ca. 700 nmol per mouse).

#### 2.7.11. Imaging Analysis

For dynamic analysis of the PET images, list mode data were replayed according to the following frame scheme: 6 × 10 s, 4 × 30 s, 1 × 2 min, 4 × 5 min, 2 × 10 min. All PET scans were corrected for normalization, detector dead time, attenuation, scatter, decay, random coincidences and prompt gamma coincidences. Attenuation corrected PET images (512 × 512 pixels, pixel size 0.40 mm, slice thickness 2.03 mm) were reconstructed by iterative reconstruction (8 iterations, 24 subsets, point spread function modelling) with a Gaussian post reconstruction filter with 1.0 mm full width at half maximum (FWHM). Additionally, static frames from 5 min to 10 min and for the entire acquisition of 45 min were reconstructed (512 × 512 pixels, pixel size 0.40 mm, slice thickness 0.6 mm) from the list mode data. By means of static recording over the complete acquisition period, possible movements of the mouse could be excluded. For determination of the tracer uptake in tumors and kidneys corresponding regions of interest (ROIs) were generated using a fixed threshold of 45% of the maximum tracer accumulation for tumors and 40% of the maximum tracer accumulation for kidneys. The resulting delineations were inspected visually and corrected manually if necessary. Manual correction was performed for all tumors in mice used for blocking experiments which exhibited only low diffuse tracer accumulation. For determination of the tracer uptake in the muscles, a ROI was delineated manually in the left femoral muscles observing a volume of approximately 0.05 mL. The ROIs were transferred to all time frames of the respective dynamic study. Time-activity-curves (TACs) were generated by computing the mean standardized uptake value normalized to body weight (SUV_mean_) in each frame. ROI definition and ROI analyses were performed using the ROVER software, version 3.0.64 (ABX GmbH, Radeberg, Germany).

## 3. Results and Discussion

### 3.1. Chemistry

**Scheme 1:**
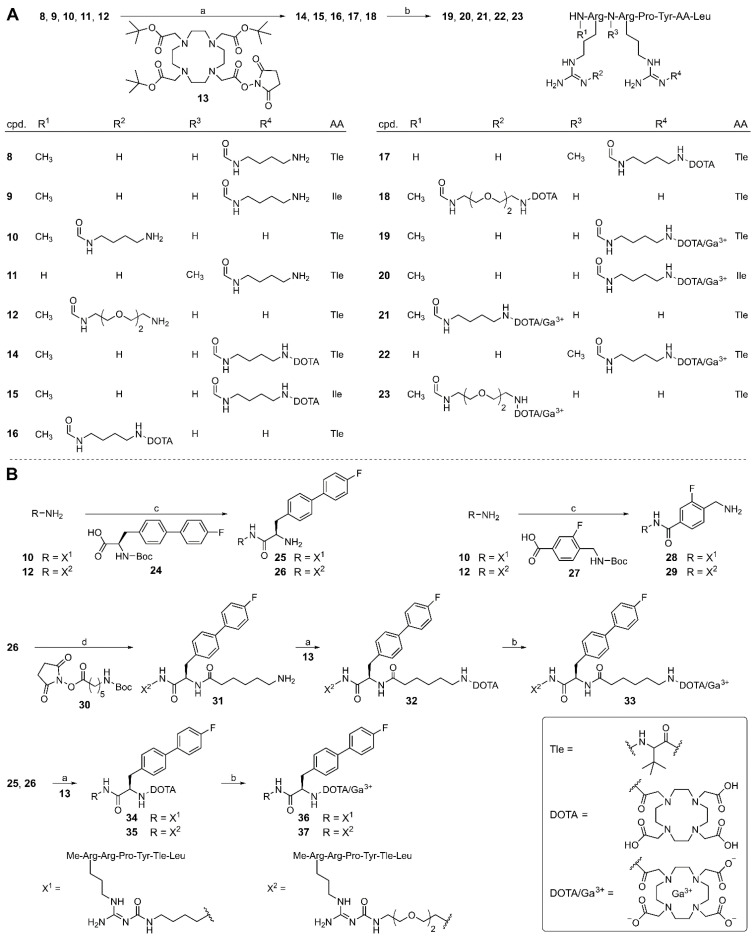
(**A**) Synthesis of the potential NTS_1_R PET ligands **19–23** from the amino-functionalized precursor peptides **8–12**. (**B**) Synthesis of amino-functionalized NT(8-13) derivatives containing a fluorinated biphenyl or benzoyl moiety (**25**, **26**, **28**, **29**, and **31**) and preparation of the potential NTS_1_R PET ligands **33**, **36** and **37**. Reagents and conditions: (a) (1) DIPEA, solvent: DMF/NMP 75:25 or 80:20 *v*/*v*, rt, 30 min, (2) TFA/H_2_O 80:20 *v*/*v*, 50 °C, overnight, 72% (**14**), 62% (**15**), 75% (**16**), 28% (**17**), 93% (**18**), 69% (**32**), 78% (**34**), 81% (**35**); (b) preheating of a solution of the peptide (4 mM) in HEPES buffer (0.2 M, pH 4.2) to 60 °C, 5 min, addition of Ga(NO_3_)_3_ × H_2_O in 10 mM HCl, 100 °C, 10–30 min, 95% (**19**), >99% (**20**), 92% (**21**), 99% (**22**), 92% (**23**), 99% (**33**), 96% (**36**), 95% (**37**); (c) (1) DIPEA, HOBt, HBTU, solvent: DMF/NMP 80:20 *v*/*v*, rt, 60–75 min, (2) TFA/H_2_O 95:5 *v*/*v*, rt, 3.5 h, 46% (**25**), 47% (**26**), 24% (**28**), 32% (**29**); (d) (1) DIPEA, solvent: DMF/NMP 75:25 *v*/*v*, rt, 45 min, (2) TFA/H_2_O 95:5 *v*/*v*, rt, 3.5 h, 66% (**31**).

Standard Fmoc strategy solid-phase peptide synthesis (SPPS) was used for the preparation of the NT(8-13)-derived peptides **8**, **9**, **10** [60], **11,** and **12**, containing an amino-functionalized arginine (position 8 or 9) derived from the reported Fmoc- and Boc-protected *N*^ω^-carbamoylated arginine building blocks **6** or **7** [39] (structures shown in Figure 1B) (Scheme 1). Peptides **8–12** were *N*^α^-methylated in position 8 (**8–10** and **12**) or 9 (**11**), and *α*-*tert*-butyl-Gly (Tle) was incorporated in position 12 in peptides **8** and **10–12** instead of Ile^12^. Details on the coupling conditions are provided in Table S1, Supplementary Materials.

A reported procedure for the on-resin *N*^α^-methylation of peptides [66], which was recently used for *N*^α^-methylation of the carbamoylated arginine in **10** [60], was also successfully applied for *N*^α^-methylation of the carbamoylated arginines in **11** and **12**. Coupling of Fmoc amino acids to an *N*^α^-methylated N-terminal amino acid using the standard coupling reagents HOBt, HBTU and DIPEA proved to be unfeasible; therefore, **11** was prepared by applying a combination of oxyma and DIC as activation reagents. Detailed information on the synthesis procedures and the applied coupling conditions are given in the Supplementary Materials. 

Compounds **8–12** served as starting materials for the syntheses of the chelator-conjugated peptides **14–18** using the tris-*t*Bu-protected DOTA reagent **13** for coupling to the amino-functionality of the *N*^ω^-carbamoylated arginine in **8–12**. Attempts to introduce the DOTA moiety using a non-protected DOTA succinimidyl ester caused severe separation problems due to nearly identical HPLC retention times of precursor peptide and product. However, the *t*Bu-protected intermediates could easily be separated from the remaining starting material, followed by deprotection with acid overnight and purification, yielding **14–18** with HPLC purities of >99%. 

The “cold” PET ligands **19–23** were prepared by incubation of **14–18** with natural ^69^Ga^3+^ in a HEPES buffer pH 4.2 at 100 °C (Scheme 1). Complete conversion of the starting material was achieved after only 10 min. Under these conditions, the peptides proved to be stable. It should be noted that the potential PET ligands **19–23** could not be separated from the remaining respective precursor peptide (C18 RP-HPLC) when using acetonitrile and 0.04% aqueous TFA as eluent. However, baseline separation was achieved using MeOH and 0.05% formic acid as mobile phase.

For the purpose of the preparation of less polar PET ligands, peptides **10** and **12**, containing a tetramethylene and a dioxaoctamethylene linker, respectively, in the amino-functionalized *N*^ω^-carbamoylated arginine, were conjugated to the fluorinated biphenyl-Ala spacer **24**, yielding **25** and **26** (after subsequent Boc-deprotection), or to the fluorinated aminomethyl-benzoyl spacer **27**, affording **28** and **29** (after subsequent Boc-deprotection), using HOBt, HBTU and DIPEA as coupling reagents. The side chain of the carbamoylated arginine in **26** was further elongated by treatment with succinimidyl ester **30**, yielding a terminal aminohexanoyl moiety in the arginine side chain after subsequent Boc-deprotection (**31**). 

Compounds **31**, **25,** and **26** were treated with **13** as described above, giving the DOTA-conjugated compounds **32**, **34** and **35** after removal of protecting groups (note: **28** and **29** were not further processed by coupling to DOTA as they proved to be more polar (shorter RP-HPLC retention times) than **25**, **31** and **26**). Finally, **32**, **34,** and **35** were converted into the potential PET ligands **33**, **36,** and **37** by insertion of Ga^3+^. Unlike the synthesis of **19–23**, complete conversion of the starting material was only achieved after incubation at 100 °C for 30 min (as verified by analytical HPLC).

**Figure 2:**
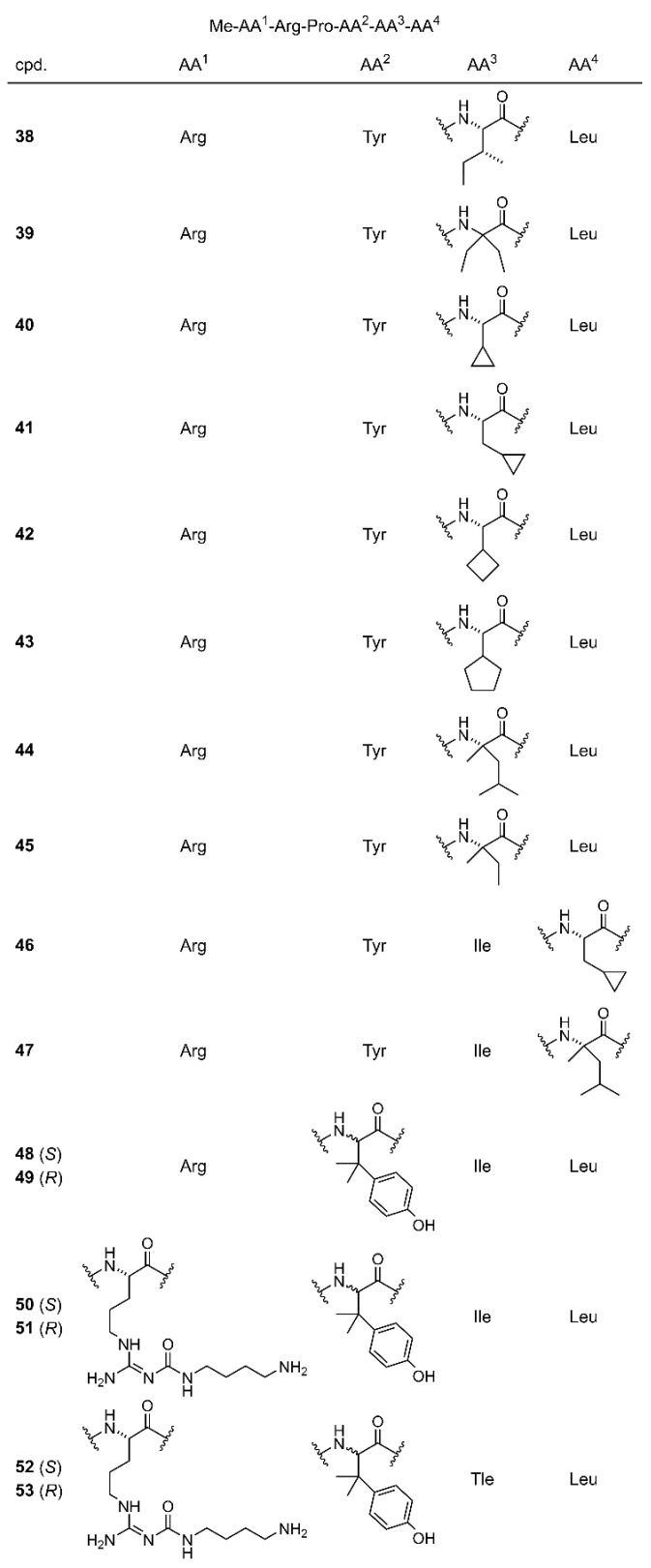
Structures of the C-terminally modified NT(8-13) derivatives **38–49** and the amino-functionalized precursor compounds **50–53**, representing derivatives of **48** and **49**.

Aiming at a PET ligand with high in vivo stability, a series of N-terminally methylated NT(8-13) derivatives was synthesized by SPPS containing various commercially available unnatural amino acids in position 12 (**38–45**), 13 (**46** and **47**), or 11 (**48** and **49**) (Figure 2). Whereas the unnatural amino acids incorporated in peptides **38–47** represent enantiomerically pure derivatives of Ile and Leu, the incorporation of racemic *β*,*β*-dimethyl-tyrosine (*β*,*β*-diMe-Tyr) in position 11 yielded the epimers **48** and **49** (see Figure 2). In the case of **46** and **47**, a 2-ClTrt-Cl resin had to be used instead of a H-Leu-2-ClTrt resin. For the coupling of the carboxy-terminal amino acid, the 2-ClTrt-Cl resin was treated with the respective Fmoc amino acid and DIPEA in CH_2_Cl_2_ overnight. After quenching of unreacted starting material with MeOH, the loading of the resin with *β*-cyclopropyl-Ala (**46**) or *α*-methyl-Leu (**47**) was estimated to amount to 50% compared to the original loading of the resin with chloride. After side chain deprotection and cleavage from the resin, the overall-yields of **38–49** amounted to 15–74%. 

Replacement of the *N*^α^-methylated Arg^8^ in **48** and **49** by an *N*^α^-methylated, *N*^ω^-carbamoylated arginine derived from **6**, led to peptides **50** and **51**, and the additional replacement of Ile by Tle yielded **52** and **53** (Figure 2). Isolation of the epimers **50**/**51** and **52**/**53**, respectively, from one batch was necessary due to the usage of the same racemic *β*,*β*-diMe-Tyr building block as described for **48** and **49**. 

**Scheme 2:**
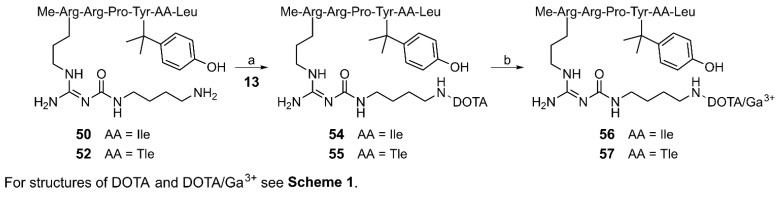
Synthesis of the potential NTS_1_R PET ligands **56** and **57** containing a *β*,*β*-dimethylated tyrosine. Reagents and conditions: (a) (1) DIPEA, solvent: DMF/NMP 80:20 *v*/*v*, rt, 30 min, (2) TFA/H_2_O 80:20 *v*/*v*, 50 °C, overnight, 76% (**54**), 79% (**55**); (b) preheating of a solution of the peptide (4 mM) in HEPES buffer (0.2 M, pH 4.2) to 60 °C, 5 min, addition of Ga(NO_3_)_3_ × H_2_O in 10 mM HCl, 100 °C, 30 min, 97% (**56**), 91% (**57**).

The amino-functionalized peptides **50** and **52** were treated with **13** as described above for the synthesis of **8–12** (*cf*. Scheme 1) to give the DOTA-conjugated compounds **54** and **55**, respectively, in high yields (76% and 79%) after subsequent Boc-deprotection (Scheme 2). Insertion of Ga^3+^ into the chelator moiety resulted in the PET ligand candidates **56** (UR-LS130) and **57** in high yields of 97% and 91%, respectively.

### 3.2. Circular Dichoism (CD) Analysis

**Figure 3:**
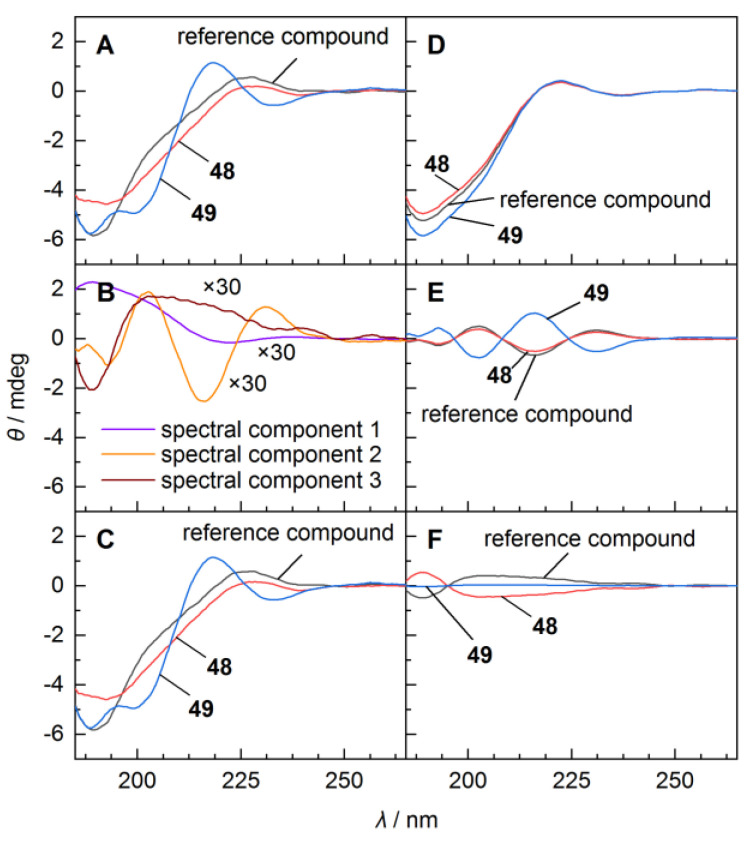
Circular dichroism (CD)-based identification of the absolute configuration of the dimethylated Tyr^11^ in **48** and **49** through spectral deconvolution and assignment of linearly independent spectral features to stereochemical and structural properties. (**A**) CD spectra of the two diastereomers **48** and **49** and an all-ʟ-reference compound (Me-Arg-Arg-Pro-Tyr-Ile-Leu [40]). (**B**) Linearly independent components (“abstract spectra”) from SVD, shared by the two diastereomers and the reference compound (rescaled by a factor of 30 for better comparison). (**C**) Reconstruction of full spectra from linear combination of the “abstract spectra” in panel B. (**D**) Contributions of component 1 to the full spectra in A or C correlating with CD contributions from the peptide backbone minus contributions from the fourth amino acid (tyrosine in the reference compound or *β*,*β*-dimethylated tyrosine in **48** and **49**). (**E**) Contributions of component 2 correlating with the configuration at the *α*-carbon of the fourth amino acid. Identical signs of the bands of this spectral component indicate the same configuration in **48** and the reference compound. (**F**) Contributions of component 3 accounting for spectral differences due to *β*,*β*-dimethylation that have not been considered in component 1 and component 2. As the SVD factorizes the experimental spectra with respect to highest spectral agreement, i.e., a ‘compromise’ spectrum formed from the spectra of the reference compound, **48** and **49**, absence of *β*,*β*-dimethylation in the reference compound is reflected by a negative contribution of component 3.

To determine the configuration at the α-carbon of the *β*,*β*-dimethylated tyrosine at position 11, we measured CD spectra of **48** and **49** and compared them to the CD spectrum of the peptide Me-Arg-Arg-Pro-Tyr-Ile-Leu [40], representing an all-l-configured reference compound with tyrosine instead of *β*,*β*-dimethylated tyrosine in position 11 as the only difference to **48** and **49** (Figure 3). To facilitate the assignment, we factorized the CD spectra into linearly independent spectral components by singular value decomposition (SVD), as described elsewhere [67]. As the three compounds differ in two properties, i.e., configuration at the α-carbon, *β*,*β*-dimethylation, or both, we expected three linear components, each contributing with a certain linear coefficient with either a positive or a negative sign. Indeed, performing the SVD on a set of nine spectra (three spectra each) resulted in three components being different from noise (*cf*. Figure 3B), whose reconstruction (under omission of linear components supposedly containing noise contribution only) resulted in nearly identical spectra as in Figure 3A (Figure 3C). Despite factorizing the SVD according to numerical variance and not to structural origin of spectral features, a rough assignment of the linear components was possible. Reconstruction of the spectra exclusively from spectral component 1 and the corresponding linear coefficients resulted in the spectra shown in Figure 3D. As this component represents the features with highest agreement between the three species, they are presumably associated with the backbone conformation of the peptides. In agreement with previous NMR and CD data on neurotensin in water [68], the maximum at 220 nm and the minimum at 190 nm indicate a lack of consecutive order in these peptides. After reconstruction of spectra with linear component 2, the resulting spectra for the reference compound and peptide **48** were nearly identical, whereas the spectrum reconstructed for **49** had the opposite sign. Therefore, component 2 is the one that indicates the configuration of the α-carbon, which allows assignment of the l-configuration to the *β*,*β*-dimethylated tyrosine in **48** and the d-configuration to the *β*,*β*-dimethylated tyrosine in **49**. Finally, component 3 accounts for all the remaining spectral differences between the three species such as contributions from presence or absence of *β*,*β*-dimethylation. The configuration (*R* or *S*) of the emethylated tyrosine in **50–53** was assigned based on the comparison of elution orders in RP-HPLC (**48**, **50** and **52** elute before **49**, **51** and **53**, respectively).

### 3.3. Peptide Stability in Human Plasma

The stability of compounds **8**, **9**, **11**, **12**, **14–23**, **38–49,** and **54–57** against proteolytic degradation was investigated in human plasma for up to 48 h as previously described [40]. For compounds **1–3** [40], **19–23**, **56,** and **57**, the amount of remaining intact peptide after incubation in plasma at 37 °C is shown in Table 2 (for plasma stability data of compounds **8–12**, **14–18**, **38–49**, **54** and **55** (Table S3) and recovery ratios of compounds **8**, **9**, **11**, **12**, **14–23,** and **54–57** (Table S2) see Supplementary Materials. 

The *N*^α^-unmethylated peptides **1** and **2** were reported to undergo very rapid degradation in plasma [40,43]. Therefore, N-terminal methylation or methylation of Arg^9^, both impairing NTS_1_R binding only to a minor extent [40], was applied to the synthesized peptides throughout.

In the initial set of prepared peptides (**8**, **9**, **11**, **12**, and **14–23**) low stability in plasma (≤15% intact peptide after 24 h) was found for compounds containing Ile in position 12 (**9**, **15** and **20**), confirming the importance of Tle^12^ for the stabilization of the C-terminus against proteolytic degradation. Compounds with Tle^12^, devoid of a Ga^3+^-occupied chelator (**8**, **10–12**, **14**, and **16–18**), showed a high stability towards proteolytic degradation (≥93% intact peptide after 24 h). However, insertion of Ga^3+^ (**19** and **21–23**) led to a considerable decrease in stability (≤46% intact peptide after 24 h, Table 2). This observation can be explained by changes in compound structure, hydrophilicity and charge distribution upon insertion of the gallium cation and rearrangement of the carboxylic arms of the chelators, thereby facilitating the recognition by proteases.

In the final set of compounds (**38–57**), high plasma stabilities (≥91% intact peptide after 24 h) were found for peptides **39**, **44**, **48,** and **49**, as well as for the labeling precursors **54** and **55**, the latter even showing >99% intact peptide after 48 h. Strikingly, peptides **48**, **49** and **54** do not comprise Tle but Ile in position 12. Therefore, the stabilizing effect must result from the diMe-Tyr in position 11. Insertion of Ga^3+^ in **54** and **55** (giving **56** and **57**) again provoked a substantial decrease in stability towards proteolytic degradation, nonetheless, the plasma half-lives of **56** and **57** were higher than those of the potential PET ligands **19–23** (*cf*. Table 2).

### 3.4. In Vitro Binding Studies at the NTS_1_R and NTS_2_R and NTS_1_R Agonistic Activities

Except for compounds **28** and **29**, NTS_1_R affinities were determined for all synthesized peptides (**8**, **9**, **11**, **12**, **14–23**, **25**, **26**, **32–50**, **52,** and **54–57**) in a radiochemical competition binding assay using intact HT-29 colon carcinoma cells expressing the hNTS_1_R [69], but not the NTS_2_R [39]. The previously described radioligand [^3^H]UR-MK300 [39] (structure see Figure S1, Supplementary Materials) was used as radioligand. Selected compounds (**1**, **3**, **8–11**, **14–17**, **19–22**, **48–53**, and **56**) were also investigated with respect to NTS_2_R binding using the same radioligand and HEK293T cells stably expressing the hNTS_2_R. The obtained *K*_i_ values are presented in Table 2 and Table S3 (Supplementary Materials). The Ile-containing peptides **9**, **15**, **20**, **50**, **54** and **56** yielded lower *K*_i_ values (NTS_1_R) than their respective Tle-containing analogs **8**, **14**, **19**, **52**, **55** and **57**, confirming the described affinity-decreasing effect of the Ile^12^/Tle^12^ exchange in NT(8-13) analogs [14,40,50,52,54,70,71]. Ga^3+^-containing DOTA-conjugated peptides consistently showed slightly higher NTS_1_ and NTS_2_ receptor affinities compared to the respective precursor compounds with an empty DOTA chelator (*cf*. Table 2 and Table S3, Supplementary Materials). In terms of NTS_1_R binding, the effect was most pronounced for the compound pairs **32**/**33** and **54**/**56** (4.8-fold and 4.9-fold increase in affinity upon insertion of Ga^3+^). This phenomenon is in agreement with reported findings for DOTA- and (1,4,7-triazacyclononane-4,7-diyl)diacetic acid-1-glutaric acid (NODA-GA)-conjugated ^68^Ga-labeled NTS_1_R ligands [16,42], which were explained by changes in ligand structure as discussed above for the reduced plasma stabilities of the Ga^3+^-containing peptides [16,42].

The difference with respect to the linker in the side chain of the *N*^ω^-carbamoylated arginines of compounds **10** and **12** did not affect NTS_1_R receptor affinity (*K*_i_ = 2.8 and 2.7 nM, respectively), but the change in the position of the *N*^α^-methylated and *N*^ω^-carbamoylated arginine (position 8 in **10**, position 9 in **11**) led to a slight decrease in NTS_1_R affinity from *K*_i_ = 2.8 nM (**10**) to 13 nM (**11**). The latter finding is in agreement with reports on the higher importance of Arg^9^ for NTS_1_R binding compared to Arg^8^ [41,72,73].

The initially prepared set of PET ligand candidates with Tle in position 12 (**19** and **21–23**) exhibited *K*_i_ values (NTS_1_R) in the range of 7.5–20 nM. The second set of potential PET ligands (**33**, **36**, and **37**), containing a lipophilic fluorinated biphenyl moiety, showed higher NTS_1_R affinities with *K*_i_ values of 2.5–4.2 nM. The position and the type of carbamoylated arginine played only a minor role in terms of NTS_1_R binding for the potential PET ligands and their precursors. Within the final set of potential PET ligands (**56** and **57**), containing *S*-configured *β*,*β*-diMe-Tyr in position 11, the peptide with Ile in position 12 (**56**) displayed excellent NTS_1_R binding (*K*_i_ = 1.2 nM), while its congener **57**, containing Tle in position 12, showed 18-fold lower NTS_1_R affinity (*K*_i_ = 21 nM).

The series of NT(8–13) derivatives containing various unnatural amino acids in position 11, 12 or 13 (**38–49**) was prepared to develop a PET ligand with improved in vivo stability. As the side chains of Ile^12^ and Leu^13^ in **1** were hypothesized to contribute to receptor binding via hydrophobic interactions with aliphatic residues in the binding pocket of the NTS_1_R [73,74], hydrophobic unnatural amino acids structurally related to Ile and Leu, were incorporated in position 12 or 13 (**38–47**, cf. Figure 2). The artificial amino acids were quite well tolerated with respect to NTS_1_R binding, provided that they contained no additional alkyl substituent at the α-carbon (**38**, **40–43**, and **46**). The incorporation of amino acids with an additional alkyl group (methyl, ethyl) at the α-carbon resulted in a loss of NTS_1_R binding (Table S3, Supplementary Materials). Strikingly, sub-nanomolar NTS_1_R affinity (*K*_i_ = 0.14 nM) was achieved with compound **48**, containing *S*-configured *β*,*β*-diMe-Tyr^11^ instead of Tyr^11^. As **48** showed also excellent in vitro plasma stability (see Table S3, Supplementary Materials), it served as a lead structure for the synthesis of the PET ligand candidates **56** and **57**. The epimer of **48** (peptide **49**), containing *R*-configured *β*,*β*-diMe-Tyr^11^, displayed considerably lower NTS_1_R binding compared to **48**. This was in agreement with the results of variations in position 11 of **1**, including the incorporation of d-configured tyrosine derivatives, revealing that d-configured tyrosine analogs caused a decrease in NTS_1_R binding [40,71,75,76,77].

All compounds that were investigated at the NTS_2_R showed lower *K*_i_ values at the NTS_1_R than at the NTS_2_R (difference most pronounced for peptide **15**: *K*_i_ values of 2.4 and 55 nM, respectively) revealing moderate NTS_1_R selectivity (Table 2). However, it is unlikely that low or missing NTS_1_R selectivity hampers the imaging of NTS_1_R-expressing tumors in the periphery, as the NTS_2_R is primarily expressed in the central nervous system [78,79,80].

In addition to the investigation of NTS_1_R and NTS_2_R binding, the agonistic activities of **21** and **56** at the G_q_-coupled NTS_1_R were determined in a Fura-2 Ca^2+^-assay using HT-29 colon carcinoma cells. The potential PET ligands **21** and **56** proved to be full agonists with maximal responses comparable to that of **1** (see Figure S6, Supplementary Materials). As also found for **1**, the NTS_1_R agonistic potencies of **21** and **56** were lower compared to their NTS_1_R binding affinities (*cf*. Table 2 and Figure S6). A plausible explanation for this observation is in the non-equilibrium conditions in the case of the functional Ca^2+^-assay precluding a complete association of the agonist to the receptor (signal is recorded within 3 min after agonist addition), which must be compensated by higher agonist concentrations.

### 3.5. Radiosynthesis and Distribution Coefficients

The potential PET tracers **21**, **33**, **37** and **56** all showed high NTS_1_R affinity and high in vitro plasma stability. Thus, radiolabeling with ^68^Ga^3+^ was performed to prepare the respective PET tracers, i.e., [^68^Ga]**21**, [^68^Ga]**33**, [^68^Ga]**37** and [^68^Ga]**56**. This selection included two highly polar PET ligands ([^68^Ga]**21**, [^68^Ga]**56**) and two ligands bearing a lipophilic spacer ([^68^Ga]**33**, [^68^Ga]**37**), which can possibly result in different pharmacokinetic properties.

Radiosynthesis was performed by incubation of the precursor compounds **16**, **32**, **35** or **54** (7.5–15 nmol) in HEPES buffer pH 5.5 with [^68^Ga]GaCl_3_ at 125 °C for 6 or 16 min (for details see Table 1). After the synthesis, the PET tracers were separated from the respective precursor using an analytical HPLC system in order to increase specific activity and to obtain a homogenous tracer preparation. The isolation from the precursors was feasible on a C18 reversed-phase material with mixtures of MeOH and 0.1% formic acid as mobile phase (comparable conditions as used for the purification of the “cold” PET ligands). For an exemplary chromatogram of the micro-preparative HPLC see Figure S7 (Supplementary Materials). Radiosynthesis and evaporation of the solvent was accomplished in approximately 90 min, and the separation of the PET tracer from the precursor including the second evaporation step took approximately another 50–60 min. 

### 3.6. Biodistribution of PET Ligands [^68^Ga]***21***, [^68^Ga]***33***, [^68^Ga]***37*** and [^68^Ga]***56***, and Cellular Uptake of [^68^Ga]***56***

The hydrophilicity values of the PET ligands [^68^Ga]**21**, [^68^Ga]**33**, [^68^Ga]**37** and [^68^Ga]**56** were evaluated by the determination of the n-octanol/PBS distribution coefficients logD_7.4_, which amounted to −3.87, −2.48, −2.59 and −3.06, respectively. Notably, compared to the most polar PET ligand ([^68^Ga]**21**), the introduction of a lipophilic spacer ([^68^Ga]**33**, [^68^Ga]**37**) only led to an increase in logD_7.4_ by less than 1.4 log units. 

**Figure 4:**
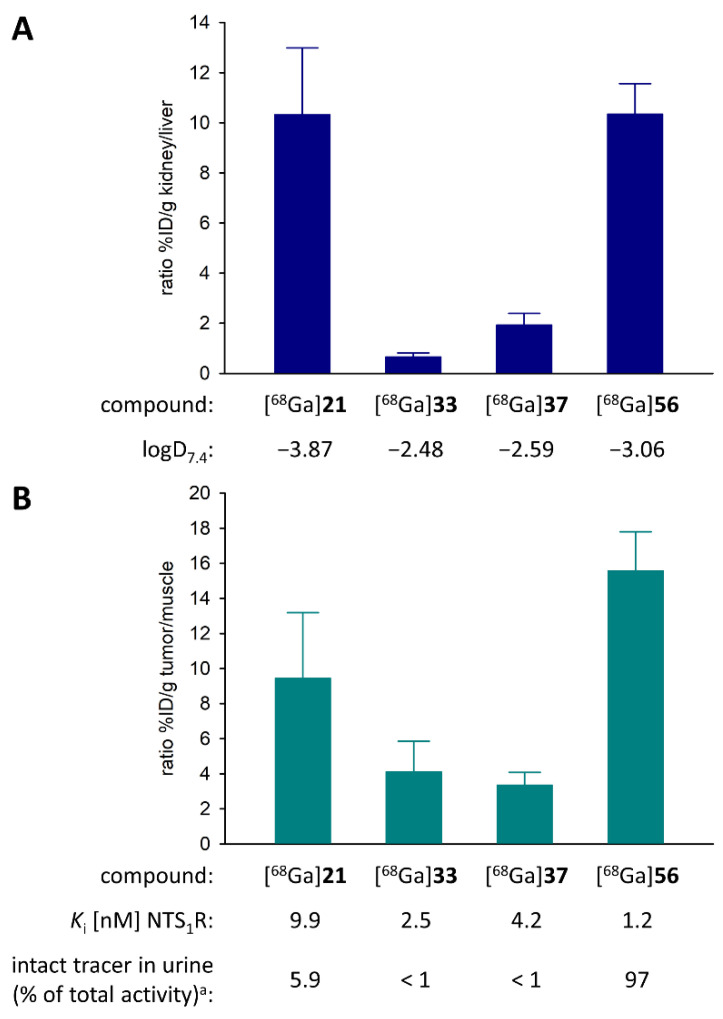
Ratio of the %ID/g values of (**A**) kidney and liver and of (**B**) tumor and muscle, obtained from biodistribution experiments with [^68^Ga]**21**, [^68^Ga]**33**, [^68^Ga]**37** and [^68^Ga]**56** in HT-29 tumor-bearing mice. Given are mean values ± SD from three ([^68^Ga]**33**, [^68^Ga]**37**) or four ([^68^Ga]**21**, [^68^Ga]**56**) independent experiments. ^a^ Given is the relative area (as percentage of the total peak area) of the peak corresponding to the intact PET ligand in the radiochromatogram of the HPLC analysis of the urine sample obtained 45 min after injection of the tracer.

Biodistribution studies in HT-29 tumor-bearing nude mice were firstly performed with [^68^Ga]**21** and resulted in a tumor-to-muscle ratio of 9.5 at 45 min after injection of the tracer (*cf*. Figure S8 and Table S4, Supplementary Materials). This result was comparable with the data reported for studies of the ^68^Ga-labeled NTS_1_R PET ligands [^68^Ga]**4** [41] and [^68^Ga]**5** [42] in the same xenograft mouse model (tumor-to-muscle ratios 60 min p.i.; approximately 14 and 8.8, respectively). As the fast renal elimination of [^68^Ga]**21** potentially compromises its accumulation in the tumor, a second attempt with the less hydrophilic tracers [^68^Ga]**33** and [^68^Ga]**37**, potentially exhibiting longer systemic circulation, which could, in turn, result in an increased tumor uptake, was made. Although the introduction of the hydrophobic spacer in [^68^Ga]**33** and [^68^Ga]**37** had a marked impact on the predominant way of tracer elimination (shift from renal to nearly balanced renal and hepatobiliary excretion, *cf*. Figure 4A), tumor-to-muscle ratios 45 min p.i. (4.1 ([^68^Ga]**33**) and 3.4 ([^68^Ga]**37**)) were diminished even in comparison to [^68^Ga]**21** (*cf*. Figure S8 and Table S4, Supplementary Materials). These results show that shifting the elimination pathway towards hepatobiliary excretion by decreasing the hydrophilicity of the tracer does not warrant an enhanced accumulation in the tumor, which might be explained by retained fast elimination despite an altered route of excretion. Irrespective of their different physicochemical properties, [^68^Ga]**21**, [^68^Ga]**33** or [^68^Ga]**37** showed low in vivo stability, as concluded from HPLC analyses of urine samples revealing that intact [^68^Ga]**21**, [^68^Ga]**33** or [^68^Ga]**37** accounted for less than 6% of the radioactivity in urine 45 min after tracer injection (Figure S9, Supplementary Materials). Notably, for these tracers, only one main metabolite was found in the urine samples, being more polar than the respective intact tracer. 

**Figure 5:**
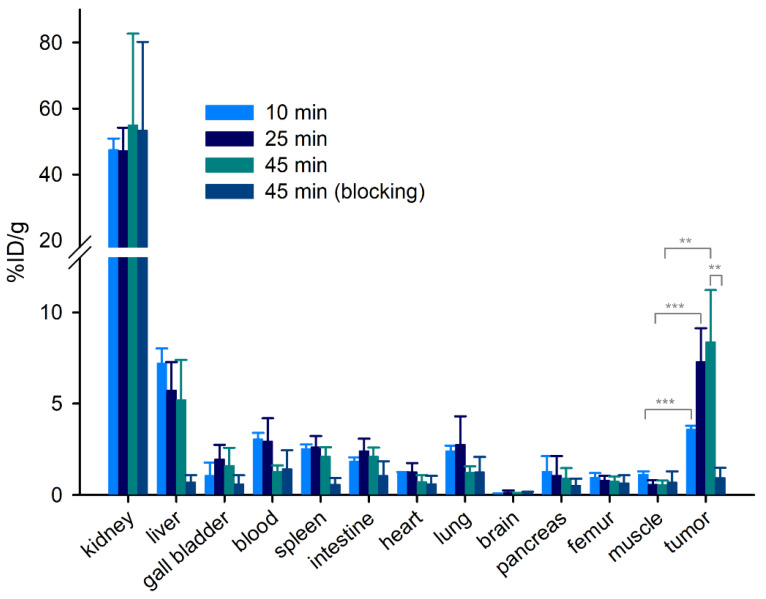
Biodistribution data (%ID/g tissue) of [^68^Ga]**56** obtained from HT-29 tumor-bearing mice. Given are mean values ± SD (n = 4). Blocking data were obtained by co-injection of **48** (560–700 nmol/mouse). Statistical analysis of differences between tracer accumulation in the tumor of unblocked and blocked animals was performed by an unpaired two-tailed *t*-test (*p* <0.05 was considered statistically significant). ** *p* <0.01. *** *p* <0.001.

Prompted by these findings, the focus was set on the development of NT(8-13) derived PET tracers with higher proteolytic stability, as a low in vivo stability of the tracers could also be a major reason (other than fast elimination) for the low tracer accumulation in the tumor. The preparation and in vitro characterization of peptides **38–49** brought forth the potential PET tracer **56** exhibiting high NTS_1_R affinity (*K*_i_ = 1.2 nM) and high in vitro plasma stability (Table 2). The biodistribution of [^68^Ga]**56** in HT-29 tumor-bearing nude mice was investigated at 10, 25, and 45 min p.i. (Figure 5 and Table 3). Blocking experiments were performed for the time of highest tumor-uptake (45 min p.i.). At 10 min after injection of [^68^Ga]**56**, the major fraction of activity was found in the kidneys (47% ID/g), remaining at this level over time (47% ID/g and 55% ID/g after 25 min and 45 min, respectively). The activity in the liver was much lower (7.2, 5.7 and 5.2% ID/g after 10, 25 and 45 min, respectively), indicating predominant renal excretion of [^68^Ga]**56**, which was expected for this highly hydrophilic tracer (Table 3, Figure 4A). The activity in the blood dropped from 3.1% ID/g (10 min p.i.) to 1.3% ID/g (45 min p.i.), while it increased in the tumor from 3.6% ID/g (10 min) over 7.3% ID/g (25 min) to 8.4% ID/g (45 min). Tumor-to-muscle ratios increased over time (3.3 at 10 min p.i. and 13 at 25 min p.i.), reaching a value of 16 after 45 min, which was considerably higher than the tumor-to-muscle ratios of [^68^Ga]**21**, [^68^Ga]**33** and [^68^Ga]**37** at 45 min p.i. (Table 3 and Table S4, Supplementary Materials). Co-injection of the non-labeled compound **48** with [^68^Ga]**56** (blocking experiments) resulted in a tumor-to-muscle ratio of 1.8 (45 min p.i.), indicating that the uptake of [^68^Ga]**56** in the tumor was NTS_1_R-mediated. To evaluate the in vivo stability of [^68^Ga]**56**, HPLC analyses of urine samples obtained 10 min and 45 min after tracer injection were performed, revealing that, in contrast to [^68^Ga]**21**, [^68^Ga]**33** and [^68^Ga]**37**, intact [^68^Ga]**56** accounted for more than 80% of the activity in urine (*cf*. Figure S11B and Figure S10, Supplementary Materials). The high in vivo stability of [^68^Ga]**56**, which obviously contributes to an increased tracer accumulation in the tumor, was confirmed by HPLC analysis of an ex vivo blood plasma sample at 10 min p.i. showing that intact [^68^Ga]**56** (identity confirmed by spiking of the plasma sample with **56**) accounted for more than 80% of the radioactivity in the processed sample (*cf*. Figure S11C–E, Supplementary Materials).

In reported studies, the ^111^In-labeled analog of compound **4** was investigated in terms of in vivo stability 15 min after injection in mice, revealing 22% of remaining intact tracer in the plasma sample [41]. The ^68^Ga-labeled analog of **5**, investigated in vitro in human serum, gave 93% intact tracer after 60 min of incubation [42]. In vivo studies in mice with a ^68^Ga-labeled tracer structurally closely related to **5**, showing low NTS_1_R affinity (*K*_i_ = 180 nM), resulted in 90% intact tracer in blood plasma 10 min after tracer administration [16]. Noteworthy, a recently reported ^18^F-labeled fluoroglycosylated NTS_1_R PET ligand derived from **10**, containing the same peptide core structure as [^68^Ga]**21**, [^68^Ga]**33** and [^68^Ga]**37**, exhibited low in vivo stability in mice (30% of remaining intact tracer in plasma 10 min p.i., and no detectable tracer in plasma 20 min p.i.) [60]. Consequently, the high in vivo stability accomplished with [^68^Ga]**56** in combination with retained high NTS_1_R affinity, represents an important achievement in the field of peptidic NTS_1_R PET ligands.

**Figure 6:**
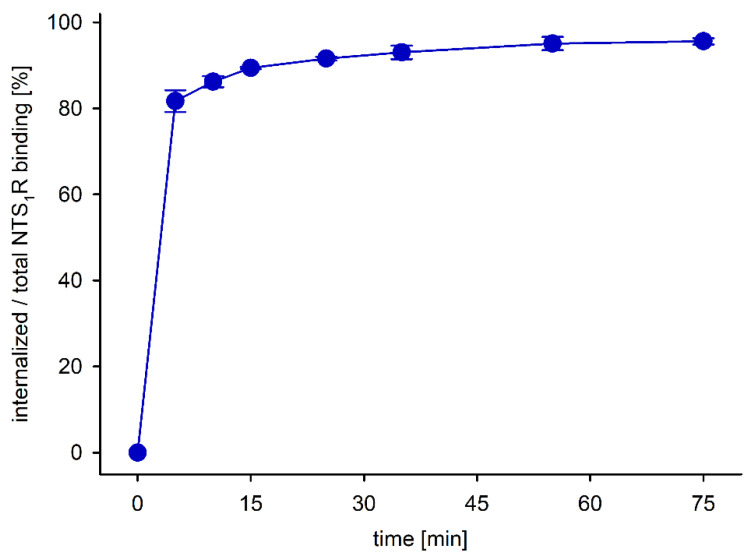
Fraction of specifically bound and internalized [^68^Ga]**56** in HT-29 cells relative to the entire specific binding determined at 37 °C. Given are mean values ± SD of two independent experiments, each performed in triplicate.

To estimate the internalization rate of NTS_1_ receptors occupied by [^68^Ga]**56**, HT-29 tumor cells were incubated with [^68^Ga]**56** at 37 °C for up to 75 min followed by removal of extracellularly bound peptidic receptor ligand using the acid-trip method. This experiment showed that the fraction of internalized tracer was >80% after only 5 min of incubation, reaching a plateau of approximately 95% after 55 min (Figure 6). This feature is considered favorable with respect to an accumulation of the tracer in the tumor in vivo, particularly when it comes to therapeutic applications using, e.g., alpha-emitting tracers such as ^225^Ac-labeled radiopharmaceuticals.

### 3.7. PET/CT Imaging with [^68^Ga]***56***

**Figure 7:**
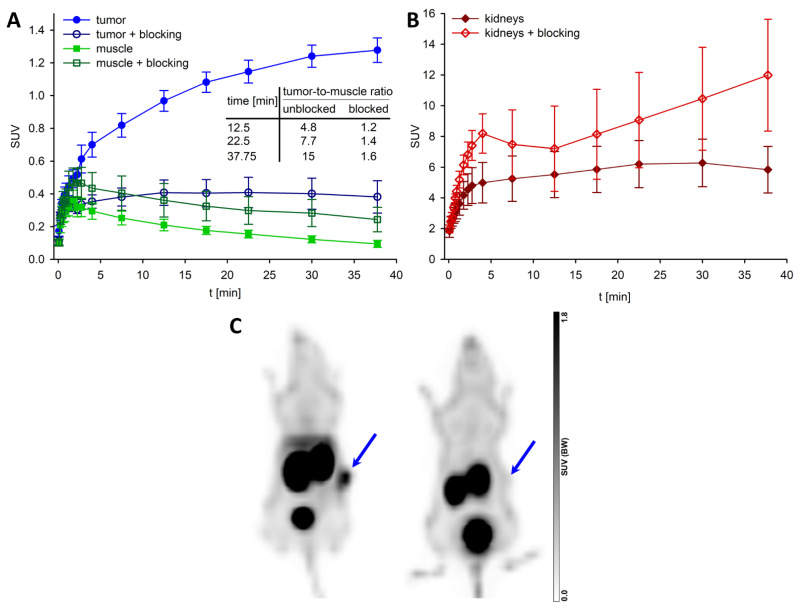
TACs and representative PET images from studies with [^68^Ga]**56** in HT-29 tumor-bearing mice. (**A**) SUV_mean_ ± SD (n = 4) from 45 min-PET scans for the tumor (circles) and muscle (squares), and tumor-to-muscle ratios for selected times calculated based on the SUV values (inset table). Blocking data were obtained by co-injection of **48** (560–700 nmol/mouse). (**B**) SUV_mean_ ± SD (n = 4) for the kidneys (same PET scans as under (**A**)). (**C**) Maximum intensity projections of two representative PET images of HT-29 tumor-bearing mice after injection of [^68^Ga]**56** (left) or co-injection of [^68^Ga]**56** and **48** (right) (time frame: 10–45 min p.i.). The blue arrows indicate the tumors.

Dynamic PET scans of HT-29 tumor-bearing nude mice injected with [^68^Ga]**56** were performed for 45 min. Notably, a PET/CT scanner (Siemens Biograph mCT-S(40)) for clinical routine tumor diagnostics in patients was used for these studies. It was shown previously that this instrument is applicable for imaging of small animals with sufficiently large tumor implants [65,81]. Blocking experiments were carried out by co-injection of an excess of the NTS_1_R ligand **48**. Time-activity-curves (TACs) for the tumor and muscle (from non-blocking and blocking experiments) as well as for the kidneys, generated from the SUVs acquired for the respective ROIs, are depicted in Figure 7A,B. These data confirmed the results from the biodistribution studies with [^68^Ga]**56**: the tracer uptake in the tumor increased over time and the activity level in the kidneys reached a plateau after approximately 10 min. Thus, tumor-to-muscle ratios, determined from the SUVs (*cf*. inset table in Figure 7A), were comparable with the tumor-to-muscle ratios obtained from biodistribution studies. The blocking experiments confirmed the specific (NTS_1_R-mediated) uptake of [^68^Ga]**56** in the tumor (Figure 7A). Representative PET images of tumor-bearing mice injected with [^68^Ga]**56** alone or with [^68^Ga]**56** and an excess of **48** are shown in Figure 7C. Accumulation of the tracer in the tumor was clearly visible.

## 4. Conclusions

We herein describe the preparation, analysis and biological characterization of a series of peptidic PET tracer candidates, which led to the discovery of the DOTA(Ga^3+^)-conjugated NTS_1_R ligand UR-LS130 (**56**) showing high stability in human plasma (*t*_1/2_ >24 h) and higher NTS_1_R affinity (*K*_i_ = 1.2 nM) compared to previously reported NTS_1_R PET ligands with high in vitro plasma stability. A novel feature of this Ga^3+^-containing peptidic PET ligand is the attachment of the chelator via the side chain of an arginine. [^68^Ga]**56** displayed high in vivo stability and a clear accumulation in NTS_1_R-expressing HT-29 tumors. Notably, **56** contains no Tle, but Ile in position 12, like endogenous neurotensin. Instead, **56** contains a *β*,*β*-diMe-Tyr in position 11, leading to excellent stability in vitro and in vivo. To date, replacement of Ile^12^ by Tle^12^ is the state of the art to achieve proteolytic stabilization of the C-terminus of NT(8-13) derived PET tracers. However, in the present study, we show that the Ile^12^/Tle^12^ exchange can be insufficient to achieve high in vivo stability. Unlike the Ile^12^/Tle^12^ exchange, which affects NTS_1_R binding, the recently introduced alternative based on the exchange of Leu^13^ by trimethylsilylalanine proved to be beneficial with respect to NTS_1_R binding, but turned out to be less favorable than the Tle^12^-approach regarding proteolytic stability [38]. In contrast, the new diMe-Tyr^11^-approach combines retained NTS_1_R affinity and high tracer stability. Taking into consideration the reported impact of the metal ion chelator on a tracer’s biodistribution profile and accumulation in the tumor [37,82], further improvement of the tracer [^68^Ga]**56** could be undertaken by conjugation to a chelator different from DOTA. With [^68^Ga]**56** we present the first peptidic NTS_1_R PET ligand with high in vivo stability exhibiting comparable NTS_1_R affinity as reported ^177^Lu-labeled NTS_1_R antagonists [83,84], which are favored over peptides for tumor endoradiotherapy due to higher in vivo stability [85]. Thus, the achievements of this work could promote the development of NT(8-13)-derived radiotherapeutics for cancer treatment, as an alternative to ^177^Lu-labeled NTS_1_R antagonists.

## Figures and Tables

**Table 1:** PET ligand specific parameters for the radiosynthesis of [^68^Ga]**21**, [^68^Ga]**33**, [^68^Ga]**37** and [^68^Ga]**56**.

PET Ligand	Labeling Precursor	Amount of Precursor	Total Product Activity [MBq] ^a^	Decay Corrected Radio-Chemical Yield (%) ^b^	HPLC Analysis: ^b^ Purity, *t*_R_, k
[^68^Ga]**21**	**16**	10–20 µg, 7.5–15.0 nmol	78.71–111.4 MBq	67–77	97–99%, 8.1–8.2 min, 4.8–4.9
[^68^Ga]**33**	**32**	20 µg, 11.5 nmol	93.51 MBq	75	95%, 8.1 min, 4.8
[^68^Ga]**37**	**35**	20 µg, 12.2 nmol	69.05 MBq	77	99%, 7.8 min, 4.6
[^68^Ga]**56**	**54**	20 µg, 14.7 nmol	95.80–168.6 MBq	67–80	92–99%, 7.3–7.4 min, 4.2–4.3

^a^ Activity after separation of the PET tracer from the precursor by HPLC, removal of the solvent and uptake in PBS; given is the range over the performed syntheses. ^b^ Given is the range over the performed syntheses.

**Table 2:** NTS_1_R affinities of **1–3**, **19–23**, **33**, **36**, **37**, **56,** and **57**, NTS_2_R affinities of **1**, **3**, **19–22** and **56**, NTS_1_R selectivities of **1**, **3**, **19–22** and **56**, and in vitro plasma stabilities of **1–3**, **19–23**, **56,** and **57**, determined at 37 °C.

cpd.	p*K*_i_ ± SD/*K*_i_ [nM] NTS_1_R ^a^	p*K*_i_ ± SD/*K*_i_ [nM] NTS_2_R ^b^	NTS_1_R Selectivity (Ratio *K*_i_ (NTS_2_R)/*K*_i_ (NTS_1_R))	% Intact Peptide in Plasma after the Given Incubation Time ^c^				
				10 min	1 h	6 h	24 h	48 h
**1**	9.49/0.33 ^d^	8.61 ± 0.09/2.5	7.6	23 ^d^	<1 ^d^	n.d.	<1 ^d^	<1 ^d^
**2**	8.93/1.2 ^d^	n.d.	-	11 ^d^	<1 ^d^	n.d.	<1 ^d^	<1 ^d^
**3**	9.07/0.88 ^d^	8.01 ± 0.07/9.9	11	n.d.	>99 ^d^	>99 ^d^	98 ^d^	87 ^d^
**19**	7.80 ± 0.03/16	7.16 ± 0.18/73	4.6	n.d.	>99	>99	36 ± 1	4.2 ± 0.6
**20**	8.70 ± 0.10/2.0	7.70 ± 0.09/20	10	n.d.	>99	77 ± 2	15 ± 1	4.6 ± 0.1
**21**	8.01 ± 0.08/9.9	7.25 ± 0.15/59	6.0	n.d.	>99	>99	26 ± 1	4.1 ± 0.1
**22**	7.70 ± 0.07/20	7.08 ± 0.16/88	4.4	n.d.	>99	87 ± 2	30 ± 1	7.7 ± 1.0
**23**	8.13 ± 0.11/7.5	n.d.	-	n.d.	>99	98 ± 6	46 ± 2	7.8 ± 0.6
**33**	8.61 ± 0.07/2.5	n.d.	-	n.d.	n.d.	n.d.	n.d.	n.d.
**36**	8.53 ± 0.02/3.0	n.d.	-	n.d.	n.d.	n.d.	n.d.	n.d.
**37**	8.38 ± 0.03/4.2	n.d.	-	n.d.	n.d.	n.d.	n.d.	n.d.
**56**	8.93 ± 0.17/1.2	8.35 ± 0.27/5.2	4.3	n.d.	>99	>99	77 ± 1	<1
**57**	7.67 ± 0.04/21	n.d.	-	n.d.	>99	>99	68 ± 1	<1

^a^ Determined by radioligand competition binding with [^3^H]UR-MK300 at HT-29 cells (*K*_d_ = 0.55 nM [40] or 0.41 nM, c = 1 nM); given are mean values ± SD (p*K*_i_) and mean values (*K*_i_) from two (**33**, **36**, and **37**), three (**19**, **20**, **56**, and **57**) or four (**21–23**) independent experiments, each performed in triplicate. ^b^ Determined by radioligand competition binding with [^3^H]UR-MK300 at HEK293T-hNTS_2_R cells (*K*_d_ = 6.9 nM or 4.0 nM, c = 10 nM); given are mean values ± SD (p*K*_i_) and mean values (*K*_i_) from two (**1**, **3**, and **21**), three (**20**) or four (**19**, **22**, and **56**) independent experiments, each performed in triplicate. ^c^ The initial concentration of the peptide in human plasma/PBS (1:2 *v*/*v*) was 100 µM. Data represent means ± SD from two or three independent experiments (SD not given when no decomposition was observed). ^d^ Schindler et al. [40].

**Table 3:** Ex vivo biodistribution data and tumor-to-muscle ratios of [^68^Ga]**56** obtained from HT-29 tumor-bearing mice ^a^.

Tissue	Uptake (%ID/g) at Given Times p.i.			
	10 min	25 min	45 min	45 min (Blocking)
kidney	47 ± 3.4	47 ± 7.0	55 ± 28	53 ± 27
liver	7.2 ± 0.83	5.7 ± 1.6	5.2 ± 2.2	0.69 ± 0.39
gall bladder (bile)	1.1 ± 0.71	1.9 ± 0.79	1.6 ± 0.99	0.59 ± 0.49
blood	3.1 ± 0.34	2.9 ± 1.3	1.3 ± 0.33	1.4 ± 1.0
spleen	2.5 ± 0.25	2.6 ± 0.60	2.1 ± 0.52	0.56 ± 0.35
intestine	1.8 ± 0.22	2.4 ± 0.68	2.1 ± 0.49	1.0 ± 0.80
heart	1.2 ± 0.10	1.3 ± 0.49	0.72 ± 0.36	0.60 ± 0.44
lung	2.4 ± 0.30	2.8 ± 1.5	1.2 ± 0.34	1.3 ± 0.83
brain	0.079 ± 0.017	0.13 ± 0.11	0.061 ± 0.049	0.10 ± 0.074
pancreas	1.3 ± 0.83	1.1 ± 1.1	0.90 ± 0.57	0.51 ± 0.37
femur	0.95 ± 0.25	0.78 ± 0.27	0.73 ± 0.29	0.65 ± 0.43
muscle	1.1 ± 0.18	0.57 ± 0.23	0.55 ± 0.23	0.68 ± 0.61
tumor	3.6 ± 0.20	7.3 ± 1.8	8.4 ± 2.9	0.94 ± 0.55
tumor-to-muscle	3.3 ± 0.46	13 ± 3.0	16 ± 2.2	1.8 ± 0.78

^a^ Given are mean values ± SD (n = 4). Blocking data were obtained by co-injection of **48** (560–700 nmol/mouse).

## Data Availability

The data presented in this study are available in this article.

## Supplementary Materials

The following supporting information can be downloaded at: https://www.mdpi.com/article/10.3390/cancers14194922/s1; Figure S1: Structure of the tritium-labeled NT(8-13)-derived radioligand [^3^H]UR-MK300 used for NTS_1_R and NTS_2_R binding studies; Figure S2: Representative saturation isotherms and unspecific binding curves from experiments with two batches of [^3^H]UR-MK300 at HEK293T-hNTS_2_R cells, giving *K*_d_ values of (A) 6.9 ± 1.8 nM (mean value ± SD from six independent determinations, each performed in triplicate) and (B) 4.0 ± 1.5 nM (mean value ± SD from three independent determinations, each performed in triplicate); Figure S3: Radioligand displacement curves from competition binding experiments with [^3^H]UR-MK300 (*K*_d_ = 0.55 nM or 0.41 nM, c = 1 nM) and **8**, **9**, **11**, **12**, **14**-**23**, **25**, **26** and **32**-**37** at intact hNTS_1_R-expressing HT-29 cells. Amino-functionalized precursor peptides are represented by circles, DOTA-conjugated peptides are represented by triangles, and Ga^3+^-containing compounds are represented by squares. Data represent mean values ± SD from at least two independent experiments (performed in triplicate); Figure S4: Radioligand displacement curves from competition binding experiments with [^3^H]UR-MK300 (*K*_d_ = 0.41 nM, c = 1 nM) and **38**-**50**, **52** and **54**-**57** at intact hNTS_1_R-expressing HT-29 cells. Amino-functionalized precursor peptides are represented by circles, DOTA-conjugated peptides are represented by triangles, and Ga^3+^-containing compounds are represented by squares. Data represent mean values ± SD from at least two independent experiments (performed in triplicate); Figure S5: Radioligand displacement curves from competition binding experiments with [^3^H]UR-MK300 (*K*_d_ = 6.9 nM or 4.0 nM, c = 10 nM) and **1**, **3**, **8**-**11**, **14**-**17**, **19**-**22**, **48**-**53** or **56** at intact HEK293T-hNTS_2_R cells. Reference compounds and amino-functionalized precursor peptides are represented by circles, DOTA-conjugated peptides are represented by triangles, and Ga^3+^-containing compounds are represented by squares. Data represent mean values ± SD from at least two independent experiments (performed in triplicate); Figure S6: Concentration response curves and agonistic potencies (pEC_50_, EC_50_) of **1**, **21** and **56** from fura-2 Ca^2+^-assays using intact hNTS_1_R-expressing HT-29 cells. Data represent mean values ± SD from three or four independent experiments (performed in singlet); Figure S7: Chromatogram of the RP-HPLC analysis of a mixture of the labeling precursor **32** and the "cold" PET ligand **33** (50 µM each, injection volume 75 µL) (black line), and chromatogram of the preparative HPLC run for the separation of the PET tracer [^68^Ga]**33** from **32** after radiosynthesis (blue line). The vertical blue lines give the beginning and the end of tracer collection; Figure S8: Biodistribution data (%ID/g tissue) of [^68^Ga]**21**, [^68^Ga]**33** and [^68^Ga]**37** from HT-29 tumor bearing mice. Given are mean values ± SD (n = 3 ([^68^Ga]**33**, [^68^Ga]**37**) or n = 4 ([^68^Ga]**21**)) gained at 45 min p.i; Figure S9: Representative chromatograms of the RP-HPLC quality controls of the PET tracers [^68^Ga]**21** (A), [^68^Ga]**33** (B) and [^68^Ga]**37** (C) after radiosynthesis (black lines) and of the RP-HPLC analyses of urine samples obtained from mice 45 min after injection of the respective PET tracers; Figure S10: RP-HPLC analysis of an ex vivo urine sample from a mouse 45 min after injection of [^68^Ga]**56**, spiked with **56** (100 µM). The blue line shows radiodetection and the black line shows UV detection at 220 nm; Figure S11: Chromatograms of the RP-HPLC analyses of different samples of [^68^Ga]**56** using radio- or UV-detection. (A) quality control after radiosynthesis; (B) ex vivo urine sample obtained from a mouse 10 min after injection of [^68^Ga]**56**; (C) ex vivo plasma sample (10 min p.i.) from the same mouse as under B; (D) UV-detection of the analysis from C; (E) plasma sample from C and D spiked with **56** (100 µM); Table S1: Equivalents and conditions applied for SPPS; Table S2: Recoveries of peptides **8**, **9**, **11**, **12**, **14**-**23** and **54**-**57** from human plasma/PBS (1:2 v/v) and ratios of peptide-recovery over recovery of IS; Table S3: NTS_1_R affinities of **8**-**12**, **14**-**18**, **25**, **26**, **32**, **34**, **35**, **38**-**50**, **52**, **54** and **55**, NTS_2_R affinities of **8**-**11**, **14**-**17** and **48**-**53**, NTS_1_R selectivities of **8**-**11**, **14**-**17**, **48**-**50** and **52**, and in vitro plasma stabilities of **8**-**12**, **14**-**18**, **38**-**49**, **54** and **55**, determined at 37 °C; Table S4: Ex vivo biodistribution data and tumor-to-muscle ratios of [^68^Ga]**21**, [^68^Ga]**33** and [^68^Ga]**37**; Synthesis protocols and analytical data of compounds **8**, **9**, **11**, **12**, **14–23**, **25**, **26**, **28**, **29** and **31–57**; RP-HPLC chromatograms of compounds **8**, **9**, **11**, **12**, **14–23**, **25**, **26**, **28**, **29** and **31–57**; ^1^H-NMR spectra of compounds **8**, **9**, **11**, **12**, **14–23**, **25**, **26**, **28**, **29** and **32–57**, and ^13^C-NMR spectra of compounds **50** and **51**.
