# Supplementary material for: Development of a Neurotensin-Derived 68Ga-Labeled PET Ligand with High In Vivo Stability for Imaging of NTS1 Receptor-Expressing Tumors
Source: Cancers (Basel). 2022 Oct 8;14(19):4922. doi: 10.3390/cancers14194922 (PMC9564337; doi:10.3390/cancers14194922)
Supplement: Supplementary file 1 [file cancers-14-04922-s001.zip › cancers-1902756-supplementary.pdf]

Supplementary Materials

Development of a Neurotensin-Derived ^{68}Ga-Labeled PET Ligand with High In Vivo Stability for Imaging of NTS_1 Receptor-Expressing Tumors

Lisa Schindler, Jutta Moosbauer, Daniel Schmidt, Thilo Spruss, Lukas Grätz, Steffen Lüdeke, Frank Hofheinz, Sebastian Meister, Bernd Echtenacher, Günther Bernhardt, Jens Pietzsch, Dirk Hellwig and Max Keller

Content Page

1. Figures S1–S11 and Tables S1–S4 S2
2. Synthesis protocols and analytical data of compounds **8**, **9**, **11**, **12**, **14-23**, **25**, **26**, **28**, **29** and **31-57** S15
3. RP-HPLC chromatograms of compounds **8**, **9**, **11**, **12**, **14-23**, **25**, **26**, **28**, **29** and **31-57** S37
4. ^{1}H-NMR spectra of compounds **8**, **9**, **11**, **12**, **14-23**, **25**, **26**, **28**, **29** and **32-57**, and ^{13}C-NMR spectra of compounds **50** and **51** S52
5. References S76

1. Figures S1–S11 and Tables S1–S4.

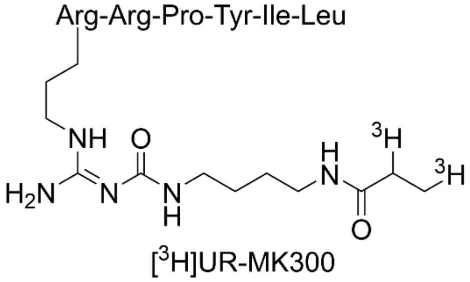

Figure S1. Structure of the tritium-labeled NT(8-13)-derived radioligand [^{3}H]UR-MK300 used for NTS_1R and NTS_2R binding studies [1].

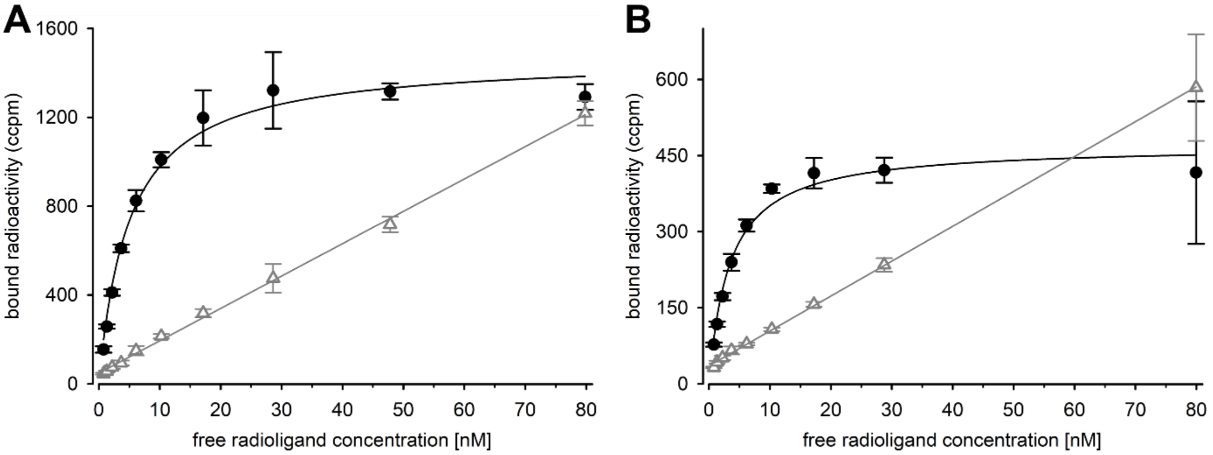

Figure S2. Representative saturation isotherms and unspecific binding curves from experiments with two batches of [^{3}H]UR-MK300 at HEK293T-hNTS_2R cells, giving K_d values of (A) 6.9 ± 1.8 nM (mean value ± SD from six independent determinations, each performed in triplicate) and (B) 4.0 ± 1.5 nM (mean value ± SD from three independent determinations, each performed in triplicate).

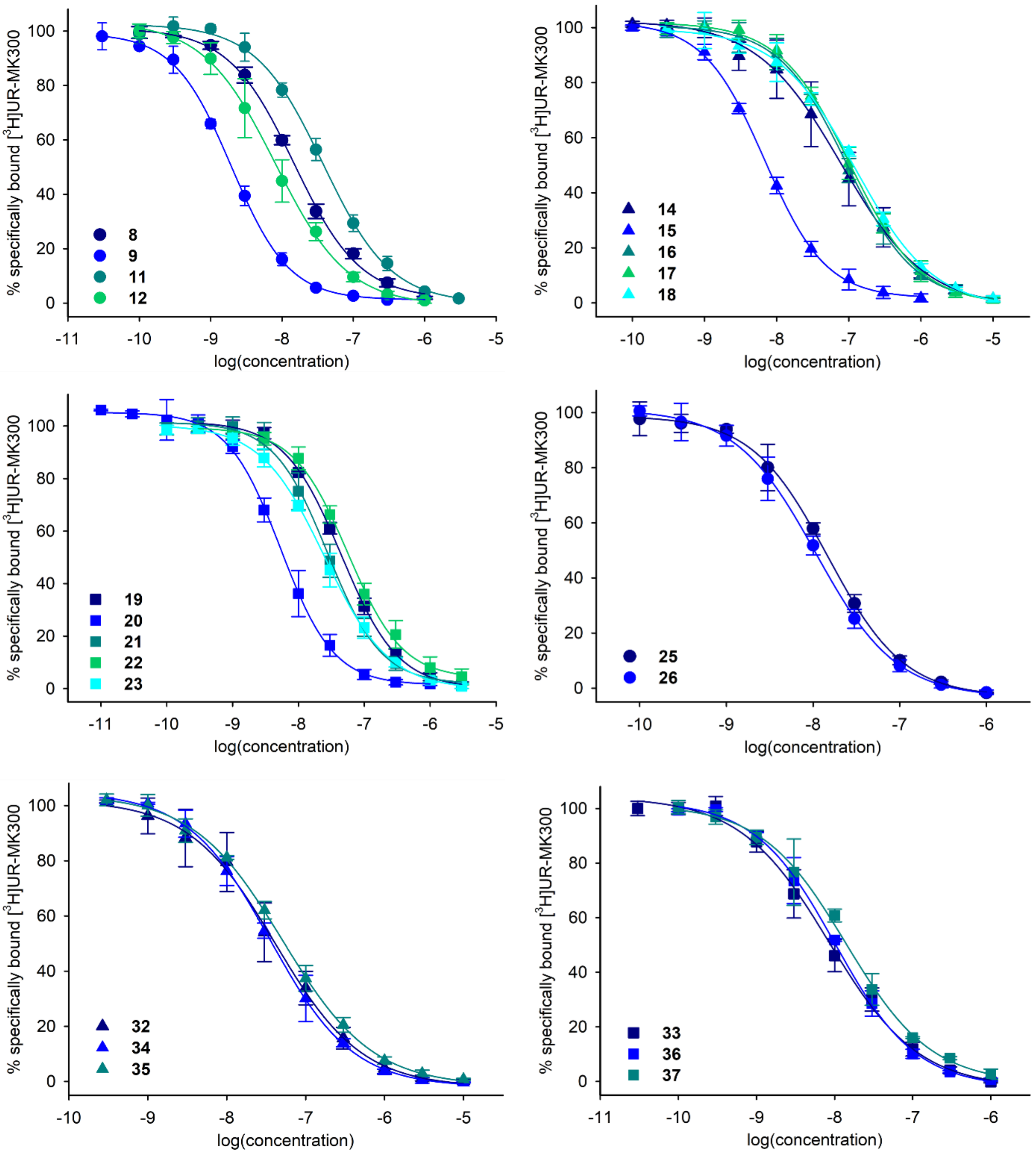

Figure S3. Radioligand displacement curves from competition binding experiments with $[^3H]$UR-MK300 (K_d = 0.55 nM or 0.41 nM, c = 1 nM) and **8**, **9**, **11**, **12**, **14-23**, **25**, **26** and **32-37** at intact $hNTS_1R$-expressing HT-29 cells. Amino-functionalized precursor peptides are represented by circles, DOTA-conjugated peptides are represented by triangles, and Ga^{3+}-containing compounds are represented by squares. Data represent mean values ± SD from at least two independent experiments (performed in triplicate).

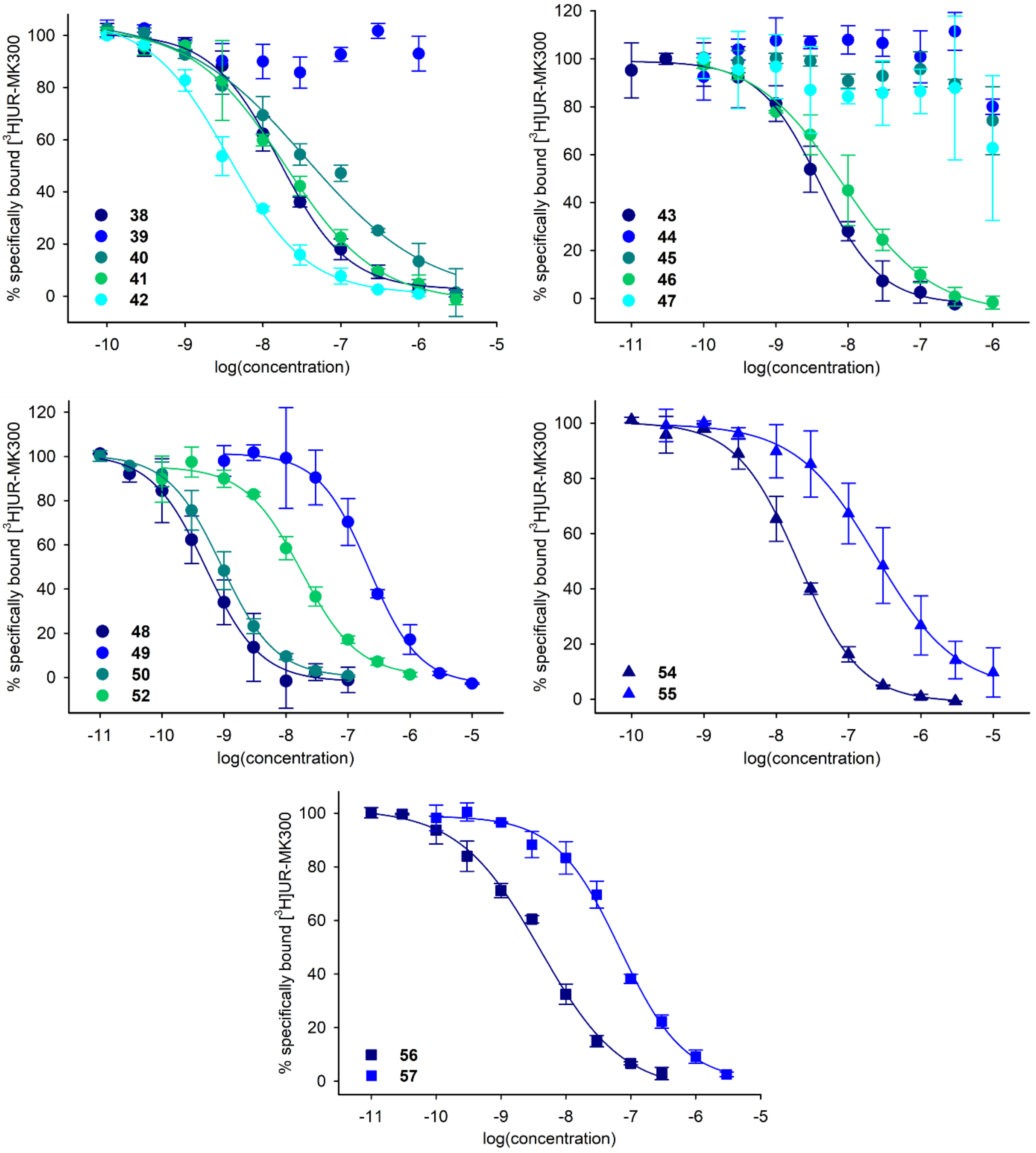

Figure S4. Radioligand displacement curves from competition binding experiments with [^{3}H]UR-MK300 (K_d = 0.41 nM, c = 1 nM) and **38**-**50**, **52** and **54**-**57** at intact hNTS$_1$R-expressing HT-29 cells. Amino-functionalized precursor peptides are represented by circles, DOTA-conjugated peptides are represented by triangles, and Ga^{3+}-containing compounds are represented by squares. Data represent mean values ± SD from at least two independent experiments (performed in triplicate).

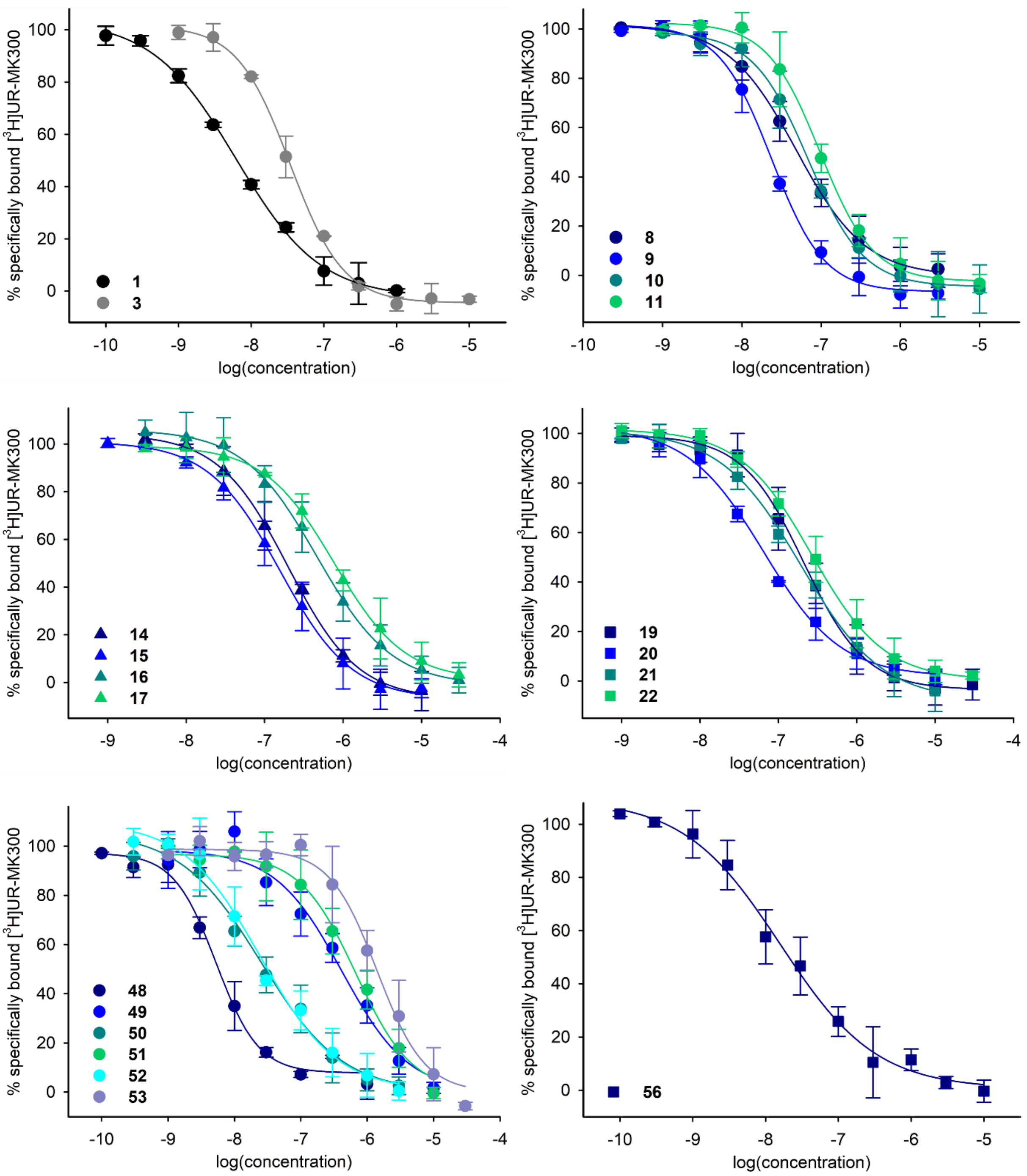

Figure S5. Radioligand displacement curves from competition binding experiments with [^{3}H]UR-MK300 (K_d = 6.9 nM or 4.0 nM, c = 10 nM) and **1**, **3**, **8-11**, **14-17**, **19-22**, **48-53** or **56** at intact HEK293T-hNTS$_2$R cells. Reference compounds and amino-functionalized precursor peptides are represented by circles, DOTA-conjugated peptides are represented by triangles, and Ga^{3+}-containing compounds are represented by squares. Data represent mean values ± SD from at least two independent experiments (performed in triplicate).

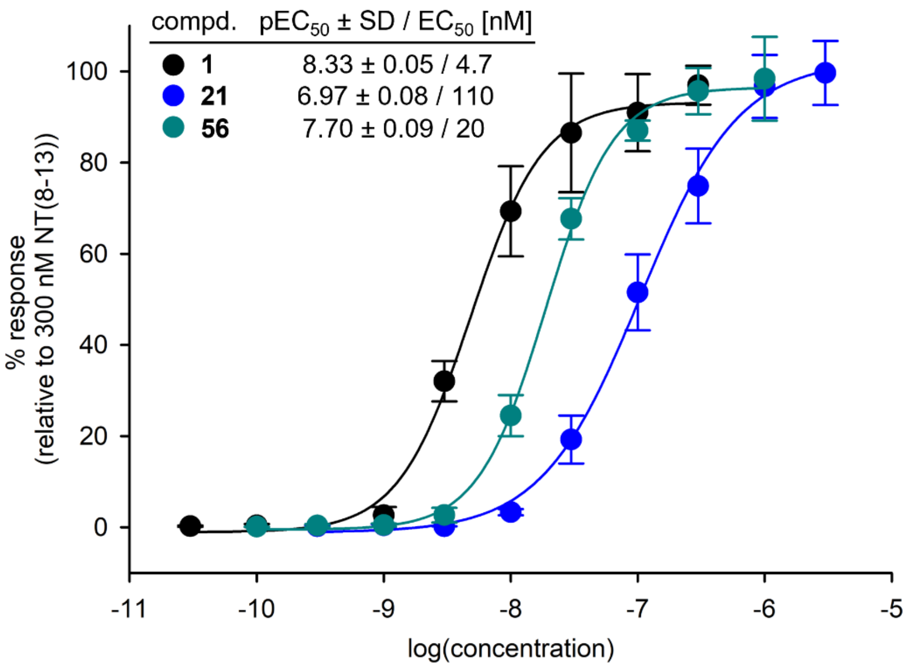

Figure S6. Concentration response curves and agonistic potencies (pEC_{50}, EC_{50}) of **1**, **21** and **56** from fura-2 Ca^{2+}-assays using intact $hNTS_1R$-expressing HT-29 cells. Data represent mean values ± SD from three or four independent experiments (performed in singlet).

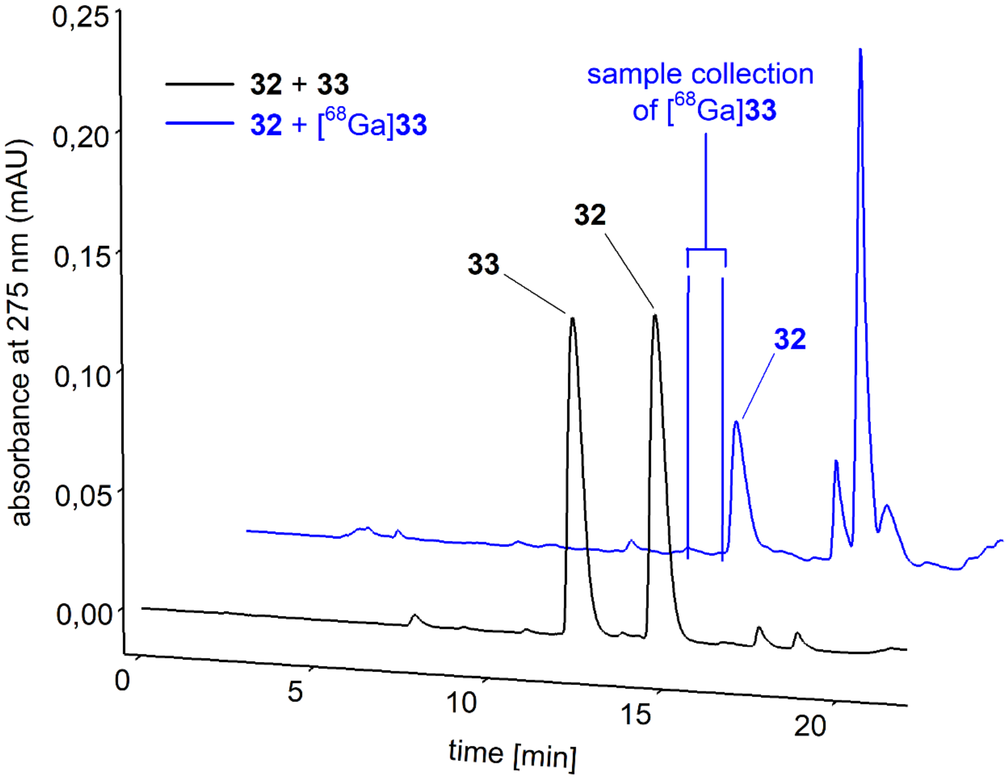

Figure S7. Chromatogram of the RP-HPLC analysis of a mixture of the labeling precursor **32** and the "cold" PET ligand **33** (50 µM each, injection volume 75 µL) (black line), and chromatogram of the preparative HPLC run for the separation of the PET tracer [^{68}Ga]**33** from **32** after radiosynthesis (blue line). The vertical blue lines give the beginning and the end of tracer collection.

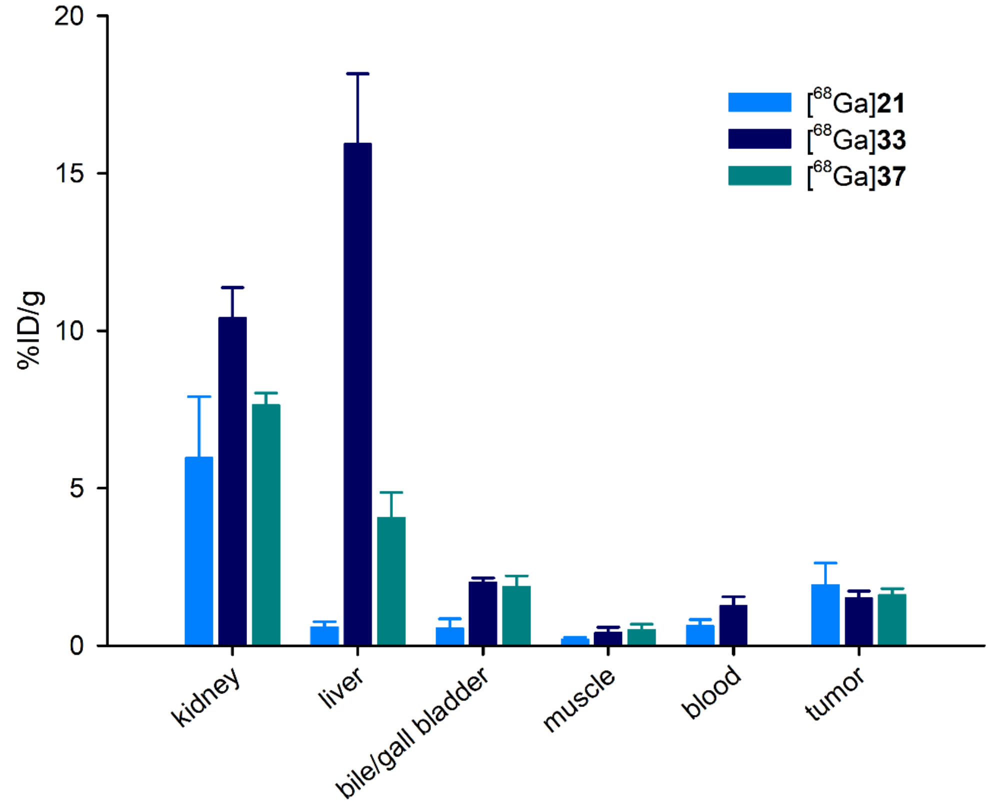

Figure S8. Biodistribution data (%ID/g tissue) of [^{68}Ga]**21**, [^{68}Ga]**33** and [^{68}Ga]**37** from HT-29 tumor bearing mice. Given are mean values ± SD (n = 3 ([^{68}Ga]**33**, [^{68}Ga]**37**) or n = 4 ([^{68}Ga]**21**)) gained at 45 min p.i.

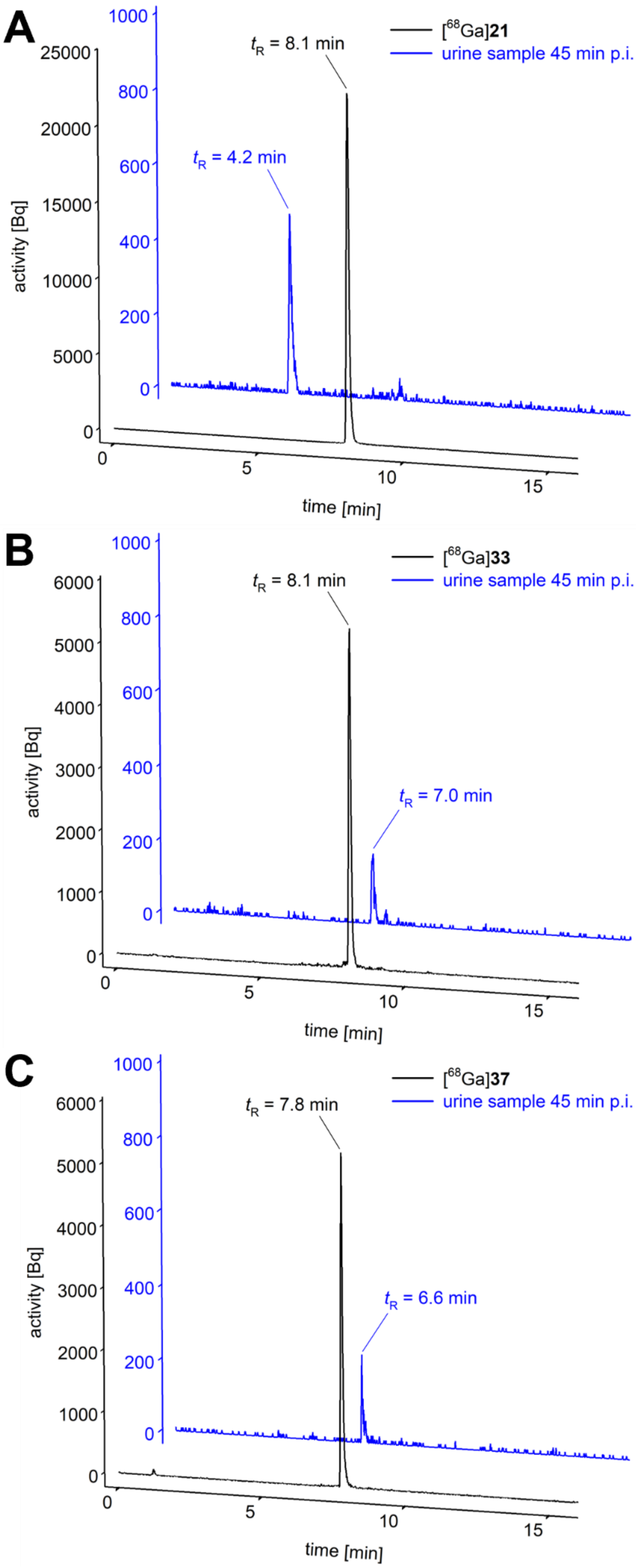

Figure S9. Representative chromatograms of the RP-HPLC quality controls of the PET tracers [^{68}Ga]**21** (A), [^{68}Ga]**33** (B) and [^{68}Ga]**37** (C) after radiosynthesis (black lines) and of the RP-HPLC analyses of urine samples obtained from mice 45 min after injection of the respective PET tracers.

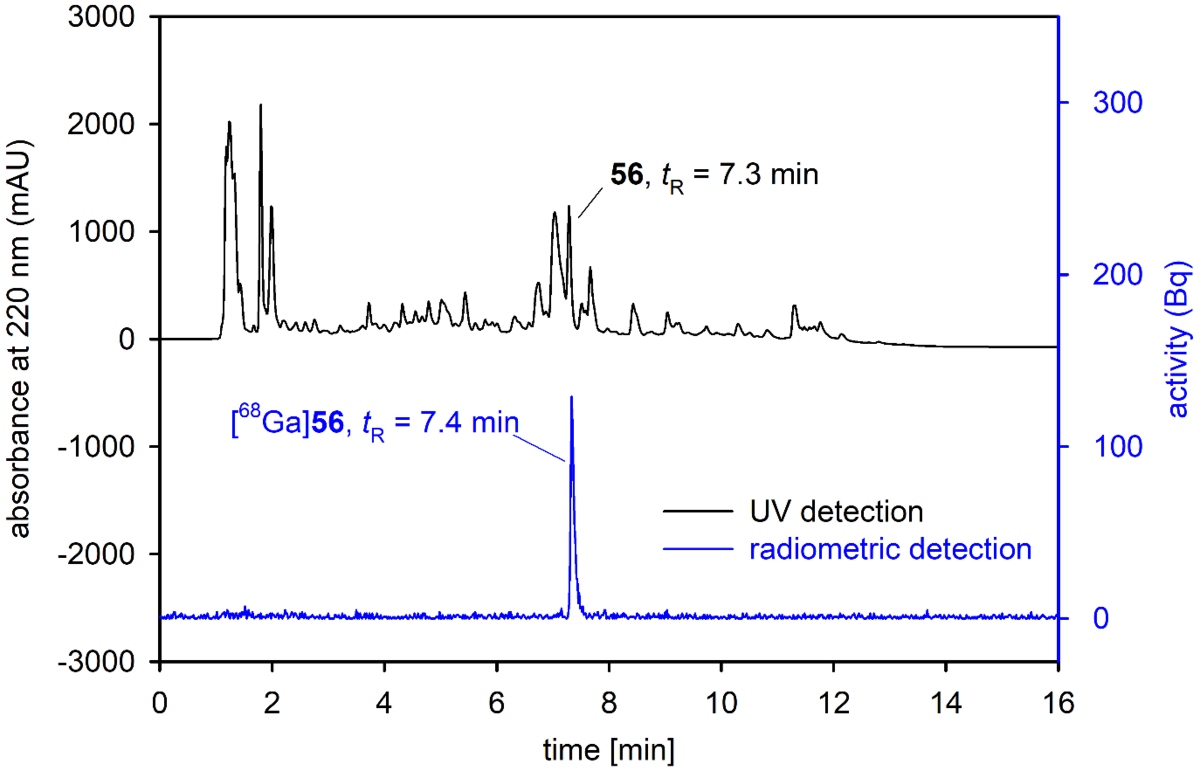

Figure S10. RP-HPLC analysis of an ex vivo urine sample from a mouse 45 min after injection of [^{68}Ga]**56**, spiked with **56** (100 µM). The blue line shows radiodetection and the black line shows UV detection at 220 nm.

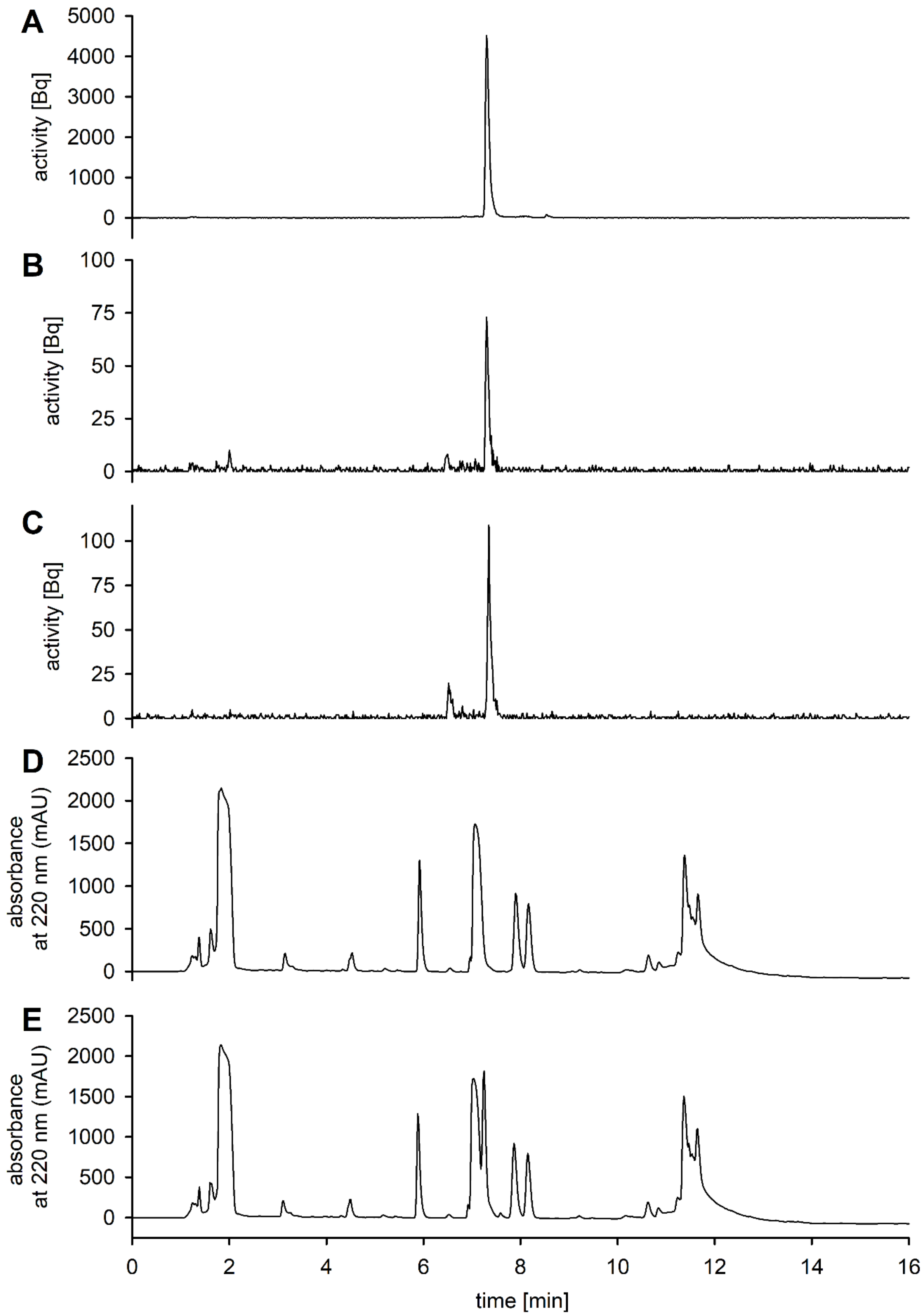

Figure S11. Chromatograms of the RP-HPLC analyses of different samples of [^{68}Ga]**56** using radio- or UV-detection. (A) quality control after radiosynthesis; (B) ex vivo urine sample obtained from a mouse 10 min after injection of [^{68}Ga]**56**; (C) ex vivo plasma sample (10 min p.i.) from the same mouse as under B; (D) UV-detection of the analysis from C; (E) plasma sample from C and D spiked with **56** (100 µM).

Table S1. Equivalents and conditions applied for SPPS.

Fmoc-aa	equiv. Fmoc-aa	equiv. HBTU/HOBt/DIPEA	coupling conditions[a]
Fmoc-Arg(Pbf)-OH	5	4.9/5/10	„double“, 35 °C
Fmoc-Pro-OH	5	4.9/5/10	„double“, 35 °C
Fmoc-Tyr(*t*Bu)-OH	5	4.9/5/10	„double“, 35 °C
Fmoc-Ile-OH	5	4.9/5/10	„double“, 35 °C
Fmoc-Tle-OH (for **8**, **52** and **53**)	4	3.95/4/8	„double“, 35 °C
Fmoc-Tle-OH (for **11**)	5		„double“, 35 °C
Fmoc-Tle-OH (for **12**)	4.4	4.35/4.4/8.8	„double“, 35 °C
Fmoc-N-Me-Arg(Pbf)-OH[b]	3.5	3.45/3.5/7	„double“, 35 °C
Fmoc-L-allo-Ile-OH[b]	5	4.9/5/10	„double“, 35 °C
Fmoc-Deg-OH[b]	5	4.9/5/10	„double“, 35 °C
Fmoc-L-cPrGly-OH[b]	5	4.9/5/10	„double“, 35 °C
Fmoc-*β*-cyclopropyl-L-Ala-OH[b]	5	4.9/5/10	„double“, 35 °C
Fmoc-(*S*)-2-amino-2-cyclobutylacetic acid[b]	5	4.9/5/10	„double“, 35 °C
Fmoc-*β*-cyclopentyl-L-Gly-OH[b]	5	4.9/5/10	„double“, 35 °C
(*S*)-Fmoc-*α*-ethyl-Ala-OH[b]	5	4.9/5/10	„double“, 35 °C
Fmoc-*α*-methyl-L-Leu-OH[b]	3	2.95/3/6	„double“, 35 °C
Fmoc-*β*,*β*-diMe-Tyr(*t*Bu)-OH (rac)[b]	3	2.95/3/6	„double“, 35 °C
**6** (for **8** and **9**)[b]	3	3/3/6	“single”, 14 h, 35 °C
**6** (for **11**)[b]	3		“single”, 14 h, 35 °C
**6** (for **50-53**)[b]	3	3/3/6	„double“, 35 °C
**7**[b]	2.6	2.6/2.6/5.2	“single”, 14 h, 35 °C

[a]In the case of “double coupling”, the length of one coupling step varied between 45 min and overnight.
[b]Anhydrous DMF and NMP was used for the coupling reactions.

Table S2. Recoveries of peptides **8**, **9**, **11**, **12**, **14-23** and **54-57** from human plasma/PBS (1:2 v/v) and ratios of peptide-recovery over recovery of IS.

Compd.	Peptide concentration 80 µM			Peptide concentration 4 µM		
	recovery peptide (%)[a]	recovery IS (%)[a]	ratio[b]	recovery peptide (%)[a]	recovery IS (%)[a]	ratio[b]
**8**	82	98	0.84	103	98	1.05
	84	102	0.83	103	102	1.00
	82	96	0.86	100	100	1.01
				106	102	1.04
			(0.84 ± 0.02)			(1.03 ± 0.02)
**9**	90	103	0.87	92	101	0.90
	85	99	0.86	92	109	0.85
	86	98	0.88	88	100	0.88
	83	97	0.86	96	113	0.85
	85	96	0.89			
			(0.87 ± 0.01)			(0.87 ± 0.03)

Table S2 continued

**11**	90	104	0.87	98	96	1.02
	89	105	0.84	101	103	0.98
	90	106	0.85	101	99	1.02
	91	106	0.86	98	99	1.00
	88	99	0.89	96	96	1.00
			(0.86 ± 0.02)			(1.00 ± 0.02)
**12**	90	95	0.95	109	101	1.07
	99	110	0.90	111	100	1.12
	95	104	0.92	107	97	1.10
	90	97	0.93	109	100	1.10
	89	98	0.91			
			(0.92 ± 0.02)			(1.09 ± 0.02)
**14**	93	101	0.92	96	103	0.93
	79	84	0.94	90	99	0.91
	82	89	0.92	90	98	0.92
				94	105	0.90
			(0.93 ± 0.01)			(0.91 ± 0.01)
**15**	89	96	0.93	73	94	0.77
	90	104	0.87	80	103	0.78
	84	98	0.86	79	105	0.76
	84	99	0.84			
	86	95	0.91			
			(0.88 ± 0.04)			(0.77 ± 0.01)
**16**	93	96	0.97	99	103	0.97
	102	107	0.95	108	110	0.99
	106	109	0.97	99	100	0.98
	99	103	0.96	107	113	0.95
				102	109	0.94
			(0.96 ± 0.01)			(0.97 ± 0.02)
**17**	84	88	0.96	123	88	1.40
	86	87	0.99	129	88	1.46
	85	88	0.96	121	82	1.48
	86	86	1.01	130	90	1.44
	85	89	0.96	124	84	1.47
			(0.97 ± 0.02)			(1.45 ± 0.03)
**18**	108	118	0.92	104	99	1.05
	104	110	0.94	105	103	1.02
	99	107	0.93	114	108	1.06
	105	112	0.95	103	100	1.03
	105	110	0.95			
			(0.94 ± 0.01)			(1.04 ± 0.02)
**19**	91	112	0.81	112	111	1.01
	88	110	0.80	110	102	1.07
	86	108	0.80	112	102	1.10
	86	105	0.82	112	109	1.03
			(0.81 ± 0.01)			(1.05 ± 0.04)
**20**	103	123	0.84	102	103	0.99
	111	134	0.82	98	102	0.96
	99	114	0.87	102	100	1.02
	90	107	0.85	108	108	1.00
	102	119	0.86			
			(0.85 ± 0.02)			(0.99 ± 0.03)

Table S2 continued

**21**	98	108	0.91	115	99	1.16
	92	104	0.89	120	105	1.14
	97	108	0.90	118	105	1.13
	91	100	0.91	120	101	1.19
	89	102	0.88	112	101	1.11
			(0.90 ± 0.02)			(1.15 ± 0.03)
**22**	80	89	0.89	96	83	1.16
	81	91	0.89	90	86	1.06
	76	86	0.88	100	89	1.13
	77	84	0.91	100	90	1.11
			(0.89 ± 0.01)			(1.11 ± 0.04)
**23**	88	100	0.87	112	111	1.01
	96	105	0.91	98	96	1.02
	97	109	0.89	107	105	1.02
	104	119	0.88	106	103	1.03
	98	109	0.90			
			(0.89 ± 0.02)			(1.02 ± 0.01)
**54**	90	97	0.94	96	96	1.00
	90	96	0.94	100	99	1.01
	94	97	0.98	106	99	1.07
	89	93	0.95	107	109	0.98
	91	99	0.92	103	95	1.09
			(0.94 ± 0.02)			(1.03 ± 0.05)
**55**	91	96	0.95	97	96	1.01
	102	105	0.97	99	101	0.99
	88	91	0.96	102	106	0.96
	89	94	0.95	94	94	0.99
				103	100	1.02
			(0.96 ± 0.01)			(0.99 ± 0.02)
**56**	94	103	0.92	99	100	0.99
	84	94	0.89	100	93	1.07
	91	101	0.91	101	97	1.04
	87	96	0.91	109	107	1.01
	90	99	0.91	105	107	0.98
			(0.91 ± 0.01)			(1.02 ± 0.04)
**57**	85	97	0.88	104	105	0.99
	83	91	0.91	96	95	1.01
	90	98	0.92	100	102	0.98
	88	92	0.96	94	95	0.99
	100	106	0.94	91	90	1.01
			(0.92 ± 0.03)			(1.00 ± 0.02)

[a]Recoveries of the peptides and of IS from human plasma/PBS (1:2 v/v) using a peptide concentration of 80 µM or 4 µM and an IS concentration of 10 µM (three, four or five independent experiments). [b]Ratios of peptide recovery over recovery of the IS calculated for individual experiments, as well as mean recovery ratios ± SD (given in parenthesis). Note: When the remaining intact peptide concentration in plasma was > 20 µM, recovery ratios based on the 80 µM peptide concentrations were used to calculate peptide recoveries of the plasma stability samples. When the remaining intact peptide concentration was < 20 µM, recovery ratios based on the 4 µM peptide concentrations were used to calculate peptide recoveries of the plasma stability samples.

Table S3. NTS_1R affinities of **8-12**, **14-18**, **25**, **26**, **32**, **34**, **35**, **38-50**, **52**, **54** and **55**, NTS_2R affinities of **8-11**, **14-17** and **48-53**, NTS_1R selectivities of **8-11**, **14-17**, **48-50** and **52**, and in vitro plasma stabilities of **8-12**, **14-18**, **38-49**, **54** and **55**, determined at 37 °C.

cpd.	pK_i ± SD /	pK_i ± SD /	NTS_1R selectivity	% intact peptide in plasma after the

	K_i [nM] NTS$_1$R[a]	K_i [nM] NTS$_2$R[b]	(ratio K_i (NTS$_2$R) / K_i (NTS$_1$R))	given incubation time[c]			
				1 h	6 h	24 h	48 h
**8**	8.28 ± 0.05 / 5.3	7.68 ± 0.11 / 22	4.2	> 99	> 99	> 99	> 99
**9**	9.16 ± 0.02 / 0.69	8.10 ± 0.19 / 8.3	12	99 ± 2	57 ± 2	3.0 ± 0.1	1.4 ± 0.3
**10**	8.55 / 2.8[d]	7.72 ± 0.09 / 19	6.8	> 99[d]	> 99[d]	> 99[d]	> 99[d]
**11**	7.88 ± 0.04 / 13	7.57 ± 0.10 / 27	2.1	> 99	> 99	> 99	> 99
**12**	8.61 ± 0.23 / 2.7	n.d.	-	97 ± 3	97 ± 3	94 ± 3	91 ± 3
**14**	7.66 ± 0.39 / 28	7.11 ± 0.07 / 79	2.8	> 99	> 99	> 99	> 99
**15**	8.62 ± 0.005 / 2.4	7.27 ± 0.10 / 55	23	78 ± 1	8.3 ± 0.4	< 1	< 1
**16**	7.48 ± 0.02 / 33	6.86 ± 0.25 / 150	4.5	> 99	> 99	93 ± 3	84 ± 2
**17**	7.45 ± 0.09 / 36	6.65 ± 0.10 / 230	6.4	97 ± 1	99 ± 1	98 ± 1	97 ± 1
**18**	7.45 ± 0.07 / 36	n.d.	-	> 99	99 ± 1	94 ± 1	86 ± 1
**25**	8.37 ± 0.08 / 4.3	n.d.	-	n.d.	n.d.	n.d.	n.d.
**26**	8.52 ± 0.11 / 3.1	n.d.	-	n.d.	n.d.	n.d.	n.d.
**32**	7.97 ± 0.19 / 12	n.d.	-	n.d.	n.d.	n.d.	n.d.
**34**	7.99 ± 0.06 / 10	n.d.	-	n.d.	n.d.	n.d.	n.d.
**35**	7.87 ± 0.12 / 14	n.d.	-	n.d.	n.d.	n.d.	n.d.
**38**	8.33 ± 0.06 / 4.8	n.d.	-	92 ± 1	87 ± 1	70 ± 1	52 ± 1
**39**	< 6 / > 1000	n.d.	-	93 ± 1	94 ± 1	93 ± 1	93 ± 1
**40**	8.05 ± 0.21 / 9.6	n.d.	-	88 ± 1	58 ± 1	< 1	< 1
**41**	8.31 ± 0.12 / 5.0	n.d.	-	92 ± 1	74 ± 1	15 ± 1	< 1
**42**	8.98 ± 0.05 / 1.1	n.d.	-	91 ± 1	79 ± 1	32 ± 1	< 1
**43**	8.92 ± 0.09 / 1.2	n.d.	-	99 ± 1	95 ± 1	78 ± 1	58 ± 1
**44**	< 6 / > 1000	n.d.	-	94 ± 1	91 ± 4	91 ± 1	87 ± 1
**45**	< 6 / > 1000	n.d.	-	93 ± 1	92 ± 1	86 ± 1	77 ± 1
**46**	8.63 ± 0.22 / 2.6	n.d.	-	25 ± 2	< 1	< 1	< 1
**47**	< 6 / > 1000	n.d.	-	89 ± 1	88 ± 1	81 ± 1	73 ± 1
**48**	9.86 ± 0.17 / 0.14	8.82 ± 0.07 / 1.5	11	90 ± 4	97 ± 6	92 ± 1	92 ± 1
**49**	7.25 ± 0.06 / 56	6.95 ± 0.12 / 110	2.0	96 ± 2	96 ± 1	94 ± 5	95 ± 2
**50**	9.60 ± 0.12 / 0.26	8.23 ± 0.28 / 6.8	26	n.d.	n.d.	n.d.	n.d.
**51**	n.d.	6.76 ± 0.07 / 170	-	n.d.	n.d.	n.d.	n.d.
**52**	8.30 ± 0.21 / 5.4	8.22 ± 0.22 / 6.5	1.2	n.d.	n.d.	n.d.	n.d.
**53**	n.d.	6.40 ± 0.24 / 450	-	n.d.	n.d.	n.d.	n.d.
**54**	8.23 ± 0.08 / 5.9	n.d.	-	> 99	> 99	> 99	> 99
**55**	7.10 ± 0.20 / 85	n.d.	-	> 99	> 99	> 99	> 99

[a]Determined by radioligand competition binding with [^{3}H]UR-MK300 at HT-29 cells (K_d = 0.55 nM [2] or 0.41 nM, c = 1 nM); given are mean values ± SD (pK_i) and mean values (K_i) from two (**8**, **15**, **42**), three (**9**, **11**, **16**, **17**, **25**, **32**, **34**, **38**, **40**, **41**, **43**, **46**, **48-50**, **52**, **54**, **55**), four (**12**, **18**, **26**, **35**) or five (**14**) independent experiments, each performed in triplicate. [b]Determined by radioligand competition binding with [^{3}H]UR-MK300 at HEK293T-hNTS$_2$R cells (K_d = 6.9 nM or 4.0 nM, c = 10 nM); given are mean values ± SD (pK_i) and mean values (K_i) from two (**9**, **14**), three (**8**, **10**, **11**, **16**, **17**, **48**, **49**, **51**, **52**) or four (**15**, **50**, **53**) independent experiments, each performed in triplicate. [c]The initial concentration of the peptide in human plasma/PBS (1:2 v/v) was 100 µM. Data represent means ± SD from two or three independent experiments (SD not given when no decomposition was observed). [d]Schindler et al. [3]

Table S4. *Ex vivo* biodistribution data and tumor-to-muscle ratios of [^{68}Ga]**21**, [^{68}Ga]**33** and [^{68}Ga]**37**.[a]

tissue	uptake (%ID/g) 45 min p.i.		
	[^{68}Ga]**21**	[^{68}Ga]**33**	[^{68}Ga]**37**
kidney	6.0 ± 1.9	10 ± 0.96	7.6 ± 0.39

liver	0.59 ± 0.17	16 ± 2.2	4.1 ± 0.79
gall bladder (bile)	0.55 ± 0.29	2.0 ± 0.14	1.9 ± 0.34
muscle	0.21 ± 0.062	0.41 ± 0.17	0.50 ± 0.18
blood	0.63 ± 0.20	1.3 ± 0.29	n.d.
tumor	1.9 ± 0.70	1.5 ± 0.22	1.6 ± 0.19
tumor-to-muscle	9.5 ± 3.7	4.1 ± 1.7	3.4 ± 0.71

[a]Given are mean values ± SD (n = 3 ([^{68}Ga]**33**, [^{68}Ga]**37**) or n = 4 ([^{68}Ga]**21**)). Organ uptake values were obtained at 45 min p.i. from HT-29 tumor bearing mice.

2. Synthesis protocols and analytical data of compounds 8, 9, 11, 12, 14-23, 25, 26, 28, 29 and 31-57.

General procedure for manual solid-phase peptide synthesis (SPPS). The synthesis was performed according to a reported procedure [1] with minor modifications. The resin was allowed to swell in the solvent for 45 min before the beginning of the synthesis. For coupling conditions see Table S1.

General procedure for the conjugation of the DOTA chelator to peptides. The reaction was performed in a 2-mL reaction vessel with screw cap, equipped with a magnetic micro stirrer. DIPEA (27 equiv. (**14-17**), 15 equiv. (**34**, **35**), 13 equiv. (**18**, **32**) or 12 equiv. (**54**, **55**)) was added to a solution of the peptide (2.2 equiv. (**14-17**), 1.25 equiv. (**34**, **35**), 1.1 equiv. (**18**, **32**) or 1 equiv. (**54**, **55**)) in DMF/NMP (75:25 v/v) or DMF/NMP (80:20 v/v) (26-230 µL), followed by the addition of DOTA tris(*tert*-butyl) succinimidyl ester (**13**, 1 equiv.) dissolved in anhydrous DMF or DMF/NMP (80:20 v/v) (6-30 µL). After stirring at rt for 30 min, 10% aq TFA (corresponding to 18 equiv. TFA (**14-17**), 10 equiv. TFA (**34**, **35**), 9 equiv. TFA (**18**, **35**) or 8 equiv. TFA (**54**, **55**)) was added. The protected intermediate was isolated by preparative HPLC. After lyophilization of the eluate, TFA/H_2O (80:20 v/v) (0.5-3 mL) was added, and the mixture was stirred at 50 °C overnight. The crude product was taken up in H_2O (25-100 mL), and the solution was subjected to lyophilization. The DOTA-conjugated peptide was purified by preparative HPLC.

General procedure for the incorporation of Ga^{3+} into DOTA-conjugated peptides. The incorporation reaction was performed in a 2-mL reaction vessel with screw cap. A solution of the peptide (4 mM) in HEPES buffer (0.2 M, pH 4.2) was heated to 60 °C for 5 min, followed by the addition of a solution of $Ga(NO_3)_3 \times H_2O$ (3 equiv., 0.4 M) in aqueous HCl (10 mM). The mixture was shaken at 100 °C for 10 min (**19-23**) or 30 min (**33**, **36**, **37**, **56**, **57**) using a Thermocell mixing block from Bioer (Hangzhou, China), and the product was purified by preparative HPLC.

***N*$^{\alpha}$-(*N*$^{\alpha}$-Methylarginyl)-*N*$^{\omega}$-[(4-aminobutyl)aminocarbonyl]Arg-Pro-Tyr-*α*-*tert*-butyl-Gly-Leu tetrakis(hydrotrifluoroacetate) (8).**

Peptide **8** was synthesized according to the general procedure for SPPS using a H-Leu-2-ClTrt resin (loading 0.79 mmol/g) (109 mg, 0.086 mmol). Purification by preparative RP-HPLC (column: Kinetex-XB C18, gradient: 0-35 min: A1/B1 92:8-57:43, t_R = 18 min) afforded **8** as white fluffy solid (68.7 mg, 57%). ^{1}H-NMR (600 MHz, DMSO-d_6): δ 0.80-0.95 (m, 15H), 1.39-1.63 (m, 12H), 1.63-1.90 (m, 6H), 1.92-2.06 (m, 1H), 2.46-2.48 (m, 3H), 2.64-2.72 (m, 1H), 2.76-2.81 (m, 2H), 2.85-2.92 (m, 1H), 3.05-3.12 (m, 4H), 3.22-3.27 (m, 2H), 3.55-3.59 (m, 2H), 3.75-3.80 (m, 1H), 4.17-4.24 (m, 1H), 4.27-4.31 (m, 1H), 4.31-4.39 (m, 1H), 4.44-4.59 (m, 2H), 6.55-6.64 (m, 2H), 6.64-7.15 (br s, 2H, interfering with the next listed signal), 6.97-7.01 (m, 2H), 7.15-7.53 (br s, 2H), 7.53-7.66 (m, 2H), 7.66-7.90 (m, 4H), 7.97 (d, 1H, *J* 7.4 Hz), 8.17-8.27 (m, 1H), 8.31-8.67 (m, 2H), 8.68-9.12 (m, 4H), 9.12-9.25 (m, 1H), 10.27-10.56 (m, 1H), 12.49 (br s, 1H). HRMS: *m/z* $[M+2H]^{2+}$ calcd. for $[C_{44}H_{78}N_{14}O_9]^{2+}$ 473.3033, found 473.3039. RP-HPLC (220 nm): > 99% (t_R = 5.8 min, k = 6.6). $C_{44}H_{76}N_{14}O_9 \cdot C_8H_4F_{12}O_8$ (945.18 + 456.09).

***N*$^{\alpha}$-(*N*$^{\alpha}$-Methylarginyl)-*N*$^{\omega}$-[(4-aminobutyl)aminocarbonyl]Arg-Pro-Tyr-Ile-Leu tetrakis(hydrotrifluoroacetate) (9).**

Peptide **9** was synthesized according to the general procedure for SPPS using a H-Leu-2-ClTrt resin (loading 0.79 mmol/g) (120 mg, 0.095 mmol). Purification by preparative RP-HPLC (column: Kinetex-XB C18, gradient: 0-35 min: A1/B1 92:8-57:43, t_R = 18 min) afforded **9** as white fluffy solid (58.8 mg, 44%). ^{1}H-NMR (600 MHz, DMSO-d_6): δ 0.76-0.87 (m, 9H), 0.87-0.92 (m, 3H), 0.99-1.10 (m, 1H), 1.40-1.64 (m, 13H), 1.64-1.89 (m, 7H), 1.94-2.05 (m, 1H), 2.45-2.48 (m, 3H), 2.64-2.72 (m, 1H), 2.76-2.82 (m, 2H), 2.83-2.89 (m, 1H), 3.07-3.13 (m, 4H), 3.22-3.28 (m, 2H), 3.58-3.62 (m, 2H), 3.76-3.80 (m, 1H), 4.17-4.26 (m, 2H), 4.28-4.39 (m, 1H), 4.39-4.50 (m, 1H), 4.50-4.61 (m, 1H), 6.53-6.65 (m, 2H), 6.65-7.13 (br s, 2H, interfering with the next listed signal), 6.98-7.00 (m, 2H), 7.13-7.48 (br s, 2H), 7.48-7.61 (m, 1H), 7.61-7.82 (m, 5H), 7.82-8.00 (m, 1H), 8.19 (d, 1H, *J* 7.7 Hz), 8.26-8.70 (m, 2H), 8.70-9.10 (m, 4H), 9.10-9.25 (m, 1H), 10.42 (s, 1H), 12.50 (br s, 1H). HRMS: *m/z* $[M+2H]^{2+}$ calcd. for $[C_{44}H_{78}N_{14}O_9]^{2+}$ 473.3033, found 473.3047. RP-HPLC (220 nm): > 99% (t_R = 6.0 min, k = 6.9). $C_{44}H_{76}N_{14}O_9 \cdot C_8H_4F_{12}O_8$ (945.18 + 456.09).

***N*$^{\alpha}$-Arginyl-*N*$^{\alpha}$-methyl-*N*$^{\omega}$-[(4-aminobutyl)aminocarbonyl]Arg-Pro-Tyr-*α*-*tert*-butyl-Gly-Leu tetrakis(hydrotrifluoroacetate)** (**11**).

Peptide **11** was synthesized according to the general procedure for SPPS using a H-Leu-2-ClTrt resin (loading 0.79 mmol/g) (60 mg, 0.047 mmol), with the following modification: Fmoc amino acids (Fmoc-Arg(Pbf)-OH, Fmoc-Pro-OH, Fmoc-Tle-OH, Fmoc-Tyr(tBu)-OH (used in 5-fold excess) and **6** (used in 3-fold excess)) were preactivated with Oxyma/DIC (5/5 equiv. and 3/3 equiv., respectively) instead of HBTU/HOBt. After coupling of arginine building block **6** and Fmoc-deprotection, the resin was washed with CH_2Cl_2 (5 ×), a solution of 2-nitrobenzenesulfonylchloride (31.5 mg, 0.142 mmol) and collidine (31.4 µL, 0.237 mmol) in CH_2Cl_2 (1.25 mL) was added and the mixture was shaken at rt for 2 h. The resin was washed with DMF (5 ×), and a solution of MTBD (27.2 µL, 0.190 mmol) and methyl-4-nitrobenzenesulfonate (51.5 mg, 0.237 mmol) in DMF (1.5 mL) was added. After shaking at rt for 30 min, the resin was washed with DMF (3 ×) followed by the addition of a solution of DBU (35.4 µL, 0.237 mmol) and 2-mercaptoethanol (33.1 µL, 0.474 mmol) in DMF (1.25 mL) and shaking at rt for 30 min. The resin was washed with DMF (5 ×) followed by coupling of Fmoc-Arg(Pbf)-OH as described above. Fmoc-deprotection and cleavage from the resin was performed as described in the general procedure for SPPS. Purification by preparative RP-HPLC (column: Gemini NX-C18, gradient: 0-35 min: A1/B1 92:8-57:43, t_R = 16 min) afforded **11** as white fluffy solid (39.0 mg, 59%). ^{1}H-NMR (600 MHz, DMSO-d_6): δ 0.79-0.95 (m, 15H), 1.42-1.80 (m, 18H), 1.92-2.03 (m, 1H), 2.66-2.72 (m, 1H), 2.76-2.81 (m, 2H), 2.84-2.95 (m, 4H), 3.06-3.14 (m, 4H), 3.22-3.28 (m, 3H), 3.50-3.51 (m, 1H), 4.17-4.23 (m, 1H), 4.25-4.32 (m, 2H), 4.32-4.40 (m, 1H), 4.41-4.47 (m, 1H), 5.12-5.18 (m, 1H), 6.59-6.64 (m, 2H), 6.64-7.19 (br s, 2H, interfering with the next listed signal), 6.98-7.01 (m, 2H), 7.19-7.52 (br s, 2H), 7.52-7.64

(m, 2H), 7.64-7.82 (m, 4H), 7.99 (d, 1H, *J* 7.9 Hz), 8.05-8.30 (m, 4H), 8.35-8.61 (m, 2H), 8.88-9.10 (m, 1H), 9.10-9.25 (m, 1H), 10.21-10.55 (m, 1H), 12.47 (br s, 1H). HRMS: *m/z* $[M+3H]^{3+}$ calcd. for $[C_{44}H_{79}N_{14}O_9]^{3+}$ 315.8713, found 315.8722. RP-HPLC (220 nm): 99% (t_R = 5.7 min, *k* = 6.5). $C_{44}H_{76}N_{14}O_9 \cdot C_8H_4F_{12}O_8$ (945.18 + 456.09).

N^α-Methyl-N^ω-[(8-amino-3,6-dioxaoctyl)aminocarbonyl]Arg-Arg-Pro-Tyr-*α*-*tert*-butyl-Gly-Leu tetrakis(hydrotrifluoroacetate) (12).

Peptide **12** was synthesized according to the general procedure for SPPS using a H-Leu-2-ClTrt resin (loading 0.79 mmol/g) (100 mg, 0.079 mmol), with the following modification: after coupling of arginine building block **7** and Fmoc-deprotection, the resin was washed with CH_2Cl_2 (5 ×), a solution of 2-nitrobenzenesul-fonylchloride (52.5 mg, 0.237 mmol) and collidine (52.4 µL, 0.395 mmol) in CH_2Cl_2 (1.5 mL) was added and the mixture was shaken at rt for 2 h. The resin was washed with DMF (5 ×), and a solution of MTBD (45.4 µL, 0.316 mmol) and methyl-4-nitrobenzenesulfonate (85.8 mg, 0.395 mmol) in DMF (1.8 mL) was added. After shaking at rt for 30 min, the resin was washed with DMF (3 ×) followed by the addition of a solution of DBU (59.0 µL, 0.395 mmol) and 2-mercaptoethanol (55.1 µL, 0.790 mmol) in DMF (1.5 mL) and shaking at rt for 30 min. The resin was washed with DMF (5 ×) followed by cleavage from the resin as described in the general procedure for SPPS. Purification by preparative RP-HPLC (column: Gemini-NX C18, gradient: 0-30 min: A1/B1 92:8-60:40, t_R = 13 min) afforded **12** as white fluffy solid (63.1 mg, 55%). ^{1}H-NMR (600 MHz, DMSO-d_6): δ 0.79-0.95 (m, 15H), 1.43-1.88 (m, 14H), 1.94-2.04 (m, 1H), 2.44-2.48 (m, 3H), 2.64-2.71 (m, 1H), 2.85-2.93 (m, 1H), 2.93-3.02 (m, 2H), 3.05-3.15 (m, 2H), 3.24-3.28 (m, 4H), 3.45-3.48 (m, 2H), 3.50-3.64 (m, 8H), 3.72-3.88 (m, 1H), 4.17-4.26 (m, 1H), 4.26-4.31 (m, 1H), 4.31-4.39 (m, 1H), 4.42-4.61 (m, 2H), 6.55-6.62 (m, 2H), 6.62-7.12 (br s, 2H, interfering with the next listed signal), 6.97-7.01 (m, 2H), 7.12-7.44 (br s, 2H), 7.44-7.56 (m, 1H), 7.56-7.67 (m, 2H), 7.67-7.92 (m, 3H), 7.97 (d, 1H, *J* 7.7 Hz), 8.22 (d, 1H, *J* 7.5 Hz), 8.27-8.66 (m, 2H), 8.66-9.32 (m, 5H), 10.20-10.54 (m, 1H), 12.48 (br s, 1H). HRMS: *m/z* $[M+3H]^{3+}$ calcd. for $[C_{46}H_{83}N_{14}O_{11}]^{3+}$ 335.8783, found 335.8791. RP-HPLC (220 nm): 89% (t_R = 6.2 min, *k* = 7.2). $C_{46}H_{80}N_{14}O_{11} \cdot C_8H_4F_{12}O_8$ (1005.23 + 456.09).

N^α-(N^α-Methylarginyl)-N^ω-{[4-(N-{2-[4,7,10-tris(carboxymethyl)-1,4,7,10-tetraazacyclododecan-1-yl]acetyl})aminobutyl]aminocarbonyl}Arg-Pro-Tyr-*α*-*tert*-butyl-Gly-Leu tetrakis(hydrotrifluoroace-tate) (14).

Compound **14** was prepared from **8** (34.2 mg, 24.4 µmol) and **13** (9.0 mg, 11.0 µmol) according to the general procedure for DOTA-conjugation. Isolation of the protected intermediate: column: Kinetex-XB C18, gradient: 0-18 min: A1/B1 92:8-75:25, 18-40 min: 75:25-38:62, t_R = 28 min. Purification of the product by preparative RP-HPLC (column: Kinetex-XB C18, gradient: 0-35 min: A1/B1 92:8-57:43, t_R = 18 min) afforded **14** as

white fluffy solid (14.1 mg, 72%). ^{1}H-NMR (600 MHz, DMSO-d_6): δ 0.81-0.85 (m, 3H), 0.87-0.95 (m, 12H), 1.40-1.64 (m, 12H), 1.64-1.89 (m, 6H), 1.95-2.06 (m, 1H), 2.46-2.48 (m, 3H), 2.65-2.71 (m, 1H), 2.85-2.90 (m, 1H), 3.00-3.27 (m, 25H), 3.53-3.84 (m, 10H), 4.18-4.24 (m, 1H), 4.27-4.31 (m, 1H), 4.31-4.39 (m, 1H), 4.45-4.61 (m, 2H), 6.56-6.64 (m, 2H), 6.64-7.09 (br s, 2H, interfering with the next listed signal), 6.97-7.00 (m, 2H), 7.09-7.56 (br s, 3H), 7.60-7.83 (m, 2H), 7.92-8.00 (m, 1H), 8.03-8.63 (m, 4H), 8.68-9.11 (m, 3H), 9.11-9.51 (m, 2H), 11.83-12.94 (m, 2H). 4 exchangeable protons (NH, OH) of the presumably 4-fold protonated molecule could not be identified. HRMS: *m/z* [*M*+2H]$^{2+}$ calcd. for $[C_{60}H_{104}N_{18}O_{16}]^{2+}$ 666.3933, found 666.3943. RP-HPLC (220 nm): > 99% (t_R = 6.1 min, *k* = 7.0). $C_{60}H_{102}N_{18}O_{16}$ · $C_8H_4F_{12}O_8$ (1331.59 + 456.09).

***N*$^\alpha$-(*N*$^\alpha$-Methylarginyl)-*N*$^\omega$-{[4-(N-{2-[4,7,10-tris(carboxymethyl)-1,4,7,10-tetraazacyclododecan-1-yl]acetyl})aminobutyl]aminocarbonyl}Arg-Pro-Tyr-Ile-Leu tetrakis(hydrotrifluoroacetate) (15).**
Compound **15** was prepared from **9** (32.8 mg, 23.4 μmol) and **13** (8.6 mg, 10.5 μmol) according to the general procedure for DOTA-conjugation. Isolation of the protected intermediate: column: Kinetex-XB C18, gradient: 0-18 min: A1/B1 92:8-75:25, 18-40 min: 75:25-38:62, t_R = 28 min. Purification of the product by preparative RP-HPLC (column: Kinetex-XB C18, gradient: 0-35 min: A1/B1 92:8-57:43, t_R = 18 min) afforded **15** as white fluffy solid (11.7 mg, 62%). ^{1}H-NMR (600 MHz, DMSO-d_6): δ 0.76-0.87 (m, 9H), 0.87-0.93 (m, 3H), 0.98-1.10 (m, 1H), 1.36-1.65 (m, 13H), 1.65-1.90 (m, 7H), 1.93-2.07 (m, 1H), 2.46-2.48 (m, 3H), 2.64-2.72 (m, 1H), 2.84-2.89 (m, 1H), 2.95-3.28 (m, 25H), 3.53-3.86 (m, 10H), 4.17-4.25 (m, 2H), 4.28-4.37 (m, 1H), 4.39-4.50 (m, 1H), 4.50-4.62 (m, 1H), 6.55-6.69 (m, 2H), 6.69-7.13 (br s, 2H, interfering with the next listed signal), 6.97-7.00 (m, 2H), 7.13-7.67 (br s, 3H), 7.67-8.06 (m, 3H), 8.06-8.49 (m, 3H), 8.49-9.63 (m, 6H), 11.80-12.93 (m, 2H). 4 exchangeable protons (NH, OH) of the presumably 4-fold protonated molecule could not be identified. HRMS: *m/z* [*M*+2H]$^{2+}$ calcd. for $[C_{60}H_{104}N_{18}O_{16}]^{2+}$ 666.3933, found 666.3946. RP-HPLC (220 nm): > 99% (t_R = 6.3 min, *k* = 7.3). $C_{60}H_{102}N_{18}O_{16}$ · $C_8H_4F_{12}O_8$ (1331.59 + 456.09).

***N*$^\alpha$-Methyl-*N*$^\omega$-{[4-(N-{2-[4,7,10-tris(carboxymethyl)-1,4,7,10-tetraazacyclododecan-1-yl]acetyl})aminobutyl]aminocarbonyl}Arg-Arg-Pro-Tyr-*α*-*tert*-butyl-Gly-Leu tetrakis(hydrotrifluoroacetate) (16).**
Compound **16** was prepared from **10** (34.2 mg, 24.4 μmol) and **13** (9.0 mg, 11.0 μmol) according to the general procedure for DOTA-conjugation. Isolation of the protected intermediate: column: Kinetex-XB C18, gradient: 0-18 min: A1/B1 92:8-75:25, 18-40 min: 75:25-38:62, t_R = 28 min. Purification of the product by preparative RP-HPLC (column: Kinetex-XB C18, gradient: 0-35 min: A1/B1 92:8-57:43, t_R = 19 min) afforded **16** as white fluffy solid (14.7 mg, 75%). ^{1}H-NMR (600 MHz, DMSO-d_6): δ 0.81-0.94 (m, 15H), 1.40-1.87 (m, 18H), 1.96-2.04 (m, 1H), 2.46-2.49 (m, 3H), 2.65-2.71 (m, 1H), 2.86-2.92 (m, 1H), 3.04-3.33 (m, 24H), 3.56-3.62 (m, 5H), 3.81-3.91 (m, 6H), 4.19-4.23 (m, 1H), 4.26-4.29 (m, 1H), 4.33-4.39 (m, 1H), 4.43-4.50 (m, 1H),

4.52-4.59 (m, 1H), 6.59-6.63 (m, 2H), 6.63-7.10 (br s, 2H, interfering with the next listed signal), 6.97-7.00 (m, 2H), 7.10-7.54 (br s, 3H), 7.54-7.72 (m, 2H), 7.97 (d, 1H, *J* 7.7 Hz), 8.07-8.74 (m, 4H), 8.74-9.38 (m, 5H), 11.21-13.33 (m, 2H). 4 exchangeable protons (NH, OH) of the presumably 4-fold protonated molecule could not be identified. HRMS: *m/z* $[M+3H]^{3+}$ calcd. for $[C_{60}H_{105}N_{18}O_{16}]^{3+}$ 444.5980, found 444.5989. RP-HPLC (220 nm): 99% (t_R = 6.0 min, k = 6.9). $C_{60}H_{102}N_{18}O_{16} \cdot C_8H_4F_{12}O_8$ (1331.59 + 456.09).

N^α-Arginyl-N^α-methyl-N^ω-{[4-(N-{2-[4,7,10-tris(carboxymethyl)-1,4,7,10-tetraazacyclododecan-1-yl]acetyl})aminobutyl]aminocarbonyl}Arg-Pro-Tyr-*α-tert*-butyl-Gly-Leu tetrakis(hydrotrifluoroacetate) (17).

Compound **17** was prepared from **11** (18.7 mg, 13.3 µmol) and **13** (4.9 mg, 6.0 µmol) according to the general procedure for DOTA-conjugation. Isolation of the protected intermediate: column: Kinetex-XB C18, gradient: 0-18 min: A1/B1 92:8-75:25, 18-40 min: 75:25-38:62, t_R = 28 min). Purification of the product by preparative RP-HPLC (column: Kinetex-XB C18, gradient: 0-35 min: A1/B1 92:8-57:43, t_R = 19 min) afforded **17** as white fluffy solid (3.0 mg, 28%). ^{1}H-NMR (600 MHz, DMSO-d_6): δ 0.81-0.85 (m, 3H), 0.86-0.94 (m, 12H), 1.40-1.79 (m, 18H), 1.92-2.02 (m, 1H), 2.66-2.72 (m, 1H), 2.82-2.95 (m, 5H), 3.02-3.29 (m, 25H), 3.47-3.84 (m, 8H), 4.17-4.25 (m, 1H), 4.25-4.32 (m, 2H), 4.32-4.40 (m, 1H), 4.43-4.49 (m, 1H), 5.12-5.19 (m, 1H), 6.58-6.63 (m, 2H), 6.63-7.17 (br s, 2H, interfering with the next listed signal), 6.98-7.02 (m, 2H), 7.17-7.53 (br s, 2H), 7.53-7.59 (m, 1H), 7.64-7.79 (m, 1H), 7.79-8.53 (m, 7H), 9.02-9.50 (m, 2H), 12.37 (br s, 1H). 7 exchangeable protons (NH, OH) of the presumably 4-fold protonated molecule could not be identified. HRMS: *m/z* $[M+3H]^{3+}$ calcd. for $[C_{60}H_{105}N_{18}O_{16}]^{3+}$ 444.5980, found 444.5994. RP-HPLC (220 nm): > 99% (t_R = 6.2 min, k = 7.2). $C_{60}H_{102}N_{18}O_{16} \cdot C_8H_4F_{12}O_8$ (1331.59 + 456.09).

N^α-Methyl-N^ω-{[8-(N-{2-[4,7,10-tris(carboxymethyl)-1,4,7,10-tetraazacyclododecan-1-yl]acetyl})amino-3,6-dioxaoctyl]aminocarbonyl}Arg-Arg-Pro-Tyr-*α-tert*-butyl-Gly-Leu tetrakis(hydrotrifluoroacetate) (18).

Compound **18** was prepared from **12** (9.3 mg, 6.36 µmol) and **13** (4.67 mg, 5.73 µmol) according to the general procedure for DOTA-conjugation. Isolation of the protected intermediate: column: Gemini-NX C18, gradient: 0-6 min: A1/B1 85:15-79:21, 6-28 min: 79:21-40:60, t_R = 18 min. Purification of the product by preparative RP-HPLC (column: Gemini-NX C18, gradient: 0-35 min: A1/B1 97:3-60:40, t_R = 22 min) afforded **18** as white fluffy solid (9.8 mg, 93%). ^{1}H-NMR (600 MHz, DMSO-d_6): δ 0.78-1.02 (m, 15H), 1.47-1.87 (m, 14H), 1.97-2.03 (m, 1H), 2.47-2.48 (m, 3H), 2.66-2.70 (m, 1H), 2.87-2.90 (m, 1H), 3.09-3.29 (m, 24H), 3.51-3.59 (m, 10H), 3.60-3.80 (m, 9H), 4.19-4.24 (m, 1H), 4.26-4.31 (m, 1H), 4.32-4.39 (m, 1H), 4.42-4.50 (m, 1H), 4.50-4.59 (m, 1H), 6.58-6.62 (m, 2H), 6.62-7.09 (br s, 2H, interfering with the next listed signal), 6.97-7.00 (m, 2H), 7.09-7.46 (br s, 2H), 7.46-7.65 (m, 3H), 7.98 (d, 1H, *J* 8.0 Hz), 8.10-8.76 (m, 4H), 8.76-9.30 (m,

4H), 11.78-12.80 (m, 1H). 6 exchangeable protons (NH, OH) of the presumably 4-fold protonated molecule could not be identified. HRMS: *m/z* [*M*+2H]$^{2+}$ calcd. for $[C_{62}H_{108}N_{18}O_{18}]^{2+}$ 696.4039, found 696.4048. RP-HPLC (220 nm): 99% (t_R = 6.6 min, *k* = 7.7). $C_{62}H_{106}N_{18}O_{18}$ • $C_8H_4F_{12}O_8$ (1391.64 + 456.09).

$N^α$-($N^α$-Methylarginyl)-$N^ω$-{[4-(N-{2-[gallium(III)-4,7,10-tris(carboxymethyl)-1,4,7,10-tetraazacyclododecan-1-yl]acetyl})aminobutyl]aminocarbonyl}Arg-Pro-Tyr-*α-tert*-butyl-Gly-Leu tris(hydrotrifluoroacetate) (19).

Compound **19** was prepared from **14** (4.8 mg, 2.7 µmol) according to the general procedure for the insertion of Ga^{3+}. Purification by preparative RP-HPLC (column: Kinetex-XB C18, gradient: 0-35 min: A2/B1 92:8-57:43, t_R = 17 min) yielded **19** as white fluffy solid (4.5 mg, 95%). ^{1}H-NMR (600 MHz, DMSO-d_6): δ 0.80-0.94 (m, 15H), 1.36-1.89 (m, 18H), 1.96-2.05 (m, 1H), 2.47-2.48 (m, 3H), 2.64-2.71 (m, 1H), 2.85-2.92 (m, 1H), 3.08-3.12 (m, 4H), 3.25-3.30 (m, 9H), 3.48-3.50 (m, 6H), 3.57-3.81 (m, 16H), 4.18-4.24 (m, 1H), 4.25-4.31 (m, 1H), 4.32-4.38 (m, 1H), 4.44-4.51 (m, 1H), 4.53-4.60 (m, 1H), 6.59-6.62 (m, 2H), 6.62-7.15 (br s, 2H, interfering with the next listed signal), 6.98-7.01 (m, 2H), 7.15-7.47 (br s, 2H), 7.47-7.56 (m, 1H), 7.56-7.70 (m, 2H), 7.90-8.05 (m, 1H), 8.15-8.27 (m, 1H), 8.28-8.65 (m, 3H), 8.85-9.02 (m, 3H), 9.14-9.21 (m, 1H), 10.03-10.30 (m, 1H), 12.25-12.80 (m, 1H), 13.03-13.57 (m, 1H). HRMS: *m/z* [*M*+2H]$^{2+}$ calcd. for $[C_{60}H_{101}GaN_{18}O_{16}]^{2+}$ 699.3444, found 699.3455. RP-HPLC (220 nm): > 99% (t_R = 10.0 min, *k* = 12.2). $C_{60}H_{99}GaN_{18}O_{16}$ • $C_6H_3F_9O_6$ (1398.29 + 342.07).

$N^α$-($N^α$-Methylarginyl)-$N^ω$-{[4-(N-{2-[gallium(III)-4,7,10-tris(carboxymethyl)-1,4,7,10-tetraazacyclododecan-1-yl]acetyl})aminobutyl]aminocarbonyl}Arg-Pro-Tyr-Ile-Leu tris(hydrotrifluoroacetate) (20).

Compound **20** was prepared from **15** (4.9 mg, 2.8 µmol) according to the general procedure for the insertion of Ga^{3+}. Purification by preparative RP-HPLC (column: Kinetex-XB C18, gradient: 0-35 min: A2/B1 92:8-57:43, t_R = 17 min) yielded **20** as white fluffy solid (4.9 mg, > 99%). ^{1}H-NMR (600 MHz, DMSO-d_6): δ 0.77-0.93 (m, 12H), 0.99-1.09 (m, 1H), 1.37-1.87 (m, 20H), 1.92-2.06 (m, 1H), 2.46-2.48 (m, 3H), 2.64-2.71 (m, 1H), 2.82-2.89 (m, 1H), 3.03-3.22 (m, 10H), 3.26-3.30 (m, 8H), 3.60-3.82 (m, 17H), 4.17-4.26 (m, 2H), 4.32-4.37 (m, 1H), 4.40-4.48 (m, 1H), 4.52-4.60 (m, 1H), 6.56-6.63 (m, 2H), 6.63-7.13 (br s, 2H, interfering with the next listed signal), 6.97-7.01 (m, 2H), 7.13-7.47 (br s, 2H), 7.47-7.56 (m, 1H), 7.62-7.70 (m, 1H), 7.70-7.81 (m, 1H), 7.83-7.94 (m, 1H), 8.20 (d, 1H, *J* 7.6 Hz), 8.25-8.64 (m, 3H), 8.86-9.20 (m, 4H), 9.99-10.35 (m, 1H), 12.51 (br s, 1H), 13.29 (br s, 1H). HRMS: *m/z* [*M*+2H]$^{2+}$ calcd. for $[C_{60}H_{101}GaN_{18}O_{16}]^{2+}$ 699.3444, found 699.3456. RP-HPLC (220 nm): > 99% (t_R = 10.7 min, *k* = 13.1). $C_{60}H_{99}GaN_{18}O_{16}$ • $C_6H_3F_9O_6$ (1398.29 + 342.07).

N^{α}-Methyl-N^{ω}-{[4-(N-{2-[gallium(III)-4,7,10-tris(carboxymethyl)-1,4,7,10-tetraazacyclododecan-1-yl]acetyl})aminobutyl]aminocarbonyl}Arg-Arg-Pro-Tyr-*α*-*tert*-butyl-Gly-Leu tris(hydrotrifluoroacetate) (21).

Compound **21** was prepared from **16** (4.8 mg, 2.7 μmol) according to the general procedure for the insertion of Ga^{3+}. Purification by preparative RP-HPLC (column: Kinetex-XB C18, gradient: 0-35 min: A2/B1 92:8-57:43, t_R = 17 min) yielded **21** as white fluffy solid (4.3 mg, 92%). ^{1}H-NMR (600 MHz, DMSO-d_6): δ 0.78-0.97 (m, 15H), 1.37-1.86 (m, 18H), 1.95-2.04 (m, 1H), 2.44-2.48 (m, 3H), 2.64-2.71 (m, 1H), 2.85-2.92 (m, 1H), 3.01-3.18 (m, 9H), 3.22-3.34 (m, 13H), 3.64-3.83 (m, 13H), 4.19-4.25 (m, 1H), 4.26-4.30 (m, 1H), 4.33-4.38 (m, 1H), 4.42-4.50 (m, 1H), 4.50-4.59 (m, 1H), 6.58-6.63 (m, 2H), 6.63-7.12 (br s, 2H, interfering with the next listed signal), 6.97-7.00 (m, 2H), 7.12-7.48 (br s, 2H), 7.48-7.57 (m, 1H), 7.57-7.74 (m, 2H), 7.97 (d, 1H, *J* 7.7 Hz), 8.11-8.60 (m, 4H), 8.85-9.22 (m, 4H), 10.03-10.30 (m, 1H), 12.49 (br s, 1H), 13.30 (br s, 1H). HRMS: *m/z* $[M+2H]^{2+}$ calcd. for $[C_{60}H_{101}GaN_{18}O_{16}]^{2+}$ 699.3444, found 699.3448. RP-HPLC (220 nm): > 99% (t_R = 9.5 min, k = 11.5). $C_{60}H_{99}GaN_{18}O_{16} \cdot C_6H_3F_9O_6$ (1398.29 + 342.07).

N^{α}-Arginyl-N^{α}-methyl-N^{ω}-{[4-(N-{2-[gallium(III)-4,7,10-tris(carboxymethyl)-1,4,7,10-tetraazacyclododecan-1-yl]acetyl})aminobutyl]aminocarbonyl}Arg-Pro-Tyr-*α*-*tert*-butyl-Gly-Leu tris(hydrotrifluoroacetate) (22).

Compound **22** was prepared from **17** (1.7 mg, 0.97 μmol) according to the general procedure for the insertion of Ga^{3+}. Purification by preparative RP-HPLC (column: Kinetex-XB C18, gradient: 0-35 min: A2/B1 92:8-57:43, t_R = 16 min) yielded **22** as white fluffy solid (1.7 mg, 99%). ^{1}H-NMR (600 MHz, DMSO-d_6): δ 0.80-0.95 (m, 15H), 1.37-1.79 (m, 18H), 1.94-2.01 (m, 1H), 2.66-2.72 (m, 1H), 2.86-2.95 (m, 4H), 3.04-3.28 (m, 21H), 3.47-3.79 (m, 13H), 4.18-4.25 (m, 1H), 4.26-4.33 (m, 2H), 4.33-4.39 (m, 1H), 4.41-4.48 (m, 1H), 5.10-5.18 (m, 1H), 6.58-6.63 (m, 2H), 6.63-7.14 (br s, 2H, interfering with the next listed signal), 6.98-7.02 (m, 2H), 7.14-7.46 (br s, 2H), 7.46-7.60 (m, 2H), 7.60-7.76 (m, 1H), 7.97 (d, 1H, *J* 7.9 Hz), 8.04-8.61 (m, 6H), 8.78-9.04 (m, 1H), 9.17 (s, 1H), 9.85-10.07 (m, 1H), 12.15-12.86 (m, 1H), 12.91-13.48 (m, 1H). HRMS: *m/z* $[M+2H]^{2+}$ calcd. for $[C_{60}H_{101}GaN_{18}O_{16}]^{2+}$ 699.3444, found 699.3454. RP-HPLC (220 nm): 98% (t_R = 9.6 min, k = 11.6). $C_{60}H_{99}GaN_{18}O_{16} \cdot C_6H_3F_9O_6$ (1398.29 + 342.07).

N^{α}-Methyl-N^{ω}-{[8-(N-{2-[gallium(III)-4,7,10-tris(carboxymethyl)-1,4,7,10-tetraazacyclododecan-1-yl]acetyl})amino-3,6-dioxaoctyl]aminocarbonyl}Arg-Arg-Pro-Tyr-*α*-*tert*-butyl-Gly-Leu tris(hydrotrifluoroacetate) (23).

Compound **23** was prepared from **18** (3.0 mg, 1.62 μmol) according to the general procedure for the insertion of Ga^{3+}. Purification by preparative RP-HPLC (column: Gemini-NX C18, gradient: 0-35 min: A2/B1 97:3-60:40, t_R = 21 min) yielded **23** as white fluffy solid (2.7 mg, 92%). ^{1}H-NMR (600 MHz, DMSO-d_6): δ 0.77-

0.97 (m, 15H), 1.42-1.90 (m, 14H), 1.94-2.04 (m, 1H), 2.45-2.49 (m, 3H), 2.64-2.73 (m, 1H), 2.85-2.92 (m, 1H), 3.07-3.31 (m, 20H), 3.40-3.83 (m, 23H), 4.16-4.25 (m, 1H), 4.25-4.31 (m, 1H), 4.33-4.40 (m, 1H), 4.42-4.59 (m, 2H), 6.56-6.63 (m, 2H), 6.63-7.12 (br s, 2H, interfering with the next listed signal), 6.97-7.00 (m, 2H), 7.12-7.49 (br s, 2H), 7.49-7.81 (m, 3H), 7.98 (d, 1H, *J* 7.7 Hz), 8.22 (d, 1H, *J* 7.6 Hz), 8.28-8.65 (m, 3H), 8.78-9.20 (m, 4H), 10.05-10.40 (m, 1H), 12.48 (br s, 1H), 13.28 (br s, 1H). HRMS: *m/z* $[M+3H]^{3+}$ calcd. for $[C_{62}H_{106}GaN_{18}O_{18}]^{3+}$ 486.5724, found 486.5733. RP-HPLC (220 nm): > 99% (t_R = 10.7 min, k = 13.1). $C_{62}H_{103}GaN_{18}O_{18}$ • $C_6H_3F_9O_6$ (1458.34 + 342.07).

N^{α}-Methyl-N^{ω}-({4-N-[4-(4-fluorophenyl)phenylalanyl]aminobutyl}aminocarbonyl)Arg-Arg-Pro-Tyr-*α-tert*-butyl-Gly-Leu tetrakis(hydrotrifluoroacetate) (25).

HBTU (4.0 mg, 10.4 µmol) and DIPEA (3.6 µL, 20.9 µmol) were added to a solution of HOBt (1.4 mg, 10.4 µmol) and **24** (5.4 mg, 14.9 µmol) in DMF/NMP (80:20 v/v) (15 µL) in a 2-mL reaction vessel. The mixture was vortexed, incubated for 5 min at rt and added to a solution of **10** (29.8 mg, 21.3 µmol) and DIPEA (14.8 µL, 85.2 µmol) in DMF/NMP (80:20 v/v) (18 µL) in a 2-mL reaction vessel with screw cap equipped with a magnetic micro stirrer. After stirring at rt for 60 min, 10% aq TFA (106.1 µL, 106 µmol) was added. The protected intermediate was isolated by preparative HPLC (column: Gemini-NX C18, gradient: 0-6 min: A1/B1 85:15-82:18, 6-15 min: 82:18-70:30, 15-25 min: 70:30-60:40, 25-30 min: 60:40-40:60, t_R = 26 min). After lyophilization of the eluate, TFA/H_2O (95:5 v/v) (2 mL) was added, and the mixture was stirred at rt for 2.5 h. Additional TFA (1 mL) was added and stirring was continued for 1 h. The crude product was dissolved in H_2O (40 mL) and the solution was subjected to lyophilization. Purification of the product by preparative RP-HPLC (column: Gemini-NX C18, gradient: 0-5 min: A1/B1 85:15-70:30, 5-15 min: 70:30-50:50, t_R = 9 min) afforded **25** as white fluffy solid (7.9 mg, 46%). ^{1}H-NMR (600 MHz, DMSO-d_6): δ 0.79-0.95 (m, 15H), 1.27-1.38 (m, 4H), 1.43-1.88 (m, 14H), 1.93-2.03 (m, 1H), 2.38-2.49 (m, 3H), 2.64-2.72 (m, 1H), 2.85-2.93 (m, 1H), 2.95-3.17 (m, 8H), 3.18-3.25 (m, 2H), 3.43-3.88 (m, 3H), 3.88-3.98 (m, 1H), 4.16-4.31 (m, 2H), 4.31-4.40 (m, 1H), 4.40-4.58 (m, 2H), 6.57-6.62 (m, 2H), 6.62-7.10 (br s, 2H, interfering with the next listed signal), 6.97-7.00 (m, 2H), 7.10-7.57 (br s, 2H, interfering with the next two listed signals), 7.27-7.32 (m, 4H), 7.40-7.50 (m, 1H), 7.57-7.78 (m, 6H), 7.78-8.60 (m, 8H), 8.60-9.44 (m, 4H), 10.32 (br s, 1H), 12.49 (br s, 1H). 1 exchangeable proton (NH, OH) of the presumably 4-fold protonated molecule could not be identified. HRMS: *m/z* $[M+2H]^{2+}$ calcd. for $[C_{59}H_{90}FN_{15}O_{10}]^{2+}$ 593.8484, found 593.8491. RP-HPLC (220 nm): > 99% (t_R = 10.9 min, k = 13.3). $C_{59}H_{88}FN_{15}O_{10}$ • $C_8H_4F_{12}O_8$ (1186.45 + 456.09).

N^{α}-Methyl-N^{ω}-({8-N-[4-(4-fluorophenyl)-phenylalanyl]amino-3,6-dioxaoctyl}aminocarbonyl)Arg-Arg-Pro-Tyr-*α-tert*-butyl-Gly-Leu tetrakis(hydrotrifluoroacetate) (26).

Compound **26** was prepared from **12** (29.2 mg, 20.0 µmol) and **24** (5.0 mg, 14.0 µmol) according to the procedure for the synthesis of **25**. Isolation of the protected intermediate: column: Gemini-NX C18, gradient: 0-6 min: A1/B1 85:15-82:18, 6-15 min: 82:18-70:30, 15-25 min: 70:30-60:40, 25-30 min: 60:40-40:60, t_R = 26 min. Purification of the product by preparative RP-HPLC (column: Gemini-NX C18, gradient: 0-5 min: A1/B1 85:15-70:30, 5-15 min: 70:30-50:50, t_R = 10 min) afforded **26** as white fluffy solid (7.76 mg, 47%). ^{1}H-NMR (600 MHz, DMSO-d_6): δ 0.80-0.95 (m, 15H), 1.38-1.90 (m, 14H), 1.94-2.05 (m, 1H), 2.40-2.48 (m, 3H), 2.64-2.71 (m, 1H), 2.86-2.92 (m, 1H), 2.97-3.02 (m, 1H), 3.04-3.26 (m, 8H), 3.34-3.44 (m, 5H), 3.45-3.49 (m, 4H), 3.52-3.64 (m, 2H), 3.64-3.92 (m, 1H), 3.95-4.03 (m, 1H), 4.18-4.30 (m, 2H), 4.32-4.39 (m, 1H), 4.43-4.59 (m, 2H), 6.57-6.63 (m, 2H), 6.63-7.11 (br s, 2H, interfering with the next listed signal), 6.97-7.00 (m, 2H), 7.11-7.57 (br s, 2H, interfering with the next two listed signals), 7.27-7.33 (m, 4H), 7.39-7.49 (m, 1H), 7.57-7.72 (m, 6H), 7.97 (d, 1H, *J* 7.7 Hz), 8.00-8.70 (m, 7H), 8.70-9.37 (m, 4H), 10.30 (br s, 1H), 12.49 (br s, 1H). 1 exchangeable proton (NH, OH) of the presumably 4-fold protonated molecule could not be identified. HRMS: *m/z* $[M+H]^+$ calcd. for $[C_{61}H_{93}FN_{15}O_{12}]^+$ 1246.7107, found 1246.7112. RP-HPLC (220 nm): 99% (t_R = 11.5 min, k = 14.1). $C_{61}H_{92}FN_{15}O_{12} \cdot C_8H_4F_{12}O_8$ (1246.50 + 456.09).

N^{α}-Methyl-N^{ω}-{[4-N-(4-aminomethyl-3-fluorobenzoyl)aminobutyl]aminocarbonyl}Arg-Arg-Pro-Tyr-*α-tert*-butyl-Gly-Leu tetrakis(hydrotrifluoroacetate) (28).

Compound **28** was prepared from **10** (16.6 mg, 11.9 µmol) and **27** (2.2 mg, 8.30 µmol) according to the procedure for the synthesis of **25** (modification: stirring of the mixture for 75 min instead of 60 min). Isolation of the protected intermediate: column: Gemini-NX C18, gradient: 0-6 min: A1/B1 85:15-82:18, 6-16 min: 82:18-70:30, 16-30 min: 70:30-60:40, t_R = 23 min. Purification of the product by preparative RP-HPLC (column: Gemini-NX C18, gradient: 0-5 min: A1/B1 85:15-80:20, 5-15 min: 80:20-60:40, t_R = 11 min) afforded **28** as white fluffy solid (2.2 mg, 24%). ^{1}H-NMR (600 MHz, DMSO-d_6): δ 0.77-0.97 (m, 15H), 1.39-1.90 (m, 18H), 1.94-2.05 (m, 1H), 2.45-2.49 (m, 3H), 2.64-2.71 (m, 1H), 2.84-2.94 (m, 1H), 3.05-3.17 (m, 4H), 3.23-3.28 (m, 4H), 3.50-3.64 (m, 2H), 3.73-3.88 (m, 1H), 4.08-4.17 (m, 2H), 4.17-4.24 (m, 1H), 4.26-4.31 (m, 1H), 4.32-4.40 (m, 1H), 4.41-4.60 (m, 2H), 6.57-6.62 (m, 2H), 6.62-7.09 (br s, 2H, interfering with the next listed signal), 6.97-7.00 (m, 2H), 7.09-7.44 (br s, 2H), 7.44-7.67 (m, 4H), 7.67-7.76 (m, 2H), 7.98 (d, 1H, *J* 7.7 Hz), 8.05-8.59 (m, 6H), 8.59-8.66 (m, 1H), 8.73-9.25 (m, 4H), 9.86-10.16 (m, 1H), 12.44 (br s, 1H). 1 exchangeable proton (NH, OH) of the presumably 4-fold protonated molecule could not be identified. HRMS: *m/z* $[M+2H]^{2+}$ calcd. for $[C_{52}H_{84}FN_{15}O_{10}]^{2+}$ 548.8249, found 548.8262. RP-HPLC (220 nm): > 99% (t_R = 7.0 min, k = 8.2). $C_{52}H_{82}FN_{15}O_{10} \cdot C_8H_4F_{12}O_8$ (1096.32 + 456.09).

N^{α}-Methyl-N^{ω}-{[8-N-(4-aminomethyl-3-fluorobenzoyl)amino-3,6-dioxaoctyl]aminocarbonyl}Arg-Arg-Pro-Tyr-*α*-*tert*-butyl-Gly-Leu tetrakis(hydrotrifluoroacetate) (29).

Compound **29** was prepared from **12** (12.2 mg, 8.35 µmol) and **27** (1.6 mg, 5.84 µmol) according to the procedure for the synthesis of **25**. Isolation of the protected intermediate: column: Gemini-NX C18, gradient: 0-6 min: A1/B1 85:15-82:18, 6-15 min: 82:18-70:30, 15-25 min: 70:30-60:40, t_R = 22 min. Purification of the product by preparative RP-HPLC (column: Gemini-NX C18, gradient: 0-5 min: A1/B1 85:15-80:20, 5-15 min: 80:20-60:40, t_R = 11 min) afforded **29** as white fluffy solid (2.1 mg, 32%). ^{1}H-NMR (600 MHz, DMSO-d_6): δ 0.78-0.96 (m, 15H), 1.44-1.88 (m, 14H), 1.95-2.04 (m, 1H), 2.44-2.48 (m, 3H), 2.65-2.72 (m, 1H), 2.85-2.91 (m, 1H), 3.07-3.15 (m, 2H), 3.23-3.29 (m, 4H), 3.42-3.47 (m, 4H), 3.53-3.61 (m, 8H), 3.78-3.80 (m, 1H), 4.12-4.15 (m, 2H), 4.20-4.23 (m, 1H), 4.27-4.29 (m, 1H), 4.34-4.38 (m, 1H), 4.44-4.57 (m, 2H), 6.57-6.62 (m, 2H), 6.62-7.11 (br s, 2H, interfering with the next listed signal), 6.97-7.00 (m, 2H), 7.11-7.46 (br s, 2H), 7.46-7.54 (m, 1H), 7.54-7.77 (m, 5H), 7.97 (d, 1H, *J* 7.9 Hz), 8.11-8.65 (m, 6H), 8.65-8.72 (m, 1H), 8.72-9.31 (m, 5H), 10.05-10.33 (m, 1H), 12.49 (br s, 1H). HRMS: *m/z* $[M+2H]^{2+}$ calcd. for $[C_{54}H_{88}FN_{15}O_{12}]^{2+}$ 578.8355, found 578.8369. RP-HPLC (220 nm): 99% (t_R = 7.1 min, k = 8.3). $C_{54}H_{86}FN_{15}O_{12}$ · $C_8H_4F_{12}O_8$ (1156.37 + 456.09).

N^{α}-Methyl-N^{ω}-({8-N-[N^{α}-(6-aminohexanoyl)-4-(4-fluorophenyl)-phenylalanyl]amino-3,6-dioxaoctyl}aminocarbonyl)Arg-Arg-Pro-Tyr-*α*-*tert*-butyl-Gly-Leu tetrakis(hydrotrifluoroacetate) (31).

The reaction was performed in a 2-mL reaction vessel with screw cap, equipped with a magnetic micro stirrer. DIPEA (2.5 µL, 14.3 µmol) and a solution of *N*-Boc-6-aminohexanoic acid succinimidyl ester (**30**) (1.06 mg, 3.22 µmol) in anhydrous DMF/NMP (75:25 v/v) (4 µL) were added to a solution of **26** (6.09 mg, 3.58 µmol) in anhydrous DMF/NMP (75:25 v/v) (41 µL). The mixture was stirred at rt for 45 min followed by the addition of 10% aq TFA (14.3 µL, 14.3 µmol). The protected intermediate was isolated by preparative HPLC (column: Gemini-NX C18, gradient: 0-5 min: A1/B1 85:15-70:30, 5-15 min: 70:30-50:50, 15-25 min: 50:50-30:70, t_R = 14 min) and the eluate was subjected to lyophilization. TFA/H_2O (95:5 v/v) (2 mL) was added, and the mixture was stirred at rt for 3 h. Additional TFA (1 mL) was added and stirring was continued for 30 min. The crude product was dissolved in H_2O (40 mL) and the mixture was subjected to lyophilization. Purification of the product by preparative RP-HPLC (column: Gemini-NX C18, gradient: 0-5 min: A1/B1 85:15-70:30, 5-15 min: 70:30-50:50, t_R = 10 min) gave **31** as white fluffy solid (3.84 mg, 66%). HRMS: *m/z* $[M+2H]^{2+}$ calcd. for $[C_{67}H_{105}FN_{16}O_{13}]^{2+}$ 680.4010, found 680.4019. RP-HPLC (220 nm): > 99% (t_R = 12.2 min, k = 15.1). $C_{67}H_{103}FN_{16}O_{13}$ · $C_8H_4F_{12}O_8$ (1359.66 + 456.09).

***N*^α^-Methyl-*N*^ω^-({8-N-[*N*^α^-(6-N-{2-[4,7,10-tris(carboxymethyl)-1,4,7,10-tetraazacyclododecan-1-yl]acetyl}-aminohexanoyl)-4-(4-fluorophenyl)-phenylalanyl]amino-3,6-dioxaoctyl}aminocarbonyl)Arg-Arg-Pro-Tyr-*α*-*tert*-butyl-Gly-Leu tetrakis(hydrotrifluoroacetate) (32).**

Compound **32** was prepared from **31** (3.84 mg, 2.11 μmol) and **13** (1.55 mg, 1.90 μmol) according to the general procedure for DOTA-conjugation (modification: stirring of the mixture for 45 min instead of 30 min). Isolation of the protected intermediate: column: Gemini-NX C18, gradient: 0-5 min: A1/B1 85:15-70:30, 5-15 min: 70:30-50:50, t_R = 14 min. Purification of the product by preparative RP-HPLC (column: Gemini-NX C18, gradient: 0-5 min: A1/B1 85:15-70:30, 5-15 min: 70:30-50:50, t_R = 10 min) afforded **32** as white fluffy solid (2.9 mg, 69%). ^{1}H-NMR (600 MHz, DMSO-d_6): δ 0.80-0.94 (m, 15H), 1.07-1.18 (m, 2H), 1.28-1.43 (m, 4H), 1.45-1.89 (m, 14H), 1.95-2.10 (m, 3H), 2.44-2.47 (m, 3H), 2.64-2.71 (m, 1H), 2.72-3.26 (m, 29H), 3.40-3.73 (m, 18H), 3.76-3.83 (m, 1H), 4.17-4.25 (m, 1H), 4.25-4.30 (m, 1H), 4.32-4.39 (m, 1H), 4.42-4.60 (m, 3H), 6.58-6.62 (m, 2H), 6.62-7.12 (br s, 2H, interfering with the next listed signal), 6.97-7.00 (m, 2H), 7.12-7.45 (br s, 2H, interfering with the next listed signal), 7.25-7.32 (m, 4H), 7.45-7.52 (m, 1H), 7.52-7.56 (m, 2H), 7.56-7.72 (m, 4H), 7.81-8.30 (m, 5H), 8.30-9.44 (m, 6H), 10.22-10.69 (m, 1H), 11.12-12.83 (m, 2H). 4 exchangeable protons (NH, OH) of the presumably 4-fold protonated molecule could not be identified. HRMS: *m/z* [*M*+2H]$^{2+}$ calcd. for $[C_{83}H_{131}FN_{20}O_{20}]^{2+}$ 873.4911, found 873.4920. RP-HPLC (220 nm): 99% (t_R = 12.5 min, *k* = 15.4). $C_{83}H_{129}FN_{20}O_{20} \cdot C_8H_4F_{12}O_8$ (1746.06 + 456.09).

***N*^α^-Methyl-*N*^ω^-({8-N-[*N*^α^-(6-N-{2-[gallium(III)-4,7,10-tris(carboxymethyl)-1,4,7,10-tetraazacyclododecan-1-yl]acetyl}-aminohexanoyl)-4-(4-fluorophenyl)-phenylalanyl]amino-3,6-dioxaoctyl}aminocarbonyl)Arg-Arg-Pro-Tyr-*α*-*tert*-butyl-Gly-Leu tris(hydrotrifluoroacetate) (33).**

Compound **33** was prepared from **32** (1.81 mg, 0.82 μmol) according to the general procedure for the insertion of Ga^{3+}. Purification by preparative RP-HPLC (column: Gemini-NX C18, gradient: 0-5 min: A1/B1 85:15-75:25, 5-10 min: 75:25-67:33, 10-20 min: 67:33-55:45, t_R = 14 min) yielded **33** as white fluffy solid (1.76 mg, 99%). ^{1}H-NMR (600 MHz, DMSO-d_6): δ 0.81-0.94 (m, 15H), 1.07-1.16 (m, 2H), 1.27-1.41 (m, 4H), 1.47-1.64 (m, 8H), 1.64-1.87 (m, 6H), 1.95-2.07 (m, 3H), 2.44-2.47 (m, 3H), 2.64-2.71 (m, 1H), 2.74-2.81 (m, 1H), 2.85-2.91 (m, 1H), 2.91-3.02 (m, 3H), 3.07-3.10 (m, 1H), 3.10-3.29 (m, 18H), 3.34-3.37 (m, 4H), 3.37-3.49 (m, 7H), 3.49-3.51 (m, 3H), 3.58-3.76 (m, 10H), 4.17-4.25 (m, 1H), 4.25-4.31 (m, 1H), 4.33-4.38 (m, 1H), 4.43-4.60 (m, 3H), 6.57-6.62 (m, 2H), 6.62-7.10 (br s, 2H, interfering with the next listed signal), 6.97-7.01 (m, 2H), 7.10-7.45 (br s, 2H, interfering with the next listed signal), 7.25-7.32 (m, 4H), 7.45-7.52 (m, 1H), 7.52-7.72 (m, 6H), 7.86-8.12 (m, 3H), 8.12-8.61 (m, 4H), 8.78-9.21 (m, 4H), 9.85-10.20 (m, 1H), 12.48 (br s, 1H), 13.27 (br s, 1H). HRMS: *m/z* [*M*+2H]$^{2+}$ calcd. for $[C_{83}H_{128}FGaN_{20}O_{20}]^{2+}$ 906.4421, found 906.4431. RP-HPLC (220 nm): 99% (t_R = 12.5 min, *k* = 15.4). $C_{83}H_{126}FGaN_{20}O_{20} \cdot C_6H_3F_9O_6$ (1812.76 + 342.07).

***N*^{α}-Methyl-*N*^{ω}-{[4-N-(*N*^{α}-{2-[4,7,10-tris(carboxymethyl)-1,4,7,10-tetraazacyclododecan-1-yl]acetyl}-4-{4-fluorophenyl}phenylalanyl)aminobutyl]aminocarbonyl}Arg-Arg-Pro-Tyr-*α*-*tert*-butyl-Gly-Leu tetrakis(hydrotrifluoroacetate) (34).**

Compound **34** was prepared from **25** (7.9 mg, 4.81 µmol) and **13** (3.14 mg, 3.85 µmol) according to the general procedure for DOTA-conjugation. Isolation of the protected intermediate: column: Gemini-NX C18, gradient: 0-5 min: A1/B1 85:15-70:30, 5-15 min: 70:30-50:50, t_R = 13 min. Purification of the product by preparative RP-HPLC (column: Gemini-NX C18, gradient: 0-5 min: A1/B1 85:15-70:30, 5-15 min: 70:30-50:50, t_R = 9 min) afforded **34** as white fluffy solid (6.1 mg, 78%). ^{1}H-NMR (600 MHz, DMSO-d_6): δ 0.81-0.94 (m, 15H), 1.33-1.44 (m, 4H), 1.46-1.87 (m, 14H), 1.95-2.05 (m, 1H), 2.46-2.49 (m, 3H), 2.65-2.70 (m, 1H), 2.77-3.26 (m, 28H), 3.55-3.67 (m, 5H), 3.67-3.93 (m, 5H), 4.19-4.24 (m, 1H), 4.26-4.30 (m, 1H), 4.33-4.39 (m, 1H), 4.42-4.60 (m, 3H), 6.58-6.62 (m, 2H), 6.62-7.12 (br s, 2H, interfering with the next listed signal), 6.97-7.00 (m, 2H), 7.12-7.54 (br s, 2H, interfering with the next listed signal), 7.26-7.33 (m, 4H), 7.54-7.57 (m, 2H), 7.57-7.71 (m, 4H), 7.71-8.53 (m, 6H), 8.53-9.32 (m, 6H), 11.49 (br s, 1H), 12.47 (br s, 1H). 4 exchangeable protons (NH, OH) of the presumably 4-fold protonated molecule could not be identified. HRMS: *m/z* $[M+2H]^{2+}$ calcd. for $[C_{75}H_{116}FN_{19}O_{17}]^{2+}$ 786.9385, found 786.9394. RP-HPLC (220 nm): > 99% (t_R = 11.2 min, k = 13.7). $C_{75}H_{114}FN_{19}O_{17}$ · $C_8H_4F_{12}O_8$ (1572.85 + 456.09).

***N*^{α}-Methyl-*N*^{ω}-{[8-N-(*N*^{α}-{2-[4,7,10-tris(carboxymethyl)-1,4,7,10-tetraazacyclododecan-1-yl]acetyl}-4-{4-fluorophenyl}-phenylalanyl)amino-3,6-dioxaoctyl]aminocarbonyl}Arg-Arg-Pro-Tyr-*α*-*tert*-butyl-Gly-Leu tetrakis(hydrotrifluoroacetate) (35).**

Compound **35** was prepared from **26** (6.6 mg, 3.86 µmol) and **13** (2.52 mg, 3.09 µmol) according to the general procedure for DOTA-conjugation. Isolation of the protected intermediate: column: Gemini-NX C18, gradient: 0-5 min: A1/B1 85:15-70:30, 5-15 min: 70:30-50:50, t_R = 13 min. Purification of the product by preparative RP-HPLC (column: Gemini-NX C18, gradient: 0-5 min: A1/B1 85:15-70:30, 5-15 min: 70:30-50:50, t_R = 10 min) afforded **35** as white fluffy solid (5.2 mg, 81%). ^{1}H-NMR (600 MHz, DMSO-d_6): δ 0.80-0.95 (m, 15H), 1.47-1.87 (m, 14H), 1.95-2.03 (m, 1H), 2.46-2.48 (m, 3H), 2.64-2.70 (m, 1H), 2.75-2.79 (m, 1H), 2.84-2.91 (m, 3H), 3.23-3.41 (m, 24H), 3.49-3.61 (m, 16H), 3.77-3.87 (m, 2H), 4.18-4.24 (m, 1H), 4.27-4.29 (m, 1H), 4.32-4.40 (m, 1H), 4.43-4.50 (m, 1H), 4.50-4.58 (m, 1H), 4.60-4.71 (m, 1H), 6.56-6.62 (m, 2H), 6.62-7.09 (br s, 2H, interfering with the next listed signal), 6.97-7.00 (m, 2H), 7.09-7.43 (br s, 2H, interfering with the next listed signal), 7.26-7.34 (m, 4H), 7.43-7.52 (m, 1H), 7.52-7.64 (m, 4H), 7.64-7.69 (m, 2H), 7.87-8.02 (m, 1H), 8.07-8.83 (m, 5H), 8.83-9.29 (m, 4H), 10.19-11.03 (m, 1H), 11.99-12.87 (m, 1H). 5 exchangeable protons (NH, OH) of the presumably 4-fold protonated molecule could not be identified. HRMS: *m/z* $[M+2H]^{2+}$ calcd. for $[C_{77}H_{120}FN_{19}O_{19}]^{2+}$ 816.9490, found 816.9497. RP-HPLC (220 nm): 97% (t_R = 12.1 min, k = 14.9). $C_{77}H_{118}FN_{19}O_{19}$ · $C_8H_4F_{12}O_8$ (1632.90 + 456.09).

N^{α}-Methyl-N^{ω}-{[4-N-(N^{α}-{2-[gallium(III)-4,7,10-tris(carboxymethyl)-1,4,7,10-tetraazacyclododecan-1-yl]acetyl}-4-(4-fluorophenyl)phenylalanyl)aminobutyl]aminocarbonyl}Arg-Arg-Pro-Tyr-*α*-*tert*-butyl-Gly-Leu tris(hydrotrifluoroacetate) (36).

Compound **36** was prepared from **34** (3.49 mg, 1.72 μmol) according to the general procedure for the insertion of Ga^{3+}. Purification by preparative RP-HPLC (column: Gemini-NX C18, gradient: 0-5 min: A1/B1 85:15-75:25, 5-10 min: 75:25-67:33, 10-20 min: 67:33-55:45, t_R = 12 min) yielded **36** as white fluffy solid (3.26 mg, 96%). ^{1}H-NMR (600 MHz, DMSO-d_6): δ 0.79-0.95 (m, 15H), 1.31-1.43 (m, 4H), 1.44-1.89 (m, 14H), 1.95-2.04 (m, 1H), 2.43-2.48 (m, 3H), 2.64-2.85 (m, 3H), 2.85-3.29 (m, 22H), 3.35-3.90 (m, 14H), 4.17-4.25 (m, 1H), 4.25-4.31 (m, 1H), 4.31-4.41 (m, 1H), 4.41-4.59 (m, 3H), 6.57-6.63 (m, 2H), 6.63-7.12 (br s, 2H, interfering with the next listed signal), 6.97-7.00 (m, 2H), 7.12-7.45 (br s, 2H, interfering with the next listed signal), 7.26-7.33 (m, 4H), 7.45-7.53 (m, 1H), 7.53-7.76 (m, 6H), 7.97 (d, 1H, *J* 7.8 Hz), 8.03-8.16 (m, 1H), 8.22 (d, 1H, *J* 7.3 Hz), 8.28-8.65 (m, 2H), 8.68-9.26 (m, 5H), 9.75-10.27 (m, 1H), 12.47 (br s, 1H), 13.27 (br s, 1H). HRMS: *m/z* $[M+3H]^{3+}$ calcd. for $[C_{75}H_{114}FGaN_{19}O_{17}]^{3+}$ 546.9288, found 546.9299. RP-HPLC (220 nm): > 99% (t_R = 14.4 min, k = 17.9). $C_{75}H_{111}FGaN_{19}O_{17}$ • $C_6H_3F_9O_6$ (1639.55 + 342.07).

N^{α}-Methyl-N^{ω}-{[8-N-(N^{α}-{2-[gallium(III)-4,7,10-tris(carboxymethyl)-1,4,7,10-tetraazacyclododecan-1-yl]acetyl}-4-{4-fluorophenyl}-phenylalanyl)amino-3,6-dioxaoctyl]aminocarbonyl}Arg-Arg-Pro-Tyr-*α*-*tert*-butyl-Gly-Leu tris(hydrotrifluoroacetate) (37).

Compound **37** was prepared from **35** (3.33 mg, 1.59 μmol) according to the general procedure for the insertion of Ga^{3+}. Purification by preparative RP-HPLC (column: Gemini-NX C18, gradient: 0-5 min: A1/B1 85:15-75:25, 5-10 min: 75:25-67:33, 10-20 min: 67:33-55:45, t_R = 12 min) yielded **37** as white fluffy solid (3.09 mg, 95%). ^{1}H-NMR (600 MHz, DMSO-d_6): δ 0.80-0.94 (m, 15H), 1.47-1.87 (m, 14H), 1.95-2.05 (m, 1H), 2.44-2.47 (m, 3H), 2.64-2.71 (m, 1H), 2.71-2.96 (m, 4H), 3.01-3.28 (m, 19H), 3.33-3.46 (m, 9H), 3.46-3.68 (m, 12H), 3.68-3.83 (m, 2H), 4.17-4.25 (m, 1H), 4.26-4.30 (m, 1H), 4.32-4.40 (m, 1H), 4.42-4.61 (m, 3H), 6.54-6.62 (m, 2H), 6.62-7.11 (br s, 2H, interfering with the next listed signal), 6.97-7.00 (m, 2H), 7.11-7.42 (br s, 2H, interfering with the next listed signal), 7.26-7.33 (m, 4H), 7.42-7.52 (m, 1H), 7.52-7.85 (m, 6H), 7.88-8.68 (m, 5H), 8.68-9.29 (m, 5H), 10.00-10.36 (m, 1H), 12.49 (br s, 1H), 13.28 (br s, 1H). HRMS: *m/z* $[M+2H]^{2+}$ calcd. for $[C_{77}H_{117}FGaN_{19}O_{19}]^{2+}$ 849.9001, found 849.9012. RP-HPLC (220 nm): > 99% (t_R = 14.5 min, k = 18.1). $C_{77}H_{115}FGaN_{19}O_{19}$ • $C_6H_3F_9O_6$ (1699.60 + 342.07).

N^{α}-Methyl-Arg-Arg-Pro-Tyr-2-((1*R*)-1-methylpropyl)-Gly-Leu tris(hydrotrifluoroacetate) (38).

Peptide **38** was synthesized according to the general procedure for SPPS using a H-Leu-2-ClTrt resin (loading 0.79 mmol/g) (40 mg, 0.0316 mmol). Purification by preparative RP-HPLC (column: Gemini-NX C18, gradient: 0-5 min: A1/B1 95:5-90:10, 5-10 min: 90:10-80:20, 10-30 min: 80:20-60:40, t_R = 18 min) afforded **38**

as white fluffy solid (13.8 mg, 37%). ^{1}H-NMR (600 MHz, DMSO-d_6): δ 0.74-0.80 (m, 3H), 0.82-0.87 (m, 6H), 0.88-0.92 (m, 3H), 1.00-1.09 (m, 1H), 1.32-1.40 (m, 1H), 1.40-1.59 (m, 7H), 1.59-1.89 (m, 8H), 1.95-2.04 (m, 1H), 2.42-2.48 (m, 3H), 2.65-2.75 (m, 1H), 2.85-2.92 (m, 1H), 3.04-3.16 (m, 4H), 3.53-3.64 (m, 2H), 3.74-3.83 (m, 1H), 4.18-4.26 (m, 1H), 4.32-4.39 (m, 2H), 4.41-4.60 (m, 2H), 6.59-6.63 (m, 2H), 6.63-7.18 (br s, 4H, interfering with the next listed signal), 6.99-7.01 (m, 2H), 7.19-7.60 (br s, 4H), 7.60-7.76 (m, 3H), 7.93 (d, 1H, *J* 7.9 Hz), 8.13 (d, 1H, *J* 7.7 Hz), 8.73-9.06 (m, 3H), 9.11-9.23 (m, 1H), 12.47 (br s, 1H). HRMS: *m/z* $[M+2H]^{2+}$ calcd. for $[C_{39}H_{68}N_{12}O_8]^{2+}$ 416.2636, found 416.2645. RP-HPLC (220 nm): 99% (t_R = 6.7 min, *k* = 7.8). $C_{39}H_{66}N_{12}O_8 \cdot C_6H_3F_9O_6$ (831.03 + 342.07).

N^{α}-Methyl-Arg-Arg-Pro-Tyr-*α,α*-diethyl-Gly-Leu tris(hydrotrifluoroacetate) (39).

Peptide **39** was synthesized according to the general procedure for SPPS using a H-Leu-2-ClTrt resin (loading 0.79 mmol/g) (40 mg, 0.0316 mmol). Purification by preparative RP-HPLC (column: Gemini-NX C18, gradient: 0-5 min: A1/B1 95:5-90:10, 5-10 min: 90:10-80:20, 10-30 min: 80:20-55:45, t_R = 18 min) afforded **39** as white fluffy solid (9.1 mg, 25%). ^{1}H-NMR (600 MHz, DMSO-d_6): δ 0.31-0.49 (m, 3H), 0.57-0.70 (m, 3H), 0.79-0.82 (m, 3H), 0.87-0.90 (m, 3H), 1.40-1.87 (m, 15H), 1.87-2.01 (m, 1H), 2.01-2.14 (m, 1H), 2.25-2.38 (m, 2H), 2.43-2.48 (m, 3H), 2.65-2.75 (m, 1H), 2.88-2.94 (m, 1H), 3.04-3.18 (m, 4H), 3.51-3.64 (m, 2H), 3.72-3.88 (m, 1H), 4.12-4.22 (m, 1H), 4.23-4.41 (m, 2H), 4.46-4.62 (m, 1H), 6.61-6.70 (m, 2H), 6.70-7.14 (br s, 4H, interfering with the next listed signal), 7.03-7.06 (m, 2H), 7.14-7.54 (br s, 4H, interfering with the next listed signal), 7.37-7.40 (m, 1H), 7.54-7.76 (m, 2H), 8.16 (d, 1H, *J* 7.9 Hz), 8.46 (d, 1H, *J* 7.4 Hz), 8.68-9.12 (m, 3H), 9.15-9.25 (m, 1H), 12.57 (br s, 1H). HRMS: *m/z* $[M+2H]^{2+}$ calcd. for $[C_{39}H_{68}N_{12}O_8]^{2+}$ 416.2636, found 416.2644. RP-HPLC (220 nm): 99% (t_R = 7.4 min, *k* = 8.7). $C_{39}H_{66}N_{12}O_8 \cdot C_6H_3F_9O_6$ (831.03 + 342.07).

N^{α}-Methyl-Arg-Arg-Pro-Tyr-*α*-cyclopropyl-Gly-Leu tris(hydrotrifluoroacetate) (40).

Peptide **40** was synthesized according to the general procedure for SPPS using a H-Leu-2-ClTrt resin (loading 0.79 mmol/g) (40 mg, 0.0316 mmol). Purification by preparative RP-HPLC (column: Gemini-NX C18, gradient: 0-5 min: A1/B1 95:5-90:10, 5-10 min: 90:10-80:20, 10-30 min: 80:20-55:45, t_R = 15 min) afforded **40** as white fluffy solid (25.3 mg, 69%). ^{1}H-NMR (600 MHz, DMSO-d_6): δ 0.29-0.35 (m, 1H), 0.35-0.42 (m, 2H), 0.42-0.48 (m, 1H), 0.83-0.87 (m, 3H), 0.87-0.92 (m, 3H), 0.99-1.05 (m, 1H), 1.42-1.87 (m, 14H), 1.96-2.06 (m, 1H), 2.42-2.48 (m, 3H), 2.65-2.73 (m, 1H), 2.84-2.91 (m, 1H), 3.06-3.15 (m, 4H), 3.54-3.63 (m, 2H), 3.76-3.86 (m, 1H), 3.91-3.98 (m, 1H), 4.21-4.30 (m, 1H), 4.30-4.37 (m, 1H), 4.37-4.44 (m, 1H), 4.46-4.58 (m, 1H), 6.55-6.65 (m, 2H), 6.65-7.13 (br s, 4H, interfering with the next listed signal), 6.99-7.02 (m, 2H), 7.13-7.56 (br s, 4H), 7.56-7.72 (m, 2H), 7.79-8.01 (m, 2H), 8.08-8.19 (m, 1H), 8.73-9.04 (m, 3H), 9.18 (s,

1H), 12.54 (br s, 1H). HRMS: *m/z* [*M*+2H]$^{2+}$ calcd. for [$C_{38}H_{64}N_{12}O_8$]$^{2+}$ 408.2480, found 408.2487. RP-HPLC (220 nm): 97% (t_R = 5.2 min, *k* = 5.8). $C_{38}H_{62}N_{12}O_8$ • $C_6H_3F_9O_6$ (814.99 + 342.07).

N^{α}-Methyl-Arg-Arg-Pro-Tyr-*β*-cyclopropyl-Ala-Leu tris(hydrotrifluoroacetate) (41).

Peptide **41** was synthesized according to the general procedure for SPPS using a H-Leu-2-ClTrt resin (loading 0.79 mmol/g) (40 mg, 0.0316 mmol). Purification by preparative RP-HPLC (column: Gemini-NX C18, gradient: 0-5 min: A1/B1 95:5-90:10, 5-10 min: 90:10-78:22, 10-30 min: 78:22-50:50, t_R = 16 min) afforded **41** as white fluffy solid (17.5 mg, 47%). ^{1}H-NMR (600 MHz, DMSO-d_6): δ 0.02-0.15 (m, 2H), 0.27-0.44 (m, 2H), 0.62-0.77 (m, 1H), 0.82-0.93 (m, 6H), 1.36-1.89 (m, 16H), 1.95-2.05 (m, 1H), 2.40-2.48 (m, 3H), 2.66-2.75 (m, 1H), 2.86-2.92 (m, 1H), 3.05-3.14 (m, 4H), 3.54-3.55 (m, 1H), 3.60-3.62 (m, 1H), 3.75-3.80 (m, 1H), 4.21-4.29 (m, 1H), 4.29-4.44 (m, 3H), 4.49-4.60 (m, 1H), 6.57-6.62 (m, 2H), 6.62-7.12 (br s, 4H, interfering with the next listed signal), 7.00-7.02 (m, 2H), 7.12-7.57 (br s, 4H), 7.57-7.73 (m, 2H), 7.77-7.96 (m, 2H), 8.11-8.21 (m, 1H), 8.74-9.03 (m, 3H), 9.09-9.27 (m, 1H), 12.55 (br s, 1H). HRMS: *m/z* [*M*+3H]$^{3+}$ calcd. for [$C_{39}H_{67}N_{12}O_8$]$^{3+}$ 277.1729, found 277.1739. RP-HPLC (220 nm): 98% (t_R = 5.9 min, *k* = 6.8). $C_{39}H_{64}N_{12}O_8$ • $C_6H_3F_9O_6$ (829.02 + 342.07).

N^{α}-Methyl-Arg-Arg-Pro-Tyr-*α*-cyclobutyl-Gly-Leu tris(hydrotrifluoroacetate) (42).

Peptide **42** was synthesized according to the general procedure for SPPS using a H-Leu-2-ClTrt resin (loading 0.79 mmol/g) (40 mg, 0.0316 mmol). Purification by preparative RP-HPLC (column: Gemini-NX C18, gradient: 0-5 min: A1/B1 95:5-90:10, 5-10 min: 90:10-78:22, 10-30 min: 78:22-50:50, t_R = 15 min) afforded **42** as white fluffy solid (27.6 mg, 74%). ^{1}H-NMR (600 MHz, DMSO-d_6): δ 0.81-0.93 (m, 6H), 1.37-1.95 (m, 20H), 1.95-2.06 (m, 1H), 2.44-2.48 (m, 3H), 2.52-2.54 (m, 1H), 2.66-2.74 (m, 1H), 2.84-2.91 (m, 1H), 3.05-3.14 (m, 4H), 3.54-3.62 (m, 2H), 3.76-3.86 (m, 1H), 4.17-4.26 (m, 1H), 4.27-4.38 (m, 2H), 4.38-4.45 (m, 1H), 4.48-4.59 (m, 1H), 6.57-6.63 (m, 2H), 6.63-7.15 (br s, 4H, interfering with the next listed signal), 6.99-7.01 (m, 2H), 7.15-7.58 (br s, 4H), 7.58-7.71 (m, 2H), 7.76-7.83 (m, 1H), 7.86-7.95 (m, 1H), 8.11-8.22 (m, 1H), 8.71-9.07 (m, 3H), 9.09-9.24 (m, 1H), 12.51 (br s, 1H). HRMS: *m/z* [*M*+3H]$^{3+}$ calcd. for [$C_{39}H_{67}N_{12}O_8$]$^{3+}$ 277.1729, found 277.1738. RP-HPLC (220 nm): 99% (t_R = 6.0 min, *k* = 6.9). $C_{39}H_{64}N_{12}O_8$ • $C_6H_3F_9O_6$ (829.02 + 342.07).

N^{α}-Methyl-Arg-Arg-Pro-Tyr-*α*-cyclopentyl-Gly-Leu tris(hydrotrifluoroacetate) (43).

Peptide **43** was synthesized according to the general procedure for SPPS using a H-Leu-2-ClTrt resin (loading 0.79 mmol/g) (40 mg, 0.0316 mmol). Purification by preparative RP-HPLC (column: Gemini-NX C18, gradient: 0-5 min: A1/B1 95:5-90:10, 5-10 min: 90:10-80:20, 10-30 min: 80:20-55:45, t_R = 17 min) afforded **43** as white fluffy solid (26.8 mg, 71%). ^{1}H-NMR (600 MHz, DMSO-d_6): δ 0.81-0.93 (m, 6H), 1.20-1.27 (m,

1H), 1.31-1.39 (m, 1H), 1.39-1.89 (m, 20H), 1.95-2.06 (m, 1H), 2.08-2.15 (m, 1H), 2.44-2.48 (m, 3H), 2.64-2.71 (m, 1H), 2.83-2.91 (m, 1H), 3.05-3.14 (m, 4H), 3.54-3.63 (m, 2H), 3.75-3.86 (m, 1H), 4.16-4.29 (m, 2H), 4.29-4.60 (m, 3H), 6.57-6.62 (m, 2H), 6.62-7.13 (br s, 4H, interfering with the next listed signal), 6.98-7.01 (m, 2H), 7.13-7.56 (br s, 4H), 7.56-7.71 (m, 2H), 7.79-7.93 (m, 2H), 8.16-8.26 (m, 1H), 8.72-9.03 (m, 3H), 9.09-9.26 (m, 1H), 12.49 (br s, 1H). HRMS: *m/z* $[M+3H]^{3+}$ calcd. for $[C_{40}H_{69}N_{12}O_8]^{3+}$ 281.8448, found 281.8457. RP-HPLC (220 nm): 99% (t_R = 6.8 min, k = 7.9). $C_{40}H_{66}N_{12}O_8 \cdot C_6H_3F_9O_6$ (843.04 + 342.07).

N^{α}-Methyl-Arg-Arg-Pro-Tyr-*α*-methyl-Leu-Leu tris(hydrotrifluoroacetate) (44).

Peptide **44** was synthesized according to the general procedure for SPPS using a H-Leu-2-ClTrt resin (loading 0.79 mmol/g) (40 mg, 0.0316 mmol). Purification by preparative RP-HPLC (column: Gemini-NX C18, gradient: 0-5 min: A1/B1 95:5-90:10, 5-10 min: 90:10-75:25, 10-30 min: 75:25-45:55, t_R = 16 min) afforded **44** as white fluffy solid (26.3 mg, 70%). ^{1}H-NMR (600 MHz, DMSO-d_6): δ 0.70-0.89 (m, 12H), 1.27-1.38 (m, 3H), 1.38-1.91 (m, 16H), 1.98-2.08 (m, 1H), 2.18-2.28 (m, 1H), 2.45-2.48 (m, 3H), 2.62-2.70 (m, 1H), 2.86-2.95 (m, 1H), 3.05-3.15 (m, 4H), 3.53-3.63 (m, 2H), 3.74-3.85 (m, 1H), 4.13-4.24 (m, 1H), 4.27-4.39 (m, 2H), 4.44-4.59 (m, 1H), 6.61-6.66 (m, 2H), 6.66-7.13 (br s, 4H, interfering with the next listed signal), 7.01-7.05 (m, 2H), 7.13-7.53 (br s, 4H), 7.53-7.72 (m, 3H), 8.04-8.12 (m, 1H), 8.31-8.37 (m, 1H), 8.74-9.03 (m, 3H), 9.14-9.23 (m, 1H), 12.55 (br s, 1H). HRMS: *m/z* $[M+3H]^{3+}$ calcd. for $[C_{40}H_{71}N_{12}O_8]^{3+}$ 282.5167, found 282.5175. RP-HPLC (220 nm): 99% (t_R = 8.3 min, k = 9.9). $C_{40}H_{68}N_{12}O_8 \cdot C_6H_3F_9O_6$ (845.06 + 342.07).

N^{α}-Methyl-Arg-Arg-Pro-Tyr-*α*-ethyl-D-Ala-Leu tris(hydrotrifluoroacetate) (45).

Peptide **45** was synthesized according to the general procedure for SPPS using a H-Leu-2-ClTrt resin (loading 0.79 mmol/g) (40 mg, 0.0316 mmol). Purification by preparative RP-HPLC (column: Gemini-NX C18, gradient: 0-5 min: A1/B1 95:5-90:10, 5-10 min: 90:10-80:20, 10-30 min: 80:20-60:40, t_R = 17 min) afforded **45** as white fluffy solid (26.3 mg, 72%). ^{1}H-NMR (600 MHz, DMSO-d_6): δ 0.58-0.72 (m, 3H), 0.80-0.91 (m, 6H), 1.35 (s, 3H), 1.40-1.89 (m, 15H), 1.98-2.07 (m, 1H), 2.12-2.21 (m, 1H), 2.45-2.48 (m, 3H), 2.65-2.75 (m, 1H), 2.88-2.95 (m, 1H), 3.05-3.18 (m, 4H), 3.49-3.68 (m, 2H), 3.70-3.87 (m, 1H), 4.19-4.39 (m, 3H), 4.48-4.59 (m, 1H), 6.61-6.67 (m, 2H), 6.67-7.13 (br s, 4H, interfering with the next listed signal), 7.01-7.05 (m, 2H), 7.13-7.51 (br s, 4H), 7.51-7.74 (m, 3H), 7.82-7.97 (m, 1H), 8.12-8.22 (m, 1H), 8.73-9.05 (m, 3H), 9.14-9.25 (m, 1H), 12.51 (br s, 1H). HRMS: *m/z* $[M+3H]^{3+}$ calcd. for $[C_{38}H_{67}N_{12}O_8]^{3+}$ 273.1729, found 273.1738. RP-HPLC (220 nm): 98% (t_R = 6.4 min, k = 7.4). $C_{38}H_{64}N_{12}O_8 \cdot C_6H_3F_9O_6$ (817.01 + 342.07).

N^{α}-Methyl-Arg-Arg-Pro-Tyr-Ile-*β*-cyclopropyl-Ala tris(hydrotrifluoroacetate) (46).

Peptide **46** was synthesized on a 2-ClTrt-Cl resin (loading 1.6 mmol/g) (40 mg, 0.064 mmol, 1 equiv.). The resin was treated with a solution of Fmoc-*β*-cyclopropyl-Ala-OH (45.0 mg, 0.128 mmol, 2 equiv.) and DIPEA

(111.5 µL, 0.64 mmol, 10 equiv.) in CH_2Cl_2 (0.5 mL) at 35 °C for 15 h. MeOH (65 µL) and CH_2Cl_2 (100 µL) were added and shaking was continued at rt for 15 min. The liquid was removed by filtration and the resin was washed with CH_2Cl_2 (3 ×), MeOH (3 ×) and DMF/NMP (80:20 v/v) (4 ×). Fmoc-deprotection of *β*-cyclopropyl-Ala and further SPPS was performed according to the general procedure for SPPS (note: the amounts of Fmoc-amino acids and coupling reagents were calculated based on the assumption that the loading of the resin with *β*-cyclopropyl-Ala was 50% compared to the original loading, i.e. 0.8 mmol/g). Purification by preparative RP-HPLC (column: Gemini-NX C18, gradient: 0-5 min: A1/B1 95:5-90:10, 5-10 min: 90:10-80:20, 10-30 min: 80:20-55:45, t_R = 15 min) afforded **46** as white fluffy solid (27.4 mg, 37%). ^{1}H-NMR (600 MHz, DMSO-d_6): δ 0.01-0.17 (m, 2H), 0.32-0.44 (m, 2H), 0.71-0.89 (m, 7H), 1.00-1.10 (m, 1H), 1.37-1.90 (m, 15H), 1.94-2.06 (m, 1H), 2.43-2.48 (m, 3H), 2.63-2.73 (m, 1H), 2.83-2.91 (m, 1H), 3.04-3.14 (m, 4H), 3.54-3.63 (m, 2H), 3.75-4.86 (m, 1H), 4.18-4.27 (m, 2H), 4.27-4.39 (m, 1H), 4.39-4.59 (m, 2H), 6.57-6.64 (m, 2H), 6.64-7.13 (br s, 4H, interfering with the next listed signal), 6.99-7.01 (m, 2H), 7.13-7.56 (br s, 4H), 7.56-7.79 (m, 3H), 7.93 (d, 1H, *J* 8.0 Hz), 8.18-8.34 (m, 1H), 8.70-9.23 (m, 4H), 12.52 (br s, 1H). HRMS: *m/z* $[M+3H]^{3+}$ calcd. for $[C_{39}H_{67}N_{12}O_8]^{3+}$ 277.1729, found 277.1741. RP-HPLC (220 nm): 99% (t_R = 5.3 min, *k* = 6.0). $C_{39}H_{64}N_{12}O_8 \cdot C_6H_3F_9O_6$ (829.02 + 342.07).

N^{α}-Methyl-Arg-Arg-Pro-Tyr-Ile-*α*-methyl-Leu tris(hydrotrifluoroacetate) (47).

Peptide **47** was synthesized on a 2-ClTrt-Cl resin (loading 1.6 mmol/g) (40 mg, 0.064 mmol, 1 equiv.). The resin was treated with a solution of Fmoc-*α*-methyl-Leu-OH (47.0 mg, 0.128 mmol, 2 equiv.) and DIPEA (111.5 µL, 0.64 mmol, 10 equiv.) in CH_2Cl_2 (0.5 mL) at 35 °C for 15 h. MeOH (65 µL) was added and shaking was continued at rt for 15 min. The liquid was removed by filtration and the resin was washed with CH_2Cl_2 (3 ×), MeOH (3 ×) and DMF/NMP (80:20 v/v) (4 ×). Fmoc-deprotection of *α*-methyl-Leu and further SPPS was performed according to the general procedure for SPPS (note: the amounts of Fmoc-amino acids and coupling reagents were calculated based on the assumption that the loading of the resin with *α*-methyl-Leu was 50% compared to the original loading, i.e. 0.8 mmol/g). Purification by preparative RP-HPLC (column: Gemini-NX C18, gradient: 0-5 min: A1/B1 95:5-90:10, 5-10 min: 90:10-75:25, 10-30 min: 75:25-45:55, t_R = 16 min) afforded **47** as white fluffy solid (11.7 mg, 15%). ^{1}H-NMR (600 MHz, DMSO-d_6): δ 0.77-0.87 (m, 12H), 1.00-1.09 (m, 1H), 1.35-1.87 (m, 19H), 1.96-2.05 (m, 1H), 2.45-2.48 (m, 3H), 2.63-2.74 (m, 1H), 2.83-2.92 (m, 1H), 3.06-3.16 (m, 4H), 3.52-3.64 (m, 2H), 3.74-3.88 (m, 1H), 4.16-4.22 (m, 1H), 4.28-4.38 (m, 1H), 4.39-4.59 (m, 2H), 6.59-6.64 (m, 2H), 6.64-7.14 (br s, 4H, interfering with the next listed signal), 7.00-7.03 (m, 2H), 7.14-7.57 (br s, 4H), 7.57-7.71 (m, 2H), 7.74-7.86 (m, 2H), 7.91 (d, 1H, *J* 8.0 Hz), 8.73-9.06 (m, 3H), 9.08-9.24 (m, 1H), 12.51 (br s, 1H). HRMS: *m/z* $[M+3H]^{3+}$ calcd. for $[C_{40}H_{71}N_{12}O_8]^{3+}$ 282.5167, found 282.5175. RP-HPLC (220 nm): > 99% (t_R = 8.1 min, *k* = 9.7). $C_{40}H_{68}N_{12}O_8 \cdot C_6H_3F_9O_6$ (845.06 + 342.07).

***N^α*-Methyl-Arg-Arg-Pro-*β,β*-dimethyl-Tyr-Ile-Leu tris(hydrotrifluoroacetate) (48) and N^α-Methyl-Arg-Arg-Pro-*β,β*-dimethyl-D-Tyr-Ile-Leu tris(hydrotrifluoroacetate) (49).**

Peptides **48** and **49** were synthesized according to the general procedure for SPPS using a H-Leu-2-ClTrt resin (loading 0.79 mmol/g) (40 mg, 0.0316 mmol). Purification by preparative RP-HPLC (column: Gemini-NX C18, gradient: 0-5 min: A1/B1 95:5-90:10, 5-10 min: 90:10-73:27, 10-30 min: 73:27-63:37, **48**: t_R = 14 min, **49**: t_R = 15 min) afforded the epimers **48** and **49** as white fluffy solids (**48**: 16.7 mg, 44%, **49**: 11.8 mg, 31%). **48**: ^{1}H-NMR (600 MHz, DMSO-d_6): δ 0.74-0.86 (m, 9H), 0.88-0.92 (m, 3H), 0.96-1.05 (m, 1H), 1.20-1.31 (m, 6H), 1.37-1.60 (m, 8H), 1.60-1.93 (m, 9H), 2.46-2.49 (m, 3H), 3.06-3.15 (m, 4H), 3.49-3.52 (m, 1H), 3.56-3.61 (m, 1H), 3.76-3.90 (m, 1H), 4.03-4.17 (m, 1H), 4.17-4.27 (m, 1H), 4.37-4.44 (m, 1H), 4.52-4.60 (m, 1H), 4.61-4.68 (m, 1H), 6.59-6.65 (m, 2H), 6.65-7.17 (br s, 4H, interfering with the next listed signal), 7.11-7.14 (m, 2H), 7.17-7.60 (br s, 4H, interfering with the next listed signal), 7.32-7.34 (m, 1H), 7.60-7.72 (m, 3H), 8.08-8.16 (m, 1H), 8.79-9.06 (m, 3H), 9.08-9.21 (m, 1H), 12.48 (br s, 1H). HRMS: *m/z* $[M+3H]^{3+}$ calcd. for $[C_{41}H_{73}N_{12}O_8]^{3+}$ 287.1886, found 287.1896. RP-HPLC (220 nm): 97% (t_R = 7.3 min, k = 8.6). $C_{41}H_{70}N_{12}O_8 \cdot C_6H_3F_9O_6$ (859.09 + 342.07). **49**: ^{1}H-NMR (600 MHz, DMSO-d_6): δ 0.72-0.81 (m, 6H), 0.82-0.86 (m, 3H), 0.87-0.97 (m, 4H), 1.20-1.25 (m, 3H), 1.25-1.35 (m, 5H), 1.40-1.60 (m, 8H), 1.60-1.83 (m, 7H), 2.46-2.48 (m, 3H), 3.04-3.14 (m, 4H), 3.45-3.48 (m, 1H), 3.53-3.57 (m, 1H), 3.73-3.81 (m, 1H), 4.08-4.19 (m, 2H), 4.36-4.43 (m, 1H), 4.46-4.55 (m, 1H), 4.86-4.92 (m, 1H), 6.58-6.65 (m, 2H), 6.65-7.11 (br s, 4H), 7.11-7.57 (br s, 4H, interfering with the next listed signal), 7.15-7.17 (m, 2H), 7.57-7.77 (m, 4H), 8.21 (d, 1H, *J* 7.1 Hz), 8.75-9.06 (m, 3H), 9.09-9.17 (m, 1H), 12.45 (br s, 1H). HRMS: *m/z* $[M+3H]^{3+}$ calcd. for $[C_{41}H_{73}N_{12}O_8]^{3+}$ 287.1886, found 287.1894. RP-HPLC (220 nm): 99% (t_R = 9.3 min, k = 11.2). $C_{41}H_{70}N_{12}O_8 \cdot C_6H_3F_9O_6$ (859.09 + 342.07).

***N^α*-Methyl-*N^ω*-[(4-aminobutyl)aminocarbonyl]Arg-Arg-Pro-*β,β*-dimethyl-Tyr-Ile-Leu tetrakis(hydrotrifluoroacetate) (50) and *N^α*-Methyl-*N^ω*-[(4-aminobutyl)aminocarbonyl]Arg-Arg-Pro-*β,β*-dimethyl-D-Tyr-Ile-Leu tetrakis(hydrotrifluoroacetate) (51).**

Peptides **50** and **51** were synthesized according to the general procedure for SPPS using a H-Leu-2-ClTrt resin (loading 0.79 mmol/g) (40 mg, 0.0316 mmol), with the following modification: after coupling of arginine building block **6** and Fmoc-deprotection, the resin was washed with CH_2Cl_2 (5 ×), a solution of 2-nitrobenzenesulfonylchloride (21.0 mg, 0.095 mmol) and collidine (20.9 µL, 0.158 mmol) in CH_2Cl_2 (0.75 mL) was added and the mixture was shaken at rt for 2 h. The resin was washed with DMF (5 ×), and a solution of MTBD (18.2 µL, 0.126 mmol) and methyl-4-nitrobenzenesulfonate (34.3 mg, 0.158 mmol) in DMF (0.9 mL) was added. After shaking at rt for 30 min, the resin was washed with DMF (3 ×) followed by the addition of a solution of DBU (23.6 µL, 0.158 mmol) and 2-mercaptoethanol (22.0 µL, 0.316 mmol) in DMF (0.75 mL) and shaking at rt for 30 min. The resin was washed with DMF (5 ×) followed by cleavage from the resin as

described in the general procedure for SPPS. Purification by preparative RP-HPLC (column: Gemini-NX C18, gradient: 0-5 min: A1/B1 85:15-80:20, 5-8 min: 80:20-73:27, 8-35 min: 73:27-70:30, **50**: t_R = 11 min, **51**: t_R = 12 min) afforded the epimers **50** and **51** as white fluffy solids (**50**: 11.5 mg, 25%, **51**: 7.9 mg, 17%). **50**: ^{1}H-NMR (600 MHz, DMSO-d_6): δ 0.73-0.96 (m, 12H), 0.96-1.06 (m, 1H), 1.15-1.33 (m, 6H), 1.33-1.96 (m, 21H), 2.46-2.49 (m, 3H), 2.75-2.82 (m, 2H), 3.04-3.20 (m, 4H), 3.20-3.32 (m, 2H), 3.44-3.63 (m, 2H), 3.80-3.93 (m, 1H), 4.08-4.18 (m, 1H), 4.20-4.27 (m, 1H), 4.38-4.44 (m, 1H), 4.51-4.69 (m, 2H), 6.58-6.65 (m, 2H), 6.79-7.27 (br s, 2H, interfering with the next listed signal), 7.11-7.14 (m, 2H), 7.27-7.30 (m, 1H), 7.30-7.72 (br s, 2H, interfering with the next listed signal), 7.55-7.68 (m, 2H), 7.72-7.91 (m, 4H), 8.06-8.16 (m, 1H), 8.31-8.73 (m, 2H), 8.80-9.39 (m, 5H), 10.61-10.87 (m, 1H), 12.50 (br s, 1H). ^{13}C-NMR (151 MHz, DMSO-d_6): δ 10.9, 15.1, 21.1, 23.0, 23.4, 24.2, 24.2, 24.2, 24.4, 24.5, 24.5, 26.0, 26.9, 27.0, 28.1, 28.6, 31.1, 36.7, 38.5, 38.6, 39.8, 40.1, 40.2, 40.4, 46.9, 50.0, 50.5, 56.6, 59.3, 59.8, 59.8, 114.3 (2 carbon atoms), 116.0 (TFA), 117.9 (TFA), 119.9, 127.4 (2 carbon atoms), 136.2, 153.9, 155.3, 156.9, 158.8 (q, *J* 32 Hz) (TFA), 167.0, 169.4, 169.4, 170.6, 170.7, 173.9. HRMS: *m/z* [*M*+H]$^+$ calcd. for [$C_{46}H_{81}N_{14}O_9$]$^+$ 973.6305, found 973.6309. RP-HPLC (220 nm): 97% (t_R = 7.1 min, *k* = 8.3). $C_{46}H_{80}N_{14}O_9$ • $C_8H_4F_{12}O_8$ (973.24 + 456.09). **51**: ^{1}H-NMR (600 MHz, DMSO-d_6): δ 0.71-0.96 (m, 13H), 1.18-1.37 (m, 8H), 1.42-1.86 (m, 19H), 2.44-2.49 (m, 3H), 2.75-2.84 (m, 2H), 3.02-3.18 (m, 4H), 3.20-3.29 (m, 2H), 3.43-3.50 (m, 1H), 3.50-3.59 (m, 1H), 3.76-3.84 (m, 1H), 4.08-4.19 (m, 2H), 4.35-4.43 (m, 1H), 4.47-4.56 (m, 1H), 4.84-4.90 (m, 1H), 6.60-6.64 (m, 2H), 6.64-7.24 (br s, 2H, interfering with the next listed signal), 7.14-7.17 (m, 2H), 7.24-7.55 (br s, 2H), 7.55-7.73 (m, 3H), 7.73-7.90 (m, 4H), 8.12-8.24 (m, 1H), 8.31-8.71 (m, 2H), 8.79-9.01 (m, 2H), 9.01-9.34 (m, 3H), 10.66-10.88 (m, 1H), 12.48 (br s, 1H). ^{13}C-NMR (151 MHz, DMSO-d_6): δ 11.1, 15.3, 21.5, 22.7, 23.4, 23.9, 24.0, 24.2, 24.3, 24.4, 24.5, 26.0, 26.3, 26.9, 28.1, 29.3, 31.1, 37.0, 38.5, 38.6, 39.7, 40.1, 40.4, 41.0, 46.7, 50.6, 50.6, 56.5, 59.2, 59.5, 59.8, 114.4 (2 carbon atoms), 116.0 (TFA), 117.9 (TFA), 119.9, 127.3 (2 carbon atoms), 136.8, 153.9, 155.4, 156.9, 158.8 (q, *J* 32 Hz) (TFA), 166.9, 168.9, 169.4, 170.2, 171.2, 173.9. HRMS: *m/z* [*M*+H]$^+$ calcd. for [$C_{46}H_{81}N_{14}O_9$]$^+$ 973.6305, found 973.6307. RP-HPLC (220 nm): 95% (t_R = 8.5 min, *k* = 10.2). $C_{46}H_{80}N_{14}O_9$ • $C_8H_4F_{12}O_8$ (973.24 + 456.09).

***N*^α^-Methyl-*N*^ω^-[(4-aminobutyl)aminocarbonyl]Arg-Arg-Pro-*β,β*-dimethyl-Tyr-*α*-*tert*-butyl-Gly-Leu tetrakis(hydrotrifluoroacetate) (52) and *N*^α^-Methyl-*N*^ω^-[(4-aminobutyl)aminocarbonyl]Arg-Arg-Pro-*β,β*-dimethyl-D-Tyr-*α*-*tert*-butyl-Gly-Leu tetrakis(hydrotrifluoroacetate) (53).**

Peptides **52** and **53** were synthesized according to the general procedure for SPPS using a H-Leu-2-ClTrt resin (loading 0.79 mmol/g) (25 mg, 0.01975 mmol), with the following modification: after coupling of arginine building block **6** and Fmoc-deprotection, the resin was washed with CH_2Cl_2 (5 ×), a solution of 2-nitrobenzenesulfonylchloride (13.1 mg, 0.059 mmol) and collidine (13.1 µL, 0.099 mmol) in CH_2Cl_2 (0.5 mL) was added and the mixture was shaken at rt for 2 h. The resin was washed with DMF (5 ×), and a solution of

MTBD (11.3 µL, 0.079 mmol) and methyl-4-nitrobenzenesulfonate (21.4 mg, 0.099 mmol) in DMF (0.6 mL) was added. After shaking at rt for 30 min, the resin was washed with DMF (3 ×) followed by the addition of a solution of DBU (14.7 µL, 0.099 mmol) and 2-mercaptoethanol (13.8 µL, 0.198 mmol) in DMF (0.5 mL) and shaking at rt for 30 min. The resin was washed with DMF (5 ×) followed by cleavage from the resin as described in the general procedure for SPPS. Purification by preparative RP-HPLC (column: Gemini-NX C18, gradient: 0-5 min: A1/B1 85:15-80:20, 5-8 min: 80:20-73:27, 8-35 min: 73:27-70:30, **52**: t_R = 11 min, **53**: t_R = 12 min) afforded the epimers **52** and **53** as white fluffy solids (**52**: 5.2 mg, 19%, **53**: 5.0 mg, 18%). **52**: ^{1}H-NMR (600 MHz, DMSO-d_6): δ 0.79-0.94 (m, 15H), 1.19-1.32 (m, 6H), 1.44-1.84 (m, 18H), 1.86-1.95 (m, 1H), 2.47-2.49 (m, 3H), 2.76-2.81 (m, 2H), 3.07-3.14 (m, 4H), 3.24-3.28 (m, 2H), 3.50-3.52 (m, 1H), 3.56-3.60 (m, 1H), 3.78-3.85 (m, 1H), 4.18-4.29 (m, 2H), 4.40-4.45 (m, 1H), 4.53-4.61 (m, 1H), 4.68-4.74 (m, 1H), 6.57-6.65 (m, 2H), 6.70-7.09 (br s, 2H), 7.09-7.15 (m, 2H), 7.15-7.51 (br s, 2H, interfering with the next listed signal), 7.37-7.45 (m, 2H), 7.53-7.78 (m, 5H), 8.12-8.18 (m, 1H), 8.31-8.64 (m, 2H), 8.78-9.22 (m, 5H), 10.16-10.53 (m, 1H), 12.46 (br s, 1H). HRMS: *m/z* [*M*+H]$^+$ calcd. for $[C_{46}H_{81}N_{14}O_9]^+$ 973.6305, found 973.6303. RP-HPLC (220 nm): 98% (t_R = 7.1 min, *k* = 8.3). $C_{46}H_{80}N_{14}O_9 \cdot C_8H_4F_{12}O_8$ (973.24 + 456.09). **53**: ^{1}H-NMR (600 MHz, DMSO-d_6): δ 0.78-0.91 (m, 15H), 1.19-1.32 (m, 7H), 1.42-1.80 (m, 18H), 2.42-2.48 (m, 3H), 2.77-2.83 (m, 2H), 3.06-3.15 (m, 4H), 3.22-3.26 (m, 2H), 3.48-3.54 (m, 2H), 3.75-3.83 (m, 1H), 4.09-4.23 (m, 2H), 4.37-4.42 (m, 1H), 4.47-4.56 (m, 1H), 4.87-4.94 (m, 1H), 6.60-6.65 (m, 2H), 6.65-7.13 (br s, 2H), 7.13-7.19 (m, 2H), 7.19-7.53 (br s, 2H, interfering with the next listed signal), 7.44-7.49 (m, 1H), 7.53-7.87 (m, 6H), 8.16-8.26 (m, 1H), 8.33-8.65 (m, 2H), 8.70-9.21 (m, 5H), 10.29-10.63 (m, 1H), 12.44 (br s, 1H). HRMS: *m/z* [*M*+H]$^+$ calcd. for $[C_{46}H_{81}N_{14}O_9]^+$ 973.6305, found 973.6307. RP-HPLC (220 nm): 93% (t_R = 8.5 min, *k* = 10.2). $C_{46}H_{80}N_{14}O_9 \cdot C_8H_4F_{12}O_8$ (973.24 + 456.09).

N^{α}-Methyl-N^{ω}-{[4-(N-{2-[4,7,10-tris(carboxymethyl)-1,4,7,10-tetraazacyclododecan-1-yl]acetyl})aminobutyl]aminocarbonyl}Arg-Arg-Pro-*β,β*-dimethyl-Tyr-Ile-Leu tetrakis(hydrotrifluoroacetate) (54). Compound **54** was prepared from **50** (9.7 mg, 6.79 µmol) and **13** (5.54 mg, 6.79 µmol) according to the general procedure for DOTA-conjugation (modification: stirring of the mixture for 45 min instead for 30 min). Isolation of the protected intermediate: column: Gemini-NX C18, gradient: 0-5 min: A1/B1 90:10-80:20, 5-10 min: 80:20-72:28, 10-20 min: 72:28-60:40, t_R = 19 min. Purification of the product by preparative RP-HPLC (column: Gemini-NX C18, gradient: 0-5 min: A1/B1 90:10-80:20, 5-10 min: 80:20-72:28, 10-20 min: 72:28-60:40, t_R = 11 min) afforded **54** as white fluffy solid (9.4 mg, 76%). ^{1}H-NMR (600 MHz, DMSO-d_6): δ 0.73-0.95 (m, 12H), 0.96-1.04 (m, 1H), 1.19-1.30 (m, 6H), 1.36-1.94 (m, 21H), 2.47-2.49 (m, 3H), 3.06-3.25 (m, 17H), 3.56-3.96 (m, 18H), 4.10-4.13 (m, 1H), 4.21-4.25 (m, 1H), 4.39-4.44 (m, 1H), 4.53-4.59 (m, 1H), 4.61-4.67 (m, 1H), 6.59-6.64 (m, 2H), 6.64-7.17 (br s, 2H, interfering with the next listed signal), 7.10-7.14 (m, 2H), 7.17-7.57 (br s, 2H, interfering with the next listed signal), 7.30-7.34 (m, 1H), 7.57-7.71 (m, 2H), 8.07-

8.16 (m, 1H), 8.22-8.56 (m, 2H), 8.77-9.46 (m, 5H), 11.28-13.36 (m, 2H). 6 exchangeable protons (NH, OH) of the presumably 4-fold protonated molecule could not be identified. HRMS: *m/z* $[M+3H]^{3+}$ calcd. for $[C_{62}H_{109}N_{18}O_{16}]^{3+}$ 453.9417, found 453.9425. RP-HPLC (220 nm): 98% (t_R = 7.0 min, *k* = 8.2). $C_{62}H_{106}N_{18}O_{16}$ · $C_8H_4F_{12}O_8$ (1359.64 + 456.09).

***N^α^*-Methyl-*N^ω^*-{[4-(N-{2-[4,7,10-tris(carboxymethyl)-1,4,7,10-tetraazacyclododecan-1-yl]acetyl})aminobutyl]aminocarbonyl}Arg-Arg-Pro-*β,β*-dimethyl-Tyr-*α-tert*-butyl-Gly-Leu tetrakis(hydrotrifluoroacetate) (55).**

Compound **55** was prepared from **52** (7.5 mg, 5.25 µmol) and **13** (4.28 mg, 5.25 µmol) according to the general procedure for DOTA-conjugation (modification: stirring of the mixture for 45 min instead for 30 min). Isolation of the protected intermediate: column: Gemini-NX C18, gradient: 0-5 min: A1/B1 90:10-80:20, 5-10 min: 80:20-72:28, 10-20 min: 72:28-60:40, t_R = 19 min. Purification of the product by preparative RP-HPLC (column: Gemini-NX C18, gradient: 0-5 min: A1/B1 90:10-80:20, 5-10 min: 80:20-72:28, 10-20 min: 72:28-60:40, t_R = 11 min) afforded **55** as white fluffy solid (7.5 mg, 79%). ^{1}H-NMR (600 MHz, DMSO-d_6): δ 0.80-0.93 (m, 15H), 1.21-1.29 (m, 6H), 1.39-1.85 (m, 18H), 1.86-1.96 (m, 1H), 2.47-2.49 (m, 3H), 2.94-3.22 (m, 17H), 3.47-3.59 (m, 5H), 3.59-3.92 (m, 13H), 4.20-4.26 (m, 2H), 4.40-4.46 (m, 1H), 4.53-4.59 (m, 1H), 4.68-4.74 (m, 1H), 6.56-6.65 (m, 2H), 6.65-7.12 (br s, 2H), 7.12-7.15 (m, 2H), 7.15-7.60 (br s, 2H, interfering with the next listed signal), 7.37-7.45 (m, 2H), 7.60-7.70 (m, 1H), 8.12-8.19 (m, 1H), 8.25-8.58 (m, 2H), 8.78-9.49 (m, 5H), 11.65-13.27 (m, 2H). 6 exchangeable protons (NH, OH) of the presumably 4-fold protonated molecule could not be identified. HRMS: *m/z* $[M+3H]^{3+}$ calcd. for $[C_{62}H_{109}N_{18}O_{16}]^{3+}$ 453.9417, found 453.9428. RP-HPLC (220 nm): 98% (t_R = 7.0 min, *k* = 8.2). $C_{62}H_{106}N_{18}O_{16}$ · $C_8H_4F_{12}O_8$ (1359.64 + 456.09).

***N^α^*-Methyl-*N^ω^*-{[4-(N-{2-[gallium(III)-4,7,10-tris(carboxymethyl)-1,4,7,10-tetraazacyclododecan-1-yl]acetyl})aminobutyl]aminocarbonyl}Arg-Arg-Pro-*β,β*-dimethyl-Tyr-Ile-Leu tris(hydrotrifluoroacetate) (56).**

Compound **56** was prepared from **54** (3.5 mg, 1.93 µmol) according to the general procedure for the insertion of Ga^{3+}. Purification by preparative RP-HPLC (column: Gemini-NX C18, gradient: 0-5 min: A1/B1 90:10-80:20, 5-10 min: 80:20-72:28, 10-20 min: 72:28-60:40, t_R = 12 min) yielded **56** as white fluffy solid (3.3 mg, 97%). ^{1}H-NMR (600 MHz, DMSO-d_6): δ 0.73-0.86 (m, 9H), 0.88-0.93 (m, 3H), 0.96-1.03 (m, 1H), 1.21-1.30 (m, 6H), 1.36-1.85 (m, 20H), 1.85-1.95 (m, 1H), 2.47-2.48 (m, 3H), 3.05-3.30 (m, 20H), 3.47-3.48 (m, 3H), 3.63-3.82 (m, 12H), 4.10-4.14 (m, 1H), 4.21-4.26 (m, 1H), 4.38-4.45 (m, 1H), 4.54-4.66 (m, 2H), 6.58-6.66 (m, 2H), 6.66-7.10 (br s, 2H), 7.10-7.15 (m, 2H), 7.15-7.48 (br s, 2H, interfering with the next listed signal), 7.31-7.35 (m, 1H), 7.48-7.56 (m, 1H), 7.56-7.67 (m, 2H), 8.07-8.16 (m, 1H), 8.30-8.63 (m, 3H), 8.89-9.15 (m, 4H), 9.97-10.25 (m, 1H), 12.48 (br s, 1H), 13.29 (br s, 1H). HRMS: *m/z* $[M+2H]^{2+}$ calcd. for

$[C_{62}H_{105}GaN_{18}O_{16}]^{2+}$ 713.3600, found 713.3605. RP-HPLC (220 nm): 98% (t_R = 7.4 min, k = 8.7). $C_{62}H_{103}GaN_{18}O_{16}$ • $C_6H_3F_9O_6$ (1426.34 + 342.07).

N^{α}-Methyl-N^{ω}-{[4-(N-{2-[gallium(III)-4,7,10-tris(carboxymethyl)-1,4,7,10-tetraazacyclododecan-1-yl]acetyl})aminobutyl]aminocarbonyl}Arg-Arg-Pro-*β,β*-dimethyl-Tyr-*α*-*tert*-butyl-Gly-Leu tris(hydrotrifluoroacetate) (57).

Compound **57** was prepared from **55** (1.8 mg, 0.991 µmol) according to the general procedure for the insertion of Ga^{3+}. Purification by preparative RP-HPLC (column: Gemini-NX C18, gradient: 0-5 min: A1/B1 90:10-80:20, 5-10 min: 80:20-72:28, 10-20 min: 72:28-60:40, t_R = 12 min) yielded **57** as white fluffy solid (1.6 mg, 91%). ^{1}H-NMR (600 MHz, DMSO-d_6): δ 0.77-0.98 (m, 15H), 1.19-1.29 (m, 6H), 1.37-1.83 (m, 18H), 1.87-1.95 (m, 1H), 2.46-2.48 (m, 3H), 3.02-3.17 (m, 9H), 3.25-3.32 (m, 11H), 3.63-3.70 (m, 7H), 3.70-3.85 (m, 8H), 4.20-4.27 (m, 2H), 4.40-4.46 (m, 1H), 4.53-4.60 (m, 1H), 4.67-4.74 (m, 1H), 6.58-6.63 (m, 2H), 6.63-7.10 (br s, 2H), 7.10-7.60 (br s, 2H, interfering with the next three listed signals), 7.12-7.15 (m, 2H), 7.37-7.45 (m, 2H), 7.49-7.54 (m, 1H), 7.60-7.65 (m, 1H), 8.15 (d, 1H, J 7.8 Hz), 8.25-8.61 (m, 3H), 8.86-9.17 (m, 4H), 10.03-10.28 (m, 1H), 12.46 (br s, 1H), 13.30 (br s, 1H). HRMS: m/z $[M+2H]^{2+}$ calcd. for $[C_{62}H_{105}GaN_{18}O_{16}]^{2+}$ 713.3600, found 713.3606. RP-HPLC (220 nm): 98% (t_R = 7.5 min, k = 8.9). $C_{62}H_{103}GaN_{18}O_{16}$ • $C_6H_3F_9O_6$ (1426.34 + 342.07).

3. RP-HPLC chromatograms of compounds 8, 9, 11, 12, 14-23, 25, 26, 28, 29 and 31-57.

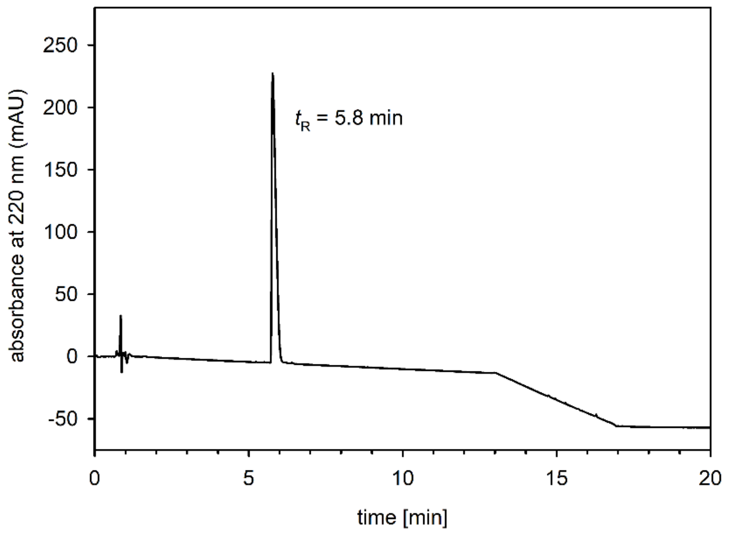

RP-HPLC analysis (purity control) of compound **8**

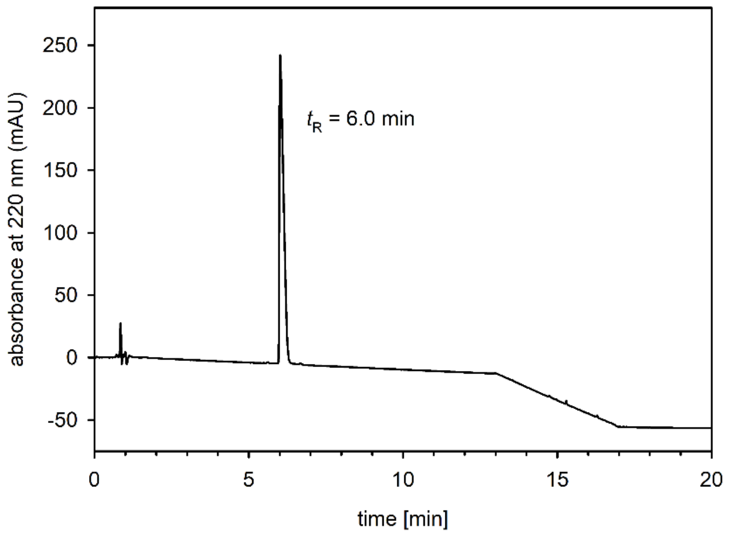

RP-HPLC analysis (purity control) of compound **9**

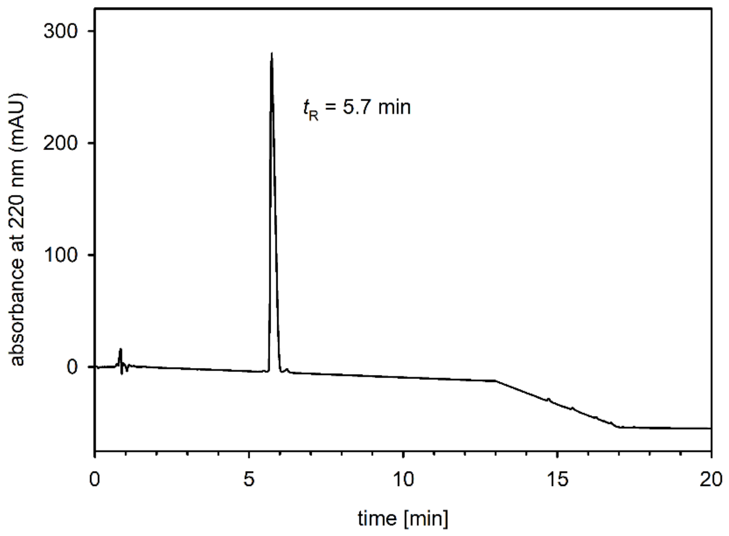

RP-HPLC analysis (purity control) of compound **11**

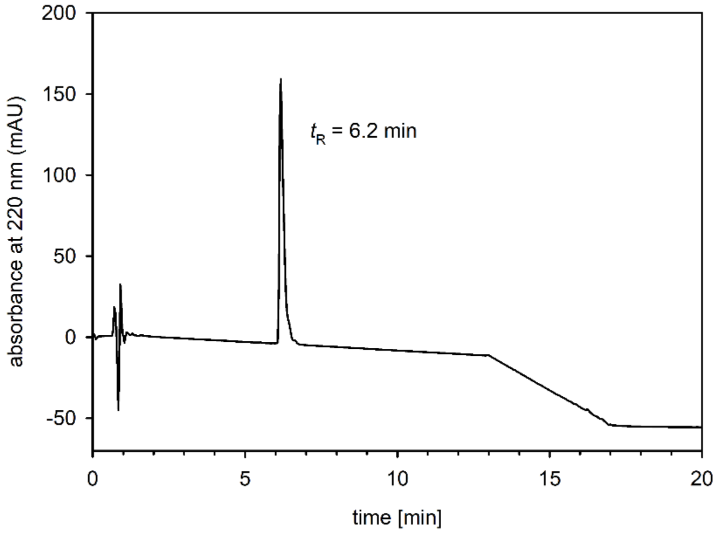

RP-HPLC analysis (purity control) of compound **12**

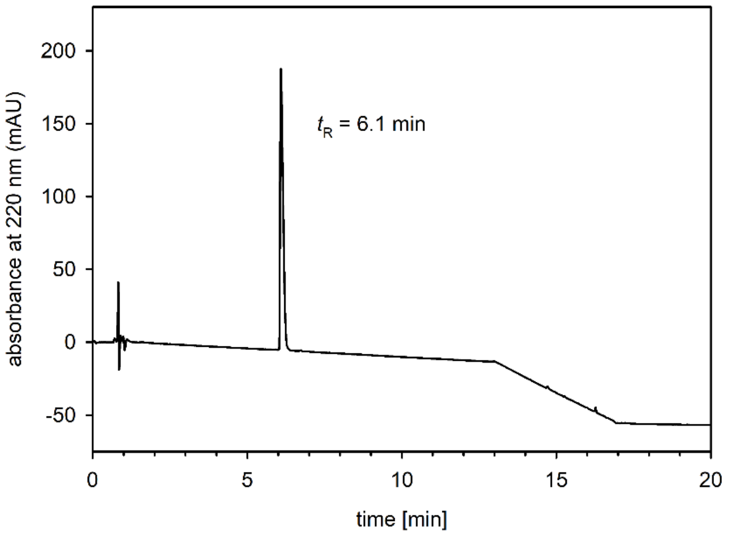

RP-HPLC analysis (purity control) of compound **14**

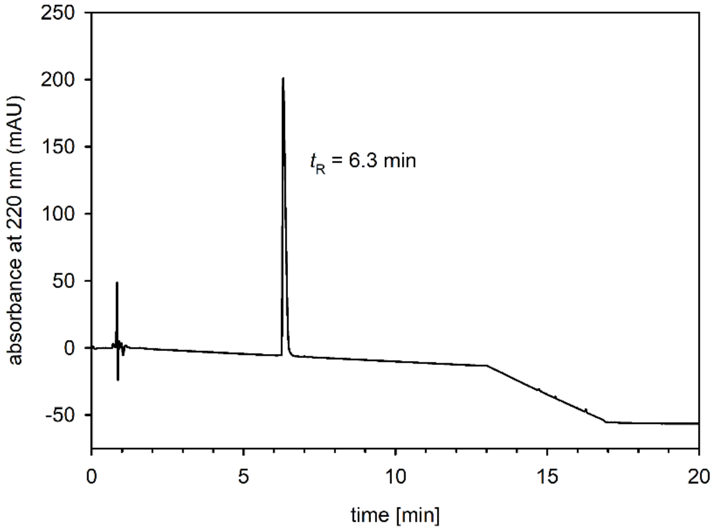

RP-HPLC analysis (purity control) of compound **15**

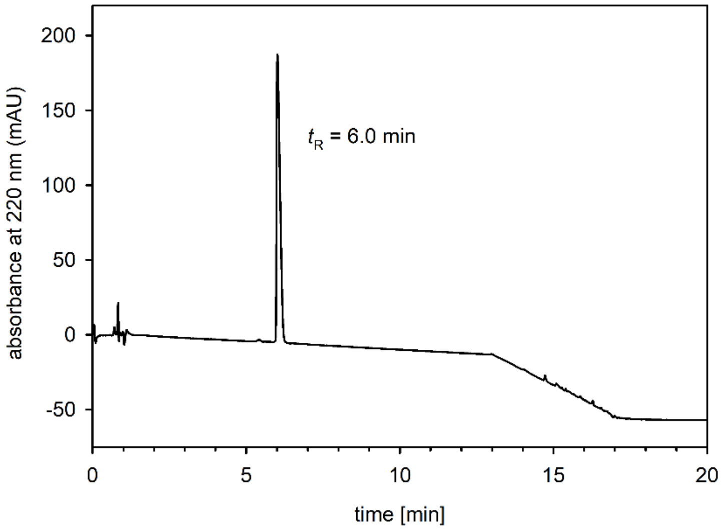

RP-HPLC analysis (purity control) of compound **16**

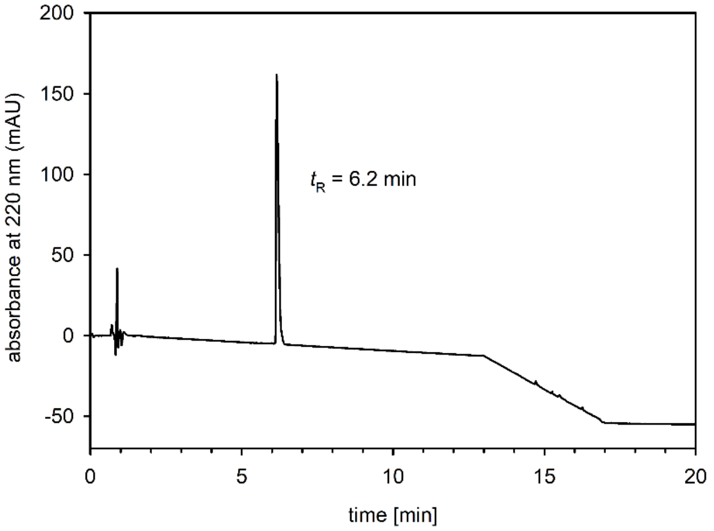

RP-HPLC analysis (purity control) of compound **17**

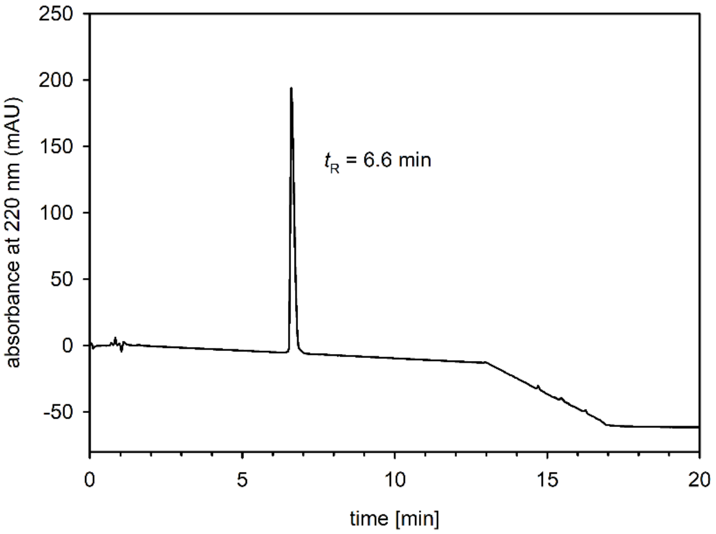

RP-HPLC analysis (purity control) of compound **18**

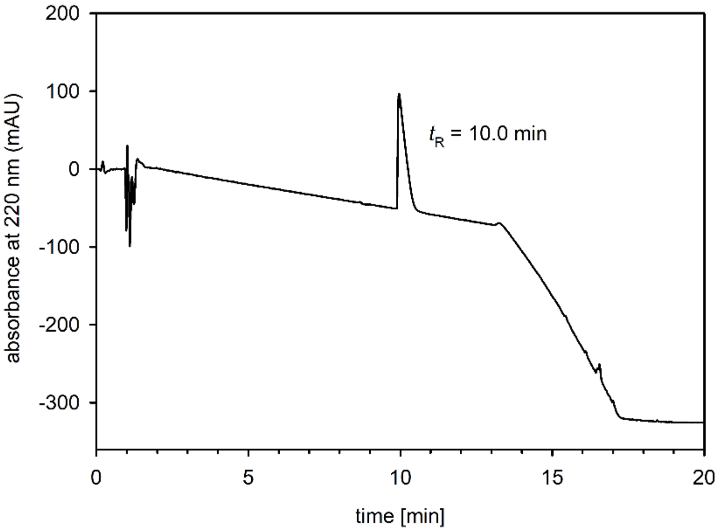

RP-HPLC analysis (purity control) of compound **19**

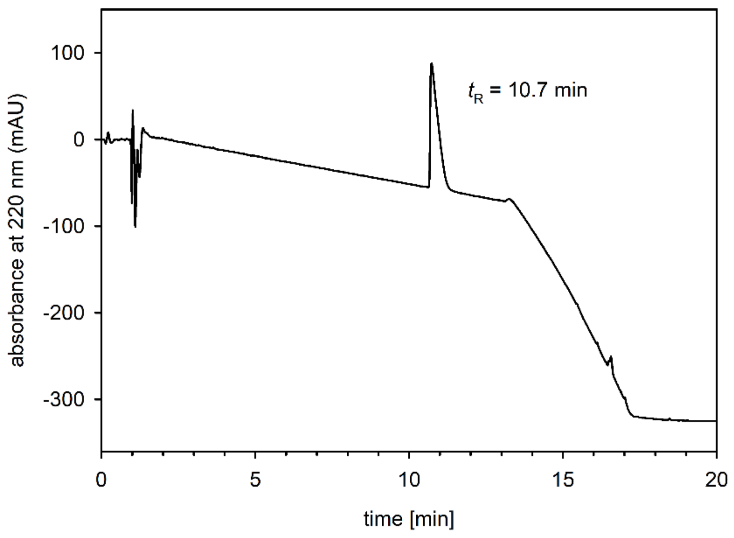

RP-HPLC analysis (purity control) of compound **20**

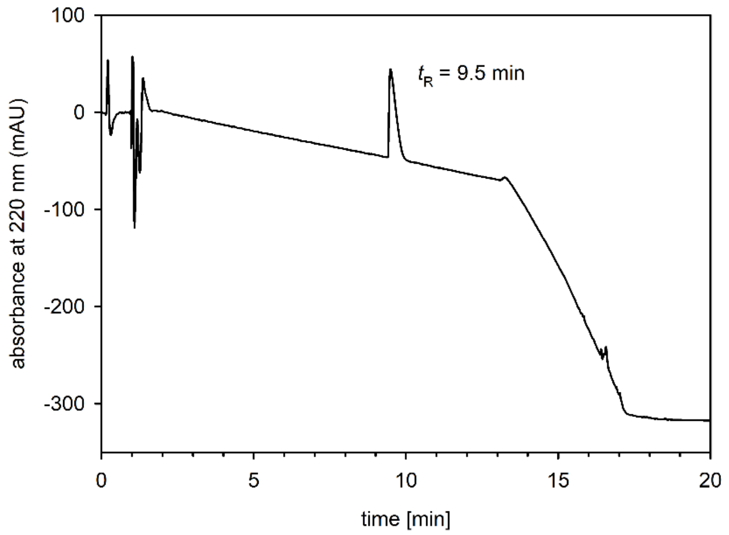

RP-HPLC analysis (purity control) of compound **21**

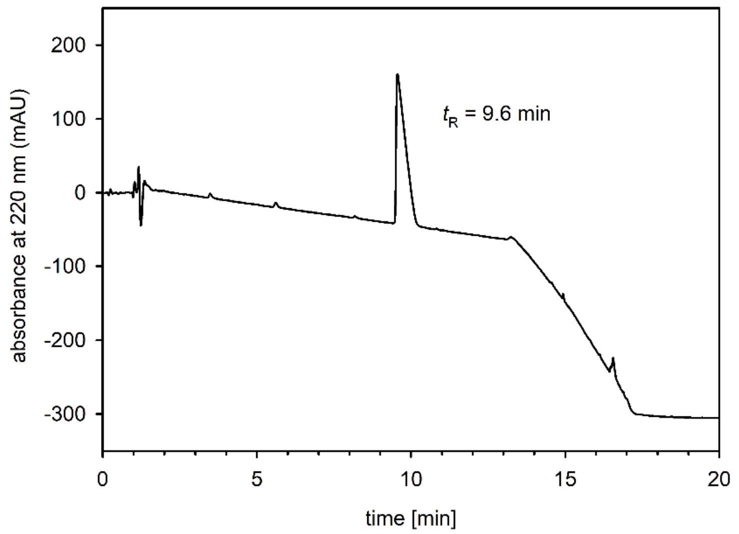

RP-HPLC analysis (purity control) of compound **22**

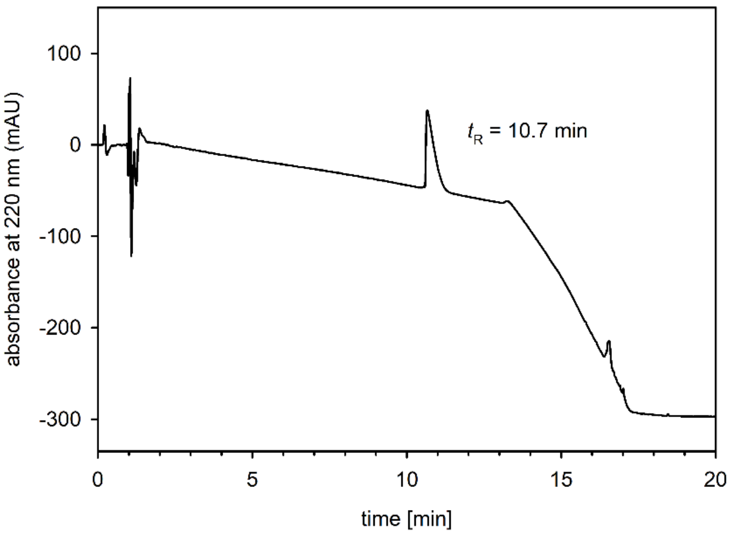

RP-HPLC analysis (purity control) of compound **23**

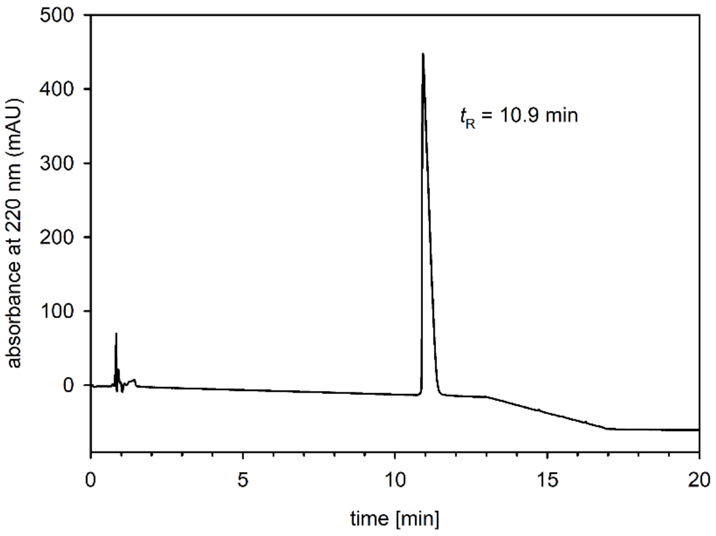

RP-HPLC analysis (purity control) of compound **25**

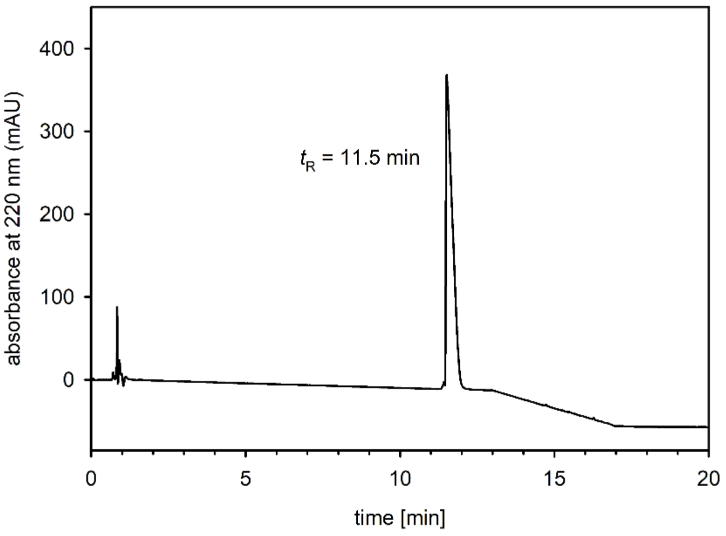

RP-HPLC analysis (purity control) of compound **26**

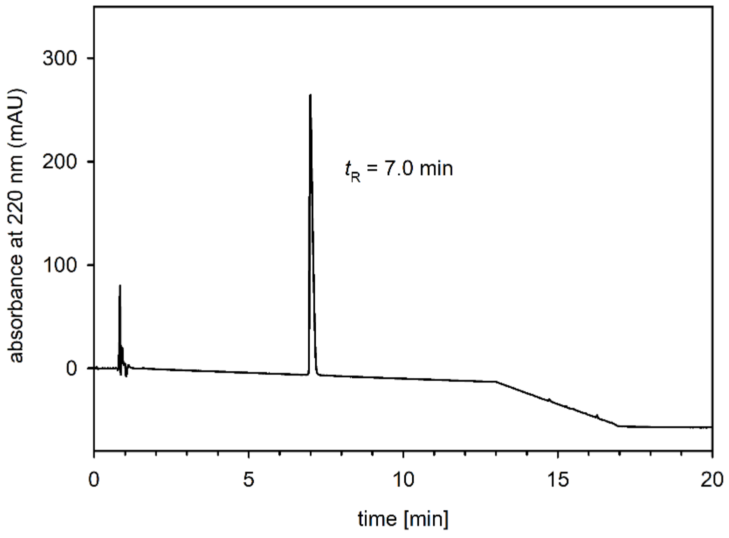

RP-HPLC analysis (purity control) of compound **28**

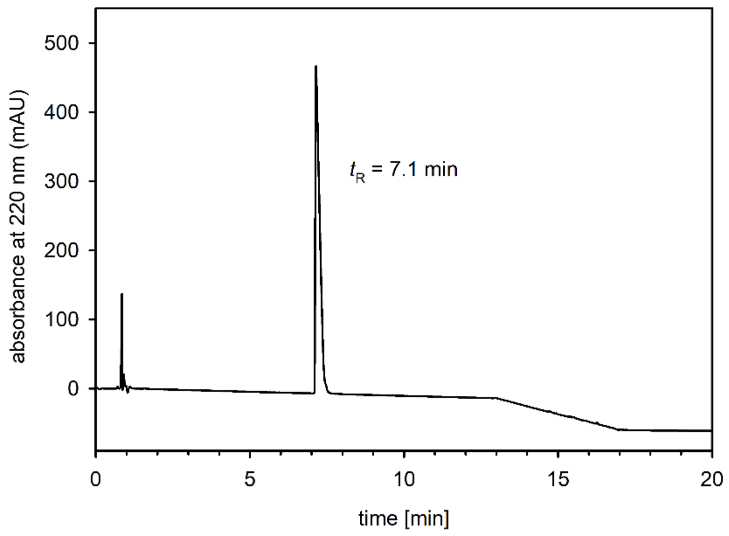

RP-HPLC analysis (purity control) of compound **29**

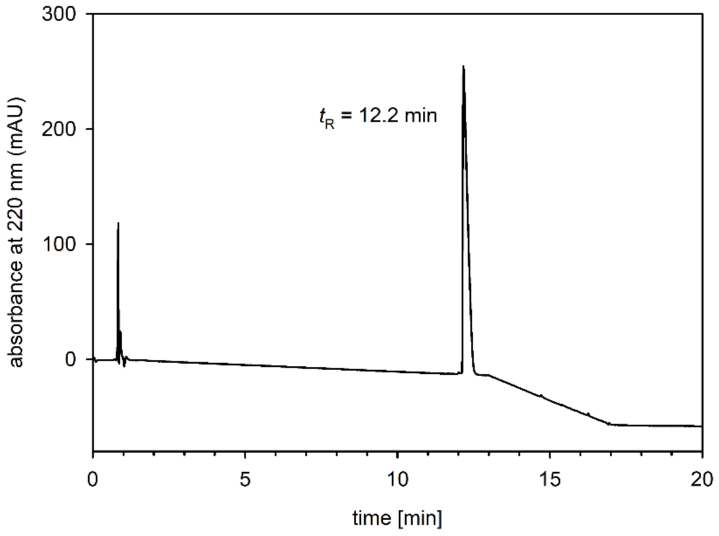

RP-HPLC analysis (purity control) of compound **31**

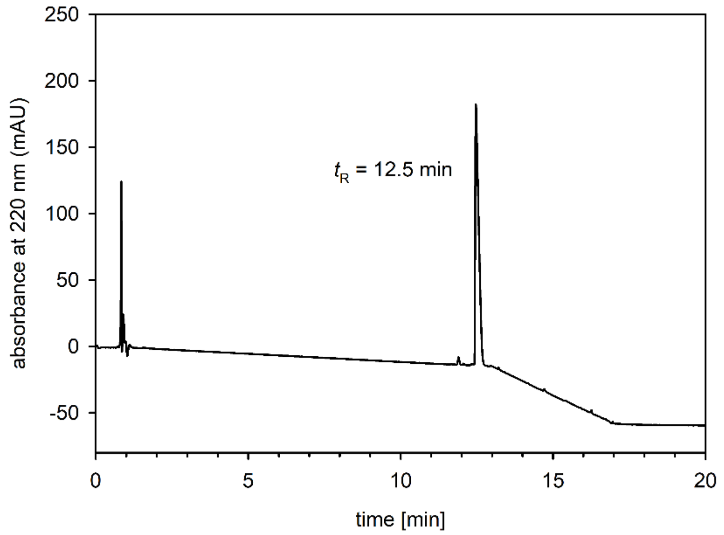

RP-HPLC analysis (purity control) of compound **32**

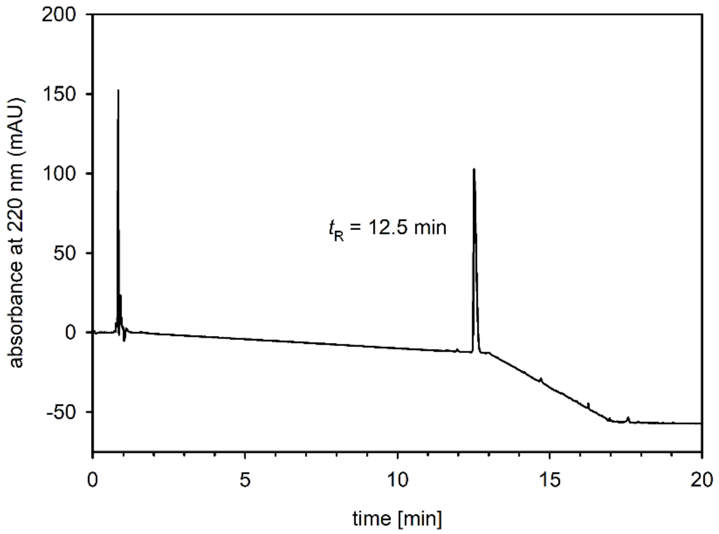

RP-HPLC analysis (purity control) of compound **33**

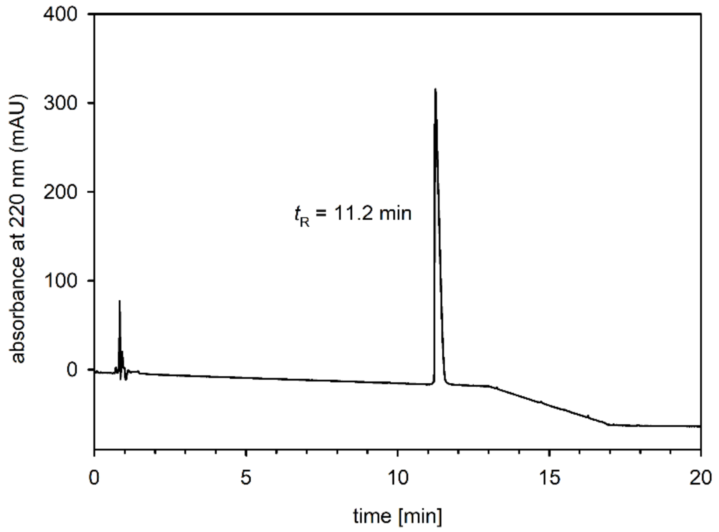

RP-HPLC analysis (purity control) of compound **34**

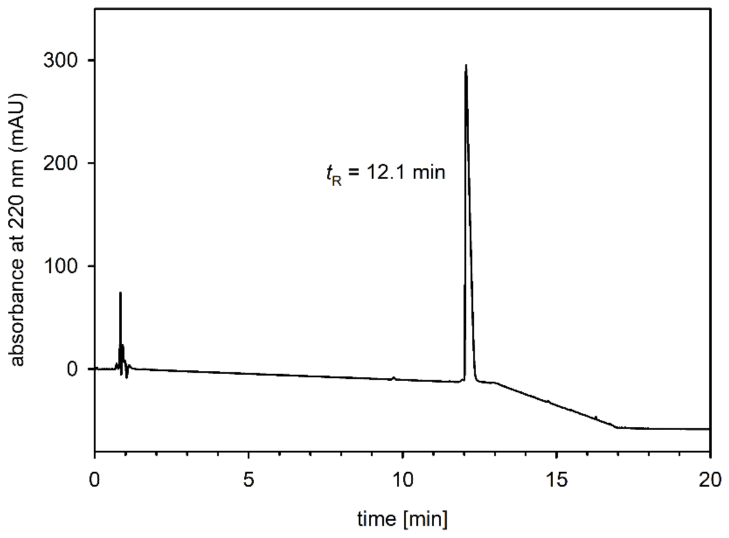

RP-HPLC analysis (purity control) of compound **35**

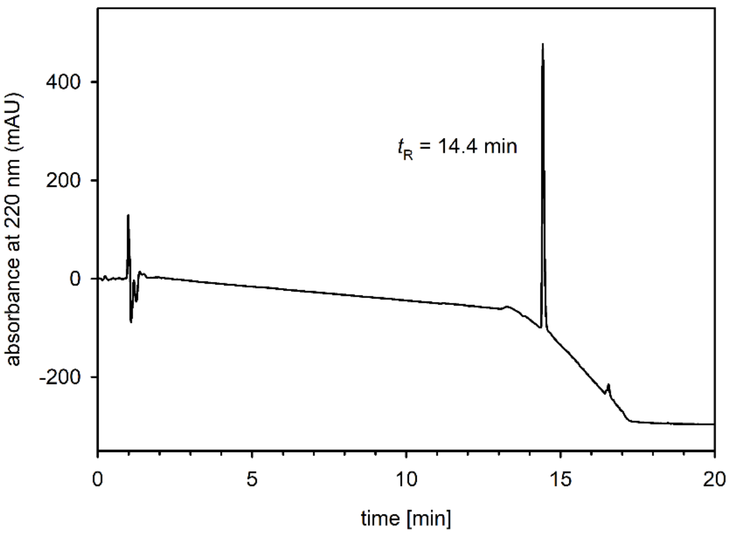

RP-HPLC analysis (purity control) of compound **36**

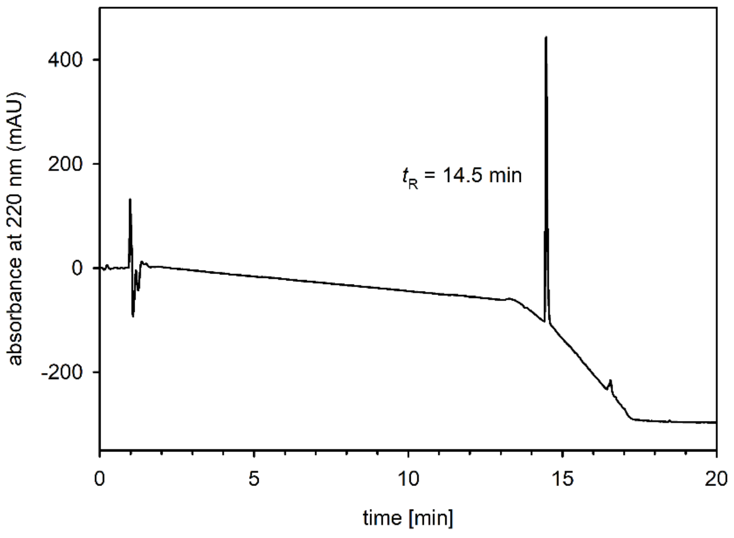

RP-HPLC analysis (purity control) of compound **37**

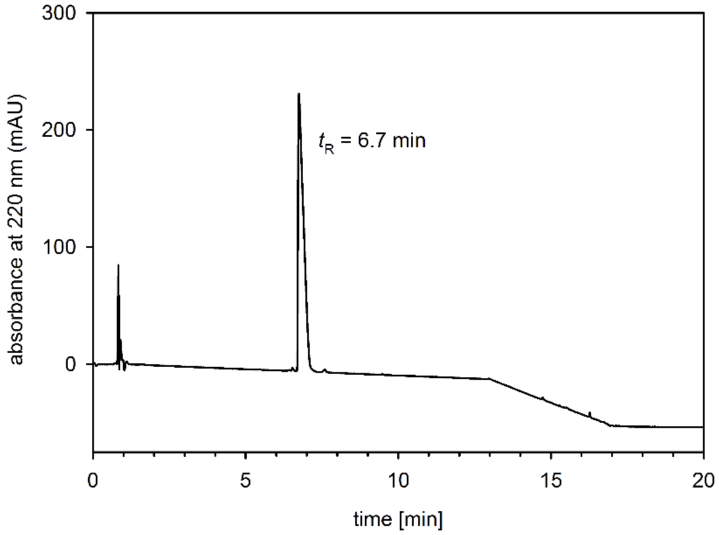

RP-HPLC analysis (purity control) of compound **38**

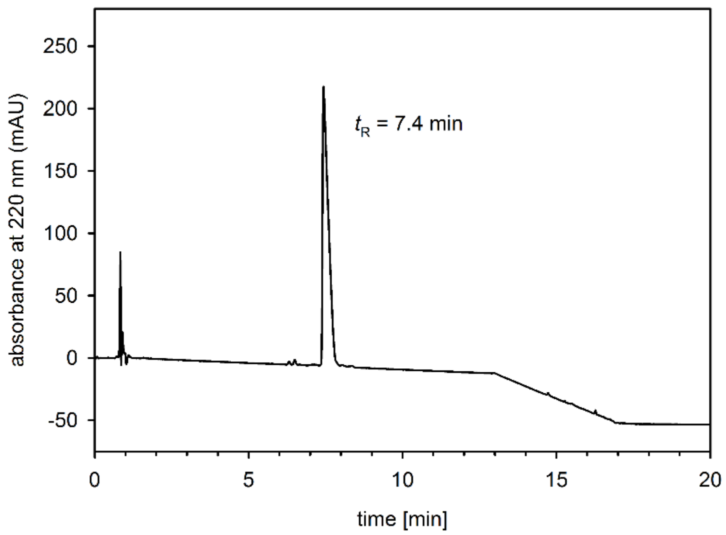

RP-HPLC analysis (purity control) of compound **39**

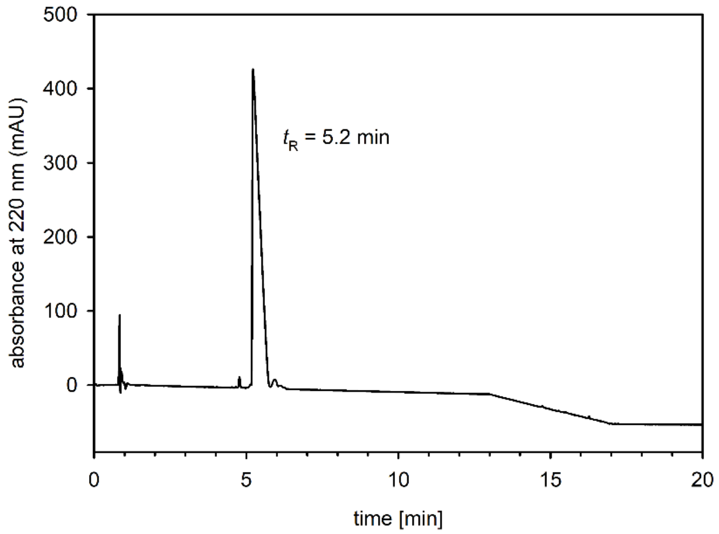

RP-HPLC analysis (purity control) of compound **40**

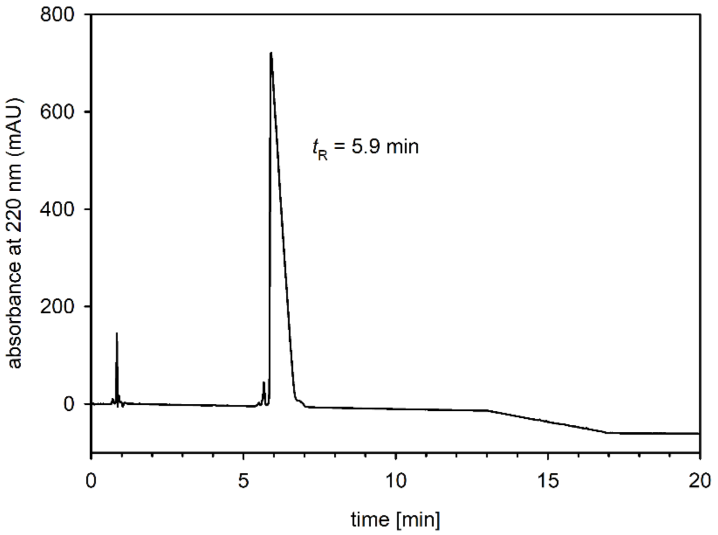

RP-HPLC analysis (purity control) of compound **41**

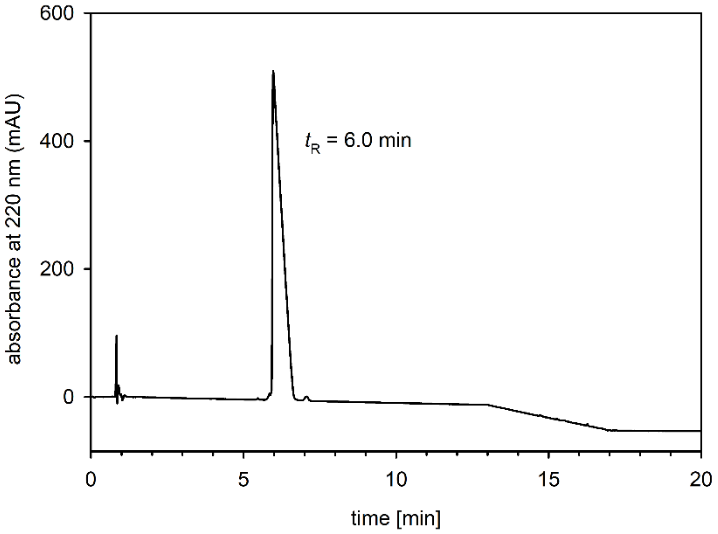

RP-HPLC analysis (purity control) of compound **42**

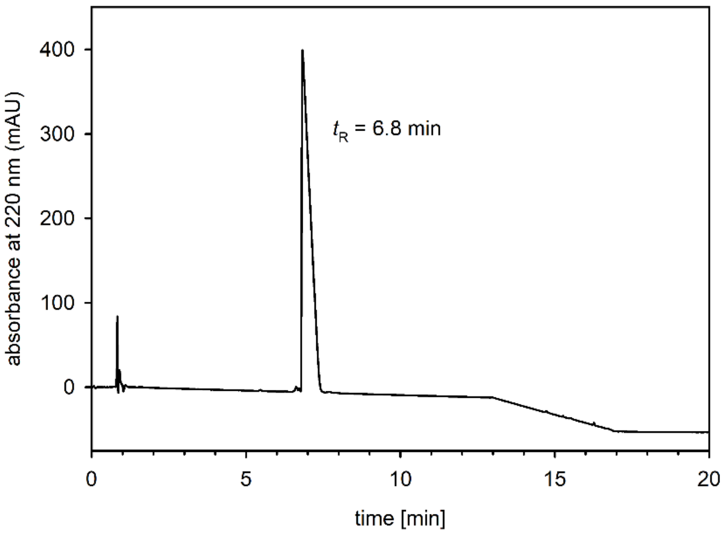

RP-HPLC analysis (purity control) of compound **43**

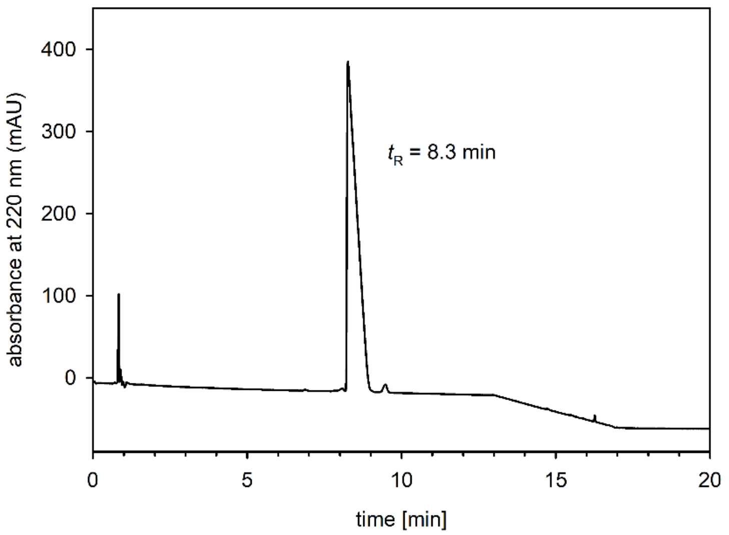

RP-HPLC analysis (purity control) of compound **44**

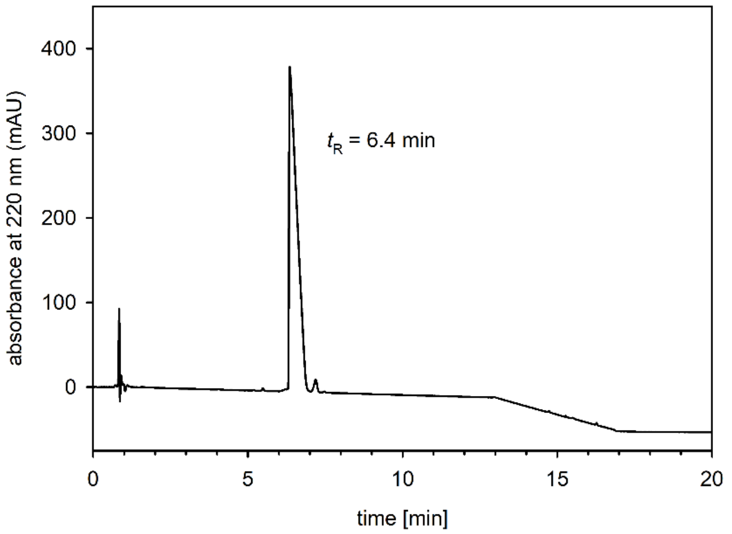

RP-HPLC analysis (purity control) of compound **45**

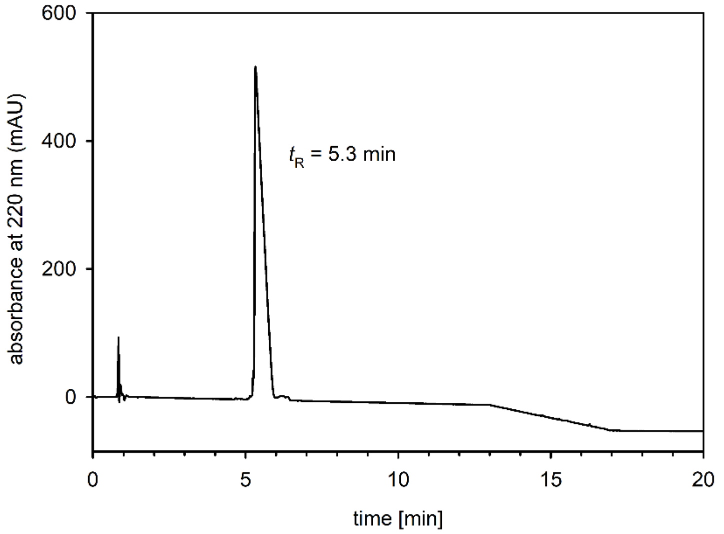

RP-HPLC analysis (purity control) of compound **46**

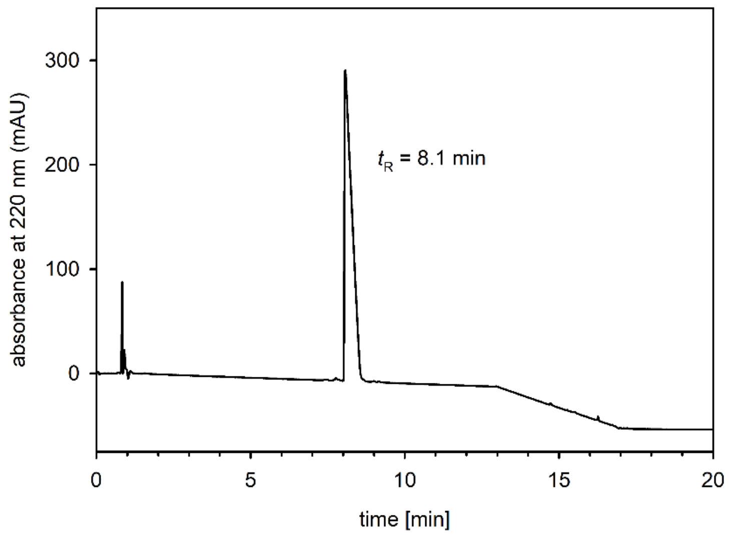

RP-HPLC analysis (purity control) of compound **47**

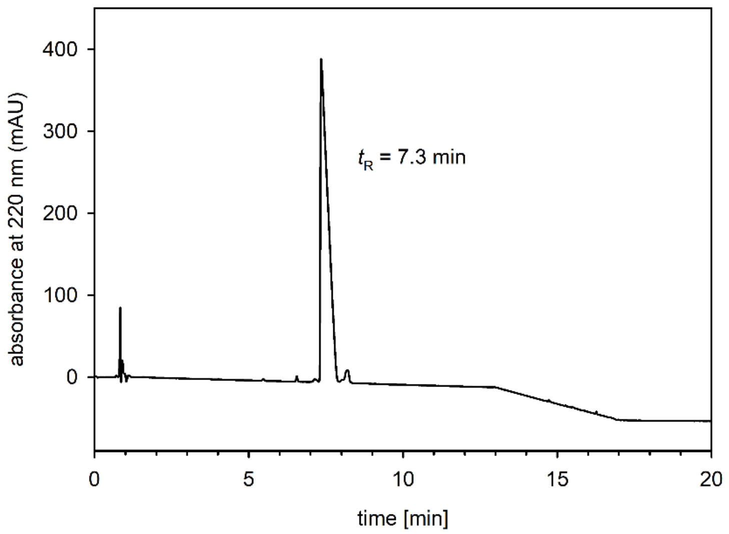

RP-HPLC analysis (purity control) of compound **48**

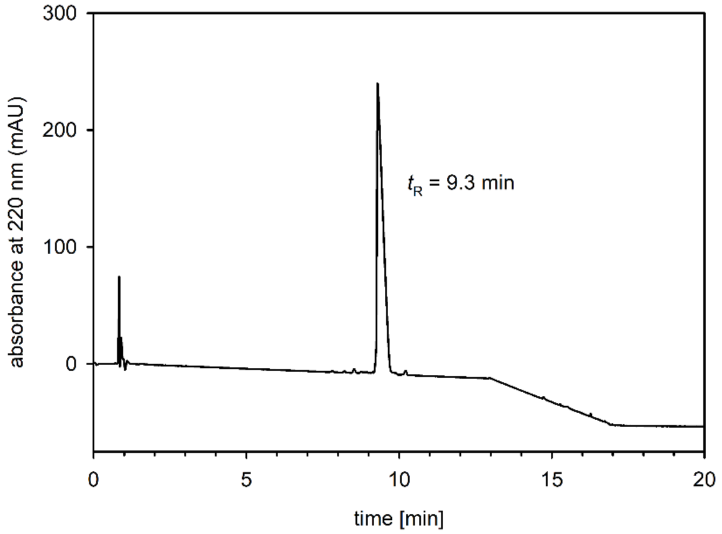

RP-HPLC analysis (purity control) of compound **49**

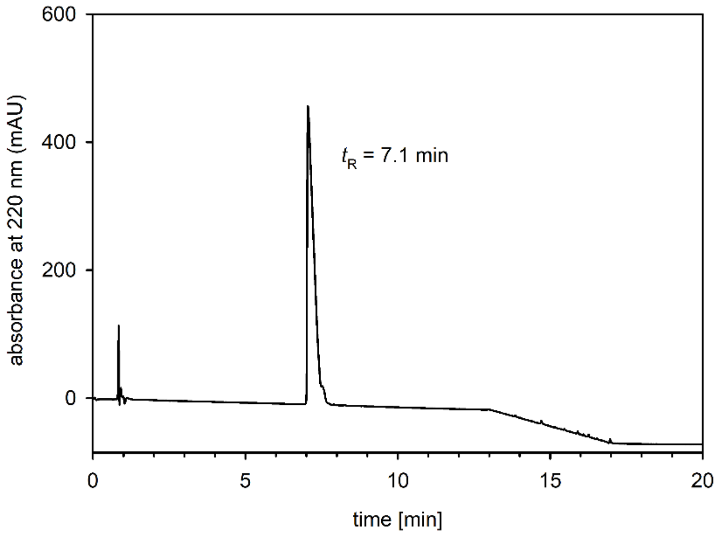

RP-HPLC analysis (purity control) of compound **50**

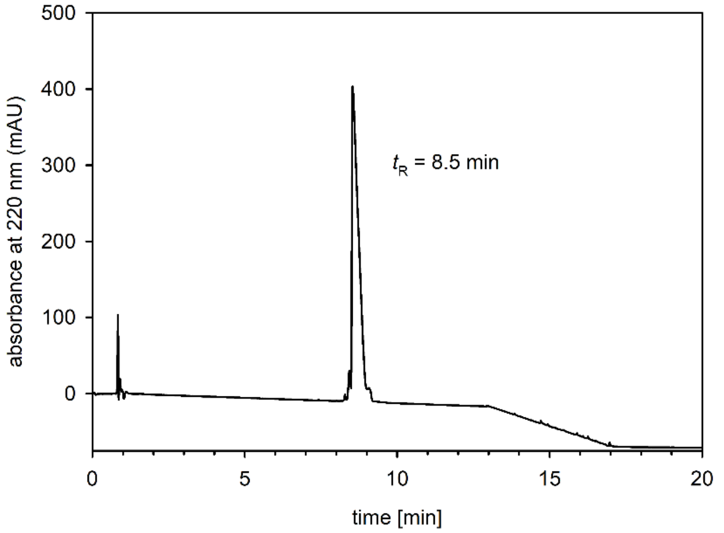

RP-HPLC analysis (purity control) of compound **51**

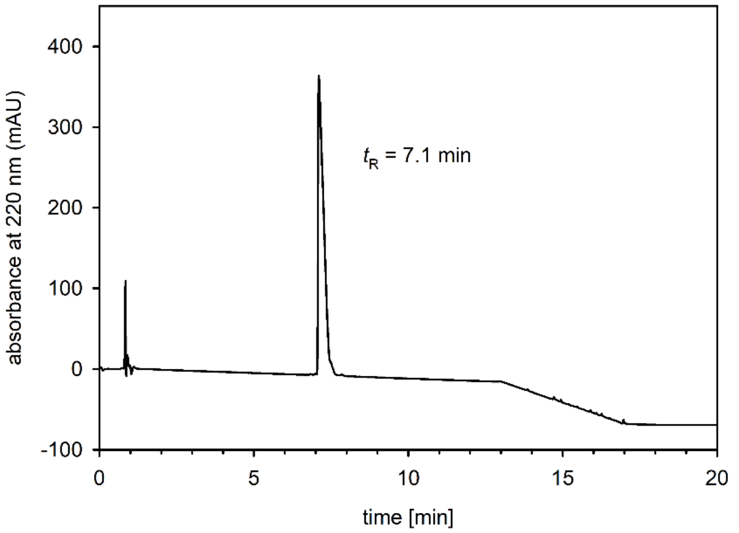

RP-HPLC analysis (purity control) of compound **52**

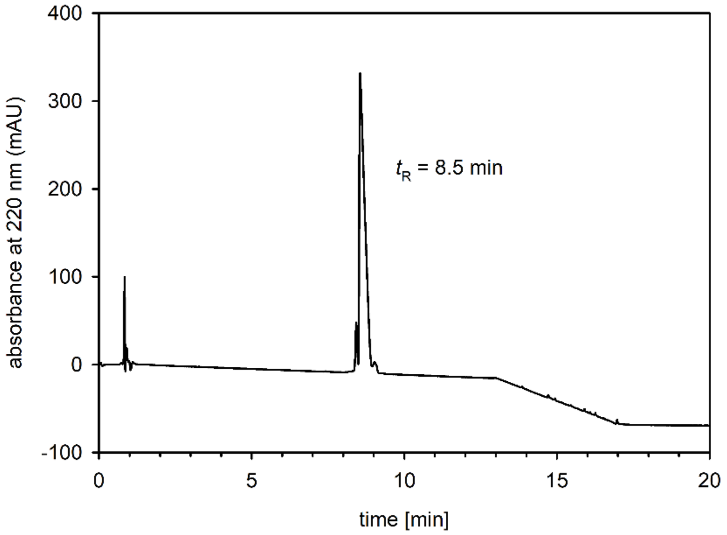

RP-HPLC analysis (purity control) of compound **53**

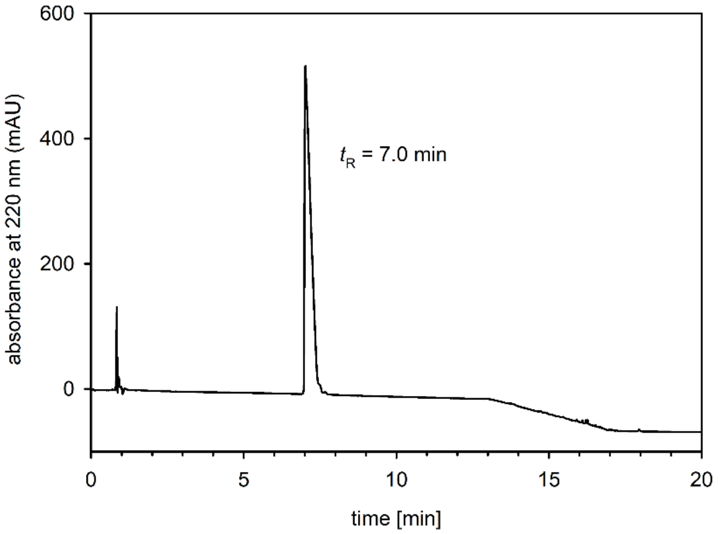

RP-HPLC analysis (purity control) of compound **54**

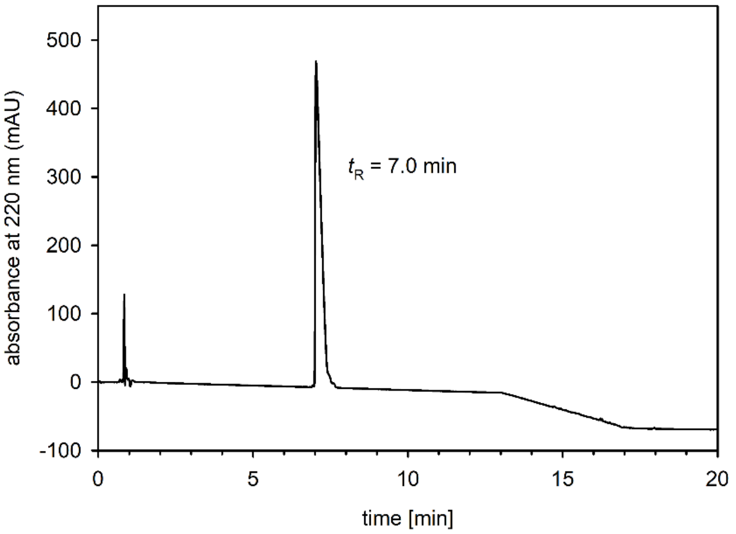

RP-HPLC analysis (purity control) of compound **55**

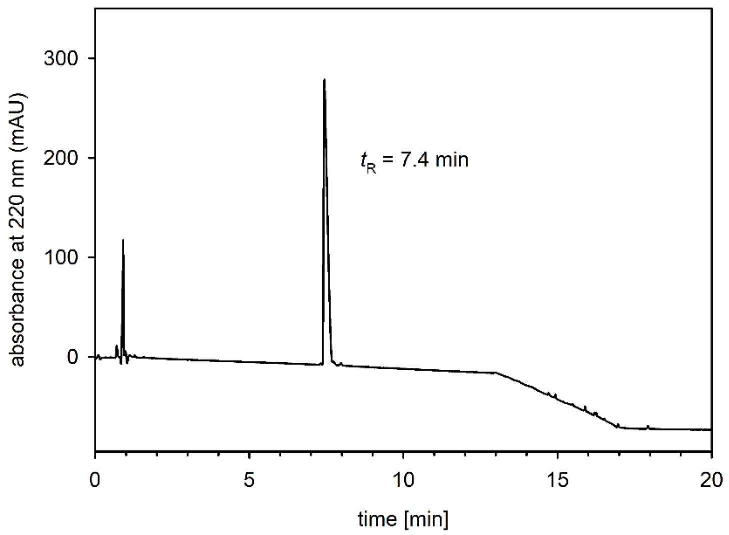

RP-HPLC analysis (purity control) of compound **56**

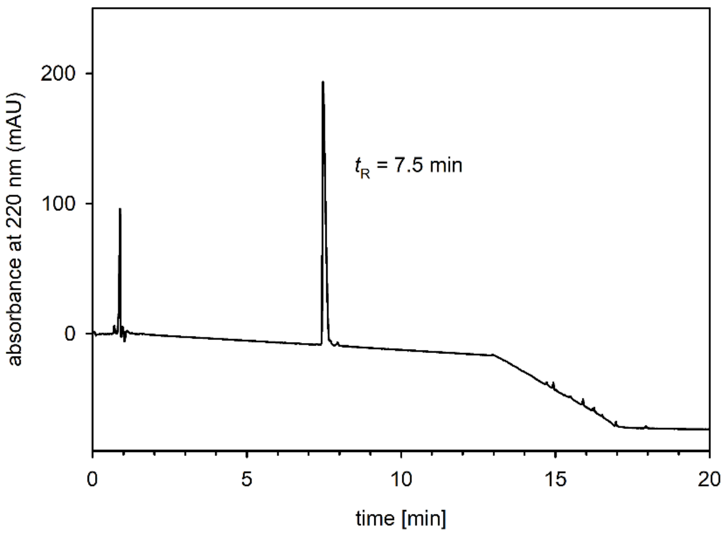

RP-HPLC analysis (purity control) of compound **57**

4. ^{1}H-NMR spectra of compounds 8, 9, 11, 12, 14-23, 25, 26, 28, 29 and 32-57, and ^{13}C-NMR spectra of compounds 50 and 51.

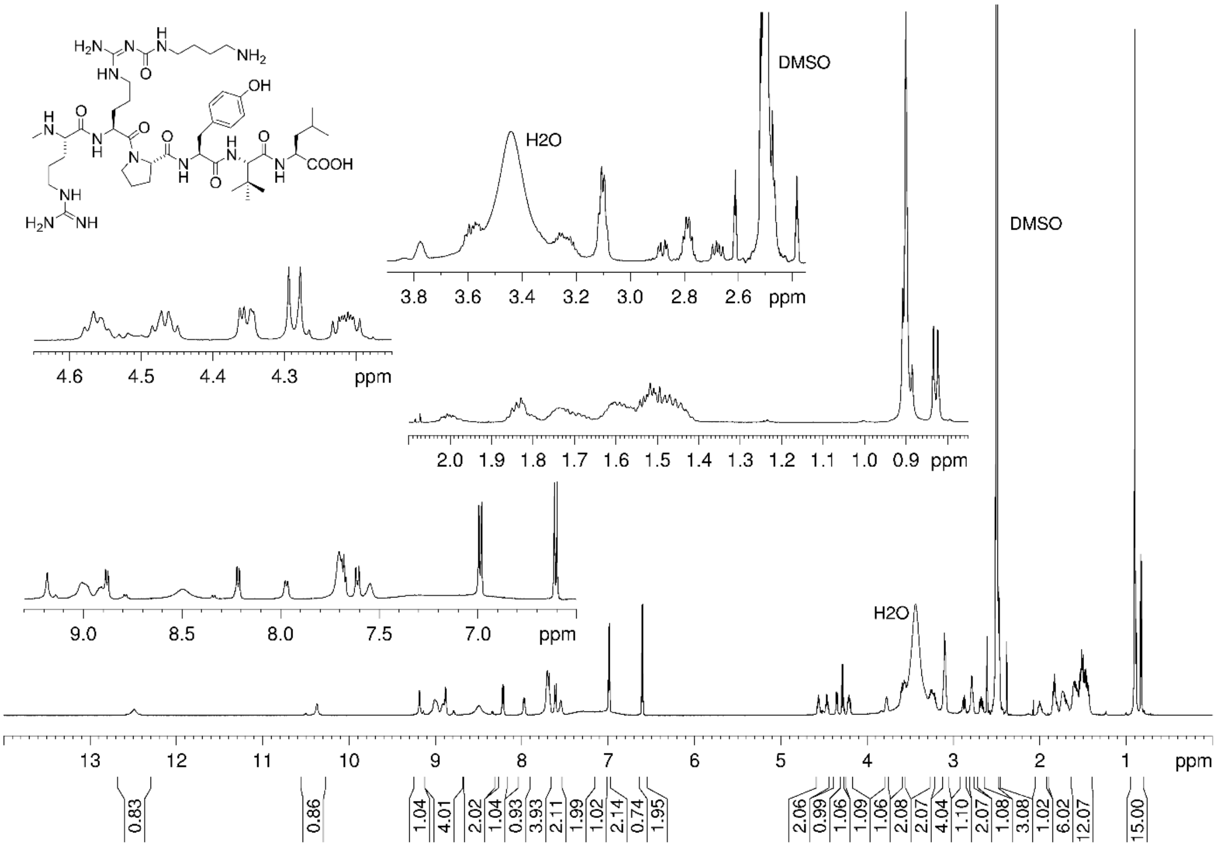

^{1}H-NMR spectrum (600 MHz, DMSO-d_6) of compound **8**

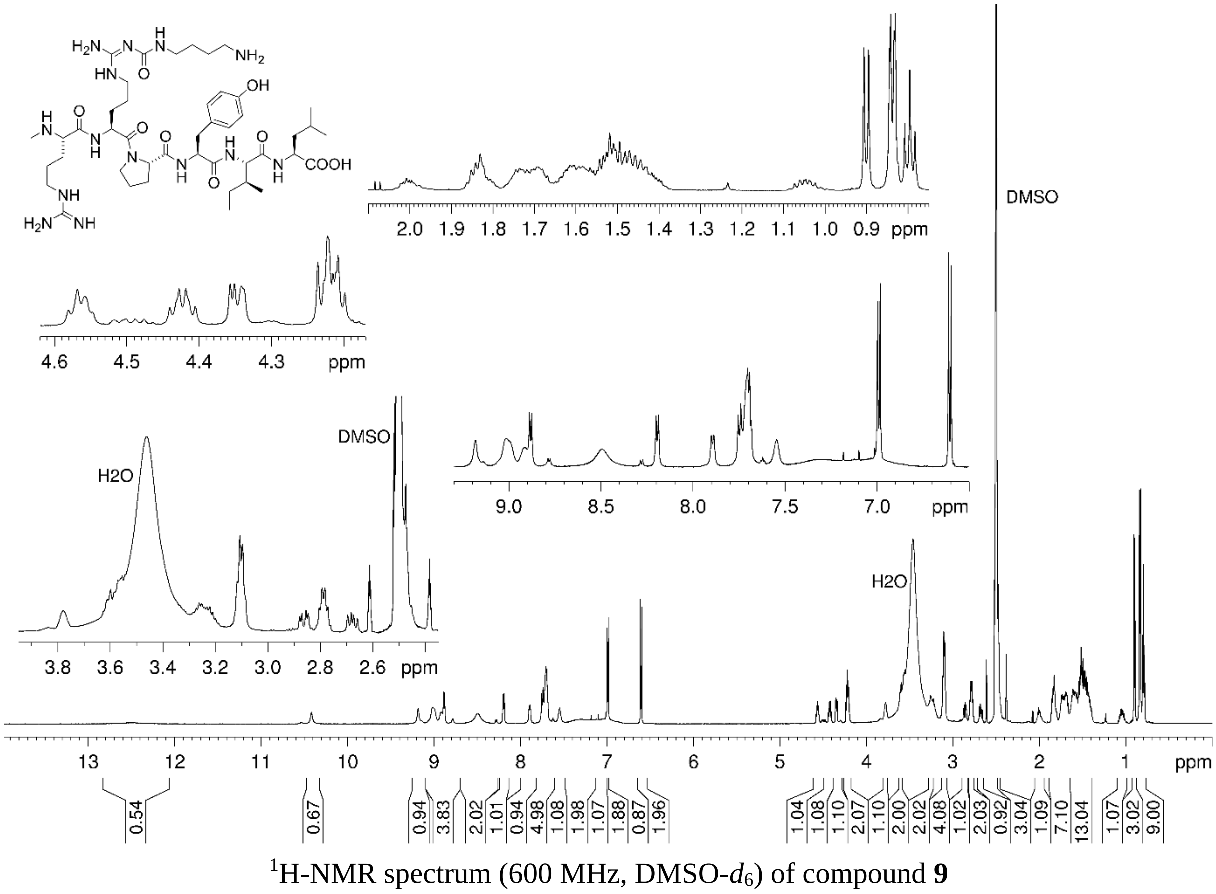

^{1}H-NMR spectrum (600 MHz, DMSO-d_6) of compound **9**

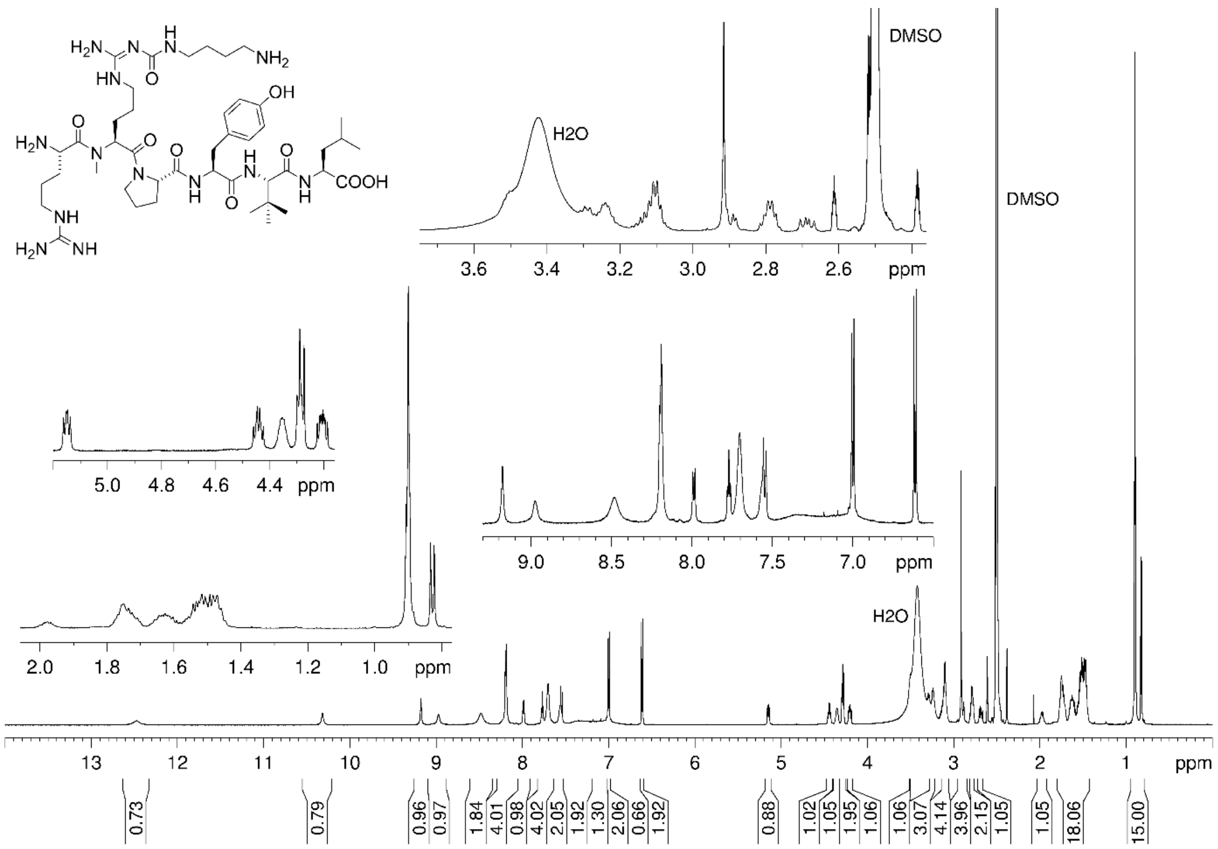

^{1}H-NMR spectrum (600 MHz, DMSO-d_6) of compound **11**

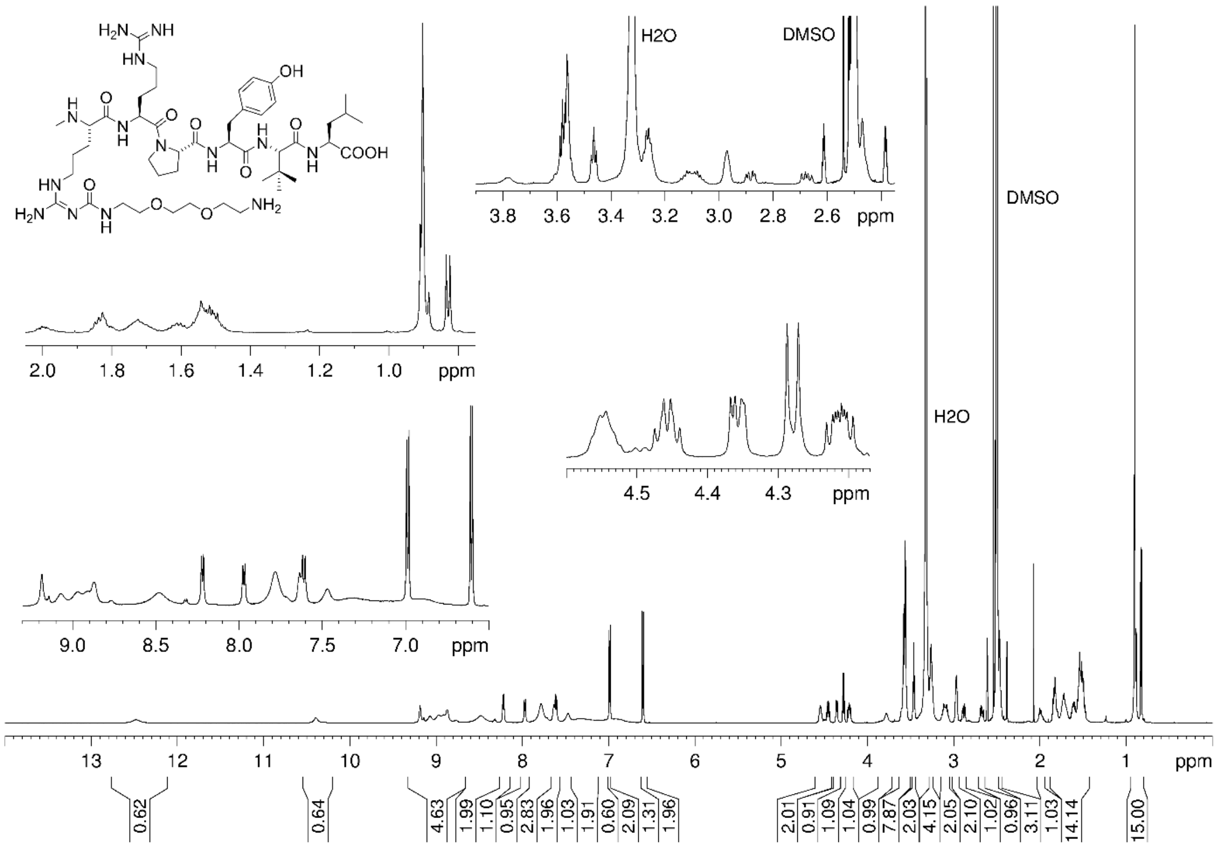

^{1}H-NMR spectrum (600 MHz, DMSO-d_6) of compound **12**

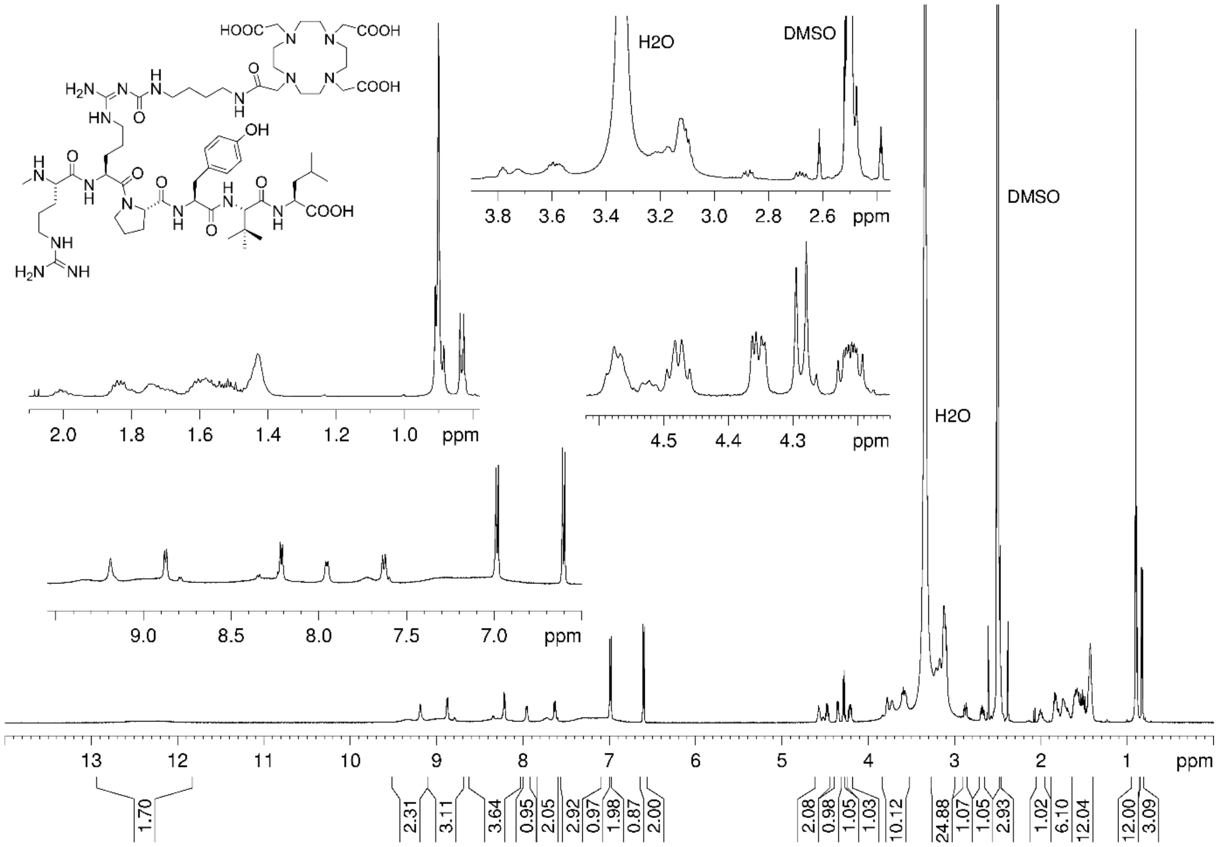

^{1}H-NMR spectrum (600 MHz, DMSO-d_6) of compound **14**

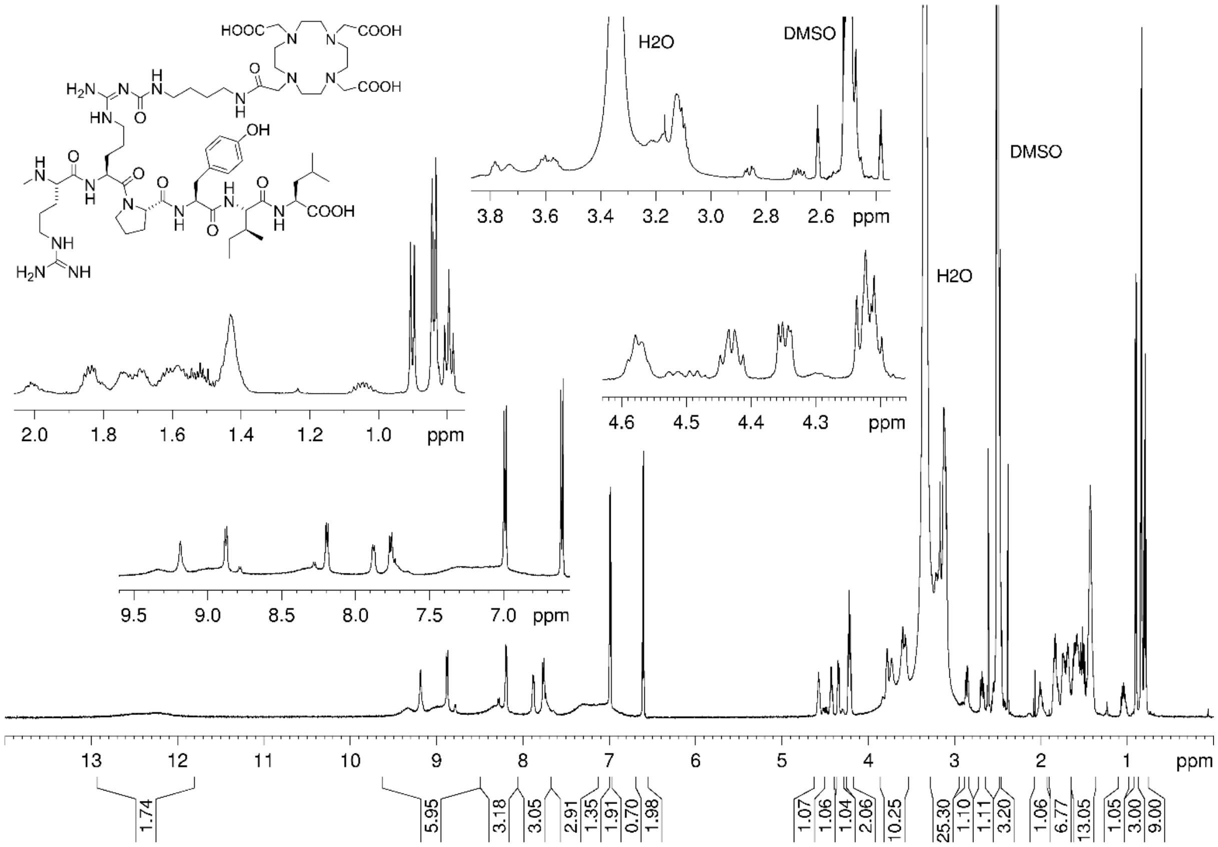

^{1}H-NMR spectrum (600 MHz, DMSO-d_6) of compound **15**

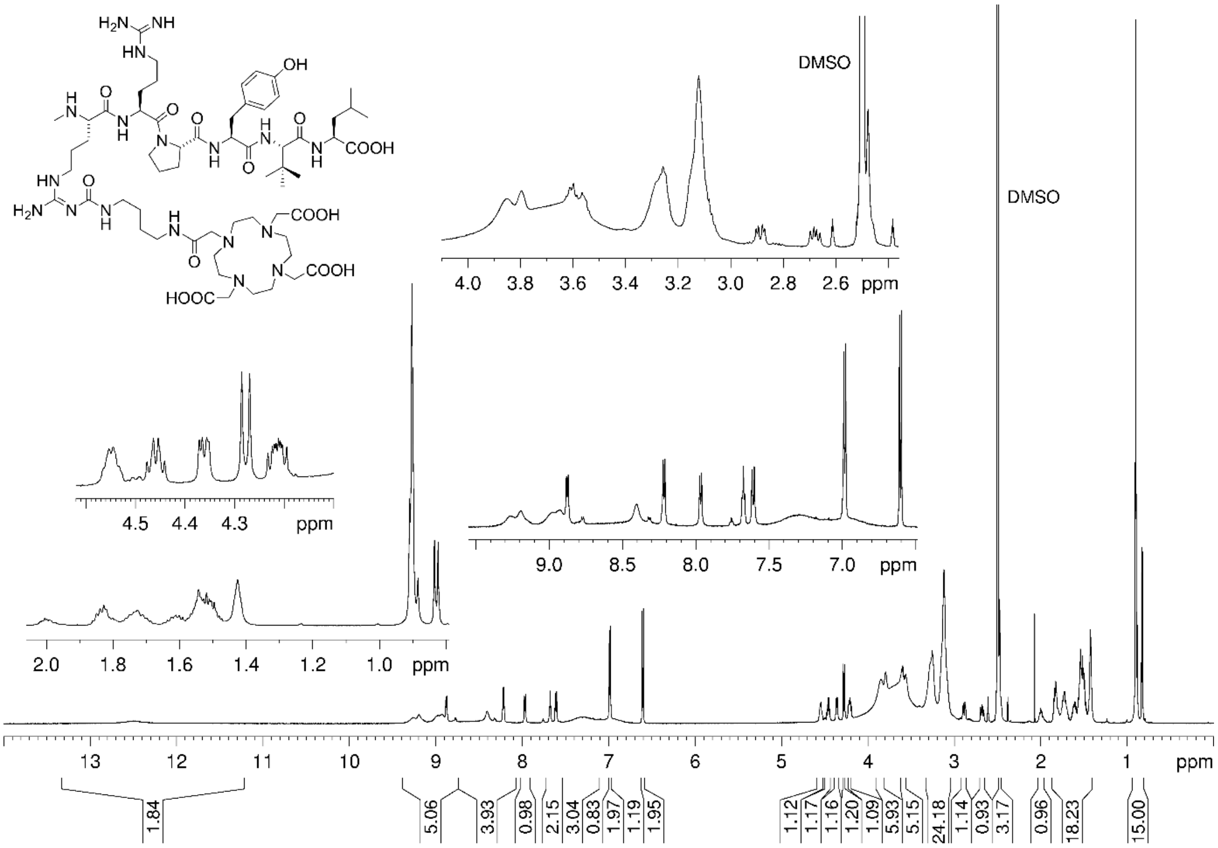

^{1}H-NMR spectrum (600 MHz, DMSO-d_6) of compound **16**

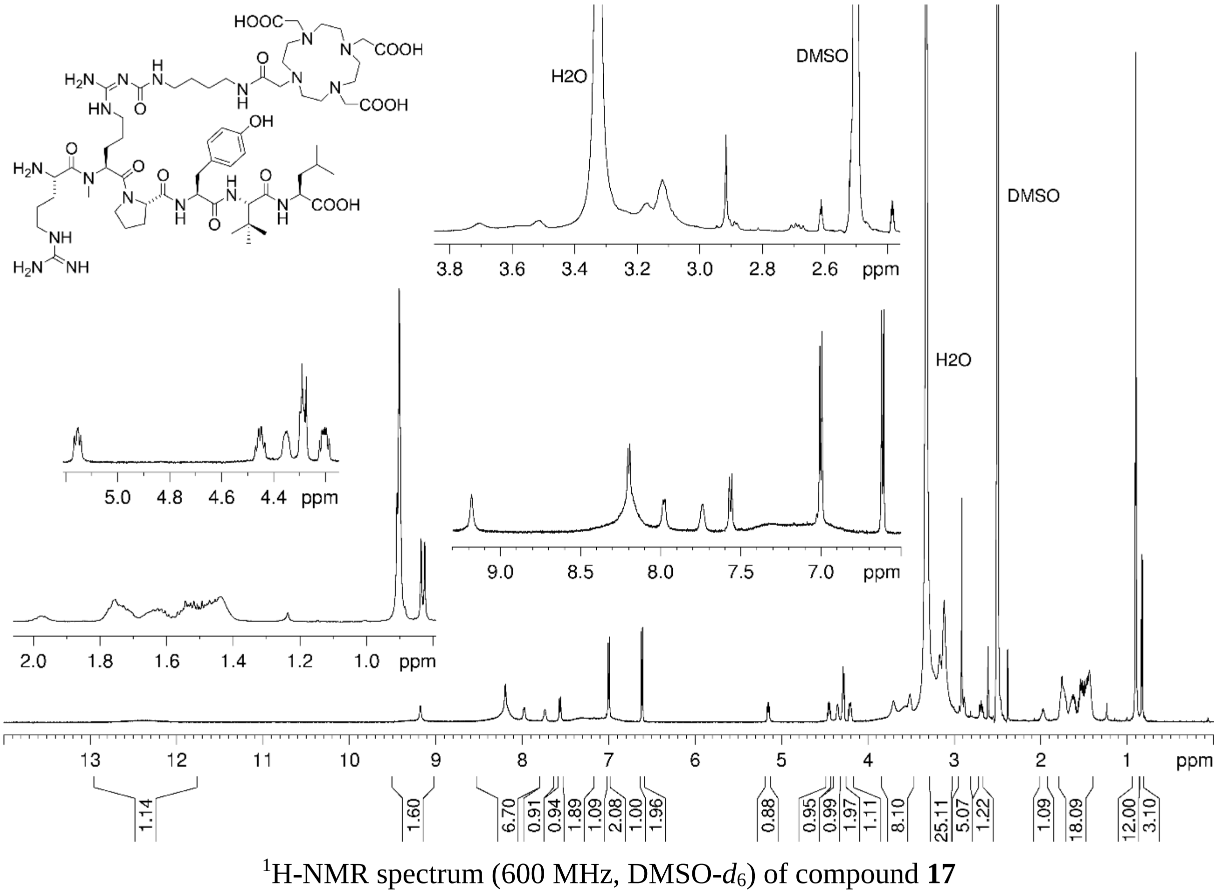

^{1}H-NMR spectrum (600 MHz, DMSO-d_6) of compound **17**

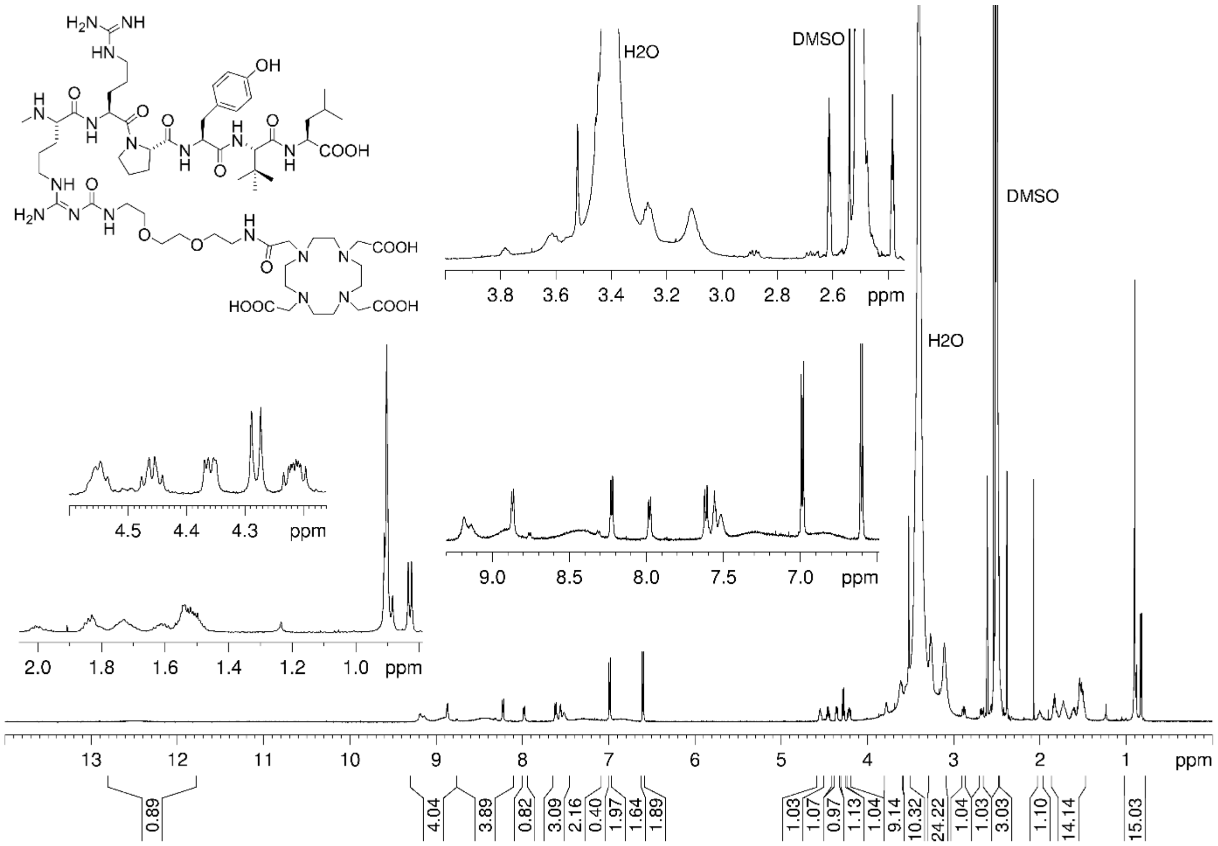

^{1}H-NMR spectrum (600 MHz, DMSO-d_6) of compound **18**

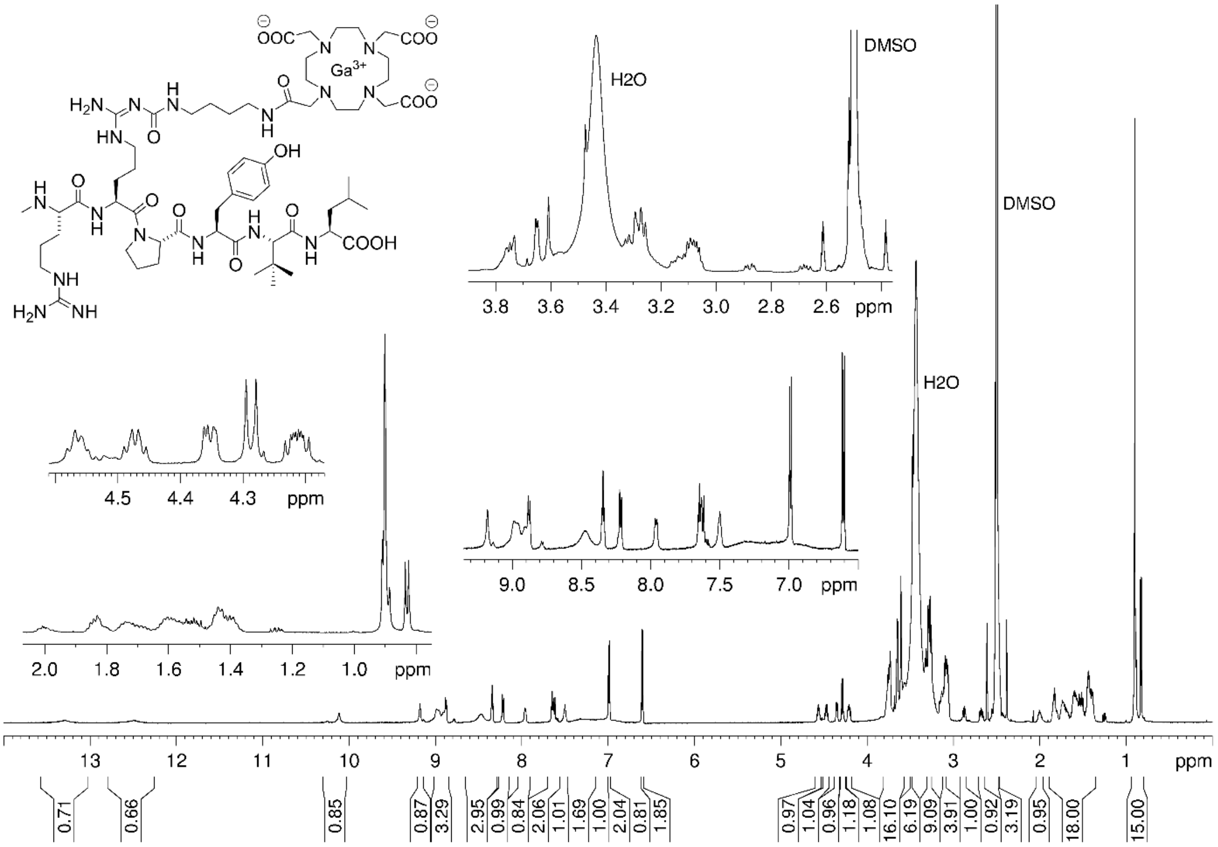

^{1}H-NMR spectrum (600 MHz, DMSO-d_6) of compound **19**

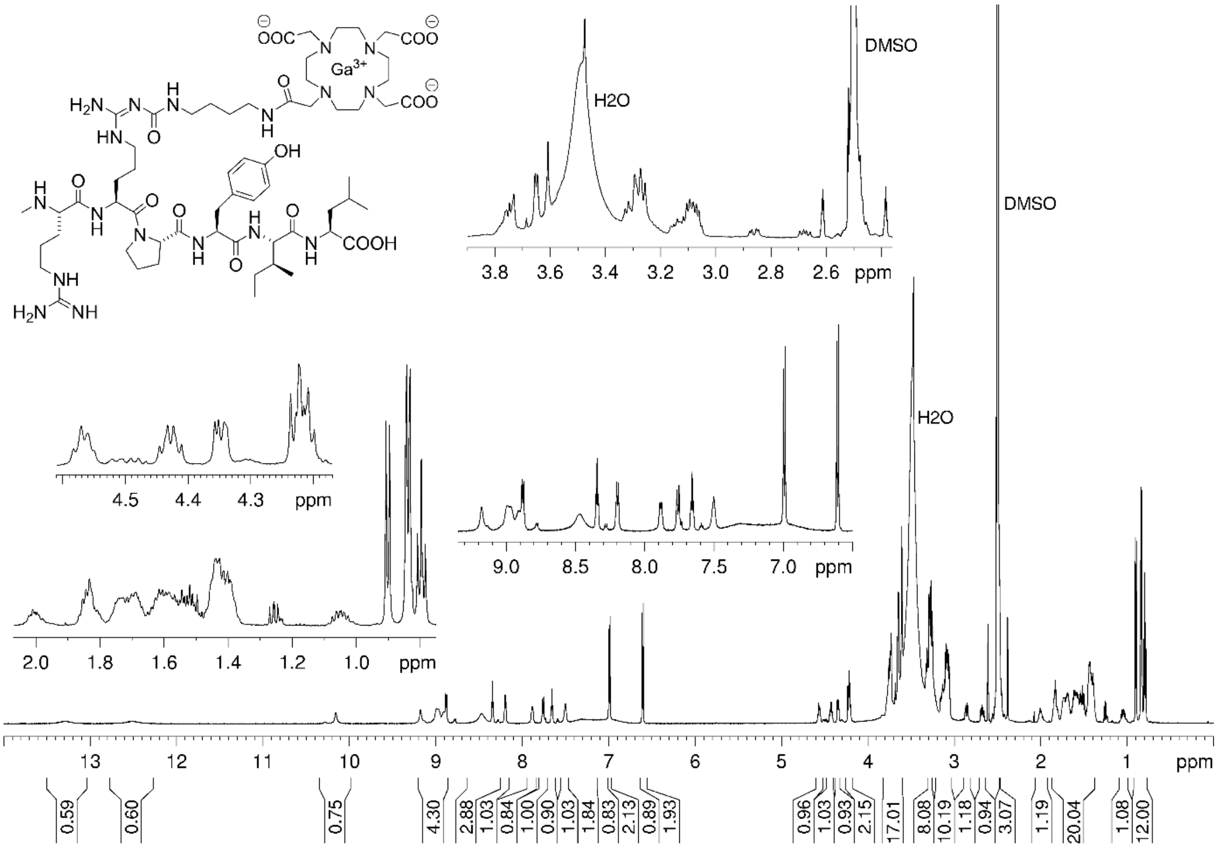

^{1}H-NMR spectrum (600 MHz, DMSO-d_6) of compound **20**

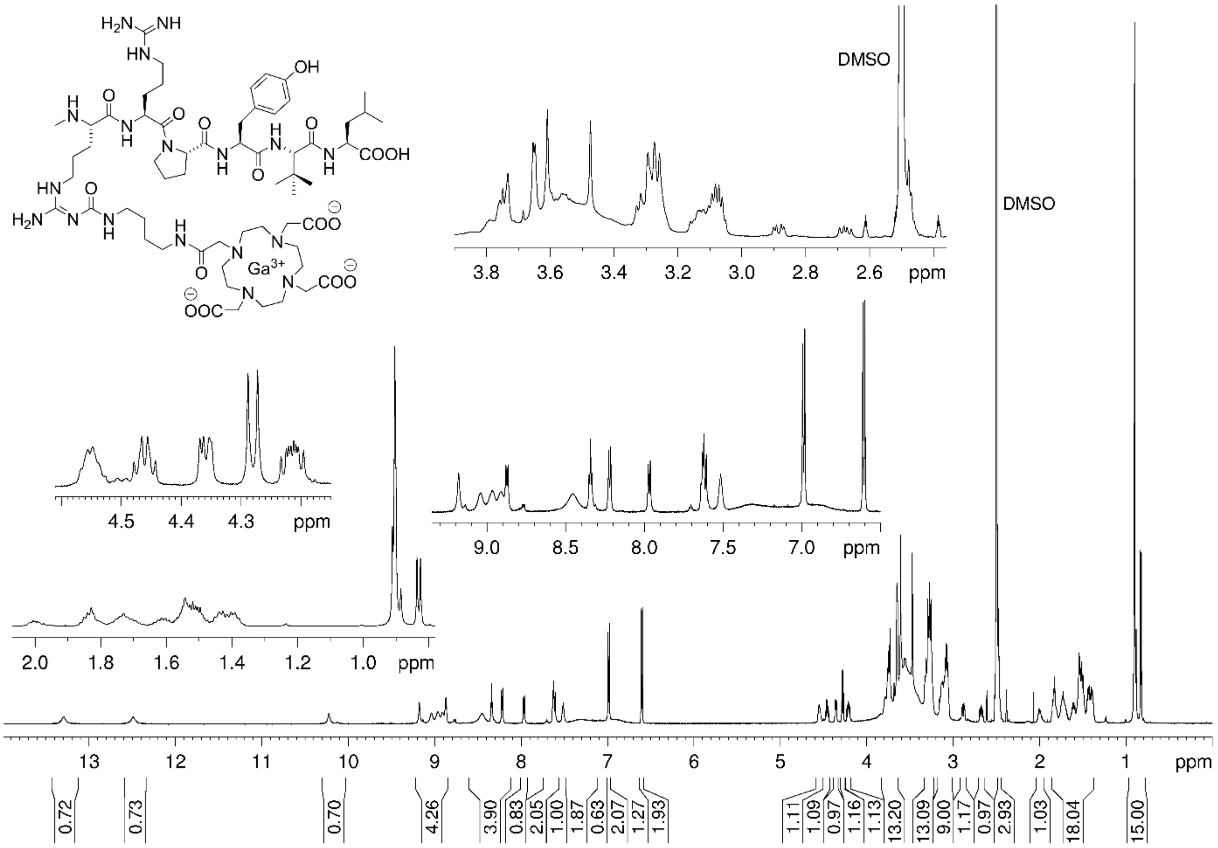

^{1}H-NMR spectrum (600 MHz, DMSO-d_6) of compound **21**

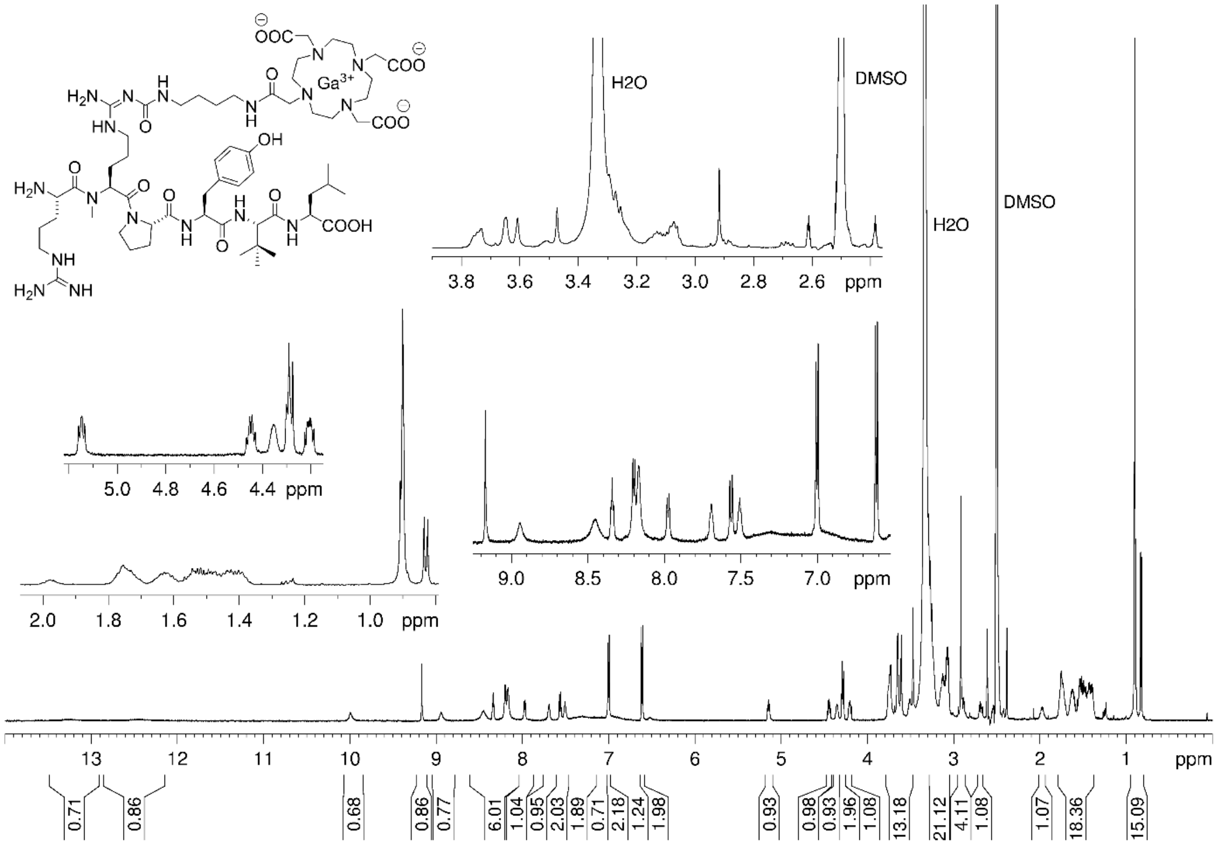

^{1}H-NMR spectrum (600 MHz, DMSO-d_6) of compound **22**

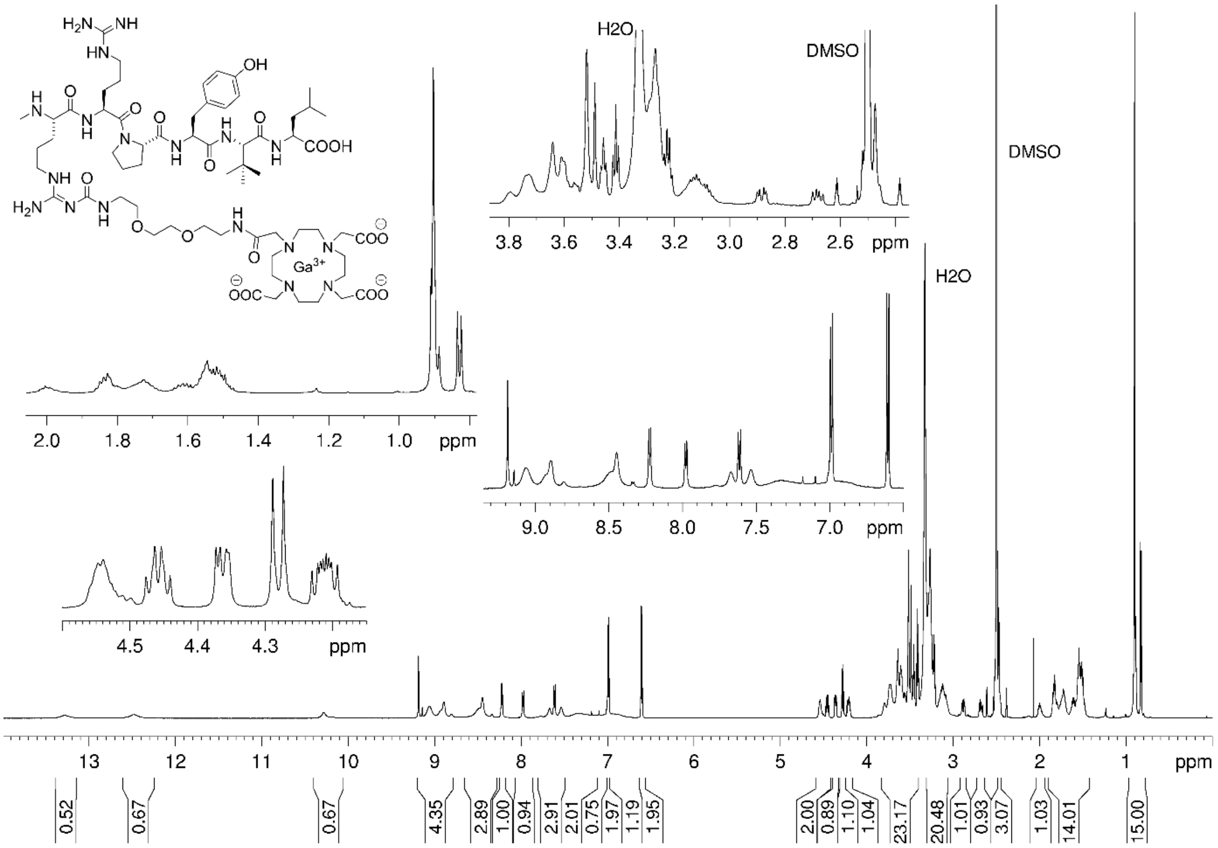

^{1}H-NMR spectrum (600 MHz, DMSO-d_6) of compound **23**

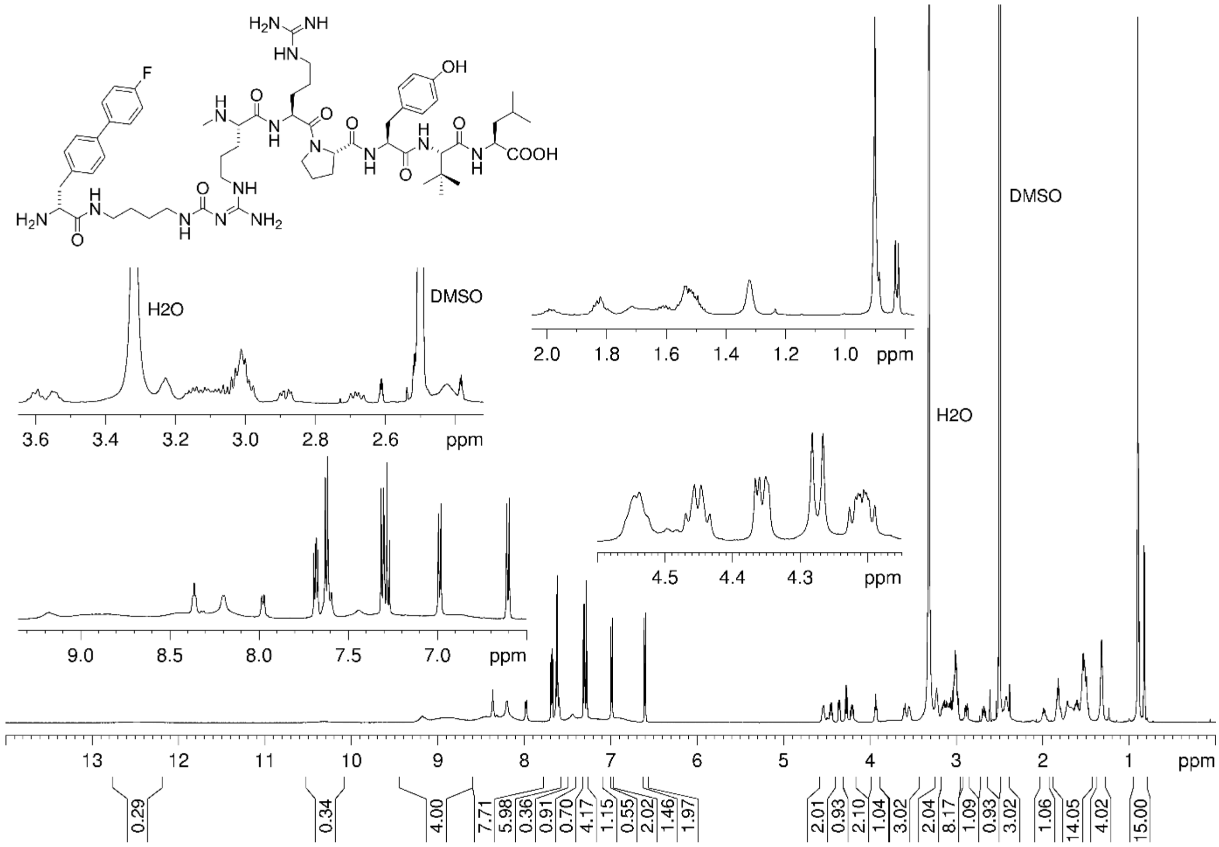

^{1}H-NMR spectrum (600 MHz, DMSO-d_6) of compound **25**

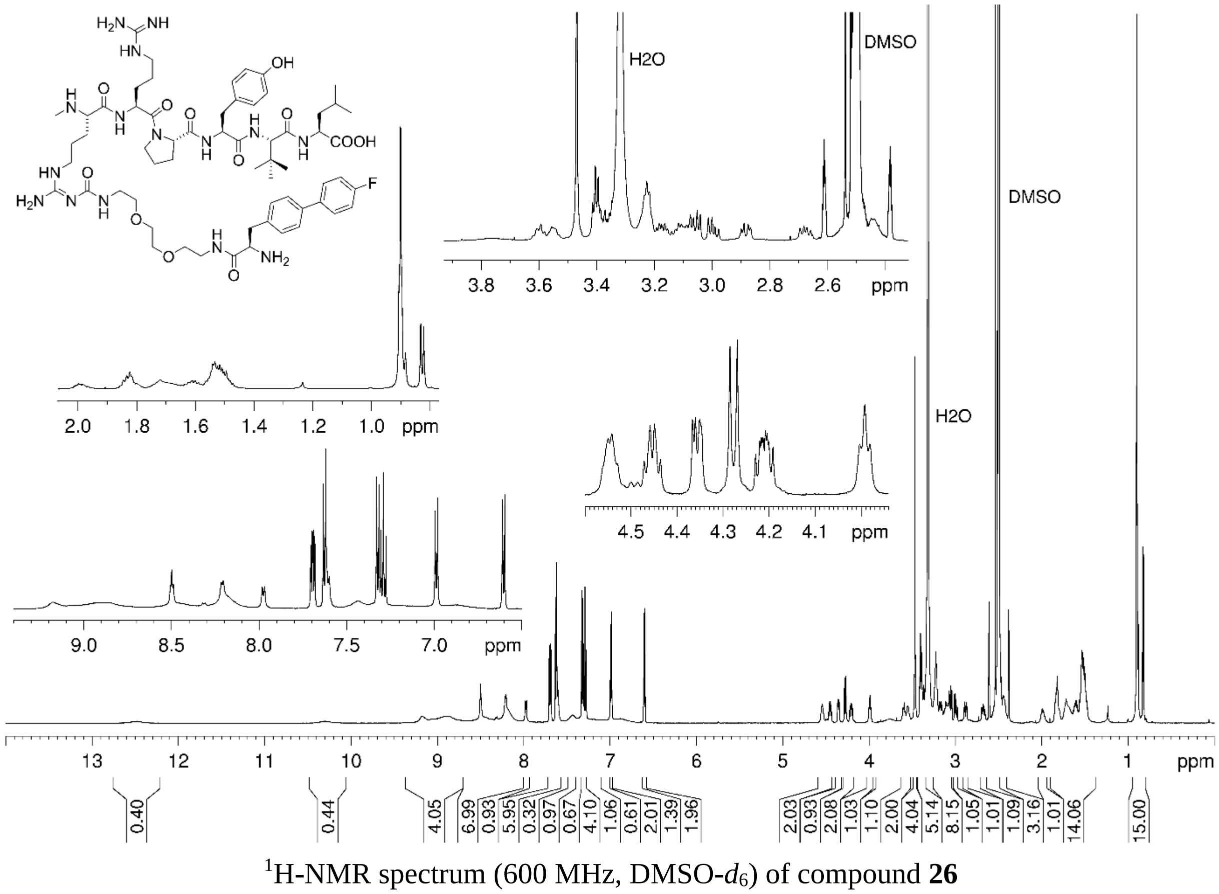

^{1}H-NMR spectrum (600 MHz, DMSO-d_6) of compound **26**

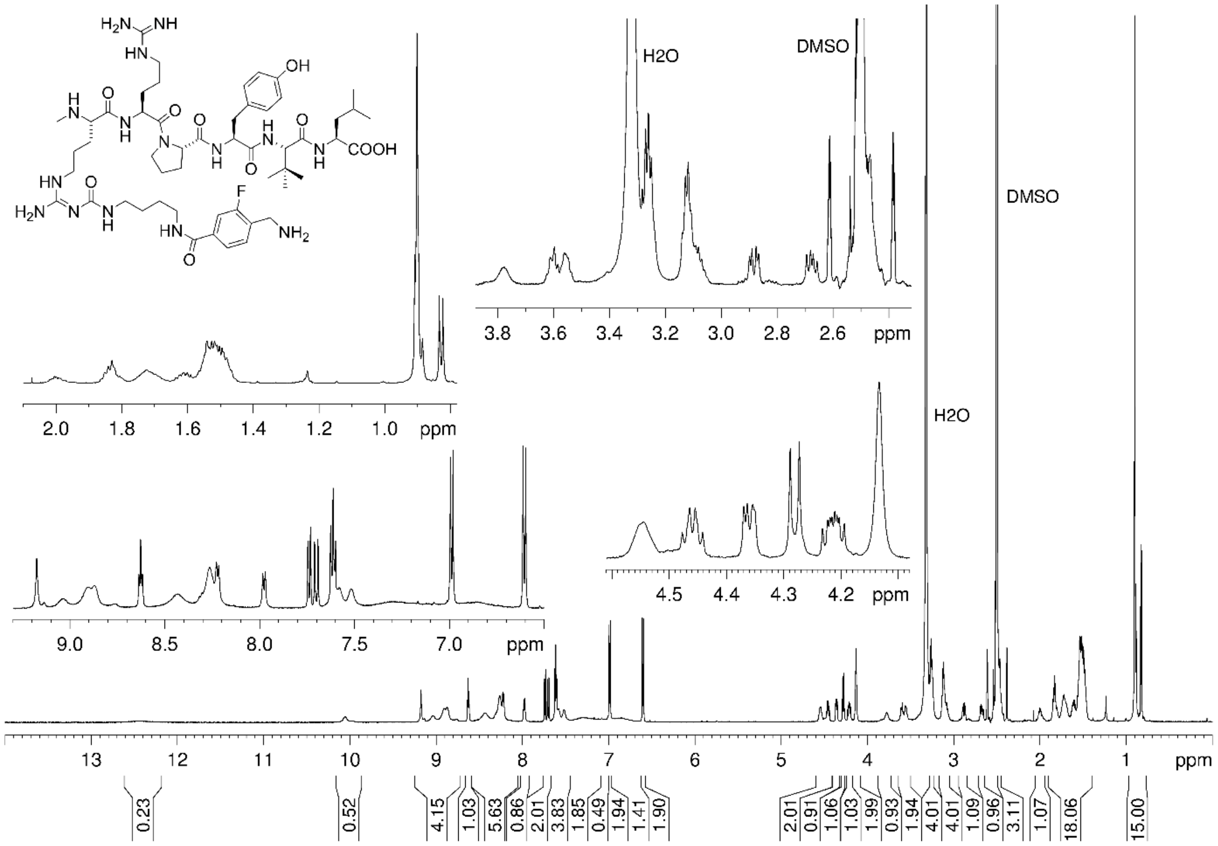

^{1}H-NMR spectrum (600 MHz, DMSO-d_6) of compound **28**

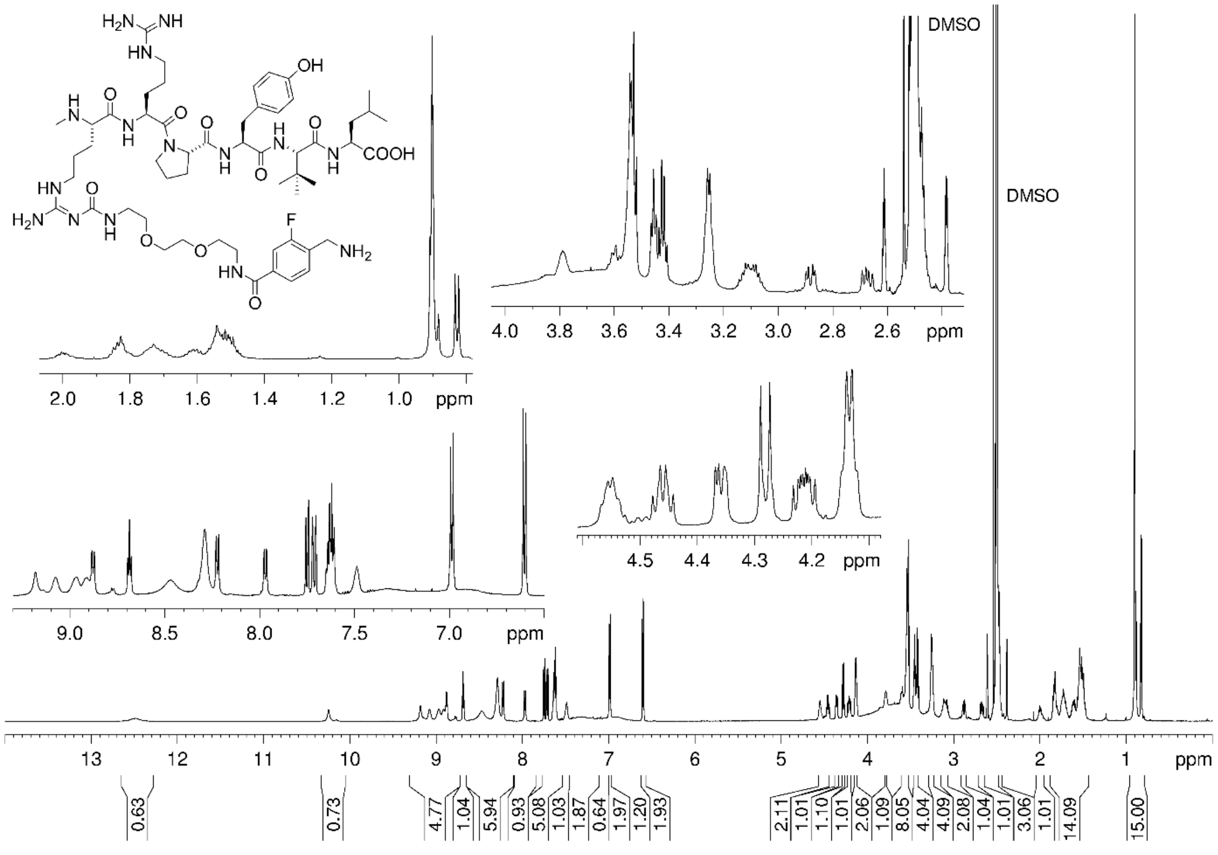

^{1}H-NMR spectrum (600 MHz, DMSO-d_6) of compound **29**

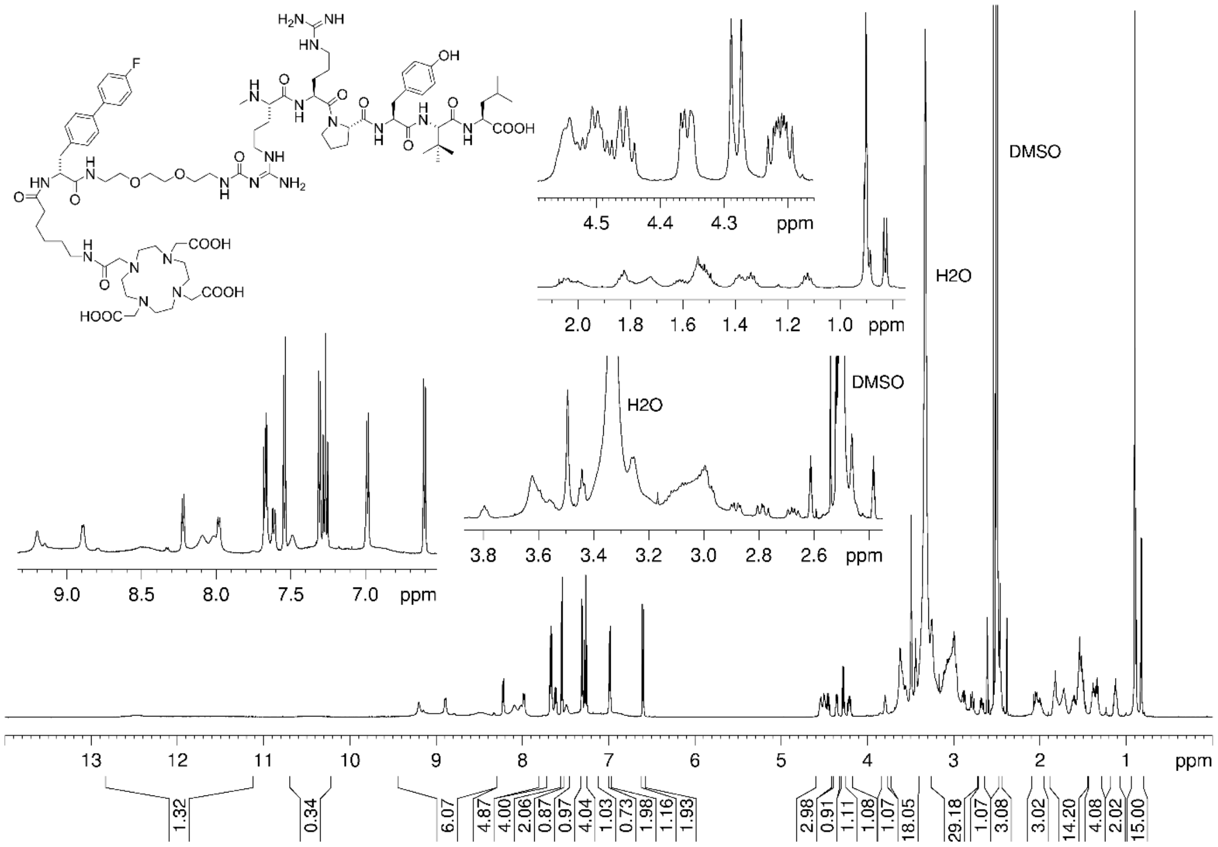

^{1}H-NMR spectrum (600 MHz, DMSO-d_6) of compound **32**

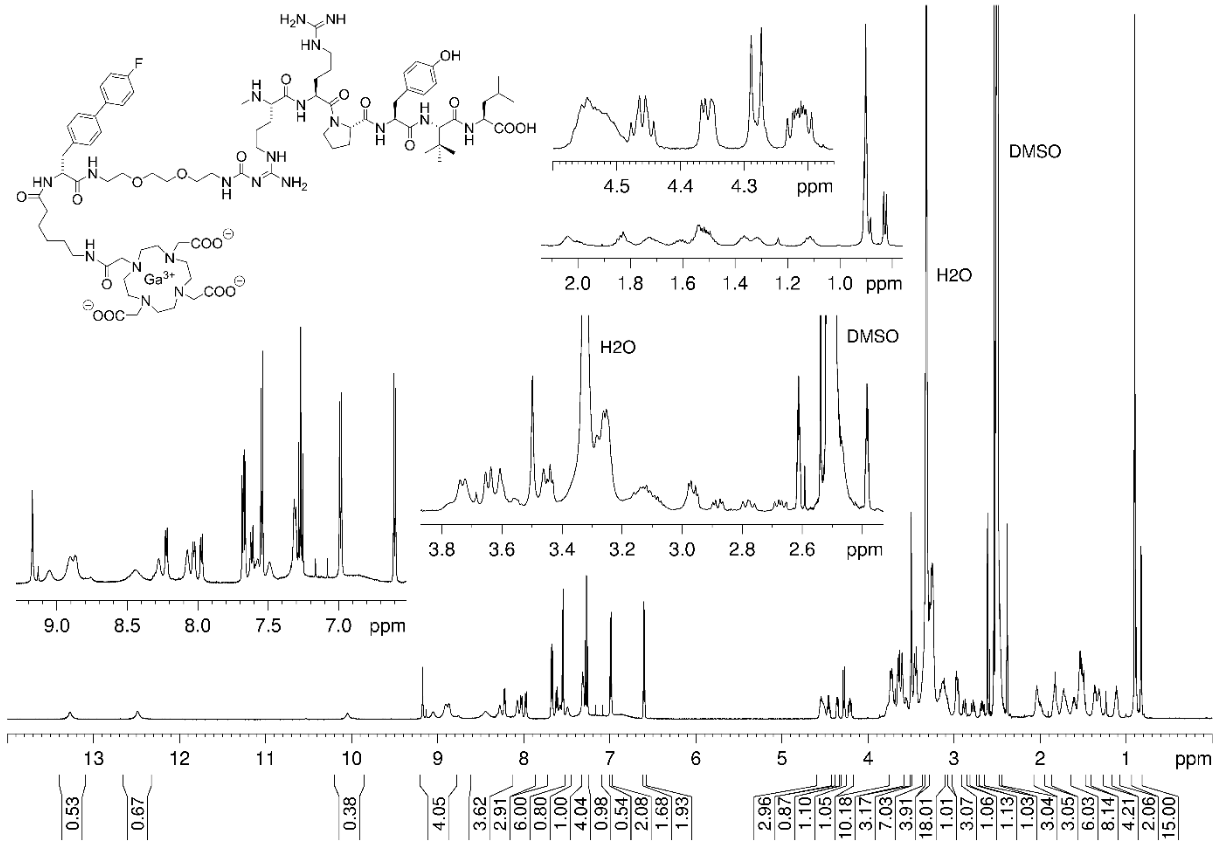

^{1}H-NMR spectrum (600 MHz, DMSO-d_6) of compound **33**

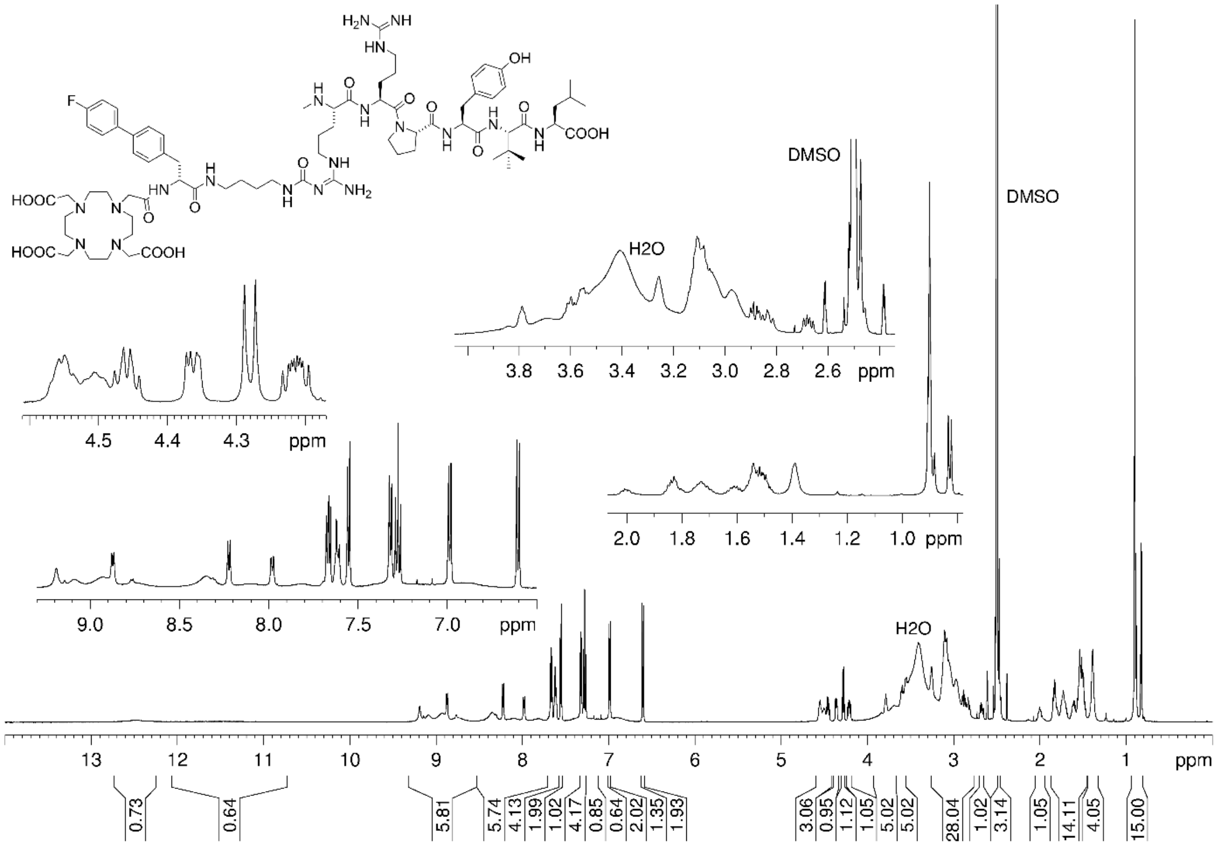

^{1}H-NMR spectrum (600 MHz, DMSO-d_6) of compound **34**

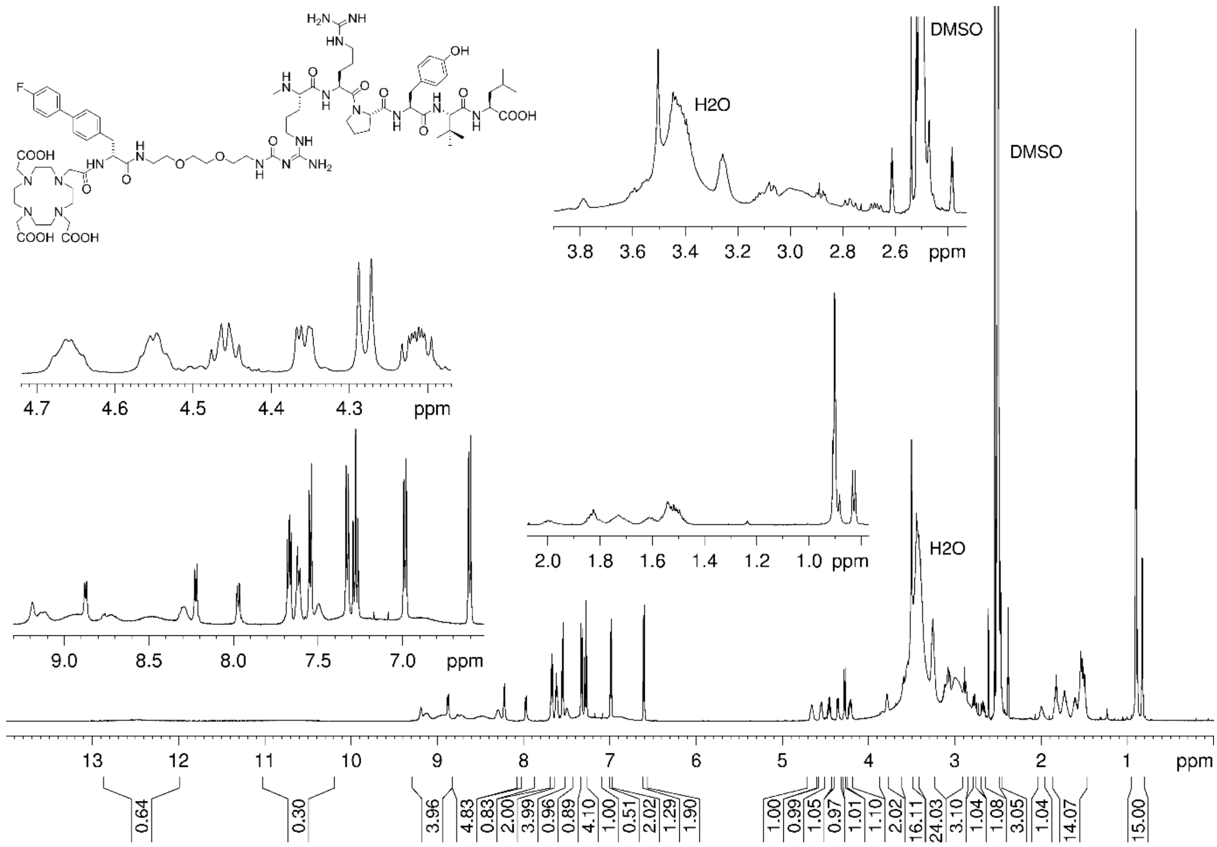

^{1}H-NMR spectrum (600 MHz, DMSO-d_6) of compound **35**

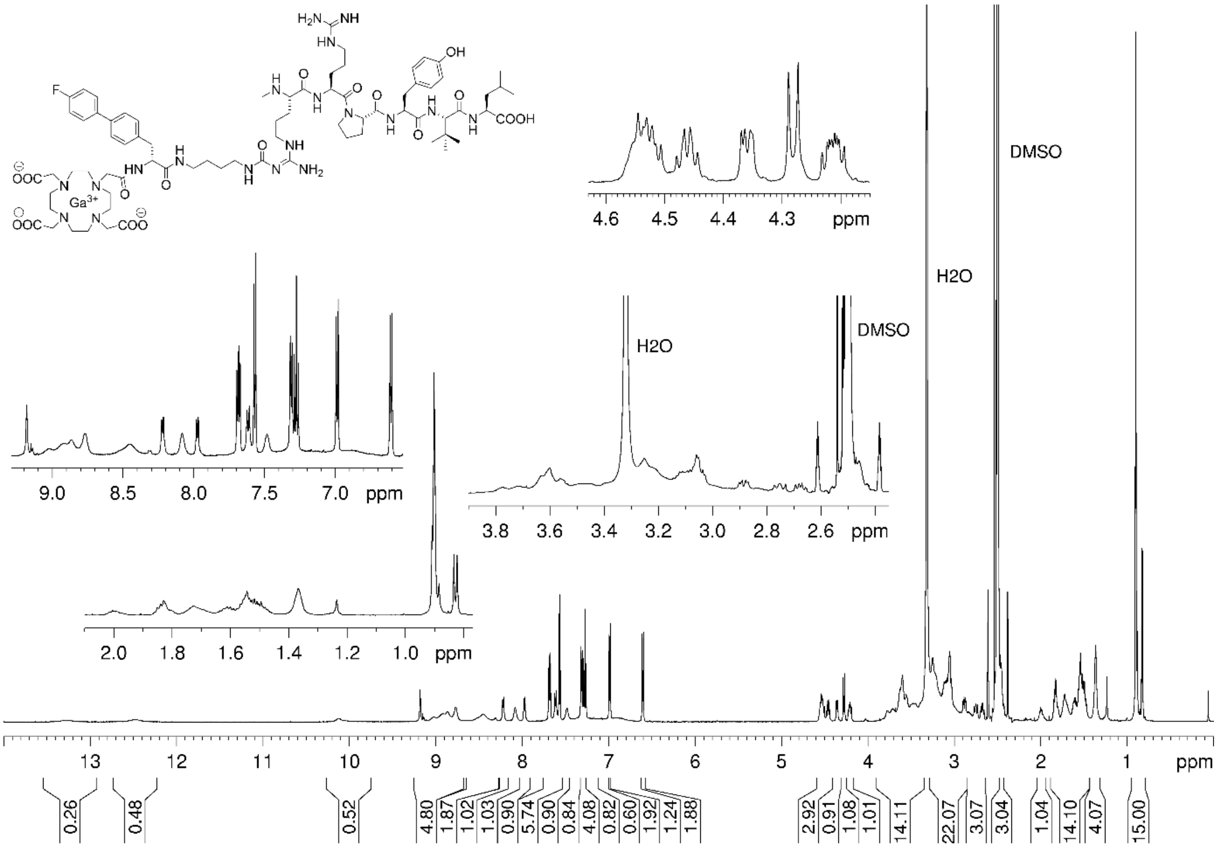

^{1}H-NMR spectrum (600 MHz, DMSO-d_6) of compound **36**

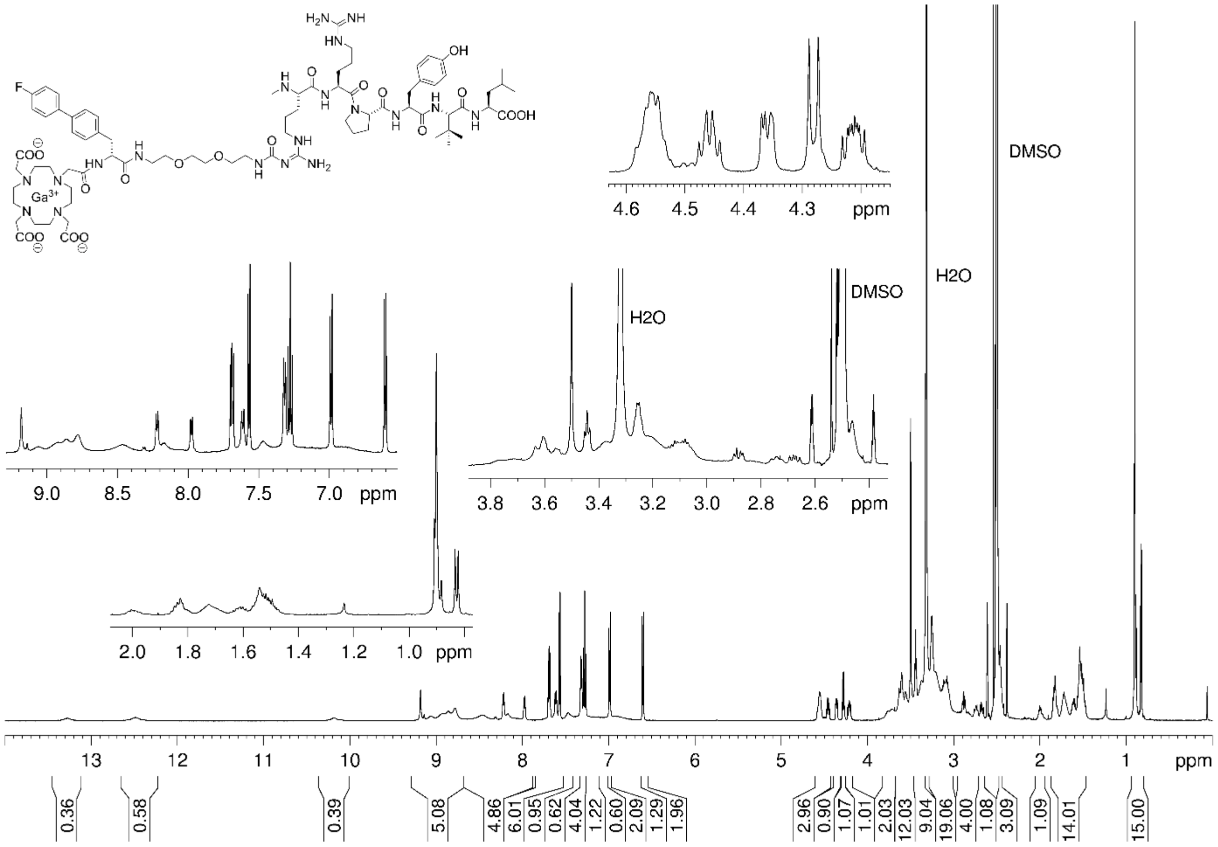

^{1}H-NMR spectrum (600 MHz, DMSO-d_6) of compound **37**

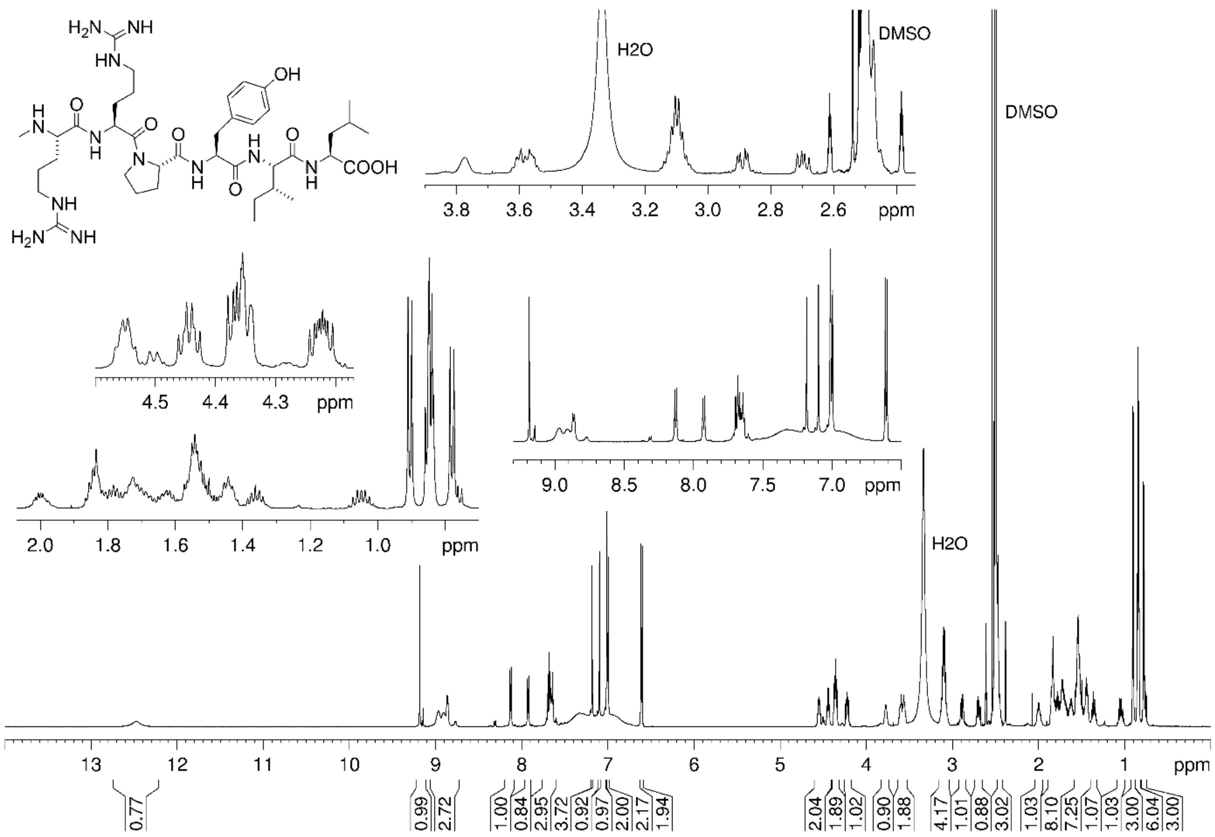

^{1}H-NMR spectrum (600 MHz, DMSO-d_6) of compound **38**

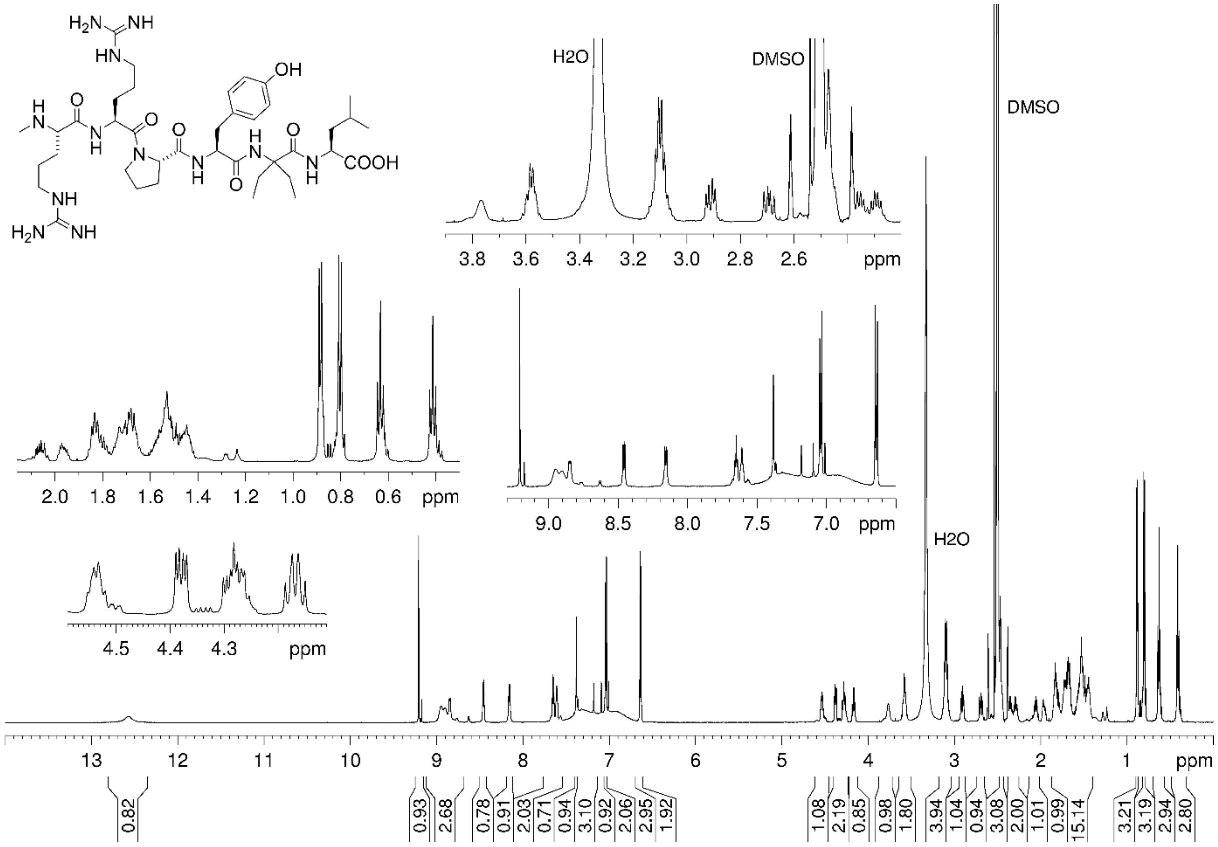

^{1}H-NMR spectrum (600 MHz, DMSO-d_6) of compound **39**

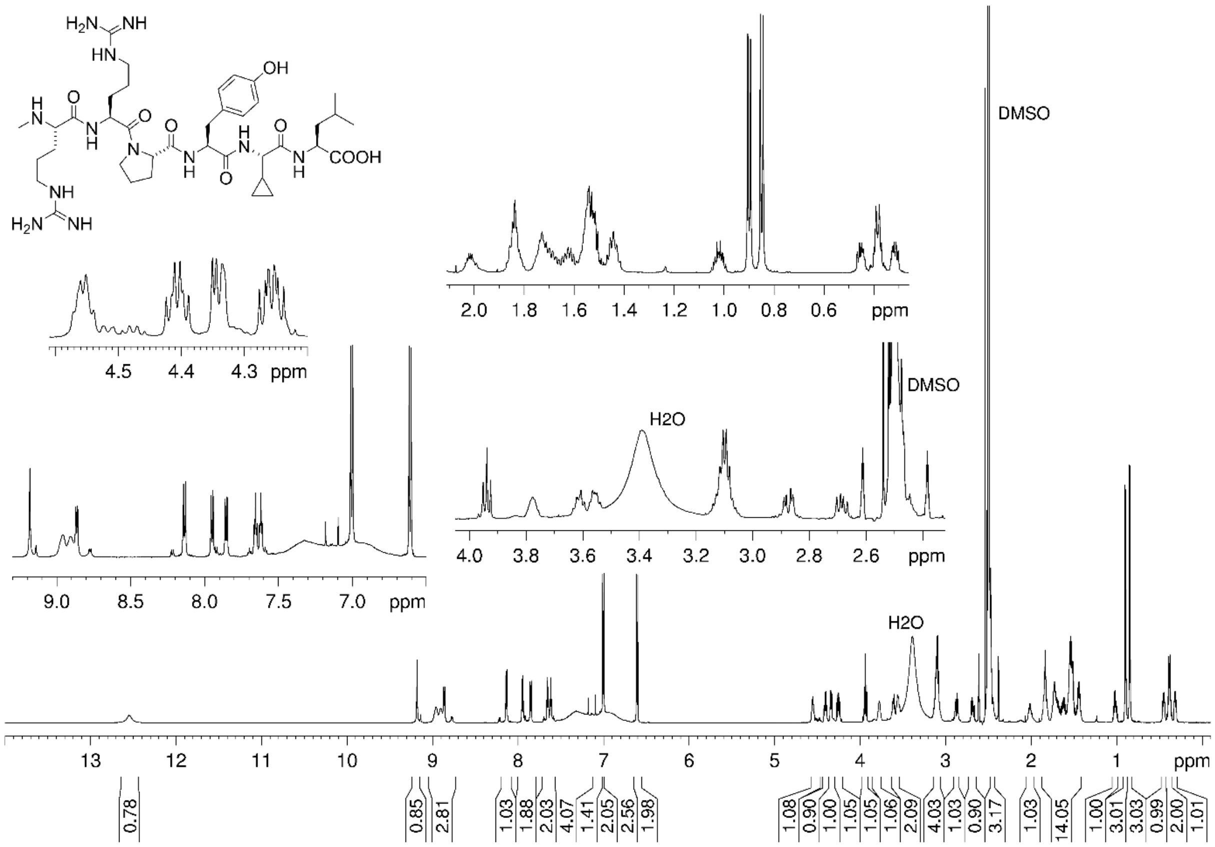

^{1}H-NMR spectrum (600 MHz, DMSO-d_6) of compound **40**

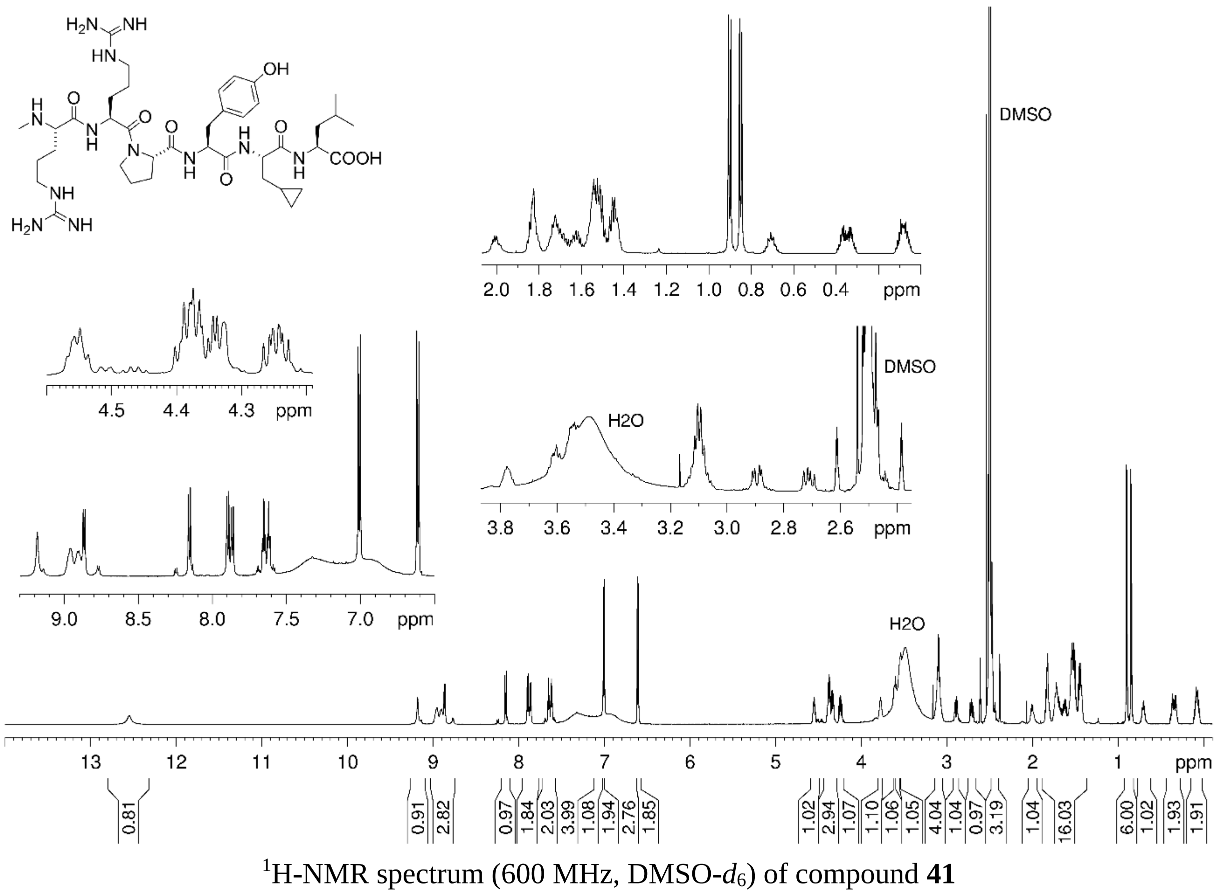

^{1}H-NMR spectrum (600 MHz, DMSO-d_6) of compound **41**

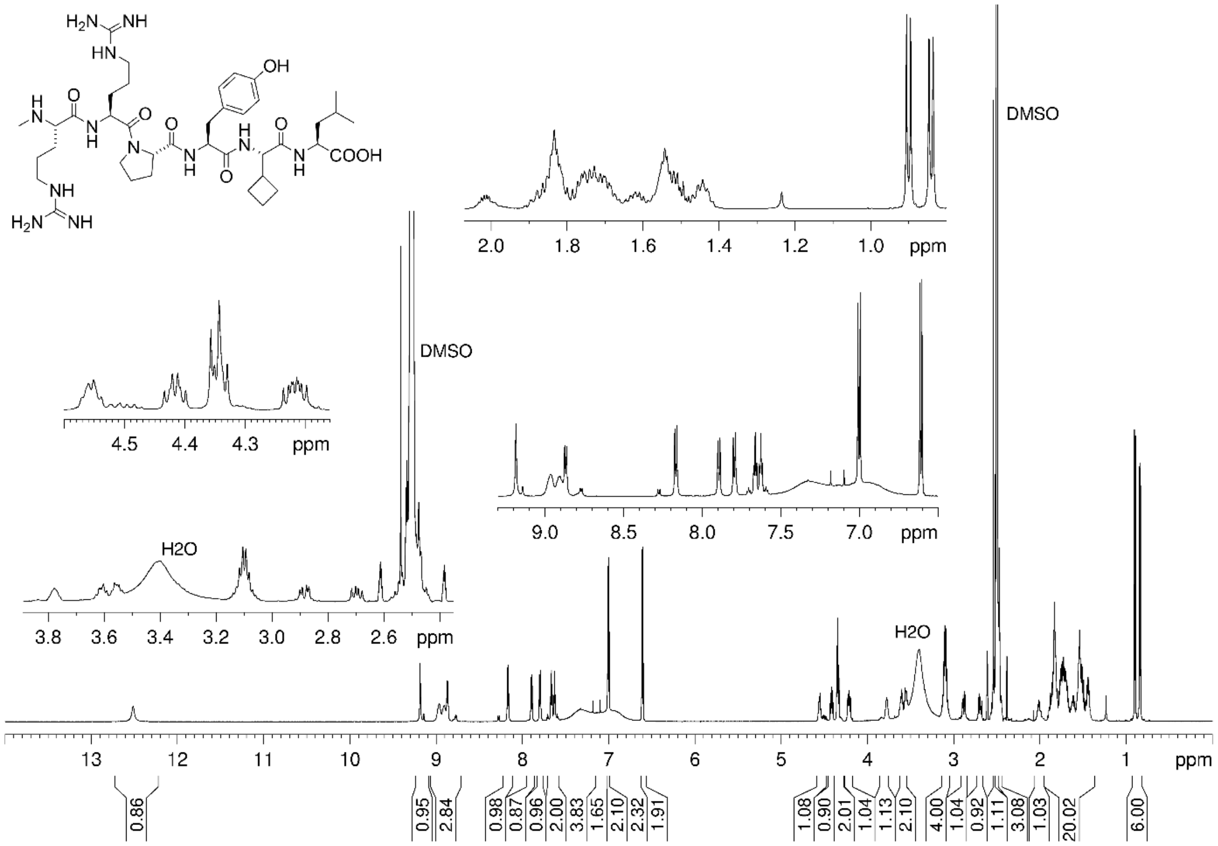

^{1}H-NMR spectrum (600 MHz, DMSO-d_6) of compound **42**

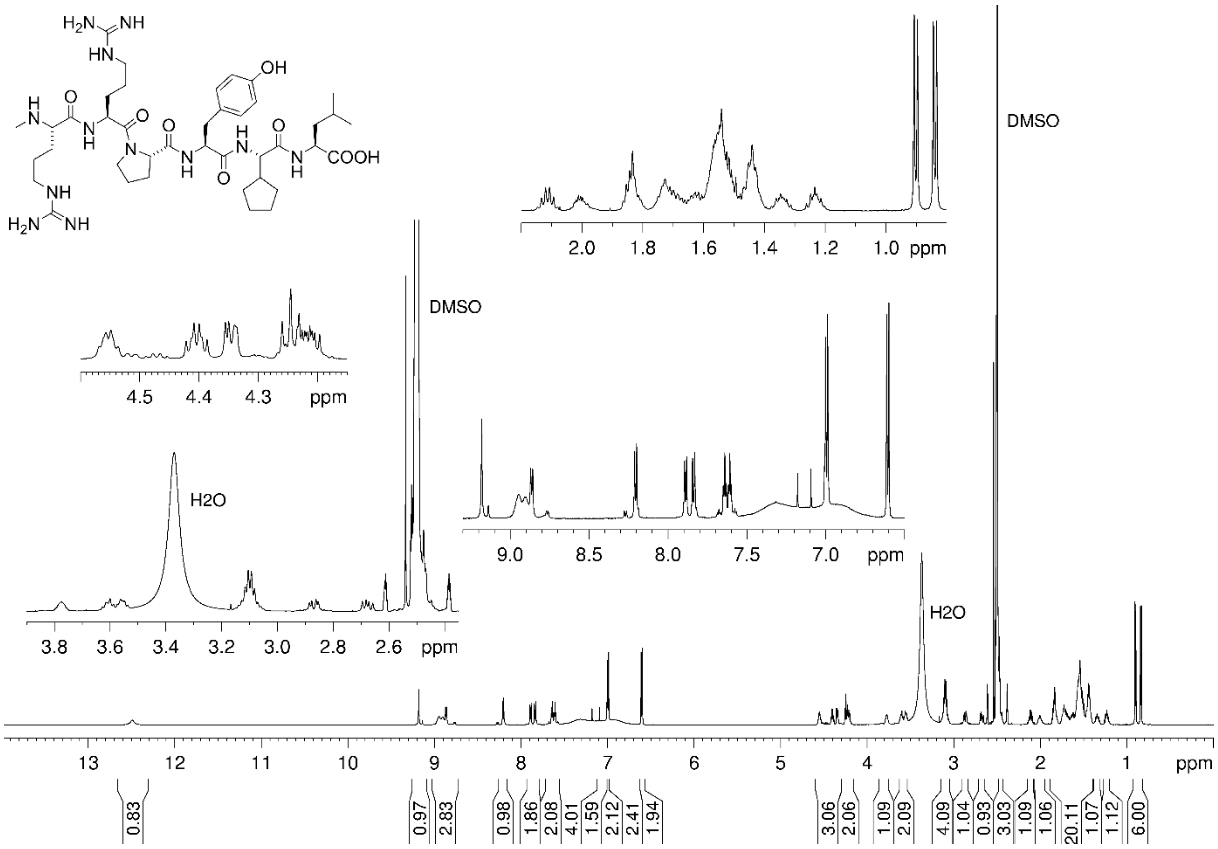

^{1}H-NMR spectrum (600 MHz, DMSO-d_6) of compound **43**

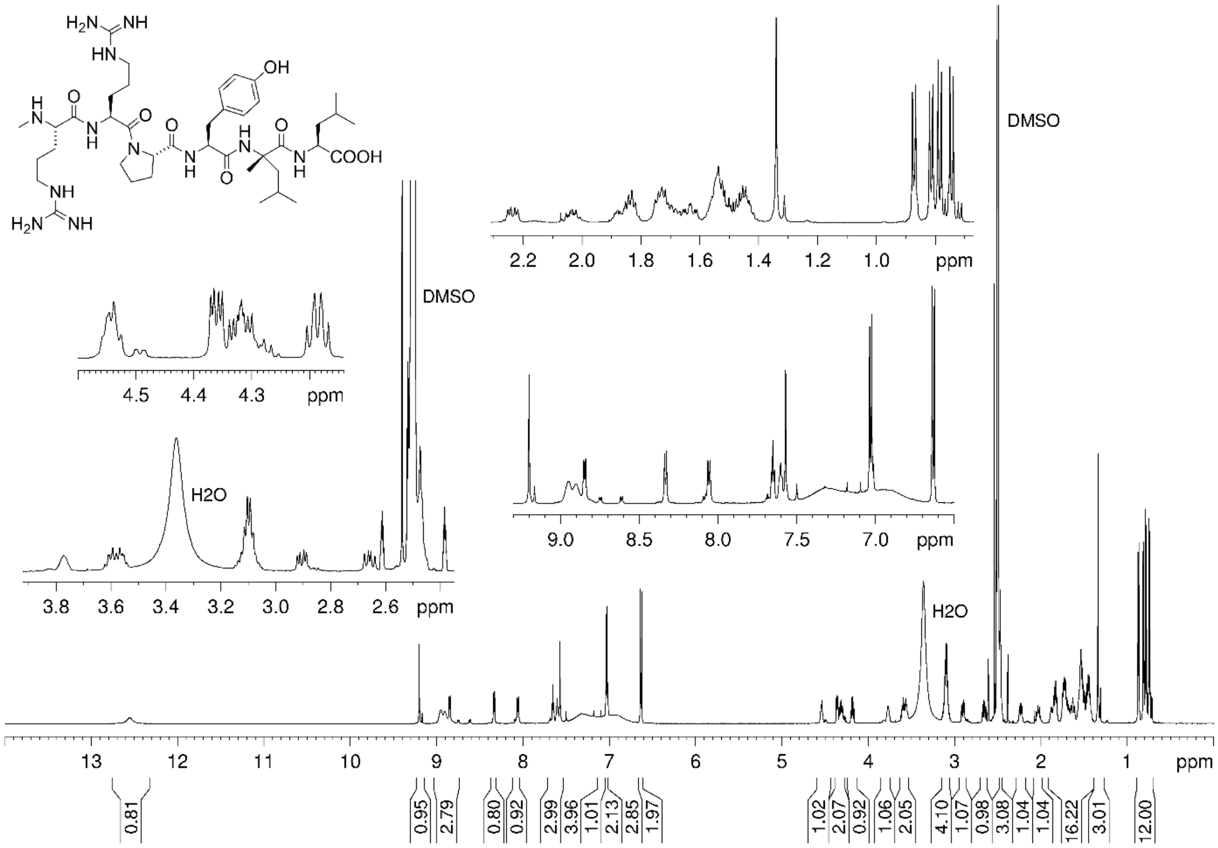

^{1}H-NMR spectrum (600 MHz, DMSO-d_6) of compound **44**

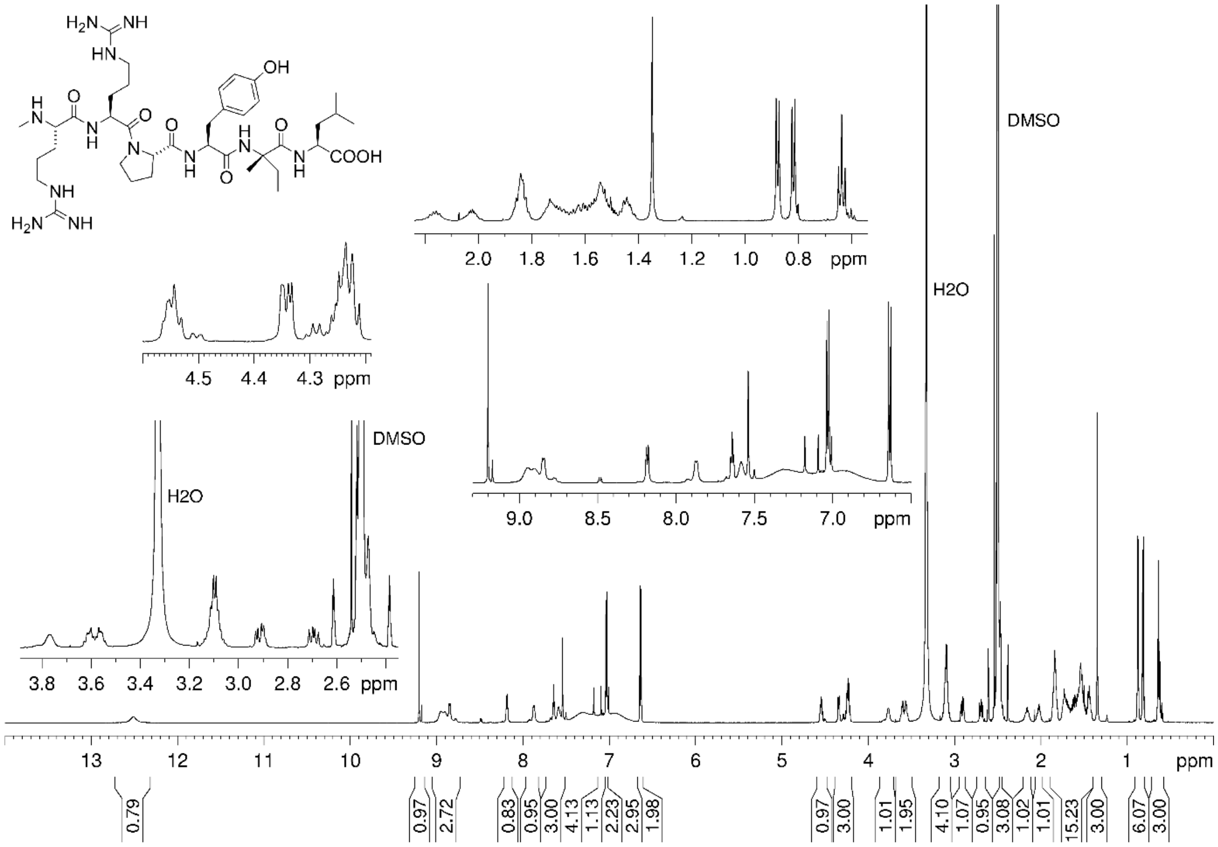

^{1}H-NMR spectrum (600 MHz, DMSO-d_6) of compound **45**

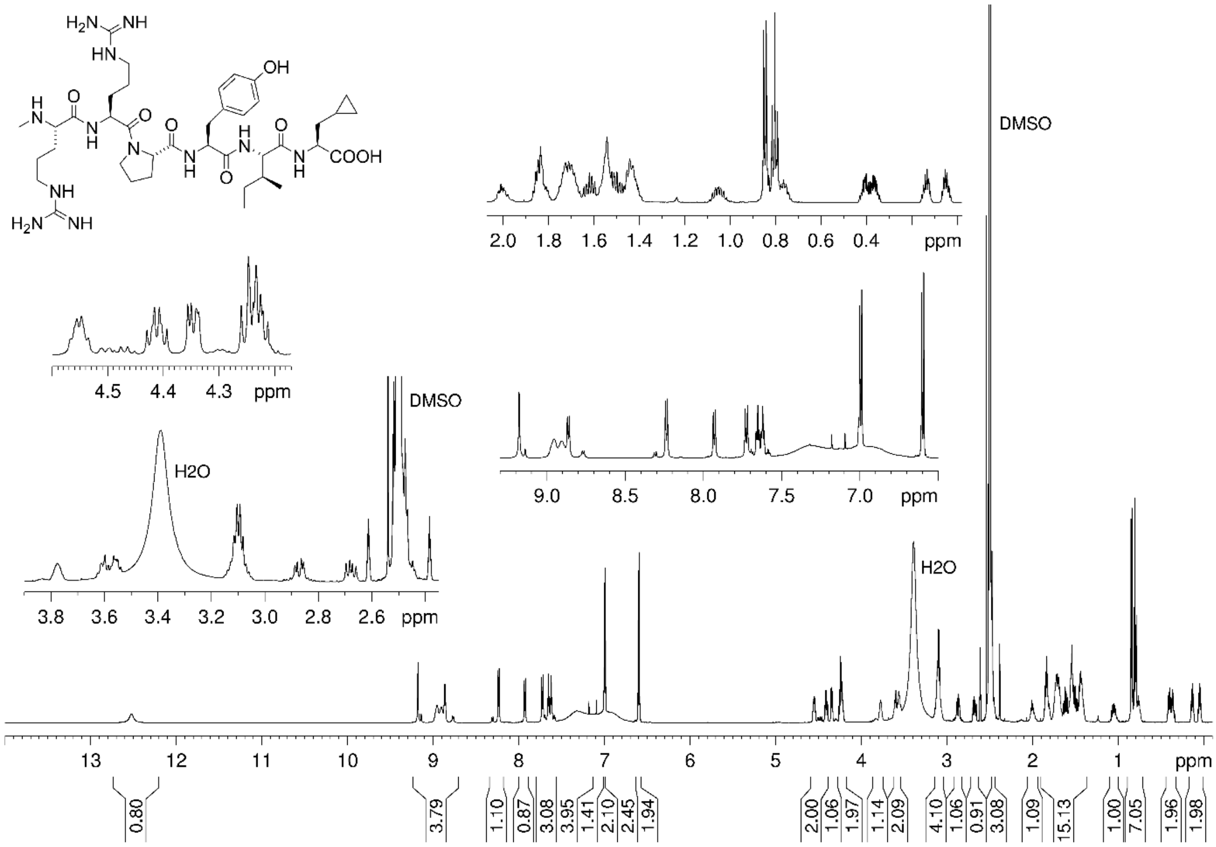

^{1}H-NMR spectrum (600 MHz, DMSO-d_6) of compound **46**

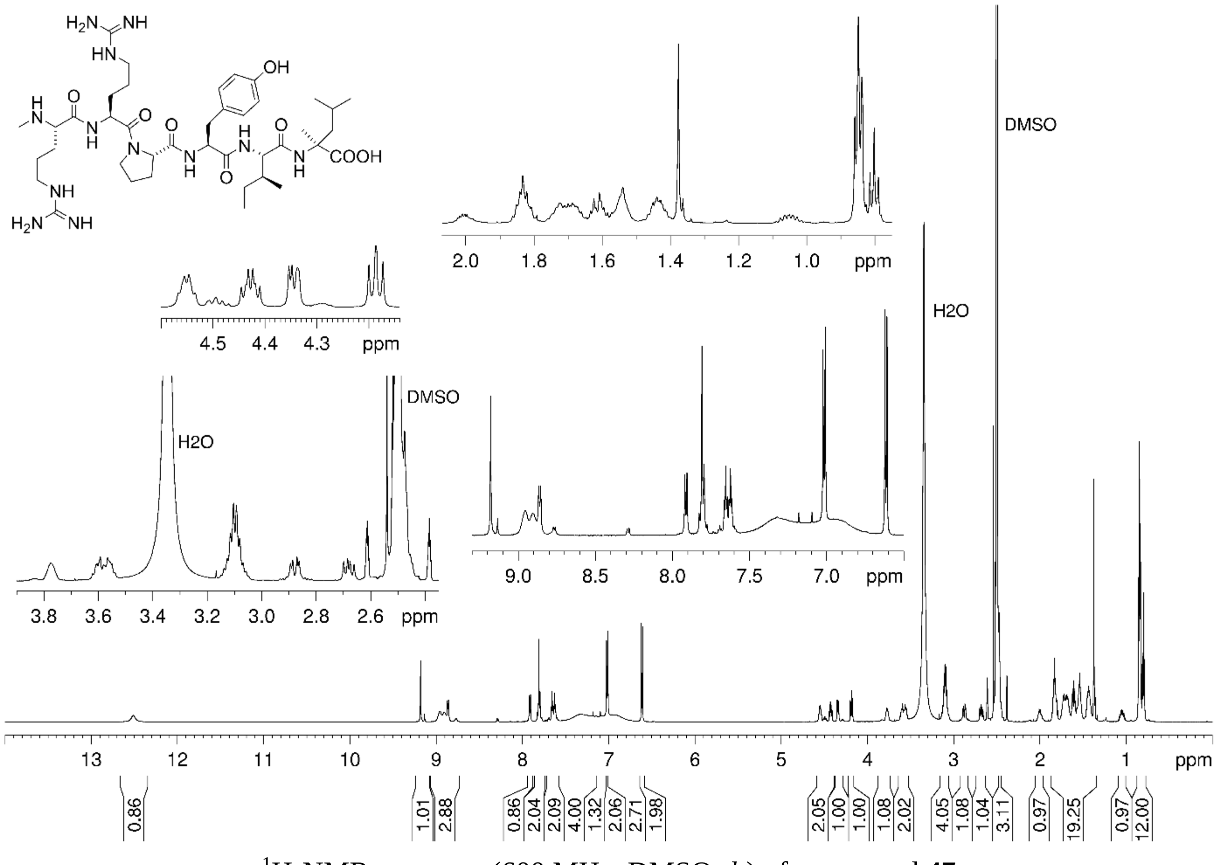

^{1}H-NMR spectrum (600 MHz, DMSO-d_6) of compound **47**

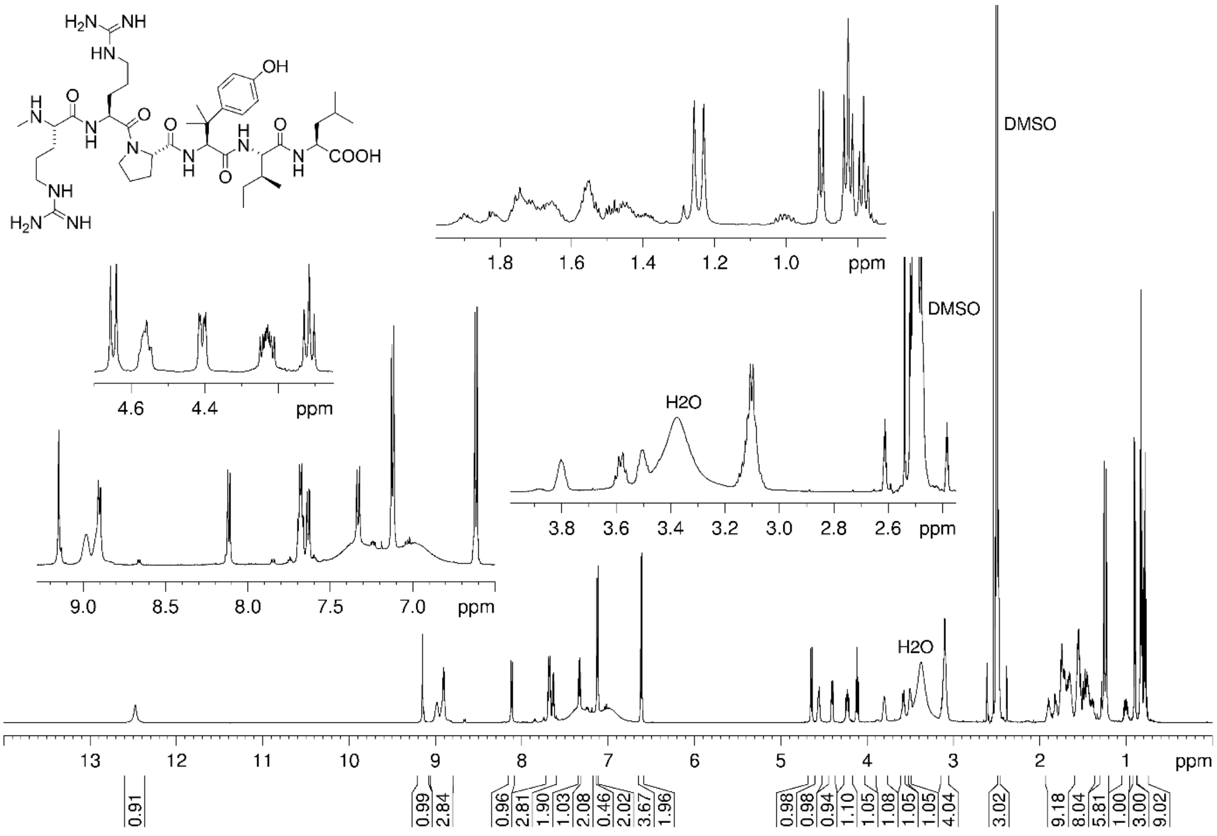

^{1}H-NMR spectrum (600 MHz, DMSO-d_6) of compound **48**

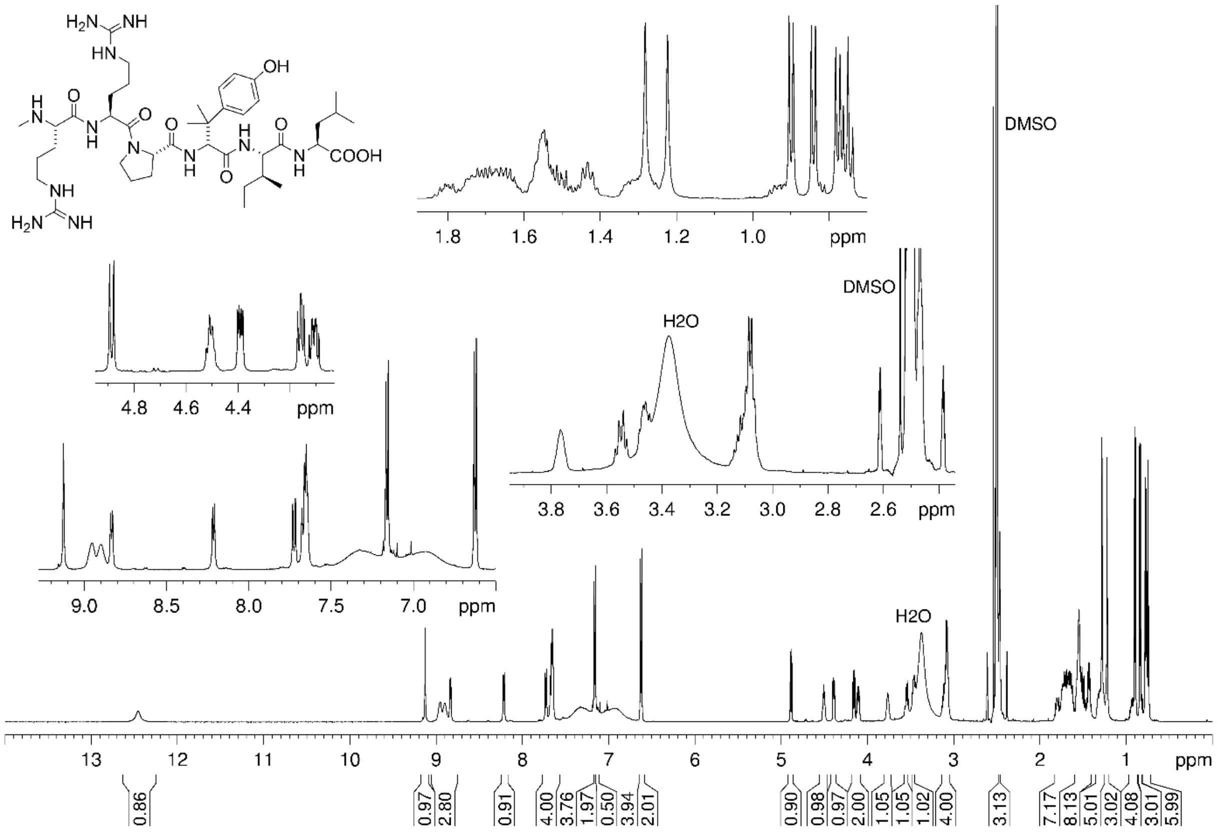

^{1}H-NMR spectrum (600 MHz, DMSO-d_6) of compound **49**

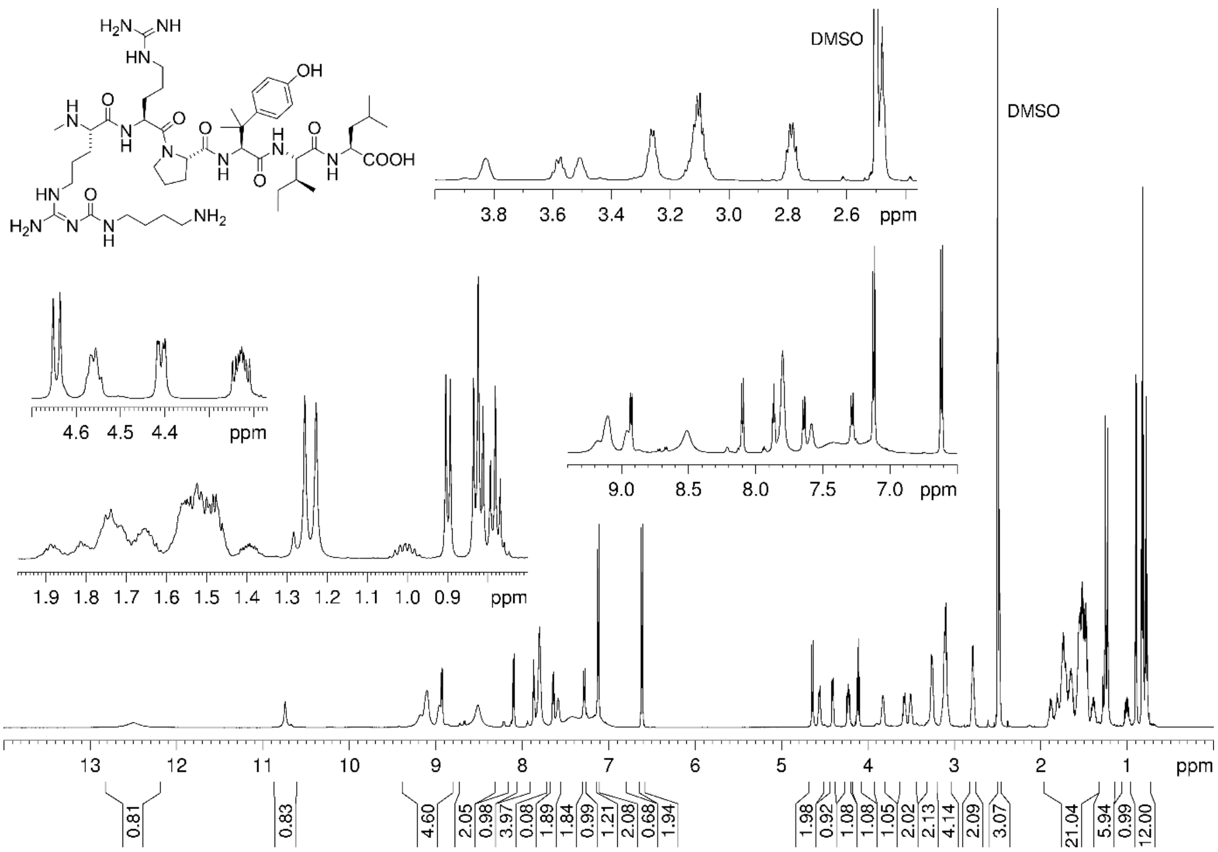

^{1}H-NMR spectrum (600 MHz, DMSO-d_6) of compound **50**

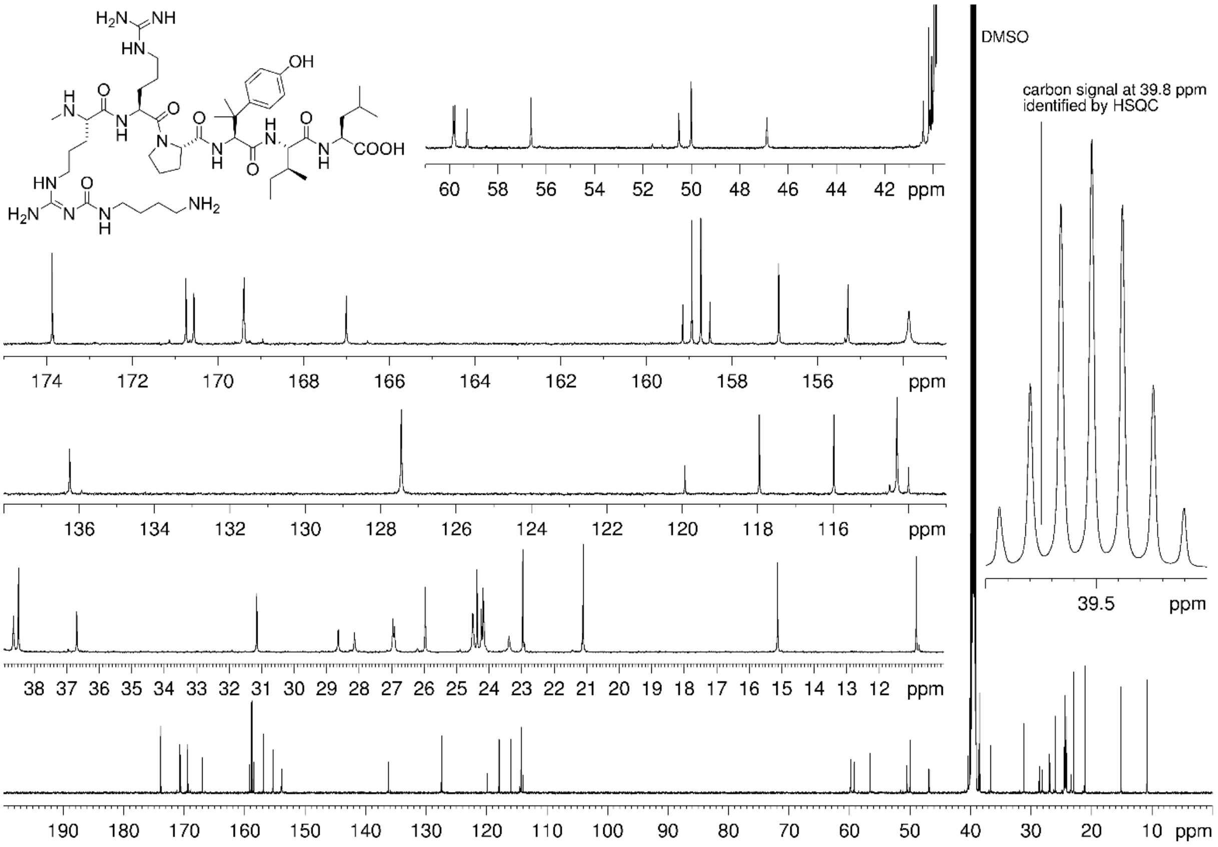

^{13}C-NMR spectrum (151 MHz, DMSO-d_6) of compound **50**

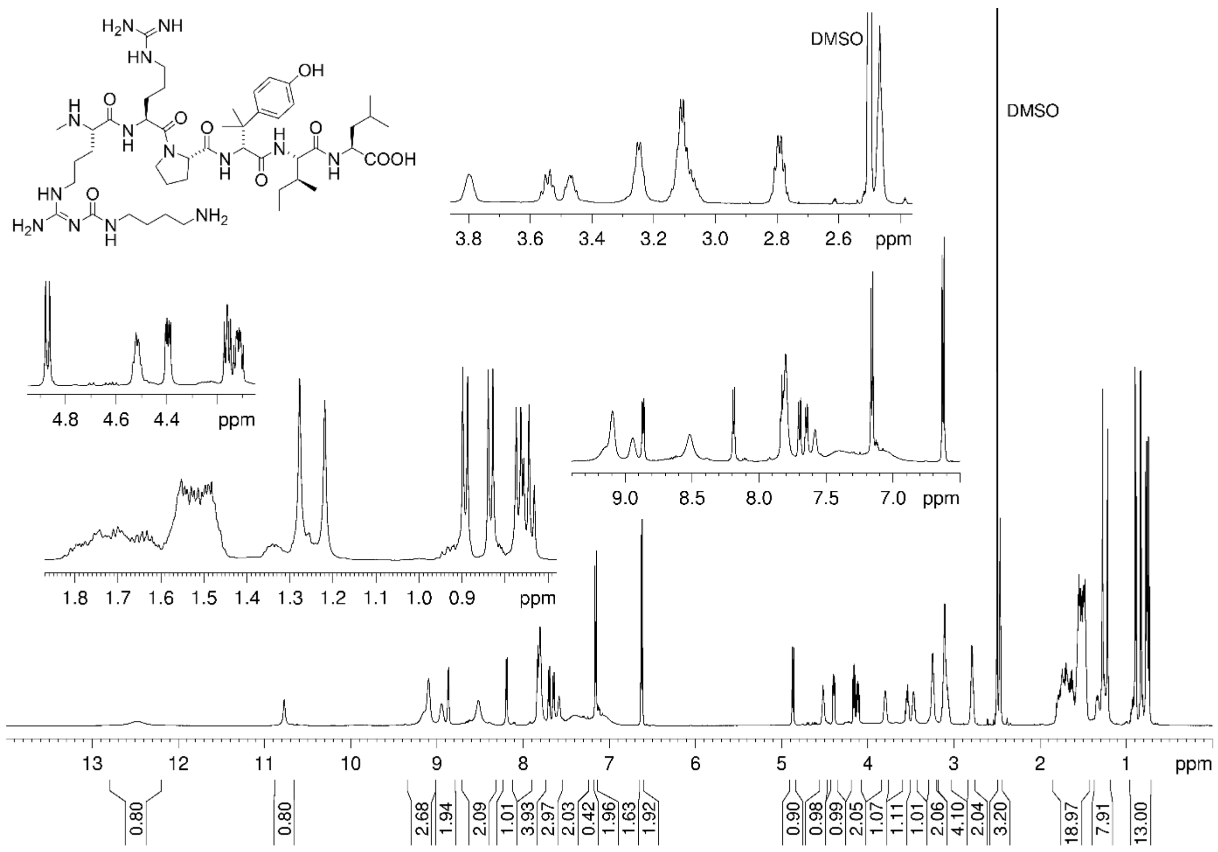

^{1}H-NMR spectrum (600 MHz, DMSO-d_6) of compound **51**

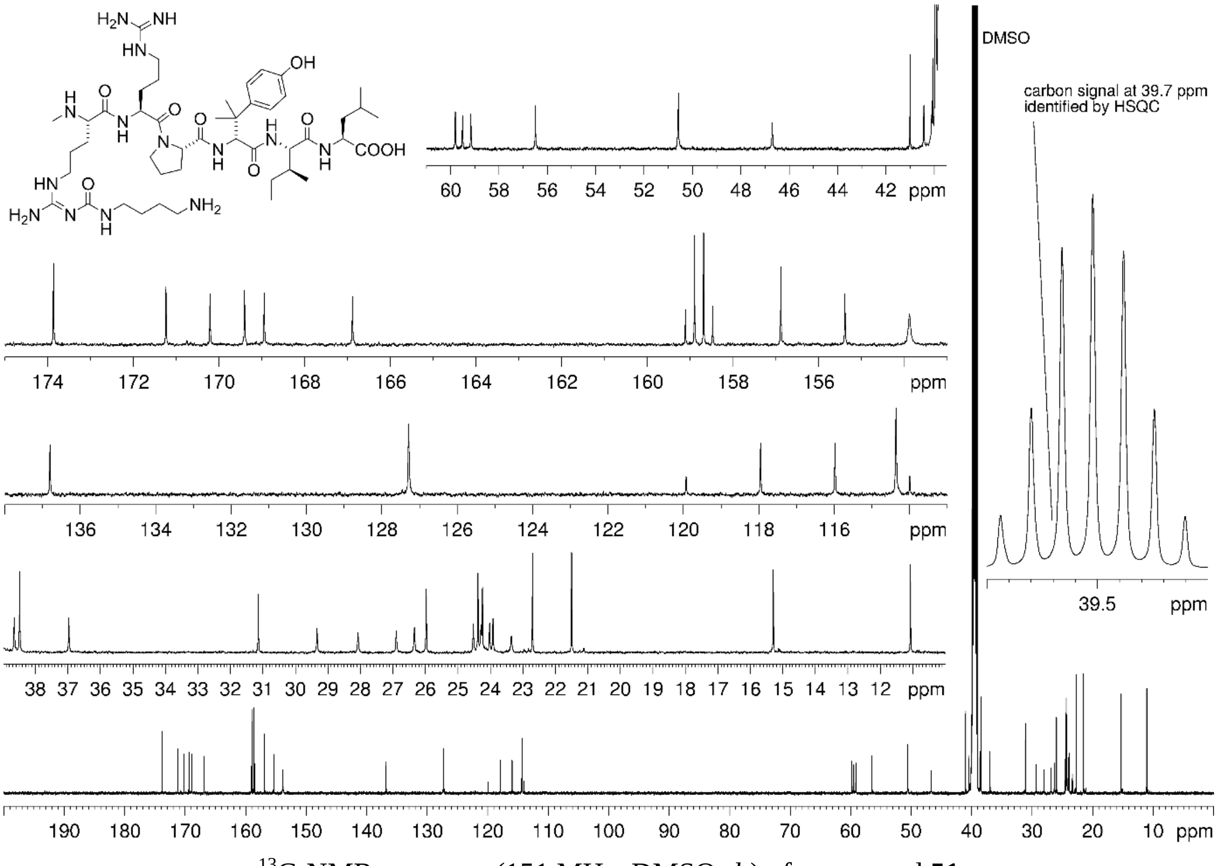

^{13}C-NMR spectrum (151 MHz, DMSO-d_6) of compound **51**

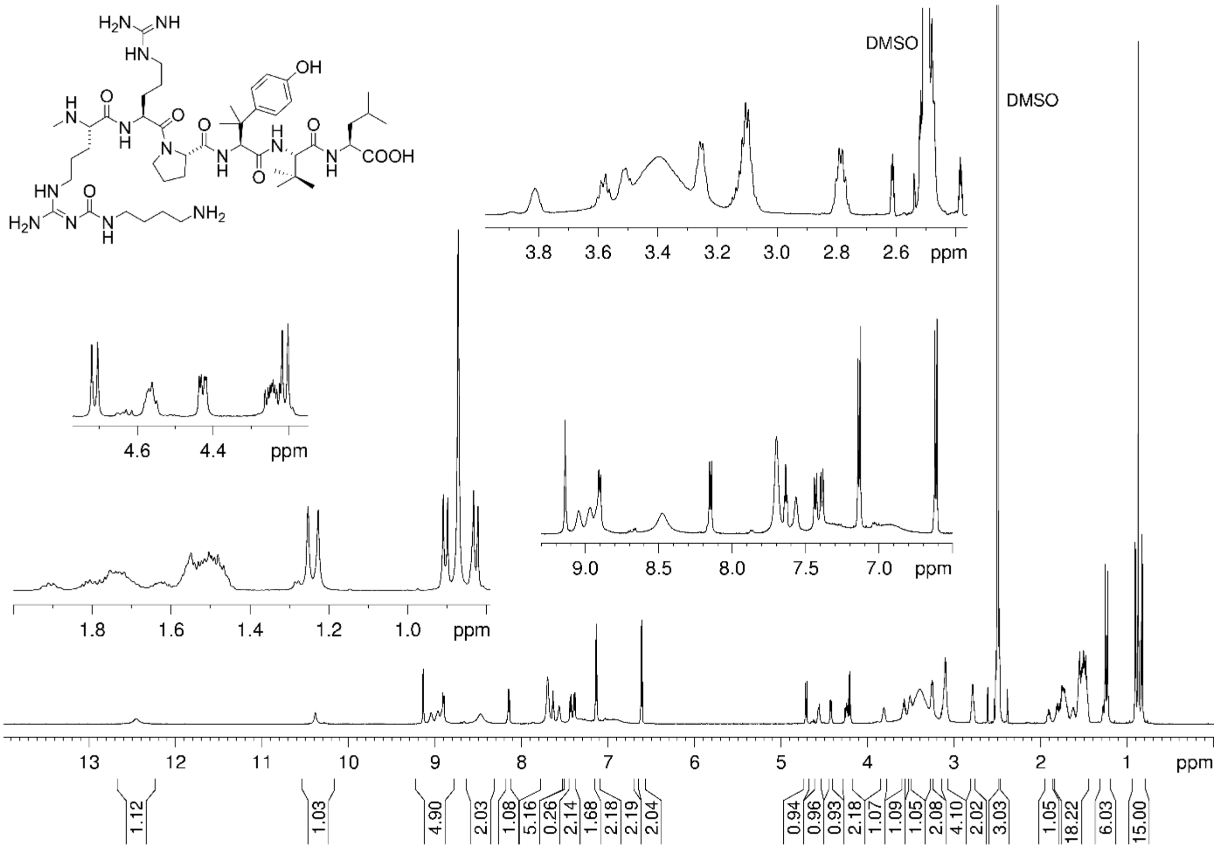

^{1}H-NMR spectrum (600 MHz, DMSO-d_6) of compound **52**

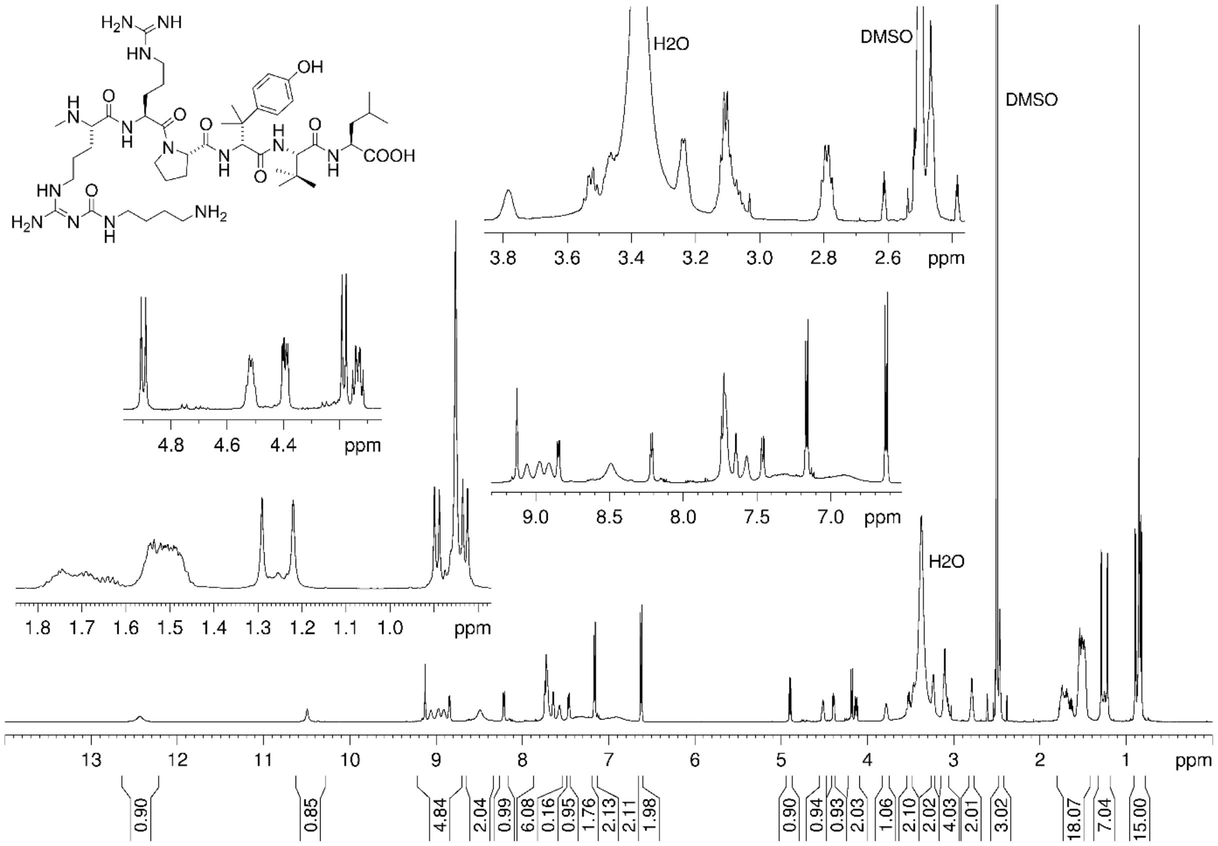

^{1}H-NMR spectrum (600 MHz, DMSO-d_6) of compound **53**

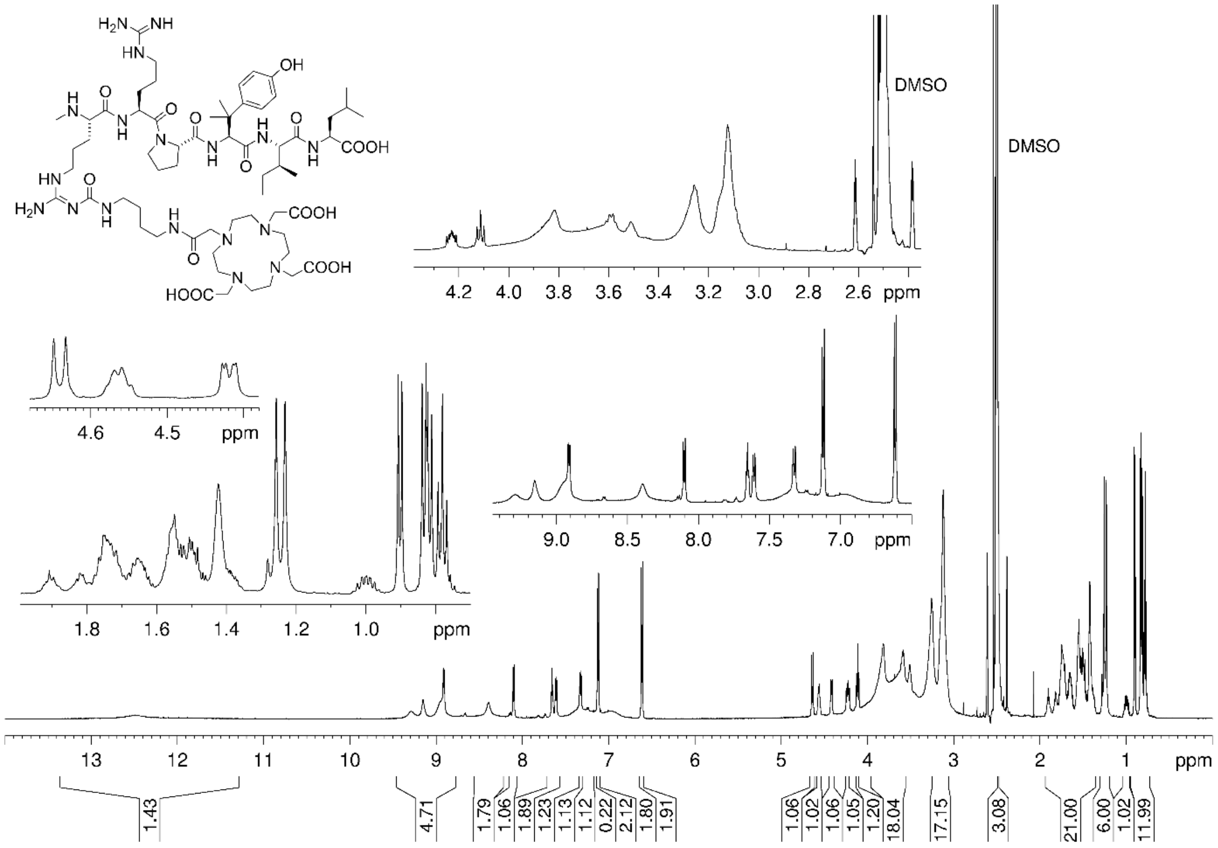

^{1}H-NMR spectrum (600 MHz, DMSO-d_6) of compound **54**

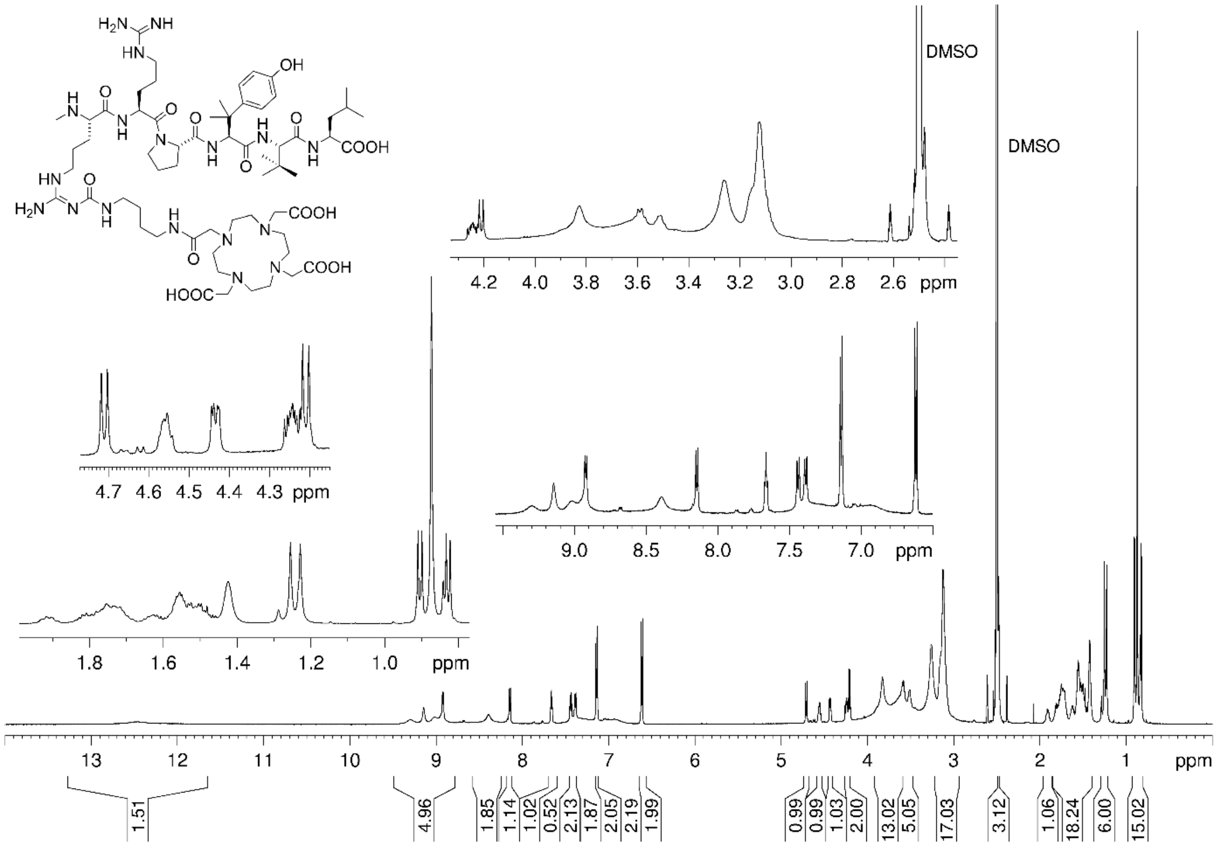

^{1}H-NMR spectrum (600 MHz, DMSO-d_6) of compound **55**

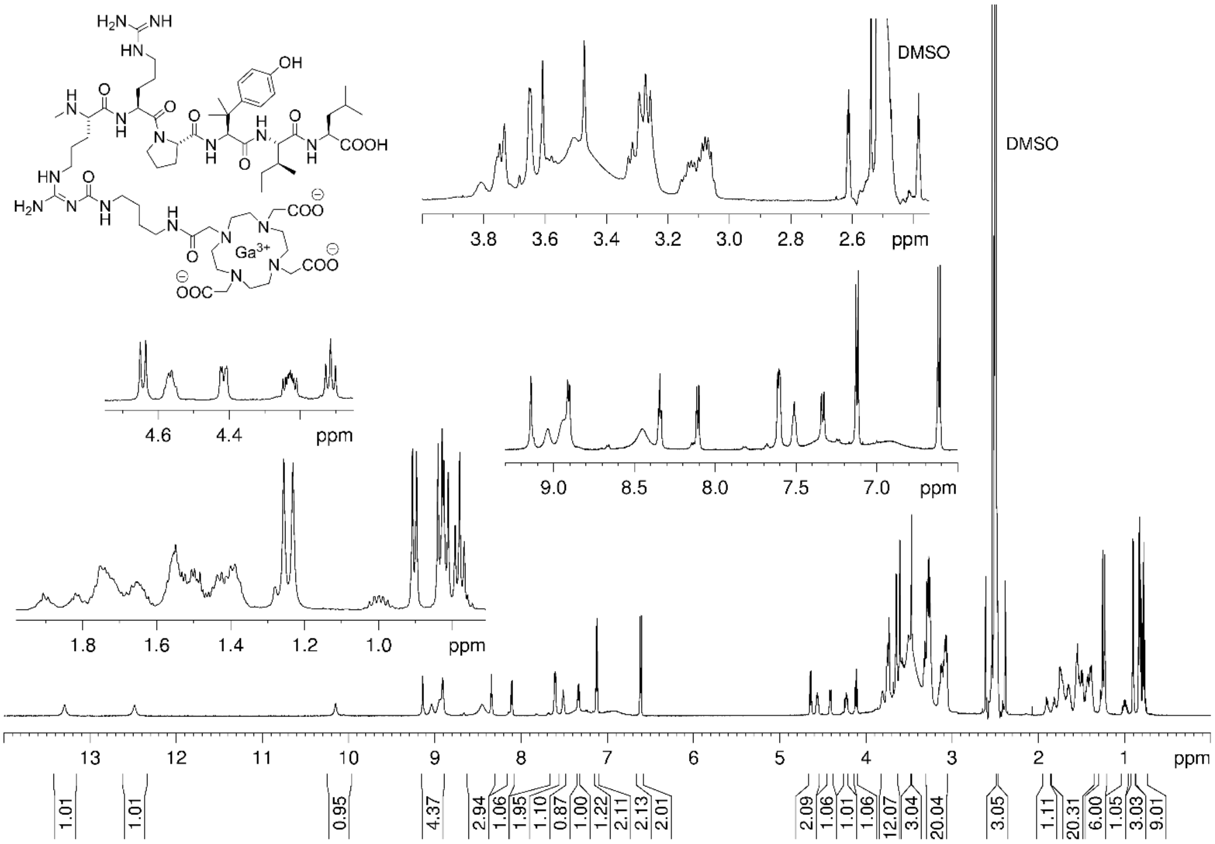

^{1}H-NMR spectrum (600 MHz, DMSO-d_6) of compound **56**

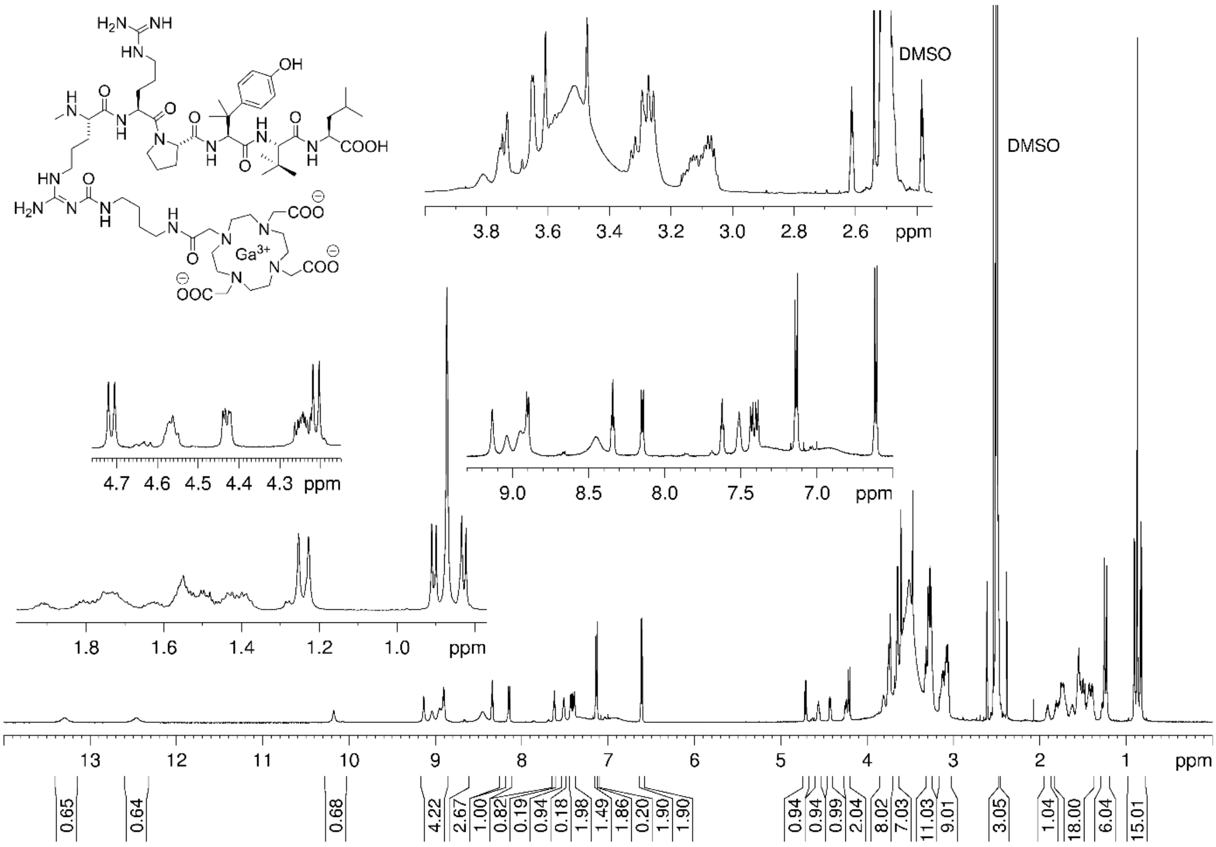

^{1}H-NMR spectrum (600 MHz, DMSO-d_6) of compound **57**

5. References

1. Keller, M.; Kuhn, K.K.; Einsiedel, J.; Hübner, H.; Biselli, S.; Mollereau, C.; Wifling, D.; Svobodová, J.; Bernhardt, G.; Cabrele, C.; et al. Mimicking of arginine by functionalized *N*$^{\omega}$-carbamoylated arginine as a new broadly applicable approach to labeled bioactive peptides: high affinity angiotensin, neuropeptide Y, neuropeptide FF, and neurotensin receptor ligands as examples. *J Med Chem* **2016**, *59*, 1925-1945, doi:10.1021/acs.jmedchem.5b01495.
2. Schindler, L.; Bernhardt, G.; Keller, M. Modifications at Arg and Ile give neurotensin(8-13) derivatives with high stability and retained NTS_1 receptor affinity. *ACS Med Chem Lett* **2019**, *10*, 960-965, doi:10.1021/acsmedchemlett.9b00122.
3. Schindler, L.; Wohlfahrt, K.; Gluhacevic von Krüchten, L.; Prante, O.; Keller, M.; Maschauer, S. Neurotensin analogs by fluoroglycosylation at *N*$^{\omega}$-carbamoylated arginines for PET imaging of NTS1-positive tumors. *Sci Rep* **2022**, *12*, 15028, doi:10.1038/s41598-022-19296-0.
